# Identification and Characterization of Wor4, a New Transcriptional Regulator of White-Opaque Switching

**DOI:** 10.1534/g3.115.024885

**Published:** 2016-01-13

**Authors:** Matthew B. Lohse, Alexander D. Johnson

**Affiliations:** * Department of Microbiology and Immunology, University of California, San Francisco, California 94158; † Department of Biochemistry and Biophysics, University of California, San Francisco, California 94158

**Keywords:** white-opaque switching, transcriptional regulation, transcription networks, transcriptional circuits, *Candida albicans*

## Abstract

The human fungal pathogen *Candida albicans* can switch between two cell types, “white” and “opaque,” each of which is heritable through many cell divisions. Switching between these two cell types is regulated by six transcriptional regulators that form a highly interconnected circuit with multiple feedback loops. Here, we identify a seventh regulator of white-opaque switching, which we have named Wor4. We show that ectopic expression of Wor4 is sufficient to drive switching from the white to the opaque cell type, and that deletion of Wor4 blocks switching from the white to the opaque cell type. A combination of ectopic expression and deletion experiments indicates that Wor4 is positioned upstream of Wor1, and that it is formally an activator of the opaque cell type. The combination of ectopic expression and deletion phenotypes for Wor4 is unique; none of the other six white-opaque regulators show this pattern. We determined the pattern of Wor4 binding across the genome by ChIP-seq and found it is highly correlated with that of Wor1 and Wor2, indicating that Wor4 is tightly integrated into the existing white-opaque regulatory circuit. We previously proposed that white-to-opaque switching relies on the activation of a complex circuit of feedback loops that remains excited through many cell divisions. The identification of a new, central regulator of white-opaque switching supports this idea by indicating that the white-opaque switching mechanism is considerably more complex than those controlling conventional, nonheritable patterns of gene expression.

Cell fate decisions are often controlled by interlocking feedback loops composed of several transcriptional regulators (Hobert 2011); white-opaque switching in the human fungal pathogen *Candida albicans* provides a well-studied example of this phenomenon. A normal part of the human microbiota, *C. albicans* can cause a range of infections in individuals with compromised immune systems or implanted medical devices (Wey *et al.* 1988; Eggimann *et al.* 2003; Gudlaugsson *et al.* 2003). *C. albicans* can switch among a number of distinct morphological states, but the maintenance of most of these states requires the continuous presence of one or more signals from the environment. The “white” and “opaque” cell types are an exception to this requirement. Each cell type is stable for many cell divisions, and switching between them occurs rarely and stochastically, with roughly one switch every 10,000 cell divisions under standard laboratory conditions (Slutsky *et al.* 1987; Soll *et al.* 1993; Johnson 2003; Lohse and Johnson 2009; Soll 2009; Morschhäuser 2010). The white and opaque cell types are easily distinguishable at the single cell and colony levels (Figure 1A); in addition, the white and opaque cell types differ in expression of roughly 20% of the genome (Lan *et al.* 2002; Tuch *et al.* 2010; Hernday *et al.* 2013), in their ability to mate (Miller and Johnson 2002), in their preferred metabolites (Lan *et al.* 2002), in their responses to environmental cues (Rikkerink *et al.* 1988; Huang *et al.* 2009, 2010; Lohse *et al.* 2013), and in their interactions with the innate immune system (Kvaal *et al.* 1997, 1999; Geiger *et al.* 2004; Lohse and Johnson 2008; Sasse *et al.* 2013).

**Figure 1:**
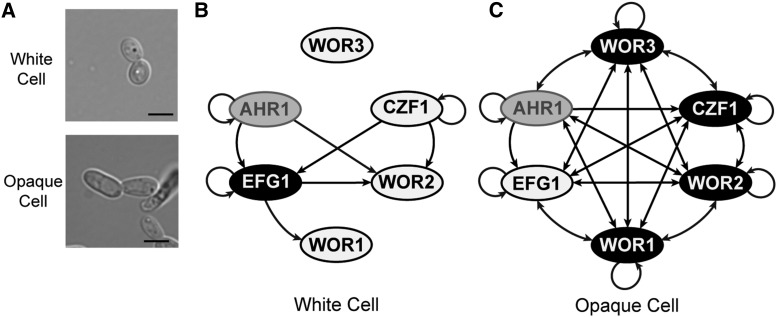
The white-opaque regulatory circuitry. (A) Typical white and opaque cells, each grown in the same liquid culture medium. Scale bar is 5 µm. (B), (C) Regulatory circuit in white (B) and opaque (C) cells based on binding events identified in previously published ChIP-chip studies of key regulators of switching (Zordan *et al.* 2007; Hernday *et al.* 2013; Lohse *et al.* 2013). Genes upregulated in a given cell type are shown in black with white text, with downregulated genes in white with black text. Genes that are not differentially regulated between cell types are shown in gray. Arrows represent direct binding interactions to the regulatory region of a given gene.

Regulation of white-opaque switching in *C. albicans* has been extensively studied, and six sequence-specific DNA binding proteins (Wor1, Wor2, Wor3, Czf1, Efg1, and Ahr1) have been identified as “core” regulators of this switch (Figure 1, B and C) (Huang *et al.* 2006; Srikantha *et al.* 2006; Zordan *et al.* 2006, 2007; Vinces and Kumamoto 2007; Wang *et al.* 2011; Hernday *et al.* 2013; Lohse *et al.* 2013). Of these six, Wor1 appears to be the most important, and it is often considered the “master regulator” of switching. It is upregulated 40-fold in opaque cells, and ectopic Wor1 expression in white cells is sufficient to drive switching, *en masse*, to the opaque cell type (Huang *et al.* 2006; Zordan *et al.* 2006). Conversely, deletion of Wor1 completely blocks switching to the opaque cell type, locking cells in the white form (Huang *et al.* 2006; Zordan *et al.* 2006). Wor2, Wor3, and Czf1 are up-regulated in opaque cells compared to white cells (but to lesser extents than Wor1), and function in the establishment and maintenance of the opaque cell type in a Wor1-dependent manner (Vinces and Kumamoto 2007; Zordan *et al.* 2007). For example, deletion of Czf1 reduces the rate of switching to the opaque cell type (establishment), but the resulting opaque cells are still stable for many generations (maintenance). Efg1, a white-enriched gene (that is, a gene whose expression is higher in the white cell type than the opaque cell type) has a role opposite to that of Wor1; it is needed for switching from the opaque cell type back to the white cell type, and for maintenance of the white cell type (Sonneborn *et al.* 1999; Zordan *et al.* 2007; Hernday *et al.* 2013). The sixth regulator, Ahr1, which is not differentially regulated between white and opaque cells, helps to repress the white-to-opaque switch in an Efg1-dependent manner (Wang *et al.* 2011; Hernday *et al.* 2013). These six regulators form an interlocking circuit in which each regulator controls the expression of the other regulators. In addition, each regulator controls a series of “target” genes whose differential expression gives white and opaque cells their unique properties (Hernday *et al.* 2013).

In this paper, we identify and characterize a new regulator of white-opaque switching, orf19.6713, which we have named Wor4. By studying the effects of deleting and overexpressing Wor4, we show that it plays a key role in both the establishment and maintenance of the opaque cell type, yet its effects are different from those of any of the previously identified regulators. Through ChIP-seq experiments, we show that Wor4 is highly integrated into the existing white-opaque regulatory circuit; the majority of the genomic sites to which it is bound overlap with those of the previously known white-opaque regulators.

## Materials and Methods

### Media

All strains were grown on synthetic complete media supplemented with 2% glucose and 100 µg/ml uridine (SD+aa+uri) at room temperature unless otherwise noted.

### Plasmid construction

A list of oligonucleotides used in this study can be found in Supporting Information, Table S1. A list of plasmids used in this study can be found in Table S2.

The mCherry tagging plasmid template pMBL180 was constructed as follows. The *mCherry* DNA sequence (Shaner *et al.* 2004) was modified to account for *C. albicans* codon usage and alternative coding of CTG (Lloyd and Sharp 1992), and synthesized by DNA2.0. The codon-optimized *mCherry* was inserted into pUC19 between the *Hin*dIII and *Pst*I sites. The *SAT1* selectable marker from pNIM1 (Park and Morschhäuser 2005) was amplified, then inserted between the *Pst*I and *Bam*HI sites.

The recyclable *SAT1* GFP tagging plasmid template pADH76 was constructed by introducing the *C. albicans* optimized *GFP* (Cormack *et al.* 1997) into the *Ava*I site of the recyclable *SAT1* marker plasmid pSFS2a (Reuss *et al.* 2004), along with an N-terminal flexible linker region. The orientation of *GFP* was selected to be such that the c-terminal end of *GFP* falls adjacent to one of the recombination sites.

The p*TDH3* ectopic expression plasmid pADH57 was created by amplifying the *TDH3* promoter region from pCJN542 (Nobile *et al.* 2008), and using it to replace the region between *Xba*I and *Bam*HI, containing the *MET3* promoter, from pADH33 (Lohse *et al.* 2013).

*WOR4* (pMBL640) and *RFG1* (pMBL639) p*MET3* ectopic expression plasmids were constructed using the previously reported pADH33 background. Full ORFs for both genes were inserted between the *Bam*HI and *Xma*I sites in pADH33. The *WOR4* ectopic expression *TDH3* plasmid (pMBL707) was created by inserting the full *WOR4* ORF between the *Bam*HI and *Pst*I sites in pADH57. The p*MET3 WOR1* ectopic expression plasmid (pADH35) has been previously reported (Hernday *et al.* 2013).

### Strain construction

A list of strains used in this study can be found in Table S3.

*WOR4* was deleted in the SN152 (a/α his– leu– arg–) strain background using the *HIS1* and *LEU2* cassettes as previously reported (Noble and Johnson 2005). This strain was then converted to the a/Δ background using pJD1 (Lin *et al.* 2013). *WOR4* heterozygous strains were constructed in the RZY47 background (Zordan *et al.* 2006)(a/a his– leu–) using the *HIS1* cassette. The *rfg1* deletion was constructed in the RZY47 background using the *HIS1* and *LEU2* cassettes. Wild type controls have been previously reported (Hernday *et al.* 2013); in brief, the *HIS1* and *LEU2* markers were added back to SN152 and RZY47. The *wor1* deletion strain, RZY219, has been previously reported (Zordan *et al.* 2006).

Ectopic expression strains were created by linearizing pADH35, pMBL639, pMBL640, and pMBL707 with *Nco*I and transforming into AHY135. Linearized pADH33 and pADH57 with no insert were used as negative controls.

The Wor4-GFP tagging cassette was generated by PCR amplifying *GFP-SAT1* from pADH76. Wor4 was tagged in both the wild type and heterozygous *wor4* deletion strains, the heterozygous strain was used to assess Wor4-GFP functionality. The Wor4-13x myc tagging cassette was created by amplifying the 13x *myc-SAT1* cassette from pADH34 (Hernday *et al.* 2010). In both cases, the *SAT1* marker was recycled as previously reported. HTB1 and HTB2 *mCherry-SAT1* tagging cassettes were PCR amplified from pMBL180.

### White-to-opaque and opaque-to-white switching assays

Plate-based quantitative white-opaque switching, and ectopic expression assays were performed as previously described (Miller and Johnson 2002; Zordan *et al.* 2007). For the quantitative white-opaque switching assay reported in Table 2, strains were grown for 7 d at room temperature. Five entirely white or opaque colonies were resuspended in water, diluted, plated on SD+aa+uri, allowed to grow for 7 d at room temperature, and scored for switching events by monitoring (A) colonies of the starting cell type, (B) colonies of the starting cell type with one or more sectors, and (C) colonies of the other cell type. We have defined switching frequency as 100 × (B + C) / (A + B + C). When no switching events were detected for a strain, we have reported the switching frequency as less than one divided by the number of colonies counted.

Strains for ectopic expression assays, as reported in Table 1, the white-to-opaque portion of Table 3, and Table S4, were grown for 7 d on repressing medium (+Met +Cys). Five entirely white or opaque colonies were resuspended in water, diluted, and then plated on either repressing or inducing (–Met –Cys) medium, allowed to grow for 7 d at room temperature, and scored with the empty vector strains used as negative controls. Switching frequencies were calculated as described above.

**Table 1:** Ectopic expression of *WOR4* induces white-to-opaque switching

White-to-Opaque Switching				
Strain	Media Condition	Switching Frequency (%)	n	Notes*^a^*
p*MET3*-Blank	Repressing	<0.38	266	1
p*MET3*-Blank	Inducing	<0.40	253	1
p*MET3-WOR1*	Repressing	0.40	148	2
p*MET3-WOR1*	Inducing	100.00	162	2
p*MET3-WOR4*	Repressing	<0.38	264	
p*MET3-WOR4*	Inducing	31.53	203	
p*MET3-RFG1*	Repressing	<0.37	269	
p*MET3-RFG1*	Inducing	<0.51	195	
Opaque-to-White Switching				
Strain	Media Condition	Switching Frequency (%)	*n*	Notes*^a^*
p*MET3*-Blank	Repressing	0.63	205	1
p*MET3*-Blank	Inducing	0.88	285	1
p*MET3-WOR4*	Repressing	<0.39	259	
p*MET3-WOR4*	Inducing	<1.39	72	
p*MET3-RFG1*	Repressing	1.17	309	
p*MET3-RFG1*	Inducing	0.85	117	

Switching frequency and number of colonies scored for ectopic expression of *WOR1*, *WOR4*, and *RFG1* under inducing and repressing conditions are indicated.

a 1, Negative control; 2, positive control.

The *wor4* deletion opaque stability assay, reported in the lower part of Table 3, modified the ectopic expression assay described above as follows. After growing for 7 d on inducing medium, representative opaque colonies were restreaked to repressing medium and allowed to grow until the morphology of colonies could be determined. Single opaque colonies were then resuspended in water, diluted, and plated on three repressing media plates and allowed to grow for 5–7 d. Two opaque colonies were independently processed for each strain (colony 1, colony 2). Colonies were then scored for white or opaque phenotypes as described above. Phenotypes were verified by microscopy of single cells taken from representative colonies. Each reported switching rate represents the average of the individual switching frequencies determined for the two colonies processed for a given strain in a given repeat [(colony 1 rate + colony 2 rate) / 2]. The total number of colonies scored (*n*) represents the sum of the colonies scored for the two parallel assays (*n* colony 1 + *n* colony 2). The four opaque-to white switching rates for the *wor4* deletions represent two switching assays conducted with each of two independent strains.

### Microscopy

Cell microscopy images were taken from overnight cultures (SD+aa+uri at room temperature) started from representative colonies. Microscopic images were taken using a Zeiss Axiovert 200M microscope.

### Western blotting

Samples for western blotting were prepared as follows. Room temperature SD+aa+uri overnights were started from 5- to 7-d-old colonies. Cultures were diluted back to OD600 = 0.067 in the morning, and allowed to regrow at 25° to an OD600 of ∼0.4, at which point 14 ml was harvested for each culture. Cells were pelleted, the supernatant discarded, and the pellet flash frozen in liquid nitrogen prior to storage at –80°.

Extracts were prepared in Urea Lysis buffer (Ubersax *et al.* 2003); 10 µl of each sample was run on an SDS-PAGE gel, and then analyzed by Western Blotting. Blots were analyzed with mouse α-human c-myc (AHO0062, Invitrogen). Rat α-tubulin (ab1616; Abcam) was used as a loading control. Secondary antibodies were goat α-mouse IrDye800 (610-132-121, Rockland Immunochemicals Inc.), and goat α-rat AlexaFluor680 (A-21096, Invitrogen). Blots were scanned on an Oddessey Imaging System scanner (LI-COR Biotechnology).

### ChIP-seq

Three independent samples were grown, processed, and sequenced for the tagged (experimental) and untagged (control) strains in both cell types. Samples for ChIP-seq were harvested and processed as follows, adapted from previous protocols (Hernday *et al.* 2010; Nobile *et al.* 2012; Lohse *et al.* 2016). Room temperature SD+aa+uri overnights were started from 5- to 7-d-old colonies. Cultures were diluted back in 200 ml to OD600 = 0.067 in the morning, and allowed to regrow at 25° to an OD600 of ∼0.4. In brief, crosslinking was done for 15 min, cells were lysed by vortexing for 2 hr with glass beads, and the chromatin was shredded by 2 × 15-min rounds of sonication in a Bioruptor (30 s on, 60 s off). For each ChIP, we used 5 µl of Living Colors Full-Length GFP Polyclonal Antibody (Clontech 632592), and Protein A-Sepharose 4B Fast Flow beads (Sigma P9424). Following protease treatment and crosslinking reversal, samples were purified and concentrated using a Qiagen MinElute Kit. Libraries for sequencing were prepared using the NEBNext ChIP Library Prep Master Mix set for Illumina with the NEBNext Multiplex Oligos for Illumina. Samples were pooled and sequenced (50 bp, single end reads) on a HiSequation 2500 at the UCSF Center for Advanced Technology.

Sequences were mapped to the *C. albicans* genome using Bowtie2 (Langmead and Salzberg 2012) with the default settings. Peaks were called using Macs2 (Zhang *et al.* 2008) with the shift size determined by the SPP R Bioconductor package for ChIP-seq analysis (Kharchenko *et al.* 2008). Initial Macs2 peak calls were further processed as follows. For the white cell Wor4-GFP dataset, we narrowed our dataset down to peaks called in at least two (out of three) replicates. The finalized peak locations reflect the union of the locations of the peaks from the individual replicates. We then culled locations that overlapped over our previously published so-called “red flag location” list, ORFs, or tRNA, leaving a set of nine peaks (Hernday *et al.* 2013; Lohse *et al.* 2013). For the opaque cell Wor4-GFP dataset, we eliminated all peaks with a q-score of over 10 (as calculated by Macs2). We then took the overlap of the three repeats, giving a total of 301 peaks. The finalized peak locations reflect the union of the locations of the peaks from the individual replicates. We then culled out locations that overlapped over our previously published so-called “red flag location” list, ORFs, or tRNA, leaving a total of 244 peaks. For both white and opaque cells, peaks were mapped to intergenic regions, and assigned to the neighboring genes. The enrichment values reported for a given peak represent the median Macs2 fold enrichment value of the corresponding peaks from the individual replicates. The enrichment values for the promoter upstream of a given gene represent this median value if only one peak was present in the intergenic region. If multiple peaks were present in the intergenic region, the largest of the median values from the individual peaks was used. Gene assignments, evaluation of overlap with previously published data sets, and determination of median Macs2 fold enrichment values were performed using MochiView v1.46 (Homann and Johnson 2010).

File S1 contains a list of the genomic locations and median fold enrichment of the Wor4-GFP peaks in both white and opaque cells. File S2 contains MochiView image plots of 12 kb regions centered on the sets of Wor4 binding sites in white and opaque cells. File S3 contains a list of genes whose control regions are bound by Wor4, as well as a summary of previously published chromatin immunoprecipitation and transcriptional profiling experiments for the other six regulators of white-opaque switching.

### qPCR

Samples for qPCR were prepared as follows. Room temperature SD+aa+uri overnights were started from 5- to 7-d-old colonies. Cultures were diluted back to OD600 = 0.2 in the morning, and allowed to regrow at 25° to an OD600 of ∼0.8. The equivalent of 10 ml at OD600 = 1 was harvested for each culture. Cells were pelleted, the supernatant discarded, and the pellet flash frozen in liquid nitrogen prior to storage at –80°. Cells from the culture were plated on SD+aa+uri plates and grown for 7 d at room temperature to verify the cell type composition of each culture.

RNA was extracted from pellets using the Ambion RiboPure Yeast Kit with the DNase I treatment step. Superscript II RT (Invitrogen) was used for cDNA synthesis. We used Power SYBR Green mix (Life Technologies) for all qPCR reactions. Three +RT and one –RT reaction were run for each strain; 5 µl of a 1:100 dilution was used in each well. A 1:10 dilution of an equal volume mixture of all +RT reactions was used as the starting point for the standard curve, which consisted of six 1:4 dilutions from the starting sample. *DYN1* was used as a control.

### Wor4 bioinformatics

The Wor4 protein sequence was analyzed using the ELM (Dinkel *et al.* 2014) and HHpred (Söding *et al.* 2005) servers. Wor4 homologs in fungal species were identified using the Fungal Genomes Search tool at the Saccharomyces Genome Database (Cherry *et al.* 2012) using the BLASTP tool (except for *Wickerhamomyces* *anomalus* which used the TBLASTN tool) (Altschul *et al.* 1990). The protein sequence for the best hit in a given species was then compared to the *C. albicans* genome using the BLASTP tool at the Candida Genome Database (Inglis *et al.* 2012). A Wor4 homolog was considered to be present in a given species if both BLAST searches gave a hit with an E-value better than 1e-5 and if Wor4 was the highest scoring hit for the *C. albicans* genome.

### Data availability

Strains and plasmids are available on request. File S1 contains a list of the genomic locations and median fold enrichment of the Wor4-GFP peaks in both white and opaque cells. File S2 contains MochiView image plots of 12 kb regions centered on the sets of Wor4 binding sites in white and opaque cells. File S3 contains a list of genes whose control regions are bound by Wor4 as well as a summary of previously published chromatin immunoprecipitation and transcriptional profiling experiments for the other six regulators of white-opaque switching. FASTA and bedgraph files for Genome-wide chromatin immunoprecipitation experiments are available at NCBI Geo, Accession Number GSE75124.

## Results

### Ectopic expression of orf19.6713 drives switching to the opaque cell type

In opaque cells, each of the six known regulators of white-opaque switching binds to the upstream regions of 11 genes coding for putative transcriptional regulators (Zordan *et al.* 2007; Hernday *et al.* 2013; Lohse *et al.* 2013). We reasoned that these were strong candidates for being central regulators of white-opaque switching, as deletion or ectopic expression of five of these regulators (*WOR1*, *WOR2*, *WOR3*, *CZF1*, *EFG1*) has been previously reported to affect white-opaque switching (Sonneborn *et al.* 1999; Huang *et al.* 2006; Srikantha *et al.* 2006; Zordan *et al.* 2006, 2007; Vinces and Kumamoto 2007; Lohse *et al.* 2013). An additional three of these regulators do not affect switching rates when deleted (*AAF1*, *BRG1*, *CRZ2*) (Hernday *et al.* 2013). [The effect of a fourth gene (*NRG1*) has been difficult to assess because the deletion causes cells to become hyperfilamentous, resembling neither white nor opaque cells (Braun *et al.* 2001; Murad *et al.* 2001; Si *et al.* 2013).] The remaining two regulators, *orf19.6713* (C3_07730W) and *RFG1* (orf19.2823, CR_02640W), had not previously been examined in the context of white-opaque switching, and we tested them for such a role.

We first ectopically overexpressed *orf19.6713* and *RFG1* using the p*MET3* system. Ectopic expression of *RFG1* in white cells or opaque cells did not produce any observable effect on white-to-opaque or opaque-to-white switching. Ectopic expression of *orf19.6713* in white cells, in contrast, caused a significant increase in switching from the white to the opaque cell type (Table 1). Specifically, we observed the formation of opaque sectors in many (20–40%) of the colonies compared with < 0.5% without ectopic expression. This behavior is similar to that of *CZF1*, whose ectopic expression results in opaque sectors in most colonies (Vinces and Kumamoto 2007; Zordan *et al.* 2007). Ectopic expression of *orf19.6713* using the p*TDH3* system, which expresses at a higher level than p*MET3*, resulted in switching to fully opaque colonies rather than opaque sectors. Switching by ectopic expression of *orf19.6713* was blocked in a *wor1* deletion strain, indicating that orf19.6713 functions upstream of Wor1 (Table S4). Given *orf19.6713*’s strong effect on switching, we have named it *WOR4* (White-Opaque Regulator 4).

### Deletion of WOR4 reduces or eliminates switching to the opaque cell type

We next determined whether the *rfg1* or *wor4* deletions affected white-opaque switching. Deletion of *RFG1* did not significantly change white-to-opaque switching rates, and showed a only a small increase in opaque-to-white switching rates (Figure S1A). In contrast, deletion of *WOR4* completely blocked white-to-opaque switching (Table 2), locking cells in the white form (Figure S1A). Specifically, we screened more than 8000 colonies from two independently constructed *wor4* deletion strains, and never observed an opaque sector or colony. Deletion of one copy of *WOR4* (to create a heterozygous strain) did not notably affect the white-to-opaque switching frequency (0.70x wild type) but did slightly increase opaque-to-white switching (3.02x wild type) (Table 2), further suggesting a role maintaining the opaque cell type. Given the relatively small effects of the *RFG1* gene ectopic expression and deletion strains, we will not consider it further in this paper.

**Table 2:** Deletion of *WOR4* blocks white-to-opaque switching

White-to-Opaque Switching			
Strain	Switching Frequency (%)	*n*	Notes*^a^*
Wild Type White	3.51	541	1
Wild Type White	2.30	609	2
*rfg1/rfg1*	3.80	527	1
*WOR4/wor4*	2.47	511	1
*wor4/wor4*	<0.06	1809	2
*wor4/wor4*	<0.06	1804	2
Opaque-to-White Switching			
Strain	Switching Frequency (%)	*n*	Notes*^a^*
Wild Type Opaque	10.29	408	1
*rfg1/rfg1*	31.25	480	1
*WOR4/wor4*	31.14	517	1

Switching frequency and number of colonies scored for heterozygous and homozygous deletions of *WOR4* and homozygous deletions of *RFG1*. Parental strains corresponding to the strain background of each strain are indicated.

a 1, Common strain background; 2, Common strain background.

The apparent inability of the *wor4* deletion to form opaque cells is similar to the effect seen for the *wor1* and *wor2* deletions (Huang *et al.* 2006; Srikantha *et al.* 2006; Zordan *et al.* 2006, 2007). Ectopic expression of *WOR1*, however, can overcome both of those deletions, and allow for the formation of opaque cells. When the ectopic source of *WOR1* was removed, however, the cells reverted to the white cell type, meaning that the endogenous Wor1 and Wor2 were needed to maintain the opaque state. Similar to the *wor1* and *wor2* deletions, ectopic expression of *WOR1* in a *wor4* deletion background produced opaque colonies (Table 3 and Figure S1B). Unlike the *wor1* or *wor2* deletions, however, a portion of these cells remained opaque even after the ectopic *WOR1* expression was ended. These *wor4* deletion opaques were not as stable as normal opaques (60–70% switching to white *vs.* ∼10%), but some cells and colonies remained opaque during multiple passages on liquid or solid media (Table 3 and Figure S1C). This observation provides additional support for the conclusion that Wor4, in addition to being required for white-to-opaque switching, is also required for the stability of the opaque state through rounds of cell division.

**Table 3:** Ectopic expression of *WOR1* can overcome the effects of the *wor4* deletion

White-to-Opaque Switching				
Strain	Media Condition	Switching Frequency (%)	*n*	Notes*^a^*
Wild Type, p*MET*3-Blank	Repressing	<0.60	168	1
Wild Type, p*MET*3-Blank	Inducing	<0.32	308	1
Wild Type, p*MET*3-*WOR1*	Repressing	<0.47	214	2
Wild Type, p*MET*3-*WOR1*	Inducing	98.96	384	2
*wor4/wor4*, p*MET3*-Blank	Repressing	<0.82	122	
*wor4/wor4*, p*MET3*-Blank	Inducing	<0.31	327	
*wor4/wor4*, p*MET3-WOR1*	Repressing	<0.74	136	
*wor4/wor4*, p*MET3-WOR1*	Inducing	100.00	315	
*wor4/wor4*, p*MET3-WOR1*	Repressing	<0.49	203	
*wor4/wor4*, p*MET3-WOR1*	Inducing	99.54	434	
Opaque-to-White Switching				
Strain	Media Condition	Switching Frequency (%)	*n*	Notes*^a^*
Wild Type, p*MET*3-Blank	Repressing	11.95	626	
Wild Type, p*MET*3-*WOR1*	Repressing	6.71	472	
*wor4/wor4*, p*MET3-WOR1*	Repressing	63.13	623	
*wor4/wor4*, p*MET3-WOR1*	Repressing	64.46	436	
*wor4/wor4*, p*MET3-WOR1*	Repressing	76.00	642	
*wor4/wor4*, p*MET3-WOR1*	Repressing	70.01	670	

Opaque-to-white switching on the p*MET3-WOR1 wor4* deletion strain was performed using opaque colonies from the white-to-opaque induction assay following several days growth on repressing media to repress *WOR1* expression. As explained in the text, the *wor4* deletion opaque cells are less stable than normal opaque cells.

a 1, Negative control; 2, positive control.

As with the *WOR4* ectopic expression results, the *wor4* deletion results suggest that Wor4 is positioned upstream of Wor1, and that it is formally an activator of the opaque cell type. The combination of ectopic expression and deletion phenotypes for *WOR4* is unique; none of the other white-opaque regulators show this pattern. In effect, Wor4 contributes to both the establishment and the stable maintenance of the opaque cell type, while the other regulators (excepting Wor1) appear dedicated to one or the other of these roles.

### Wor4 expression and localization are similar in the white and opaque cell types

Although Wor4 was annotated in the *Candida* genome sequence as a putative transcriptional regulator, little is known about it. *WOR4* transcription is not differentially regulated between white and opaque cell types (Tuch *et al.* 2010; Hernday *et al.* 2013), but we do not know whether translational or post-translational regulation might produce differential protein expression. To address these issues, we created a C-terminal GFP tagged Wor4 fusion. This fusion protein was functional, as a *WOR4-GFP/wor4* strain could form opaque cells.

**Figure 2:**
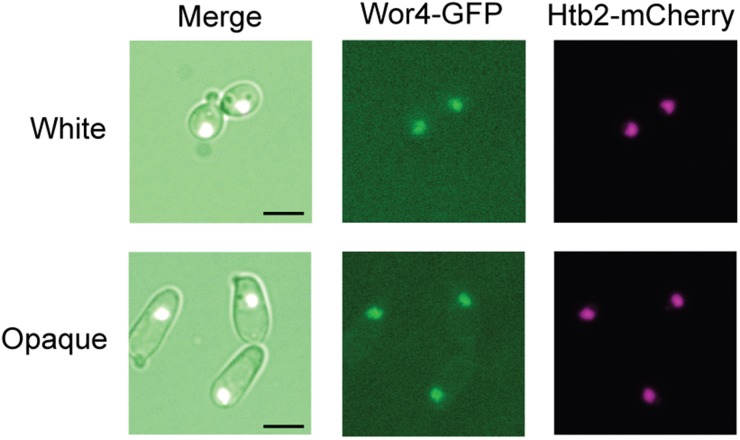
Wor4 localizes to the nucleus in both white and opaque cells. Visualization of Wor4-GFP and Htb2-mCherry fusion proteins in both white and opaque cells. Merged images (DIC, GFP, mCherry), GFP fluorescence, and mCherry fluorescence are shown. Scale bar is 5 µm.

Wor4-GFP colocalized with histone marker proteins (HTB1- and HTB2-mCherry fusions) in both cell types, indicating that it is nuclear localized (Figure 2 and Figure S2A). We did not observe dramatic changes in GFP intensity or localization on white-opaque switching, suggesting that any changes in Wor4 protein levels or localization between cell types is minor. Western blotting of a Wor4-13x myc fusion also indicated similar protein levels between the two cell types (opaque cell levels ∼1.5x those of white cells) (Figure S2B).

### Wor4 is highly integrated into the opaque cell regulatory network

**Figure 3:**
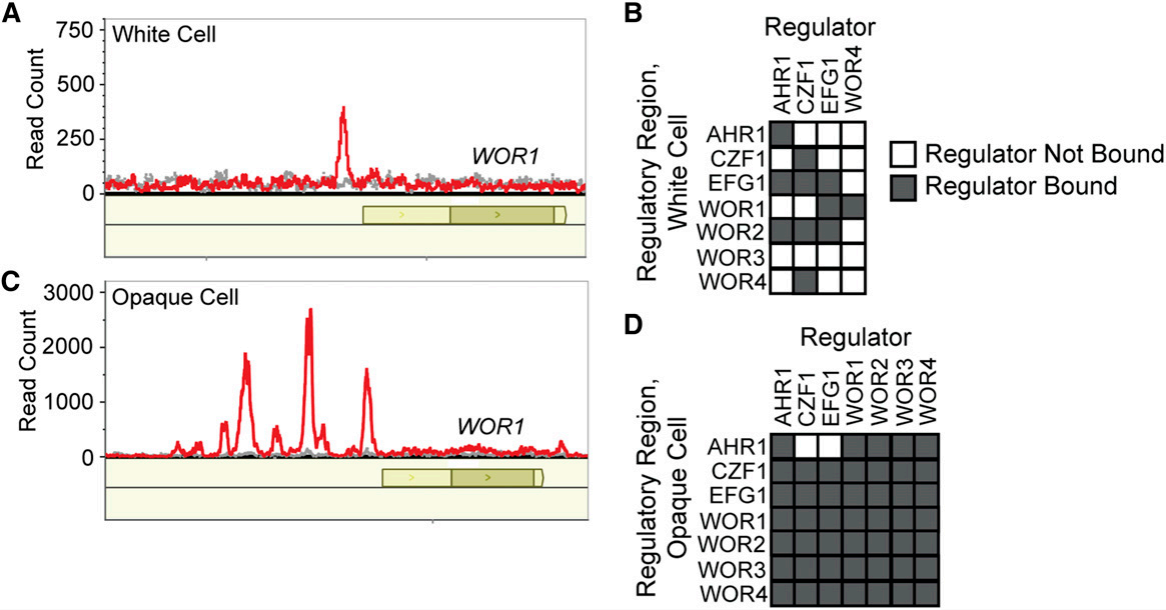
Wor4 localization across the genome as determined by ChIP-seq. (A) Wor4 binds to the upstream region of Wor1 in white cells. ChIP-seq reads for Wor4-GFP in red and the untagged control in gray. Open reading frames are represented as dark yellow boxes, and untranslated regions are represented in light yellow. Data are plotted using MochiView (Homann and Johnson 2010). (B) Regulatory circuit in white cells, incorporating Wor4 binding data from this study with previously published ChIP-chip studies of other regulators (Hernday *et al.* 2013). The charts indicate which regulators (columns) were enriched at the control region of each regulator (rows) (see Figure 1). (C) Wor4 binds to multiple positions in the upstream region of Wor1 in opaque cells. The data are displayed as in panel (A). (D) Regulatory circuit in opaque cells, incorporating Wor4 binding data from this study, as well as previously published ChIP-chip studies (Zordan *et al.* 2007; Hernday *et al.* 2013; Lohse *et al.* 2013).

We next performed ChIP-seq on the Wor4-GFP fusion in both white and opaque cells in order to determine its regulatory targets. In white cells, we identified nine peaks of Wor4 binding, which corresponded to nine intergenic regions controlling 13 genes (Figure 3A, File S1, File S2, and File S3). Of these 13 genes, three are differentially expressed by at least twofold between white and opaque cells based on our previously reported RNA-seq data (three opaque-enriched and no white-enriched genes) (Tuch *et al.* 2010). Seven of the nine intergenic regions bound by Wor4 are also bound by Ahr1, Czf1, or Efg1 in white cells, indicating that Wor4 is a part of the white cell regulatory network, consistent with its expression in white cells (Table S5) (Hernday *et al.* 2013). Among its 13 targets in white cells is *WOR1*, which supports the previous conclusion, based solely on genetic interactions, that Wor4 functions upstream of Wor1 (Figure 3, A and B). Although Wor4 binds upstream of *WOR1*, we observed only modest changes in *WOR1* transcript levels in the *wor4* deletion strain (1.3x and 2x increases relative to wild type by qPCR). It should be noted, however, that the levels of the *WOR1* transcript are very low in white cells; the main effect of Wor4 on regulation of *WOR1* probably occurs during the transition between white and opaque cells.

In opaque cells, the positions of Wor4 binding across the genome were greatly expanded relative to white cells (Figure 3C). We identified 244 Wor4 peaks corresponding to 137 intergenic regions upstream of 172 genes (File S1, File S2, and File S3). Of these 172 genes, 78 are differentially expressed by at least twofold between white and opaque cells based on our previously reported RNA-seq data (47 opaque-enriched, and 31 white-enriched genes) (Tuch *et al.* 2010). Of the 137 Wor4 bound intergenic regions, 133 are bound by at least one of the other core white-opaque transcriptional regulators, with approximately 90% of Wor4 bound intergenic regions having Wor1 or Wor2 also bound (Table S6) (Zordan *et al.* 2007; Hernday *et al.* 2013; Lohse *et al.* 2013). These results indicate that Wor4 is highly integrated into the existing opaque cell regulatory network. Consistent with this conclusion, we observed Wor4 peaks at the control regions of all the known white-opaque regulators (*WOR1*, *WOR2*, *WOR3*, *WOR4*, *AHR1*, *CZF1*, and *EFG1*) (Figure 3D).

### Wor4 is a C2H2 zinc finger with homologs in many fungal species

Both the Eukaryotic Linear Motif (ELM) (Dinkel *et al.* 2014), and the homology detection and structure prediction by HMM-HMM comparison (HHpred) (Söding *et al.* 2005) servers identify a 26- to 46-amino-acid region of the Wor4 protein (aa ∼175–221 in *C. albicans*) that is broadly conserved across much of the fungal domain. It is related by sequence to the C2H2 zinc finger family of proteins (ZnF_C2H2, P 5.7e-20 for top HHpred hit), the largest eukaryotic family of transcriptional regulators (Weirauch and Hughes 2011) (Figure S3A). Although members of the C2H2 family typically contain multiple instances of this zinc finger motif arranged in tandem (Stubbs *et al.* 2011), the Wor4 family appears to contain just a single zinc finger. Traditionally, a single instance of the C2H2 zinc finger recognizes a 3-bp DNA sequence; suggesting that Wor4 may recognize only a short DNA sequence (Wolfe *et al.* 2000). Our ChIP-seq experiments show clearly that Wor4 associates with specific sequences of DNA; the extent to which this occurs through association with other DNA-bound proteins (Wor1 and Wor2, most likely), or through Wor4’s intrinsic DNA-binding activity is not known. We stress that, regardless of its exact mode of recruitment, Wor4 is clearly bound to a majority of the intergenic sequences that are bound by the other key regulators of white-opaque switching.

Proteins with extensive sequence similarity to Wor4 exist in the great majority of fungal species. In addition to being found across the *Candida* or CTG clade, Wor4 homologs are found in many filamentous fungi as well as Basidiomycota and Mucoromycotina. However, Wor4 orthologs appear absent in a number of species, including *Saccharomyces cerevisiae*, *Kluyveromyces lactis*, and *Schizosaccharomyces pombe*. This distribution of Wor4 orthologs is consistent with an origin early in the fungal kingdom followed by several losses (Figure S3B), a pattern unlike that observed for the previously characterized regulators of white-opaque switching. *C. albicans* is the only species in which a biological role of the Wor4 class of proteins has been determined.

## Discussion

The regulatory circuit controlling white-opaque switching in *C. albicans* is complex, highly interconnected, and produces two cell types, each of which is stable over thousands of cell divisions. In this paper, we identified Wor4 as a new member of the white-opaque regulatory circuit, and demonstrated that it plays a role in both the establishment and the maintenance of the opaque cell type. ChIP-seq experiments indicate that Wor4 is highly integrated into the previously described white-opaque regulatory circuit, and its overlap with Wor1 and Wor2 binding suggests that it is recruited to these sites, at least in part, through interactions with these proteins. Consistent with this observation, we observed the DNA sequence recognized by Wor1 at many of the Wor4 peaks in opaque cells.

Wor4 differs in several ways from the previously reported regulators of white-opaque switching. The effects of *WOR4* ectopic expression and deletion do not phenocopy those of any of the previously identified regulators of the opaque cell type; more specifically, Wor4 is unusual in playing roles in both the establishment and the maintenance of the opaque cell type, which had only previously been seen for Wor1. Unlike Wor1, however, Wor4 does not appear to be differentially regulated or differentially localized between the two cell types. It is not yet clear how Wor4 functions in the regulation of switching. One hypothesis is that Wor4 functions through cooperative assembly on DNA with Wor1 and/or Wor2, perhaps stabilizing otherwise transient binding events to allow for stabilization of the opaque transcriptional circuit. Such a role for Wor4 is consistent with the phenotypes observed when Wor4 is deleted or over-expressed.

Although the white and opaque cell types are normally highly stable, with switching between them rare and stochastic, environmental cues can cause rapid mass switching between cell types. For example, a shift to 37° causes opaque cells to switch to white cells *en masse*, and the presence of N-acetylglucosamine causes the opposite shift (Rikkerink *et al.* 1988; Huang *et al.* 2010). We propose that the successive layering of transcriptional regulators (seven, so far) allows the switch to be essentially binary yet be on a “hair-trigger” in response to environmental perturbations.

## Supplementary Material

Supporting Information
