# Supplementary material for: Identification and Characterization of Wor4, a New Transcriptional Regulator of White-Opaque Switching
Source: G3 (Bethesda). 2016 Jan 13;6(3):721–9. doi: 10.1534/g3.115.024885 (PMC4777133; doi:10.1534/g3.115.024885)
Supplement: Supporting Information [file supp_6_3_721__index.html]

Identification and Characterization of Wor4, a New Transcriptional Regulator of White-Opaque Switching — Supporting Information 

# Identification and Characterization of Wor4, a New Transcriptional Regulator of White-Opaque Switching

## Supporting Information for Lohse and Johnson, 2016

**Files in this Data Supplement:**

- Supporting Information - File contains supporting Figures and Tables, and References. It also contains the legends for Files S1-S3. (.pdf, 681 KB)
- Figure S3 - Wor4 belongs to the C2H2 Zinc Finger family of proteins and is found across the fungal domain. (.pdf, 65 KB)
- Table S1 - Oligonucleotides used in this study. (.pdf, 132 KB)
- Table S2 - Plasmids used in this study. (.pdf, 199 KB)
- Table S3 - Strains used in this study. (.pdf, 234 KB)
- Table S4 - Ectopic expression of *WOR4* does not induce white-to-opaque switching in a *wor1* deletion strain. (.pdf, 198 KB)
- Table S5 - Breakdown of overlap between Wor4 binding and binding of other core regulators in white cells. (.pdf, 109 KB)
- Table S6 - Breakdown of overlap between Wor4 binding and binding of other core regulators in opaque cells. (.pdf, 116 KB)
- File S2 - MochiView image plots of 12kb regions centered on the Wor4 binding sites in white and opaque cells. (.pdf, 2,490 KB)
- Figure S1 - Single cell morphology of *wor4* and *rfg1* deletions. (.pdf, 136 KB)
- Figure S2 - Wor4 localizes to the nucleus in both white and opaque cells. (.pdf, 136 KB)
- File S1 - Genomic location and median fold enrichment of Wor4-GFP peaks in white and opaque cells. (.xlsx, 18 KB)
- File S3 - Compilation of microarray, RNA-seq, ChIP-seq, and ChIP-chip data presented in this study and from previous studies. (.xlsx, 850 KB)
