# Supplementary material for: Identification and Characterization of Wor4, a New Transcriptional Regulator of White-Opaque Switching
Source: G3 (Bethesda). 2016 Jan 13;6(3):721–9. doi: 10.1534/g3.115.024885 (PMC4777133; doi:10.1534/g3.115.024885)
Supplement: Supporting Information [file supp_g3.115.024885_024885SI.pdf]

Identification and characterization of Wor4, a new transcriptional regulator of white-opaque switching

Matthew B. Lohse[*,1] and Alexander D. Johnson[*,†,2]

[*]Department of Microbiology and Immunology, 600 16th Street, University of California, San Francisco, San Francisco, CA 94158, USA

[†]Department of Biochemistry and Biophysics, 600 16th Street, University of California, San Francisco, San Francisco, CA 94158, USA

[1]Present address: BioSynesis Inc., San Francisco, CA, USA

[2]Address correspondence to: Alexander D. Johnson (ajohnson@cgl.ucsf.edu)

Supplemental Figures

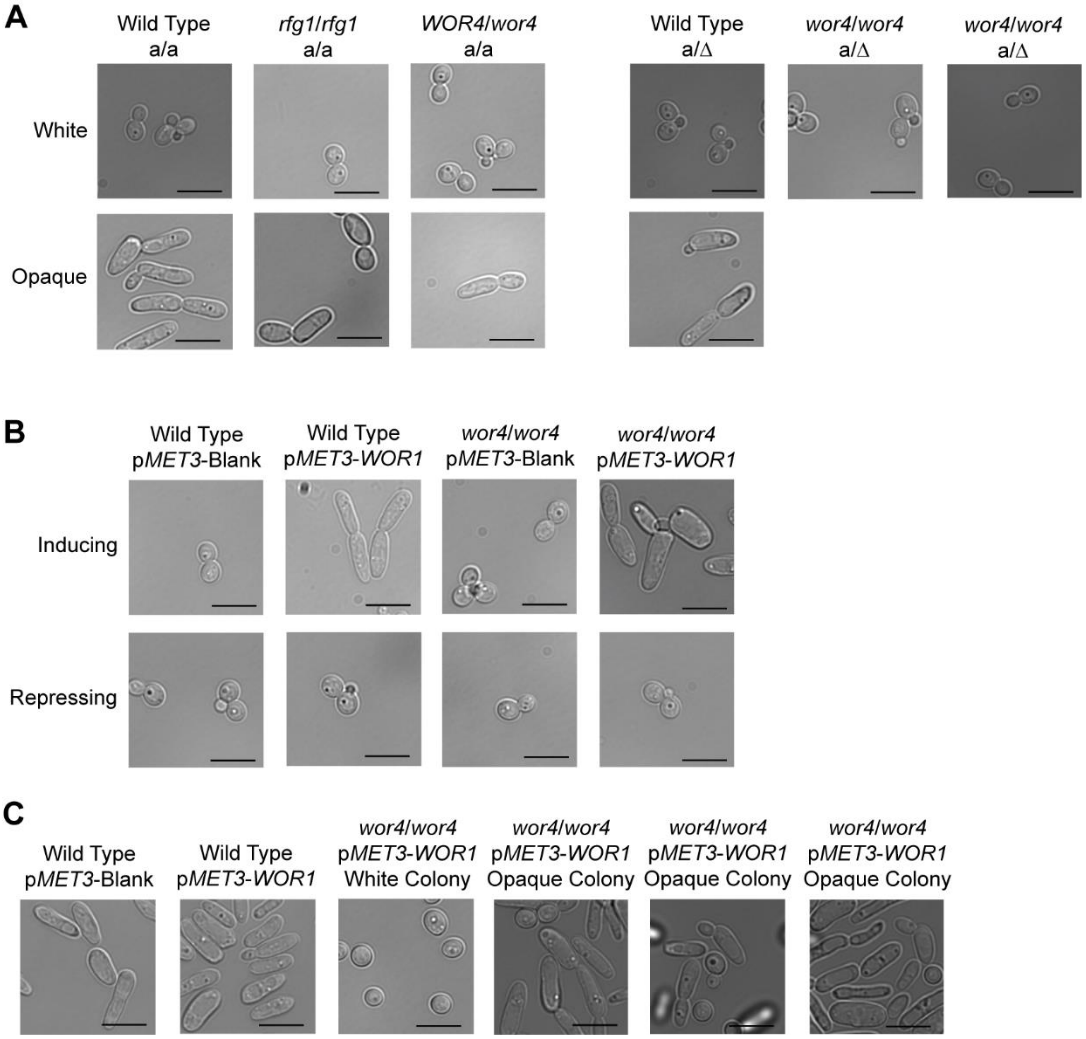

Figure S1: Single cell morphology of *wor4* and *rfg1* deletions. (a) Typical white and opaque cells for the *wor4* heterozygous deletion as well as the *wor4* and *rfg1* homozygous deletions as well as the matching wild type strains. (b) Typical cellular morphology for strains with the p*MET3* ectopic expression system driving *WOR1* or an empty control in the wild type and *wor4* deletion backgrounds on inducing or repressing media. (c) Opaque colonies from the WOR1 ectopic expression assay in (b) were restreaked to repressing media plates and the resulting colonies were then resuspended in water, diluted, and plated on repressing media and allowed to grow.

Images were taken of typical cells from one white and three opaque looking p*MET3-WOR1 wor4/wor4* colonies as well as opaque colonies of the control strains. Scale bars are 10µm.

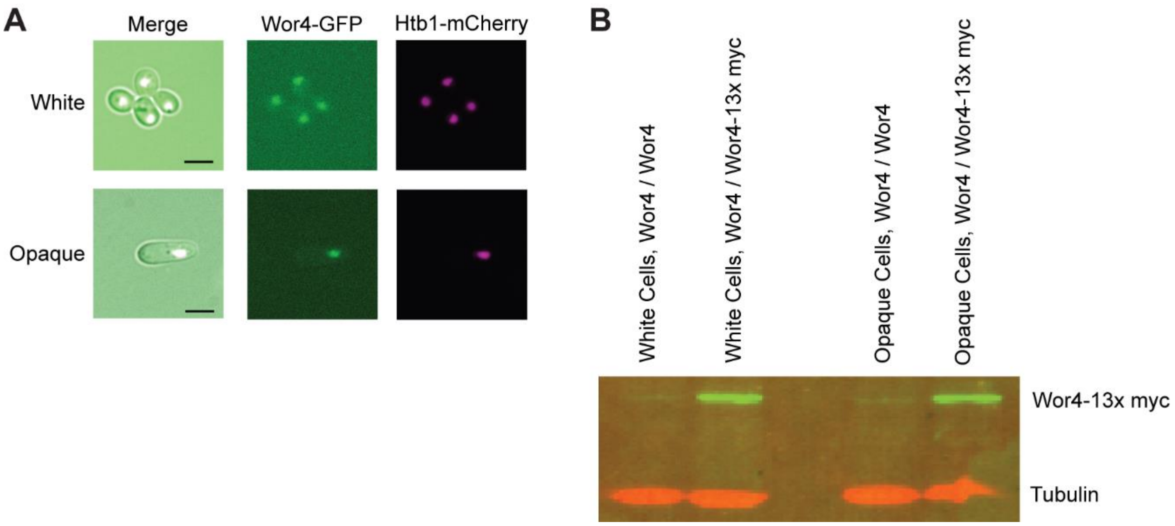

Figure S2: Wor4 localizes to the nucleus in both white and opaque cells. (a) Visualization of Wor4-GFP and Htb1-mCherry fusion proteins in both white and opaque cells. Merged images (DIC, GFP, mCherry), GFP fluorescence, and mCherry fluorescence are shown. Scale bar is 5µm. (b) Western blot of white and opaque strains either containing (Wor4/Wor4-13x myc) or lacking (Wor4/Wor4) c-terminally 13x myc tagged Wor4. Wor4 in green, α-tubulin loading control in red.

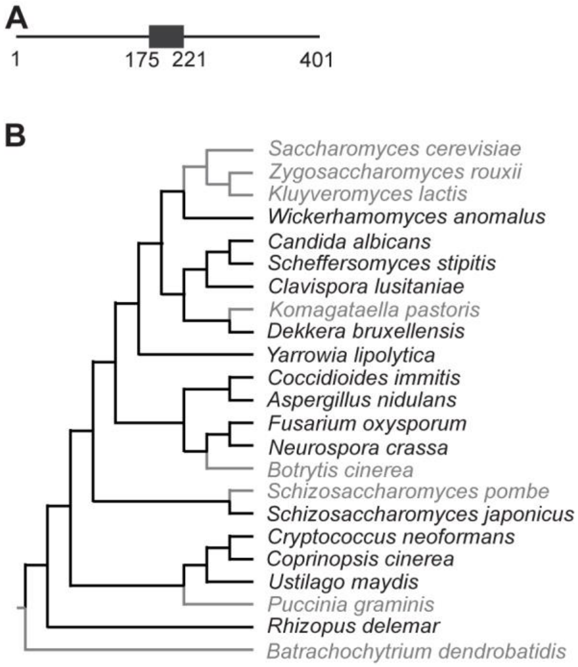

Figure S3: Wor4 belongs to the C2H2 Zinc Finger family of proteins and is found across the fungal domain. (a) Scale model of Wor4, the black box represents the 47aa (amino acids 175-221) region identified by HHpred as belonging to the C2H2 Zinc Finger family. ELM identified a similar, although smaller, region (amino acids 186-212) as belonging to the same family. (b) Phylogenetic tree of 23 fungal species, species with a Wor4 homolog are in black and species lacking a Wor4 homolog are in grey. Branch lengths are not to scale.

Supplemental Tables

Table S1: Oligonucleotides used in this study.

Category	Name	Description	Oligonucleotide sequence
pMBL180 construction	MBL122	mCherry 5' HindIII	gttacaaagcttATG GTT TCT AAG GGT GAA GAA GAT
pMBL180 construction	MBL121	mCherry 3' PstI	ctcggactgcagTTAC TTG TAC AAT TCA TCC ATA CCA CC
pMBL180 construction	MBL354	SAT1 5' PstI	Gttaca ctgcag GAG TGA AAT TCT GGA AAT CTG GAA ATC
pMBL180 construction	MBL355	SAT1 3' HindIII	ctcgga ggatcc GCA GGA CCA CCT TTG ATT GTA AAT AG
pADH76 Construction	AHO696	GFP 5' AvaI	aaaactcgagcggatccccgggttaattaacggtatgtctaaaggtgaagaattattcactggtg
pADH76 Construction	AHO698	GFP 3' AvaI	cttcctcgagTTATTTGTACAATTCATCCATACCATG
pADH57 construction	AHO407	TDH3 5' XbaI	tgggtatctagatgctcctcgtcgacaacgac
pADH57 construction	AHO408	TDH3 3' BamHI	CCCGGGGGATCCTAATTTGATTGTAAAGTTTGTTGATGTTAATTG
WOR4 Ectopic Expression	MLP780	Wor4 5' BamHI	Gttaca GGATCC ATG TCG AGT GAT AAA CCT GAA CAA G
WOR4 Ectopic Expression	MLP781	Wor4 3' XmaI	CTC GGA CCC GGG TTA AAT GCC TGG TTG GGT TG
WOR4 Ectopic Expression	MLP1137	Wor4 3' PstI	CTC GGA CTG CAG TTA AAT GCC TGG TTG GGT TG

RFG1 Ectopic Expression	MLP777	Rfg1 5' BamHI	Gttaca GGATCC ATG TCT ACT GCT ATC TAC TAT TCA ACT C
RFG1 Ectopic Expression	MLP778	Rfg1 3' XmaI	CTC GGA CCC GGG TTA TTG TGG AGG TTG TTG TTG ATG AT
WOR4 Deletion	NHO320	WOR4 5' External	TTGGGCTGATCTATACTTCTGGGTA
WOR4 Deletion	NHO321	WOR4 5' Internal	CACGGCGCGCCTAGCAGCGGCCTCAAGCTGCAAAAATAGTTTCTCTC
WOR4 Deletion	NHO322	WOR4 3' Internal	GTCAGCGGCCGCATCCCTGCGAAATGAGGATTTGAATGATCCTCTGTTG
WOR4 Deletion	NHO323	WOR4 3' External	TCAGCACTATCCATATCTTCAGCCA
WOR4 Deletion	NHO324	WOR4 5' Check	GAAGGAATTGAGCAATAGGACAAAGATTTG
WOR4 Deletion	NHO325	WOR4 3' Check	AGCGATGAATCAACATCACCAACTTG
WOR4 Deletion	NHO326	WOR4 5' ORF Check Set 1	ATGCCAATTACCAACCTCAAGCTC
WOR4 Deletion	NHO327	WOR4 3' ORF Check Set 1	CCATACCAATAGTTTCCAGCAGT
WOR4 Deletion	MLP1051	WOR4 5' ORF Check Set 2	TAA CCA AAA AGC ATG GTC ACA GAA AAT C
WOR4 Deletion	MLP1052	WOR4 3' ORF Check Set 2	CAA CGT CAT TGA TTC CTA AGA GAG AC
WOR4 Deletion	MLp1053	WOR4 5' ORF Check Set 3	CCT AGA TCC AAA GGA TTC AAA AAA CGC
WOR4 Deletion	MLp1054	WOR4 3' ORF	CAG GAA ACG AAC AAG GAT ATT TTC TCT C

		Check Set 3	
RFG1 Deletion	NHO3 47	RFG1 5' External	GGTGACTCCGATTTTCCGAACTAA
RFG1 Deletion	NHO3 48	RFG1 5' Internal	CACGGCGCGCCTAGCAGCGGTGGTGTGATGGTTTGCAAATAAATAC
RFG1 Deletion	NHO3 49	RFG1 3' Internal	GTCAGCGGCCGCATCCCTGCGTAGCTTGCTTTCTCTACAGAAAAGAGAAT
RFG1 Deletion	NHO3 50	RFG1 3' External	AAGGTGGCCGTGTGAGAAAGTTTA
RFG1 Deletion	NHO3 51	RFG1 5' Check	CACACACCTGCACACCTACATTTG
RFG1 Deletion	NHO3 52	RFG1 3' Check	TTGGTTTAGGACCTGGGCGT
RFG1 Deletion	NHO3 53	RFG1 5' ORF Check Set	TTGGTGGTGGTATTGATGGTAACTTT
RFG1 Deletion	NH354	RFG1 3' ORF Check Set	ACGAGAAGTTAATCCACCACTGAGACT
WOR4 Tagging	MLP87 4	WOR4-13x Myc and -GFP 5'	Cttaggaatcaatgacgttgattcaagaaaccaaattgacgataacaaaccaacccaaccaggcatt cggatccccgggttaattaacgg
WOR4 Tagging	MLP87 5	WOR4-13x Myc and -GFP 3'	TTA ATT TTC TAA AAT AAA TCT ATA TGA ATA CAA CAG AGG ATC ATT CAA ATC CTC ATT TCA TTT A GGCGGCCGCTCTAGAACTAGTGGATC
WOR4 Tagging	mLp89 3	WOR4 5' check	Atgattacactgctggaaactattggtatg
WOR4 Tagging	MLP88 0	WOR4 3' check	GTG GTA CGT GAC AAT GAG GTT GGG
WOR4 Tagging	MLP87 6	myc 5' check	CCGTTAATTAACCCGGGGATC
WOR4 Tagging	MLP87 7	myc 3' in check	Ggaacttcagatccactagttctagagc

WOR4 Tagging	MLP878	myc 3' flipped check	TCACTAGTGAATTCGCGCTCGAG
WOR4 Tagging	MBL209	GFP 5' check	GGT TGG CCA TGG AAC TGG CA
WOR4 Tagging	MBL382	GFP 3' flipped check	GGT GAT GGT CCA GTC TTG TTA CCA GAC
HTB1 and HTB2 Tagging	MLP1194	HTB1-mCherry 5'	gccgtttccgaaggtaccagagccgtcacaaaatactcatctgcttctagt ggtAGAAGAatcCCAGGTttaattaac GTT TCT AAG GGT GAA GAA GAT A
HTB1 and HTB2 Tagging	MLP1195	HTB1-mCherry 3'	AAA AAA AAA GTG GGC AAC TAA AAA TAC AAT TGG GAG ACA ATA CAA GAT CCA TCA CAT CTA GCA GGA CCA CCT TTG ATT GTA AAT AG
HTB1 and HTB2 Tagging	MLP1192	HTB2-mCherry 5'	gctgtttctgaaggtactagagctgttaccaaatactcttctgcttctaat ggtAGAAGAatcCCAGGTttaattaac GTT TCT AAG GGT GAA GAA GAT A
HTB1 and HTB2 Tagging	MLP1193	HTB2-mCherry 3'	AAC AAT AAT TTG GAG AAA TAA ACC ATT CAT GAC AAA CCT CTC TCT CTC TTT CTT TTT TTA GCA GGA CCA CCT TTG ATT GTA AAT AG
HTB1 and HTB2 Tagging	mLP1198	HTB1 5' check	TCT CCT TCT CTC TCT TGT CAC TTC TTC TTC CTC
HTB1 and HTB2 Tagging	MLP1199	HTB1 3' check	CCA AGA GGT CCC TGA AAT TCA GAA GTT TCT TG
HTB1 and HTB2 Tagging	MLp1196	HTB2 5' check	CAA TAA CAA CAA GAG ATT CAC GTG ACA CAC AAA
HTB1 and	MLP1197	HTB2 3' check	GGA GAT AGA AAA GAA TTG GGT CCA ACA CCA

HTB2 Tagging			
HTB1 and HTB2 Tagging	MLP12 03	mCherr y 5' check	TTG TGG AGA CAA AAT ATC CCA GGC GAA TG
HTB1 and HTB2 Tagging	MLP12 04	mCherr y 3' Check	TTG ACC TCT TCA CGT ATA AAA CTA GAC CTC
qPCR	MLP11 86	Wor1 5'	TGGGTATGGTAACCACCTT
qPCR	MLP11 87	Wor1 3'	TGATACTACCTGTACCAGTCGCAA
qPCR	MLP11 90	Dyn1 control 5'	CATCCAACACTTCCAACCAATTACATTA
qPCR	MLP11 91	Dyn1 control 3'	TAATTCTTGTTCAAAAGCCAAAGATTCG

Table S1: Oligonucleotides used in this study.

Table S2: Plasmids used in this study.

Description	Name	Reference
LEU2 Knock Out	pSN40	1
HIS1 Knock Out	pSN52	1
Arg MTL Knock Out Cassette	pJD1	2
p*MET3-blank-SAT*1	pADH33	3
p*TDH3-blank-SAT*1	pADH57	This Study
p*MET3-WOR1-SAT1*	pADH35	4
p*MET3-WOR4-SAT1*	pMBL640	This Study
p*MET3-RFG1-SAT1*	pMBL639	This Study
p*TDH3-WOR4-SAT1*	pMBL707	This Study
C-terminal CaGFP Source	pADH76	This Study
C-terminal 13x Myc Source	pADH34	5
C-terminal mCherry Source	pMBL180	This Study

References

1 Noble, S. M., and A. D. Johnson, 2005 Strains and strategies for large-scale gene deletion studies of the diploid human fungal pathogen *Candida albicans*. Eukaryot. Cell 4: 298–309.

2 Lin, C. H., S. Kabrawala, E. P. Fox, C. J. Nobile, A. D. Johnson et al., 2013 Genetic control of conventional and pheromone-stimulated biofilm formation in *Candida albicans*. PLoS Pathog. 9: e1003305.

3 Lohse, M. B., A. D. Hernday, P. M. Fordyce, L. Noiman, T. R. Sorrells et al., 2013 Identification and characterization of a previously undescribed family of sequence-specific DNA-binding domains. Proc. Natl. Acad. Sci. U. S. A. 110: 7660–7665.

4 Hernday, A. D., M. B. Lohse, P. M. Fordyce, C. J. Nobile, J. D. DeRisi et al., 2013 Structure of the Transcriptional Network Controlling White-Opaque Switching in *Candida albicans*. Mol. Microbiol. 90: 22–35.

5 Hernday, A. D., S. M. Noble, Q. M. Mitrovich, and A. D. Johnson, 2010 Genetics and molecular biology in *Candida albicans*. Methods Enzymol. 470: 737–758.

Table S2: Plasmids used in this study.

Table S3: Strains used in this study.

Description	Number	Genotype	Reference
Starting Strains			
a/α -His -Leu -Arg Strain	SNY152	*a/alpha leu2Δ/leu2Δ his1Δ/his1Δ URA3/ura3Δ::imm^{434} IRO1/iro1Δ::imm^{434} arg1::hisG/arg1::hisG*	1
a/a -His -Leu Strain	RZY47	*a/a leu2Δ/leu2Δ his1Δ/his1Δ URA3/ura3Δ::imm434 IRO1/iro1Δ::imm434*	2
Controls			
Wild Type, White	WTwhite, AHY304	*a/Δalpha C.m.LEU2/leu2Δ C.d.HIS1/his1Δ URA3/ura3Δ::imm^{434} IRO1/iro1Δ::imm^{434} arg1::hisG/arg1::hisG ΔMTLalpha ::ARG1*	3
Wild Type, Opaque	WTopaque, AHY336	*a/Δalpha C.m.LEU2/leu2Δ C.d.HIS1/his1Δ URA3/ura3Δ::imm^{434} IRO1/iro1Δ::imm^{434} arg1::hisG/arg1::hisG ΔMTLalpha ::ARG1*	3
Wild Type, White	AHY135	*a/a C.m.LEU2/leu2Δ C.d.HIS1/his1Δ URA3/ura3Δ::imm^{434} IRO1/iro1Δ::imm^{434}*	3
Wild Type, Opaque	AHY136	*a/a C.m.LEU2/leu2Δ C.d.HIS1/his1Δ URA3/ura3Δ::imm^{434} IRO1/iro1Δ::imm^{434}*	3
Ectopic Expression Strains			
Wild Type, White, p*MET3*-Blank	AHY214	*a/a C.m.LEU2/leu2Δ* C.d.HIS1/his1Δ URA3/ura3Δ::imm^{434} IRO1/iro1Δ::imm^{434} p*MET3*-blank; SAT1; RP10	3
Wild Type, Opaque, p*MET3*-Blank	AHY375, MLY1166	*a/a C.m.LEU2/leu2Δ* C.d.HIS1/his1Δ URA3/ura3Δ::imm^{434} IRO1/iro1Δ::imm^{434} p*MET3*-blank; SAT1; RP10	This study
Wild Type, White, p*MET3-WOR1*	AHY204	*a/a C.m.LEU2/leu2Δ* C.d.HIS1/his1Δ URA3/ura3Δ::imm^{434} IRO1/iro1Δ::imm^{434} p*MET3-WOR1*; SAT1; RP10	3
Wild Type, Opaque, p*MET3-WOR1*	MLY1165	*a/a C.m.LEU2/leu2Δ* C.d.HIS1/his1Δ URA3/ura3Δ::imm^{434} IRO1/iro1Δ::imm^{434} p*MET3-WOR1*; SAT1; RP10	This Study
Wild Type, White, p*MET3-WOR4*	MLY1214	*a/a C.m.LEU2/leu2Δ* C.d.HIS1/his1Δ URA3/ura3Δ::imm^{434} IRO1/iro1Δ::imm^{434} p*MET3-WOR4*; SAT1; RP10	This Study
Wild Type, Opaque, p*MET3-WOR4*	MLY1230	*a/a C.m.LEU2/leu2Δ* C.d.HIS1/his1Δ URA3/ura3Δ::imm^{434} IRO1/iro1Δ::imm^{434} p*MET3-WOR4*; SAT1; RP10	This Study

Wild Type, White, p*MET3-RFG1*	MLY1221	*a/a C.m.LEU2/leu2*Δ C.d.HIS1/his1Δ URA3/ura3Δ::imm^{434} IRO1/iro1Δ::imm^{434} p*MET3-RFG1*; SAT1; RP10	This Study
Wild Type, Opaque, p*MET3-RFG1*	MLY1241	*a/a C.m.LEU2/leu2*Δ C.d.HIS1/his1Δ URA3/ura3Δ::imm^{434} IRO1/iro1Δ::imm^{434} p*MET3-RFG1*; SAT1; RP10	This Study
wor4/wor4, White, p*MET3*-Blank	MLY1372, MLY1379	*a/Δalpha leu2Δ/leu2Δ his1Δ/his1Δ URA3/ura3Δ::imm^{434} IRO1/iro1Δ::imm^{434} arg1::hisG/arg1::hisG wor4Δ::C.m.LEU2/wor4Δ::C.d.HIS1 ΔMTLalpha ::ARG1* p*MET3*-blank; SAT1; RP10	This Study
wor4/wor4, White, p*MET3-WOR1*	MLY1373, MLY1380	*a/Δalpha leu2Δ/leu2Δ his1Δ/his1Δ URA3/ura3Δ::imm^{434} IRO1/iro1Δ::imm^{434} arg1::hisG/arg1::hisG wor4Δ::C.m.LEU2/wor4Δ::C.d.HIS1 ΔMTLalpha ::ARG1* p*MET3-WOR1*; SAT1; RP10	This Study
Wild Type, White, p*MET3*-Blank	MLY1365	*a/Δalpha C.m.LEU2/leu2Δ C.d.HIS1/his1Δ URA3/ura3Δ::imm^{434} IRO1/iro1Δ::imm^{434} arg1::hisG/arg1::hisG ΔMTLalpha ::ARG1 pMET3*-blank; SAT1; RP10	This Study
Wild Type, White, p*MET3-WOR1*	MLY1366	*a/Δalpha C.m.LEU2/leu2Δ C.d.HIS1/his1Δ URA3/ura3Δ::imm^{434} IRO1/iro1Δ::imm^{434} arg1::hisG/arg1::hisG ΔMTLalpha ::ARG1 pMET3-WOR1*; SAT1; RP10	This Study
wor1/wor1, White, p*MET3*-Blank	MLY1393	*a/a leu2Δ/leu2Δ his1Δ/his1Δ URA3/ura3Δ::imm^{434} IRO1/iro1Δ::imm^{434} wor1Δ::C.m.LEU2/wor1Δ::C.d.HIS1* p*MET3*-blank; SAT1; RP10	This Study
wor1/wor1, White, p*MET3-WOR1*	MLY1394	*a/a leu2Δ/leu2Δ his1Δ/his1Δ URA3/ura3Δ::imm^{434} IRO1/iro1Δ::imm^{434} wor1Δ::C.m.LEU2/wor1Δ::C.d.HIS1* p*MET3-WOR1*; SAT1; RP10	This Study
wor1/wor1, White, p*MET3-WOR4*	MLY1395	*a/a leu2Δ/leu2Δ his1Δ/his1Δ URA3/ura3Δ::imm^{434} IRO1/iro1Δ::imm^{434} wor1Δ::C.m.LEU2/wor1Δ::C.d.HIS1* p*MET3-WOR4*; SAT1; RP10	This Study
Wild Type, p*TDH3-WOR4*	MLY1420	*a/a C.m.LEU2/leu2*Δ C.d.HIS1/his1Δ URA3/ura3Δ::imm^{434} IRO1/iro1Δ::imm^{434} p*TDH3-WOR4*; SAT1; RP10	This Study
Deletion Strains			

wor1/wor1, White	RZY219	*a/a leu2Δ/leu2Δ his1Δ/his1Δ URA3/ura3Δ::imm^{434} IRO1/iro1Δ::imm^{434} wor1Δ::C.m.LEU2/wor1Δ::C.d.HIS1*	2
WOR4/wor4, White	MLY1135	*a/a leu2Δ/leu2Δ his1Δ/his1Δ URA3/ura3Δ::imm^{434} IRO1/iro1Δ::imm^{434} WOR4/wor4Δ::C.d.HIS1*	This Study
WOR4/wor4, Opaque	MLY1137	*a/a leu2Δ/leu2Δ his1Δ/his1Δ URA3/ura3Δ::imm^{434} IRO1/iro1Δ::imm^{434} WOR4/wor4Δ::C.d.HIS1*	This Study
wor4/wor4, White	MLY1355A, MLY1355B	*a/Δalpha leu2Δ/leu2Δ his1Δ/his1Δ URA3/ura3Δ::imm^{434} IRO1/iro1Δ::imm^{434} arg1::hisG/arg1::hisG wor4Δ::C.m.LEU2/wor4Δ::C.d.HIS1 ΔMTLalpha ::ARG1*	This Study
rfg1/rfg1, White	MLY1136	*a/a leu2Δ/leu2Δ his1Δ/his1Δ URA3/ura3Δ::imm^{434} IRO1/iro1Δ::imm^{434} rfg1Δ::C.m.LEU2/rfg1Δ::C.d.HIS1*	This Study
rfg1/rfg1, Opaque	MLY1138	*a/a leu2Δ/leu2Δ his1Δ/his1Δ URA3/ura3Δ::imm^{434} IRO1/iro1Δ::imm^{434} rfg1Δ::C.m.LEU2/rfg1Δ::C.d.HIS1*	This Study
Tagged Strains			
Wor4-GFP, White	MLY1295	*a/a C.m.LEU2/leu2Δ C.d.HIS1/his1Δ URA3/ura3Δ::imm^{434} IRO1/iro1Δ::imm^{434} WOR4/WOR4-GFP*	This Study
Wor4-GFP, Opaque	MLY1304	*a/a C.m.LEU2/leu2Δ C.d.HIS1/his1Δ URA3/ura3Δ::imm^{434} IRO1/iro1Δ::imm^{434} WOR4/WOR4-GFP*	This Study
Wor4-13x myc, White	MLY1286	*a/a C.m.LEU2/leu2Δ C.d.HIS1/his1Δ URA3/ura3Δ::imm^{434} IRO1/iro1Δ::imm^{434} WOR4/WOR4-13x myc*	This Study
Wor4-13x myc, Opaque	MLY1298	*a/a C.m.LEU2/leu2Δ C.d.HIS1/his1Δ URA3/ura3Δ::imm^{434} IRO1/iro1Δ::imm^{434} WOR4/WOR4-13x myc*	This Study
Microscopy Strains			
HTB1-mCherry, Wor4-GFP, White	MLY1460	*a/a C.m.LEU2/leu2Δ C.d.HIS1/his1Δ URA3/ura3Δ::imm^{434} IRO1/iro1Δ::imm^{434} WOR4/WOR4-GFP HTB1/HTB1-mCherry::SAT1*	This Study
HTB1-mCherry, Wor4-GFP, Opaque	MLY1467	*a/a C.m.LEU2/leu2Δ C.d.HIS1/his1Δ URA3/ura3Δ::imm^{434} IRO1/iro1Δ::imm^{434} WOR4/WOR4-GFP HTB1/HTB1-mCherry::SAT1*	This Study

HTB2-mCherry, Wor4-GFP, White	MLY1462	*a/a C.m.LEU2/leu2Δ C.d.HIS1/his1Δ URA3/ura3Δ::imm*^{434} *IRO1/iro1Δ::imm*^{434} *WOR4/WOR4-GFP HTB2/HTB2-mCherry::SAT1*	This Study
HTB2-mCherry, Wor4-GFP, Opaque	MLY1469	*a/a C.m.LEU2/leu2Δ C.d.HIS1/his1Δ URA3/ura3Δ::imm*^{434} *IRO1/iro1Δ::imm*^{434} *WOR4/WOR4-GFP HTB2/HTB2-mCherry::SAT1*	This Study

References

1 Noble, S. M., and A. D. Johnson, 2005 Strains and strategies for large-scale gene deletion studies of the diploid human fungal pathogen *Candida albic*ans. Eukaryot. Cell 4: 298–309.

2 Zordan, R. E., D. J. Galgoczy, and A. D. Johnson, 2006 Epigenetic properties of white-opaque switching in *Candida albicans* are based on a self-sustaining transcriptional feedback loop. Proc. Natl. Acad. Sci. U. S. A. 103: 12807–12812.

3 Hernday, A. D., M. B. Lohse, P. M. Fordyce, C. J. Nobile, J. D. DeRisi et al., 2013 Structure of the Transcriptional Network Controlling White-Opaque Switching in *Candida albicans*. Mol. Microbiol. 90: 22–35.

Table S3: Strains used in this study.

Table S4: Ectopic expression of *WOR4* does not induce white-to-opaque switching in a *wor1* deletion strain.
White-to-Opaque Switching

Strain	Media Condition	Switching Frequency (%)	n	Notes
Wild Type, p*MET*3-Blank	Repressing	<0.48	208	1
Wild Type, p*MET*3-Blank	Inducing	<0.26	378	1
Wild Type, p*MET*3-*WOR1*	Repressing	<0.48	208	2
Wild Type, p*MET*3-*WOR1*	Inducing	96.98	430	2
Wild Type, p*MET*3-*WOR4*	Repressing	<0.48	210	
Wild Type, p*MET*3-*WOR4*	Inducing	20.71	449	
wor1/wor1, p*MET3*-Blank	Repressing	<0.46	219	
wor1/wor1, p*MET3*-Blank	Inducing	<0.23	432	
wor1/wor1, p*MET3-WOR1*	Repressing	<0.49	205	
wor1/wor1, p*MET3-WOR1*	Inducing	100.00	383	
wor1/wor1, p*MET3-WOR4*	Repressing	<0.37	272	
wor1/wor1, p*MET3-WOR4*	Inducing	<0.18	546	

Notes

1 Negative Control
2 Positive Control

Table S4: Ectopic expression of *WOR4* does not induce white-to-opaque switching in a *wor1* deletion strain. Switching frequency and number of colonies scored for ectopic expression of *WOR1* and *WOR4* in the *wor1* deletion background are indicated.

Table S5: Breakdown of overlap between Wor4 binding and binding of other core regulators in white cells.

Class	Observed Binding	Number of Regions	Percent of Wor4 sites
Single TR	Wor4 alone	2	22.22
Two TR	Ahr1	1	11.11
Two TR	Czf1	1	11.11
Two TR	Efg1	3	33.33
Three TR	Ahr1+Czf1	1	11.11
Three TR	Ahr1+Efg1	1	11.11
Total		9	100.00
With Ahr1		3	33.33
With Czf1		2	22.22
With Efg1		4	44.44
Two or More		7	77.78
Three or More		2	22.22

Table S5: Breakdown of overlap between Wor4 binding and binding of other core regulators in white cells. Instances of specific binding combinations, overall overlap with specific regulators, and the number of sites with at least a given number of regulators bound are indicated. Only binding events with Wor4 present are considered. Binding of Ahr1, Czf1, and Efg1 have been previously reported (Hernday *et al.* 2013).

Table S6: Breakdown of overlap between Wor4 binding and binding of other core regulators in opaque cells.

Class	Observed Binding	Number of Regions	Percent of Wor4 sites
Single TR	Wor4 alone	4	2.92
Two TR	Wor1	8	5.84
Two TR	Wor2	3	2.19
Two TR	Wor3	1	0.73
Three TR	Ahr1+Wor2	1	0.73
Three TR	Efg1+Wor1	1	0.73
Three TR	Efg1+Wor2	1	0.73
Three TR	Wor1+Wor2	6	4.38
Four TR	Ahr1+Wor1+Wor2	2	1.46
Four TR	Czf1+Wor2+Wor3	1	0.73
Four TR	Efg1+Wor1+Wor2	27	19.71
Five TR	Ahr1+Efg1+Wor1+Wor2	12	8.76
Five TR	Ahr1+Wor1+Wor2+Wor3	2	1.46
Five TR	Czf1+Efg1+Wor1+Wor2	7	5.11
Five TR	Efg1+Wor1+Wor2+Wor3	17	12.41
Six TR	Ahr1+Czf1+Efg1+Wor1+Wor2	3	2.19
Six TR	Ahr1+Efg1+Wor1+Wor2+Wor3	14	10.22
Six TR	Czf1+Efg1+Wor1+Wor2+Wor3	5	3.65
Seven TR	Ahr1+Czf1+Efg1+Wor1+Wor2+Wor3	22	16.06
Total		137	100.00
With Ahr1		56	40.88
With Czf1		38	27.74
With Efg1		109	79.56
With Wor1		126	91.97
With Wor2		123	89.78
With Wor3		62	45.26
Two or More		133	97.08
Three or More		121	88.32
Four or More		112	81.75
Five or More		82	59.85
Six or More		44	32.12
Seven or More		22	16.06

Table S6: Breakdown of overlap between Wor4 binding and binding of other core regulators in opaque cells. Instances of specific binding combinations, overall overlap with specific regulators, and the number of sites with at least a given number of regulators bound are indicated. Only binding events with Wor4 present are considered. Binding of Ahr1, Czf1, Efg1, Wor1, Wor2, and Wor3 have been previously reported (Zordan *et al.* 2007; Hernday *et al.* 2013; Lohse *et al.* 2013).

Supplemental File Captions

File S1: Genomic location and median fold enrichment of Wor4-GFP peaks in white and opaque cells.

File S2: MochiView image plots of 12kb regions centered on the Wor4 binding sites in white and opaque cells. Plots produced using the SnapShot Function in MochiView v1.46 (Homann and Johnson 2010).

File S3: Compilation of microarray, RNA-seq, ChIP-seq, and ChIP-chip data presented in this study and from previous studies. From left to right in the Excel spreadsheet, columns are as follows. (A) Orf19 number designation based on the Candida Genome Database (CGD). (B) Gene name, where applicable. (C) Whether the gene is a transcriptional regulator, based on Homann *et al.* (Homann *et al.* 2009), a "1" represents yes. (D) Maximum Czf1 enrichment in the upstream region for the gene in a white cell, values are on a log2 scale (Hernday *et al.* 2013). (E) Maximum Efg1 enrichment in the upstream region for the gene in a white cell; values are on a log2 scale (Hernday *et al.* 2013). (F) Maximum Ahr1 enrichment in the upstream region for the gene in a white cell; values are on a log2 scale (Hernday *et al.* 2013). (G) Maximum Wor4 enrichment in the upstream region for the gene in a white cell; values are MACS2 fold enrichment. (H) Maximum Wor1 enrichment in the upstream region for the gene in an opaque cell (Zordan *et al.* 2007); values are on a log2 scale. (I) Maximum Wor2 enrichment in the upstream region for the gene in an opaque cell; values are on a log2 scale (Hernday *et al.* 2013). (J) Maximum Czf1 enrichment in the upstream region for the gene in an opaque cell; values are on a log2 scale (Hernday *et al.* 2013). (K) Maximum Efg1 enrichment in the upstream region for

the gene in an opaque cell; values are on a log2 scale (Hernday *et al.* 2013). (L) Maximum Wor3 enrichment in the upstream region for the gene in an opaque cell; values are on a log2 scale (Lohse *et al.* 2013). (M) Maximum Ahr1 enrichment in the upstream region for the gene in an opaque cell; values are on a log2 scale (Hernday *et al.* 2013). (N) Maximum Wor4 enrichment in the upstream region for the gene in an opaque cell; values are MACS2 fold enrichment. (O) Previously published RNA-seq of opaque versus white cells (Tuch *et al.* 2010); values are on a log2 scale. (P) Previously published microarray analysis of opaque versus white cells (Lohse *et al.* 2013); values are on a log2 scale. (Q) Microarray analysis of a white *czf1* deletion strain versus wild-type white cells; values are on a log2 scale (Hernday *et al.* 2013). (R) Microarray analysis of a white *efg1* deletion strain versus wild-type white cells; values are on a log2 scale (Hernday *et al.* 2013). (S) Microarray analysis of a white *wor3* deletion strain versus wild-type white cells; values are on a log2 scale (Lohse *et al.* 2013). (T) Microarray analysis of a white *ahr1* deletion strain versus wild-type white cells; values are on a log2 scale (Hernday *et al.* 2013). (U) Microarray analysis of an opaque *wor2* deletion strain with ectopically expressed Wor1 versus wildtype opaque cells with ectopically expressed Wor1; values are on a log2 scale (Hernday *et al.* 2013). (V) Microarray analysis of an opaque *czf1* deletion strain versus wildtype opaque cells; values are on a log2 scale (Hernday *et al.* 2013). (W) Microarray analysis of an opaque *efg1* deletion strain versus wildtype opaque cells; values are on a log2 scale (Hernday *et al.* 2013). (X) Microarray analysis of an opaque *wor3* deletion strain versus wildtype opaque cells; values are on a log2 scale (Hernday *et al.* 2013; Lohse *et al.* 2013). (Y) Microarray analysis of an opaque *ahr1* deletion strain versus wildtype opaque cells; values are on a log2 scale (Hernday *et al.* 2013).

Supplemental Literature Cited

Hernday, A. D., M. B. Lohse, P. M. Fordyce, C. J. Nobile, J. D. DeRisi *et al.*, 2013 Structure of the transcriptional network controlling white-opaque switching in *Candida albicans*. Mol. Microbiol. 90: 22–35.

Homann, O. R., J. Dea, S. M. Noble, and A. D. Johnson, 2009 A phenotypic profile of the *Candida albicans* regulatory network. PLoS Genet. 5: e1000783.

Homann, O. R., and A. D. Johnson, 2010 MochiView: versatile software for genome browsing and DNA motif analysis. BMC Biol. 8: 49.

Lohse, M. B., A. D. Hernday, P. M. Fordyce, L. Noiman, T. R. Sorrells *et al.*, 2013 Identification and characterization of a previously undescribed family of sequence-specific DNA-binding domains. Proc. Natl. Acad. Sci. U. S. A. 110: 7660–7665.

Tuch, B. B., Q. M. Mitrovich, O. R. Homann, A. D. Hernday, C. K. Monighetti *et al.*, 2010 The transcriptomes of two heritable cell types illuminate the circuit governing their differentiation. PLoS Genet. 6: e1001070.

Zordan, R., M. Miller, D. Galgoczy, B. Tuch, and A. Johnson, 2007 Interlocking transcriptional feedback loops control white-opaque switching in *Candida albicans*. PLoS Biol. 5: e256.
