# Supplementary material for: Identification and Characterization of Wor4, a New Transcriptional Regulator of White-Opaque Switching
Source: G3 (Bethesda). 2016 Jan 13;6(3):721–9. doi: 10.1534/g3.115.024885 (PMC4777133; doi:10.1534/g3.115.024885)
Supplement: Supporting Information [file supp_g3.115.024885_FigureS3.pdf]

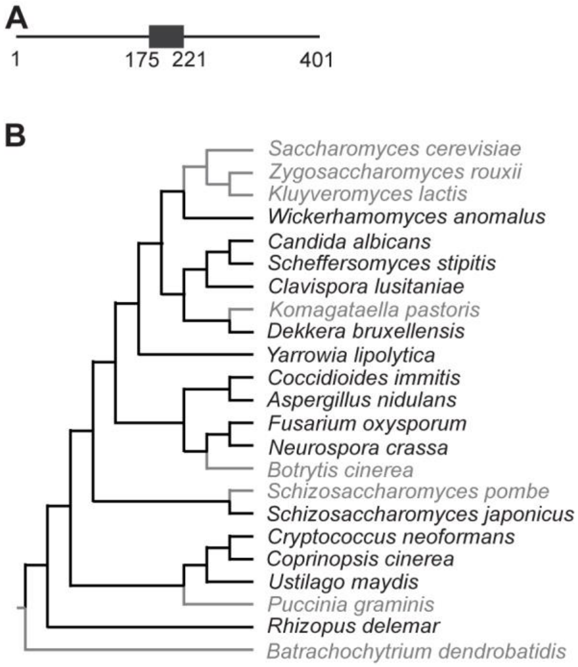

Figure S3: Wor4 belongs to the C2H2 Zinc Finger family of proteins and is found across the fungal domain. (a) Scale model of Wor4, the black box represents the 47aa (amino acids 175-221) region identified by HHpred as belonging to the C2H2 Zinc Finger family. ELM identified a similar, although smaller, region (amino acids 186-212) as belonging to the same family. (b) Phylogenetic tree of 23 fungal species, species with a Wor4 homolog are in black and species lacking a Wor4 homolog are in grey. Branch lengths are not to scale.
