# Supplementary material for: Identification and Characterization of Wor4, a New Transcriptional Regulator of White-Opaque Switching
Source: G3 (Bethesda). 2016 Jan 13;6(3):721–9. doi: 10.1534/g3.115.024885 (PMC4777133; doi:10.1534/g3.115.024885)
Supplement: Supporting Information [file supp_g3.115.024885_TableS1.pdf]

Table S1: Oligonucleotides used in this study.

Category	Name	Description	Oligonucleotide sequence
pMBL180 construction	MBL122	mCherry 5' HindIII	gttacaaagcttATG GTT TCT AAG GGT GAA GAA GAT
pMBL180 construction	MBL121	mCherry 3' PstI	ctcggactgcagTTAC TTG TAC AAT TCA TCC ATA CCA CC
pMBL180 construction	MBL354	SAT1 5' PstI	Gttaca ctgcag GAG TGA AAT TCT GGA AAT CTG GAA ATC
pMBL180 construction	MBL355	SAT1 3' HindIII	ctcgga ggatcc GCA GGA CCA CCT TTG ATT GTA AAT AG
pADH76 Construction	AHO696	GFP 5' AvaI	aaaactcgagcggatccccgggttaattaacggtatgtctaaaggtgaagaattattcactggtg
pADH76 Construction	AHO698	GFP 3' AvaI	cttcctcgagTTATTTGTACAATTCATCCATACCATG
pADH57 construction	AHO407	TDH3 5' XbaI	tgggtatctagatgctcctcgtcgacaacgac
pADH57 construction	AHO408	TDH3 3' BamHI	CCCGGGGGATCCTAATTTGATTGTAAAGTTTGTTGATGTTAATTG
WOR4 Ectopic Expression	MLP780	Wor4 5' BamHI	Gttaca GGATCC ATG TCG AGT GAT AAA CCT GAA CAA G
WOR4 Ectopic Expression	MLP781	Wor4 3' XmaI	CTC GGA CCC GGG TTA AAT GCC TGG TTG GGT TG
WOR4 Ectopic Expression	MLP1137	Wor4 3' PstI	CTC GGA CTG CAG TTA AAT GCC TGG TTG GGT TG

RFG1 Ectopic Expression	MLP777	Rfg1 5' BamHI	Gttaca GGATCC ATG TCT ACT GCT ATC TAC TAT TCA ACT C
RFG1 Ectopic Expression	MLP778	Rfg1 3' XmaI	CTC GGA CCC GGG TTA TTG TGG AGG TTG TTG TTG ATG AT
WOR4 Deletion	NHO320	WOR4 5' External	TTGGGCTGATCTATACTTCTGGGTA
WOR4 Deletion	NHO321	WOR4 5' Internal	CACGGCGCGCCTAGCAGCGGCCTCAAGCTGCAAAAATAGTTTCTCTC
WOR4 Deletion	NHO322	WOR4 3' Internal	GTCAGCGGCCGCATCCCTGCGAAATGAGGATTTGAATGATCCTCTGTTG
WOR4 Deletion	NHO323	WOR4 3' External	TCAGCACTATCCATATCTTCAGCCA
WOR4 Deletion	NHO324	WOR4 5' Check	GAAGGAATTGAGCAATAGGACAAAGATTTG
WOR4 Deletion	NHO325	WOR4 3' Check	AGCGATGAATCAACATCACCAACTTG
WOR4 Deletion	NHO326	WOR4 5' ORF Check Set 1	ATGCCAATTACCAACCTCAAGCTC
WOR4 Deletion	NHO327	WOR4 3' ORF Check Set 1	CCATACCAATAGTTTCCAGCAGT
WOR4 Deletion	MLP1051	WOR4 5' ORF Check Set 2	TAA CCA AAA AGC ATG GTC ACA GAA AAT C
WOR4 Deletion	MLP1052	WOR4 3' ORF Check Set 2	CAA CGT CAT TGA TTC CTA AGA GAG AC
WOR4 Deletion	MLp1053	WOR4 5' ORF Check Set 3	CCT AGA TCC AAA GGA TTC AAA AAA CGC
WOR4 Deletion	MLp1054	WOR4 3' ORF	CAG GAA ACG AAC AAG GAT ATT TTC TCT C

		Check Set 3	
RFG1 Deletion	NHO3 47	RFG1 5' External	GGTGACTCCGATTTTCCGAACTAA
RFG1 Deletion	NHO3 48	RFG1 5' Internal	CACGGCGCGCCTAGCAGCGGTGGTGTGATGGTTTGCAAATAAATAC
RFG1 Deletion	NHO3 49	RFG1 3' Internal	GTCAGCGGCCGCATCCCTGCGTAGCTTGCTTTCTCTACAGAAAAGAGAAT
RFG1 Deletion	NHO3 50	RFG1 3' External	AAGGTGGCCGTGTGAGAAAGTTTA
RFG1 Deletion	NHO3 51	RFG1 5' Check	CACACACCTGCACACCTACATTTG
RFG1 Deletion	NHO3 52	RFG1 3' Check	TTGGTTTAGGACCTGGGCGT
RFG1 Deletion	NHO3 53	RFG1 5' ORF Check Set	TTGGTGGTGGTATTGATGGTAACTTT
RFG1 Deletion	NH354	RFG1 3' ORF Check Set	ACGAGAAGTTAATCCACCACTGAGACT
WOR4 Tagging	MLP87 4	WOR4-13x Myc and -GFP 5'	Cttaggaatcaatgacgttgattcaagaaaccaaattgacgataacaaaccaacccaaccaggcatt cggatccccgggttaattaacgg
WOR4 Tagging	MLP87 5	WOR4-13x Myc and -GFP 3'	TTA ATT TTC TAA AAT AAA TCT ATA TGA ATA CAA CAG AGG ATC ATT CAA ATC CTC ATT TCA TTT A GGCGGCCGCTCTAGAACTAGTGGATC
WOR4 Tagging	mLp89 3	WOR4 5' check	Atgattacactgctggaaactattggtatg
WOR4 Tagging	MLP88 0	WOR4 3' check	GTG GTA CGT GAC AAT GAG GTT GGG
WOR4 Tagging	MLP87 6	myc 5' check	CCGTTAATTAACCCGGGGATC
WOR4 Tagging	MLP87 7	myc 3' in check	Ggaacttcagatccactagttctagagc

WOR4 Tagging	MLP878	myc 3' flipped check	TCACTAGTGAATTCGCGCTCGAG
WOR4 Tagging	MBL209	GFP 5' check	GGT TGG CCA TGG AAC TGG CA
WOR4 Tagging	MBL382	GFP 3' flipped check	GGT GAT GGT CCA GTC TTG TTA CCA GAC
HTB1 and HTB2 Tagging	MLP1194	HTB1-mCherry 5'	gccgtttccgaaggtaccagagccgtcacaaaatactcatctgcttctagt ggtAGAAGAatcCCAGGTttaattaac GTT TCT AAG GGT GAA GAA GAT A
HTB1 and HTB2 Tagging	MLP1195	HTB1-mCherry 3'	AAA AAA AAA GTG GGC AAC TAA AAA TAC AAT TGG GAG ACA ATA CAA GAT CCA TCA CAT CTA GCA GGA CCA CCT TTG ATT GTA AAT AG
HTB1 and HTB2 Tagging	MLP1192	HTB2-mCherry 5'	gctgtttctgaaggtactagagctgttaccaaatactcttctgcttctaat ggtAGAAGAatcCCAGGTttaattaac GTT TCT AAG GGT GAA GAA GAT A
HTB1 and HTB2 Tagging	MLP1193	HTB2-mCherry 3'	AAC AAT AAT TTG GAG AAA TAA ACC ATT CAT GAC AAA CCT CTC TCT CTC TTT CTT TTT TTA GCA GGA CCA CCT TTG ATT GTA AAT AG
HTB1 and HTB2 Tagging	mLP1198	HTB1 5' check	TCT CCT TCT CTC TCT TGT CAC TTC TTC TTC CTC
HTB1 and HTB2 Tagging	MLP1199	HTB1 3' check	CCA AGA GGT CCC TGA AAT TCA GAA GTT TCT TG
HTB1 and HTB2 Tagging	MLp1196	HTB2 5' check	CAA TAA CAA CAA GAG ATT CAC GTG ACA CAC AAA
HTB1 and	MLP1197	HTB2 3' check	GGA GAT AGA AAA GAA TTG GGT CCA ACA CCA

HTB2 Tagging			
HTB1 and HTB2 Tagging	MLP1203	mCherry 5' check	TTG TGG AGA CAA AAT ATC CCA GGC GAA TG
HTB1 and HTB2 Tagging	MLP1204	mCherry 3' Check	TTG ACC TCT TCA CGT ATA AAA CTA GAC CTC
qPCR	MLP1186	Wor1 5'	TGGGTATGGTAACCACCTT
qPCR	MLP1187	Wor1 3'	TGATACTACCTGTACCAGTCGCAA
qPCR	MLP1190	Dyn1 control 5'	CATCCAACACTTCCAACCAATTACATTA
qPCR	MLP1191	Dyn1 control 3'	TAATTCTTGTTCAAAAGCCAAAGATTCG
