# Supplementary material for: Identification and Characterization of Wor4, a New Transcriptional Regulator of White-Opaque Switching
Source: G3 (Bethesda). 2016 Jan 13;6(3):721–9. doi: 10.1534/g3.115.024885 (PMC4777133; doi:10.1534/g3.115.024885)
Supplement: Supporting Information [file supp_g3.115.024885_TableS2.pdf]

Table S2: Plasmids used in this study.

Description	Name	Reference
LEU2 Knock Out	pSN40	1
HIS1 Knock Out	pSN52	1
Arg MTL Knock Out Cassette	pJD1	2
p*MET3-blank-SAT*1	pADH33	3
p*TDH3-blank-SAT*1	pADH57	This Study
p*MET3-WOR1-SAT1*	pADH35	4
p*MET3-WOR4-SAT1*	pMBL640	This Study
p*MET3-RFG1-SAT1*	pMBL639	This Study
p*TDH3-WOR4-SAT1*	pMBL707	This Study
C-terminal CaGFP Source	pADH76	This Study
C-terminal 13x Myc Source	pADH34	5
C-terminal mCherry Source	pMBL180	This Study

References

1 Noble, S. M., and A. D. Johnson, 2005 Strains and strategies for large-scale gene deletion studies of the diploid human fungal pathogen *Candida albicans*. Eukaryot. Cell 4: 298–309.

2 Lin, C. H., S. Kabrawala, E. P. Fox, C. J. Nobile, A. D. Johnson et al., 2013 Genetic control of conventional and pheromone-stimulated biofilm formation in *Candida albicans*. PLoS Pathog. 9: e1003305.

3 Lohse, M. B., A. D. Hernday, P. M. Fordyce, L. Noiman, T. R. Sorrells et al., 2013 Identification and characterization of a previously undescribed family of sequence-specific DNA-binding domains. Proc. Natl. Acad. Sci. U. S. A. 110: 7660–7665.

4 Hernday, A. D., M. B. Lohse, P. M. Fordyce, C. J. Nobile, J. D. DeRisi et al., 2013 Structure of the Transcriptional Network Controlling White-Opaque Switching in *Candida albicans*. Mol. Microbiol. 90: 22–35.

5 Hernday, A. D., S. M. Noble, Q. M. Mitrovich, and A. D. Johnson, 2010 Genetics and molecular biology in *Candida albicans*. Methods Enzymol. 470: 737–758.
