# Supplementary material for: Identification and Characterization of Wor4, a New Transcriptional Regulator of White-Opaque Switching
Source: G3 (Bethesda). 2016 Jan 13;6(3):721–9. doi: 10.1534/g3.115.024885 (PMC4777133; doi:10.1534/g3.115.024885)
Supplement: Supporting Information [file supp_g3.115.024885_TableS3.pdf]

Table S3: Strains used in this study.

Description	Number	Genotype	Reference
Starting Strains			
a/α -His -Leu -Arg Strain	SNY152	*a/alpha leu2Δ/leu2Δ his1Δ/his1Δ*<br>*URA3/ura3Δ::imm*^{434} *IRO1/iro1Δ::imm*^{434}<br>*arg1::hisG/arg1::hisG*	1
a/a -His -Leu Strain	RZY47	*a/a leu2Δ/leu2Δ his1Δ/his1Δ*<br>*URA3/ura3Δ::imm434 IRO1/iro1Δ::imm434*	2
Controls			
Wild Type, White	WTwhite,<br>AHY304	*a/Δalpha C.m.LEU2/leu2Δ C.d.HIS1/his1Δ*<br>*URA3/ura3Δ::imm*^{434} *IRO1/iro1Δ::imm*^{434}<br>*arg1::hisG/arg1::hisG ΔMTLalpha ::ARG1*	3
Wild Type, Opaque	WTopaque,<br>AHY336	*a/Δalpha C.m.LEU2/leu2Δ C.d.HIS1/his1Δ*<br>*URA3/ura3Δ::imm*^{434} *IRO1/iro1Δ::imm*^{434}<br>*arg1::hisG/arg1::hisG ΔMTLalpha ::ARG1*	3
Wild Type, White	AHY135	*a/a C.m.LEU2/leu2Δ C.d.HIS1/his1Δ*<br>*URA3/ura3Δ::imm*^{434} *IRO1/iro1Δ::imm*^{434}	3
Wild Type, Opaque	AHY136	*a/a C.m.LEU2/leu2Δ C.d.HIS1/his1Δ*<br>*URA3/ura3Δ::imm*^{434} *IRO1/iro1Δ::imm*^{434}	3
Ectopic Expression Strains			
Wild Type, White,<br>p*MET3*-Blank	AHY214	*a/a C.m.LEU2/leu2*Δ C.d.HIS1/his1Δ<br>URA3/ura3Δ::imm^{434} IRO1/iro1Δ::imm^{434}<br>p*MET3*-blank; SAT1; RP10	3
Wild Type, Opaque,<br>p*MET3*-Blank	AHY375,<br>MLY1166	*a/a C.m.LEU2/leu2*Δ C.d.HIS1/his1Δ<br>URA3/ura3Δ::imm^{434} IRO1/iro1Δ::imm^{434}<br>p*MET3*-blank; SAT1; RP10	This study
Wild Type, White,<br>p*MET3-WOR1*	AHY204	*a/a C.m.LEU2/leu2*Δ C.d.HIS1/his1Δ<br>URA3/ura3Δ::imm^{434} IRO1/iro1Δ::imm^{434}<br>p*MET3-WOR1*; SAT1; RP10	3
Wild Type, Opaque,<br>p*MET3-WOR1*	MLY1165	*a/a C.m.LEU2/leu2*Δ C.d.HIS1/his1Δ<br>URA3/ura3Δ::imm^{434} IRO1/iro1Δ::imm^{434}<br>p*MET3-WOR1*; SAT1; RP10	This Study
Wild Type, White,<br>p*MET3-WOR4*	MLY1214	*a/a C.m.LEU2/leu2*Δ C.d.HIS1/his1Δ<br>URA3/ura3Δ::imm^{434} IRO1/iro1Δ::imm^{434}<br>p*MET3-WOR4*; SAT1; RP10	This Study
Wild Type, Opaque,<br>p*MET3-WOR4*	MLY1230	*a/a C.m.LEU2/leu2*Δ C.d.HIS1/his1Δ<br>URA3/ura3Δ::imm^{434} IRO1/iro1Δ::imm^{434}<br>p*MET3-WOR4*; SAT1; RP10	This Study

Wild Type, White, p*MET3-RFG1*	MLY1221	*a/a C.m.LEU2/leu2Δ* C.d.HIS1/his1Δ URA3/ura3Δ::imm^{434} IRO1/iro1Δ::imm^{434} p*MET3-RFG1*; SAT1; RP10	This Study
Wild Type, Opaque, p*MET3-RFG1*	MLY1241	*a/a C.m.LEU2/leu2Δ* C.d.HIS1/his1Δ URA3/ura3Δ::imm^{434} IRO1/iro1Δ::imm^{434} p*MET3-RFG1*; SAT1; RP10	This Study
wor4/wor4, White, p*MET3*-Blank	MLY1372, MLY1379	*a/Δalpha leu2Δ/leu2Δ his1Δ/his1Δ URA3/ura3Δ::imm^{434} IRO1/iro1Δ::imm^{434} arg1::hisG/arg1::hisG wor4Δ::C.m.LEU2/wor4Δ::C.d.HIS1 ΔMTLalpha ::ARG1* p*MET3*-blank; SAT1; RP10	This Study
wor4/wor4, White, p*MET3-WOR1*	MLY1373, MLY1380	*a/Δalpha leu2Δ/leu2Δ his1Δ/his1Δ URA3/ura3Δ::imm^{434} IRO1/iro1Δ::imm^{434} arg1::hisG/arg1::hisG wor4Δ::C.m.LEU2/wor4Δ::C.d.HIS1 ΔMTLalpha ::ARG1* p*MET3-WOR1*; SAT1; RP10	This Study
Wild Type, White, p*MET3*-Blank	MLY1365	*a/Δalpha C.m.LEU2/leu2Δ C.d.HIS1/his1Δ URA3/ura3Δ::imm^{434} IRO1/iro1Δ::imm^{434} arg1::hisG/arg1::hisG ΔMTLalpha ::ARG1 pMET3*-blank; SAT1; RP10	This Study
Wild Type, White, p*MET3-WOR1*	MLY1366	*a/Δalpha C.m.LEU2/leu2Δ C.d.HIS1/his1Δ URA3/ura3Δ::imm^{434} IRO1/iro1Δ::imm^{434} arg1::hisG/arg1::hisG ΔMTLalpha ::ARG1 pMET3-WOR1*; SAT1; RP10	This Study
wor1/wor1, White, p*MET3*-Blank	MLY1393	*a/a leu2Δ/leu2Δ his1Δ/his1Δ URA3/ura3Δ::imm^{434} IRO1/iro1Δ::imm^{434} wor1Δ::C.m.LEU2/wor1Δ::C.d.HIS1* p*MET3*-blank; SAT1; RP10	This Study
wor1/wor1, White, p*MET3-WOR1*	MLY1394	*a/a leu2Δ/leu2Δ his1Δ/his1Δ URA3/ura3Δ::imm^{434} IRO1/iro1Δ::imm^{434} wor1Δ::C.m.LEU2/wor1Δ::C.d.HIS1* p*MET3-WOR1*; SAT1; RP10	This Study
wor1/wor1, White, p*MET3-WOR4*	MLY1395	*a/a leu2Δ/leu2Δ his1Δ/his1Δ URA3/ura3Δ::imm^{434} IRO1/iro1Δ::imm^{434} wor1Δ::C.m.LEU2/wor1Δ::C.d.HIS1* p*MET3-WOR4*; SAT1; RP10	This Study
Wild Type, p*TDH3-WOR4*	MLY1420	*a/a C.m.LEU2/leu2Δ* C.d.HIS1/his1Δ URA3/ura3Δ::imm^{434} IRO1/iro1Δ::imm^{434} p*TDH3-WOR4*; SAT1; RP10	This Study
Deletion Strains			

wor1/wor1, White	RZY219	*a/a leu2Δ/leu2Δ his1Δ/his1Δ URA3/ura3Δ::imm^{434} IRO1/iro1Δ::imm^{434} wor1Δ::C.m.LEU2/wor1Δ::C.d.HIS1*	2
WOR4/wor4, White	MLY1135	*a/a leu2Δ/leu2Δ his1Δ/his1Δ URA3/ura3Δ::imm^{434} IRO1/iro1Δ::imm^{434} WOR4/wor4Δ::C.d.HIS1*	This Study
WOR4/wor4, Opaque	MLY1137	*a/a leu2Δ/leu2Δ his1Δ/his1Δ URA3/ura3Δ::imm^{434} IRO1/iro1Δ::imm^{434} WOR4/wor4Δ::C.d.HIS1*	This Study
wor4/wor4, White	MLY1355A, MLY1355B	*a/Δalpha leu2Δ/leu2Δ his1Δ/his1Δ URA3/ura3Δ::imm^{434} IRO1/iro1Δ::imm^{434} arg1::hisG/arg1::hisG wor4Δ::C.m.LEU2/wor4Δ::C.d.HIS1 ΔMTLalpha ::ARG1*	This Study
rfg1/rfg1, White	MLY1136	*a/a leu2Δ/leu2Δ his1Δ/his1Δ URA3/ura3Δ::imm^{434} IRO1/iro1Δ::imm^{434} rfg1Δ::C.m.LEU2/rfg1Δ::C.d.HIS1*	This Study
rfg1/rfg1, Opaque	MLY1138	*a/a leu2Δ/leu2Δ his1Δ/his1Δ URA3/ura3Δ::imm^{434} IRO1/iro1Δ::imm^{434} rfg1Δ::C.m.LEU2/rfg1Δ::C.d.HIS1*	This Study
Tagged Strains			
Wor4-GFP, White	MLY1295	*a/a C.m.LEU2/leu2Δ C.d.HIS1/his1Δ URA3/ura3Δ::imm^{434} IRO1/iro1Δ::imm^{434} WOR4/WOR4-GFP*	This Study
Wor4-GFP, Opaque	MLY1304	*a/a C.m.LEU2/leu2Δ C.d.HIS1/his1Δ URA3/ura3Δ::imm^{434} IRO1/iro1Δ::imm^{434} WOR4/WOR4-GFP*	This Study
Wor4-13x myc, White	MLY1286	*a/a C.m.LEU2/leu2Δ C.d.HIS1/his1Δ URA3/ura3Δ::imm^{434} IRO1/iro1Δ::imm^{434} WOR4/WOR4-13x myc*	This Study
Wor4-13x myc, Opaque	MLY1298	*a/a C.m.LEU2/leu2Δ C.d.HIS1/his1Δ URA3/ura3Δ::imm^{434} IRO1/iro1Δ::imm^{434} WOR4/WOR4-13x myc*	This Study
Microscopy Strains			
HTB1-mCherry, Wor4-GFP, White	MLY1460	*a/a C.m.LEU2/leu2Δ C.d.HIS1/his1Δ URA3/ura3Δ::imm^{434} IRO1/iro1Δ::imm^{434} WOR4/WOR4-GFP HTB1/HTB1-mCherry::SAT1*	This Study
HTB1-mCherry, Wor4-GFP, Opaque	MLY1467	*a/a C.m.LEU2/leu2Δ C.d.HIS1/his1Δ URA3/ura3Δ::imm^{434} IRO1/iro1Δ::imm^{434} WOR4/WOR4-GFP HTB1/HTB1-mCherry::SAT1*	This Study

HTB2-mCherry, Wor4-GFP, White	MLY1462	*a/a C.m.LEU2/leu2Δ C.d.HIS1/his1Δ URA3/ura3Δ::*imm^{434} *IRO1/iro1Δ::*imm^{434} *WOR4/WOR4-GFP HTB2/HTB2-mCherry::SAT1*	This Study
HTB2-mCherry, Wor4-GFP, Opaque	MLY1469	*a/a C.m.LEU2/leu2Δ C.d.HIS1/his1Δ URA3/ura3Δ::*imm^{434} *IRO1/iro1Δ::*imm^{434} *WOR4/WOR4-GFP HTB2/HTB2-mCherry::SAT1*	This Study

References

1 Noble, S. M., and A. D. Johnson, 2005 Strains and strategies for large-scale gene deletion studies of the diploid human fungal pathogen *Candida albic*ans. Eukaryot. Cell 4: 298–309.

2 Zordan, R. E., D. J. Galgoczy, and A. D. Johnson, 2006 Epigenetic properties of white-opaque switching in *Candida albicans* are based on a self-sustaining transcriptional feedback loop. Proc. Natl. Acad. Sci. U. S. A. 103: 12807–12812.

3 Hernday, A. D., M. B. Lohse, P. M. Fordyce, C. J. Nobile, J. D. DeRisi et al., 2013 Structure of the Transcriptional Network Controlling White-Opaque Switching in *Candida albicans*. Mol. Microbiol. 90: 22–35.
