# Supplementary material for: Identification and Characterization of Wor4, a New Transcriptional Regulator of White-Opaque Switching
Source: G3 (Bethesda). 2016 Jan 13;6(3):721–9. doi: 10.1534/g3.115.024885 (PMC4777133; doi:10.1534/g3.115.024885)
Supplement: Supporting Information [file supp_g3.115.024885_TableS4.pdf]

Table S4: Ectopic expression of *WOR4* does not induce white-to-opaque switching in a *wor1* deletion strain.
White-to-Opaque Switching

Strain	Media Condition	Switching Frequency (%)	n	Notes
Wild Type, p*MET*3-Blank	Repressing	<0.48	208	1
Wild Type, p*MET*3-Blank	Inducing	<0.26	378	1
Wild Type, p*MET*3-*WOR1*	Repressing	<0.48	208	2
Wild Type, p*MET*3-*WOR1*	Inducing	96.98	430	2
Wild Type, p*MET*3-*WOR4*	Repressing	<0.48	210	
Wild Type, p*MET*3-*WOR4*	Inducing	20.71	449	
wor1/wor1, p*MET3*-Blank	Repressing	<0.46	219	
wor1/wor1, p*MET3*-Blank	Inducing	<0.23	432	
wor1/wor1, p*MET3-WOR1*	Repressing	<0.49	205	
wor1/wor1, p*MET3-WOR1*	Inducing	100.00	383	
wor1/wor1, p*MET3-WOR4*	Repressing	<0.37	272	
wor1/wor1, p*MET3-WOR4*	Inducing	<0.18	546	

Notes

1 Negative Control
2 Positive Control

Table S4: Ectopic expression of *WOR4* does not induce white-to-opaque switching in a *wor1* deletion strain. Switching frequency and number of colonies scored for ectopic expression of *WOR1* and *WOR4* in the *wor1* deletion background are indicated.
