# Supplementary material for: Identification and Characterization of Wor4, a New Transcriptional Regulator of White-Opaque Switching
Source: G3 (Bethesda). 2016 Jan 13;6(3):721–9. doi: 10.1534/g3.115.024885 (PMC4777133; doi:10.1534/g3.115.024885)
Supplement: Supporting Information [file supp_g3.115.024885_TableS5.pdf]

Table S5: Breakdown of overlap between Wor4 binding and binding of other core regulators in white cells.

Class	Observed Binding	Number of Regions	Percent of Wor4 sites
Single TR	Wor4 alone	2	22.22
Two TR	Ahr1	1	11.11
Two TR	Czf1	1	11.11
Two TR	Efg1	3	33.33
Three TR	Ahr1+Czf1	1	11.11
Three TR	Ahr1+Efg1	1	11.11
Total		9	100.00
With Ahr1		3	33.33
With Czf1		2	22.22
With Efg1		4	44.44
Two or More		7	77.78
Three or More		2	22.22

Table S5: Breakdown of overlap between Wor4 binding and binding of other core regulators in white cells. Instances of specific binding combinations, overall overlap with specific regulators, and the number of sites with at least a given number of regulators bound are indicated. Only binding events with Wor4 present are considered. Binding of Ahr1, Czf1, and Efg1 have been previously reported (Hernday *et al.* 2013).
