# Supplementary material for: Identification and Characterization of Wor4, a New Transcriptional Regulator of White-Opaque Switching
Source: G3 (Bethesda). 2016 Jan 13;6(3):721–9. doi: 10.1534/g3.115.024885 (PMC4777133; doi:10.1534/g3.115.024885)
Supplement: Supporting Information [file supp_g3.115.024885_TableS6.pdf]

Table S6: Breakdown of overlap between Wor4 binding and binding of other core regulators in opaque cells.

Class	Observed Binding	Number of Regions	Percent of Wor4 sites
Single TR	Wor4 alone	4	2.92
Two TR	Wor1	8	5.84
Two TR	Wor2	3	2.19
Two TR	Wor3	1	0.73
Three TR	Ahr1+Wor2	1	0.73
Three TR	Efg1+Wor1	1	0.73
Three TR	Efg1+Wor2	1	0.73
Three TR	Wor1+Wor2	6	4.38
Four TR	Ahr1+Wor1+Wor2	2	1.46
Four TR	Czf1+Wor2+Wor3	1	0.73
Four TR	Efg1+Wor1+Wor2	27	19.71
Five TR	Ahr1+Efg1+Wor1+Wor2	12	8.76
Five TR	Ahr1+Wor1+Wor2+Wor3	2	1.46
Five TR	Czf1+Efg1+Wor1+Wor2	7	5.11
Five TR	Efg1+Wor1+Wor2+Wor3	17	12.41
Six TR	Ahr1+Czf1+Efg1+Wor1+Wor2	3	2.19
Six TR	Ahr1+Efg1+Wor1+Wor2+Wor3	14	10.22
Six TR	Czf1+Efg1+Wor1+Wor2+Wor3	5	3.65
Seven TR	Ahr1+Czf1+Efg1+Wor1+Wor2+Wor3	22	16.06
Total		137	100.00
With Ahr1		56	40.88
With Czf1		38	27.74
With Efg1		109	79.56
With Wor1		126	91.97
With Wor2		123	89.78
With Wor3		62	45.26
Two or More		133	97.08
Three or More		121	88.32
Four or More		112	81.75
Five or More		82	59.85
Six or More		44	32.12
Seven or More		22	16.06

Table S6: Breakdown of overlap between Wor4 binding and binding of other core regulators in opaque cells. Instances of specific binding combinations, overall overlap with specific regulators, and the number of sites with at least a given number of regulators bound are indicated. Only binding events with Wor4 present are considered. Binding of Ahr1, Czf1, Efg1, Wor1, Wor2, and Wor3 have been previously reported (Zordan *et al.* 2007; Hernday *et al.* 2013; Lohse *et al.* 2013).
