# Supplementary material for: Identification and Characterization of Wor4, a New Transcriptional Regulator of White-Opaque Switching
Source: G3 (Bethesda). 2016 Jan 13;6(3):721–9. doi: 10.1534/g3.115.024885 (PMC4777133; doi:10.1534/g3.115.024885)

File S2: MochiView image plots of 12kb regions centered on the sets of Wor4 binding sites in white and opaque cells. Plots produced using the SnapShot Function in MochiView v1.46

Full genome chromatin immunoprecipitation (ChIP-seq) mapping of Wor4 binding sites in white cells.

Plots of 12kb regions centered on the set of 9 Wor4 binding sites in white cells. Read counts for the Wor4-GFP strain repeats are shown in red, blue, and green. Read counts for the untagged strain repeats are shown in grey. The called peaks of Wor4 binding are indicated by the brown boxes in the lower track in each image. Peaks are arranged in order of decreasing Wor4 enrichment. Read counts are indicated on the y-axis. Chromosomal locations and specific enrichment levels for the peak are indicated in the strip above each panel; when multiple peaks are present the enrichment value corresponds to the peak at the center of the plot. Yellow boxes correspond to genes. Genes plotted above the bold line read in the sense direction; genes plotted below the line read in the antisense direction. Plots produced using the SnapShot Function in MochiView v1.46.

[3.81] Ca21chr1_C_albicans_SC5314:2222133-2234132 [+] [WOR1]

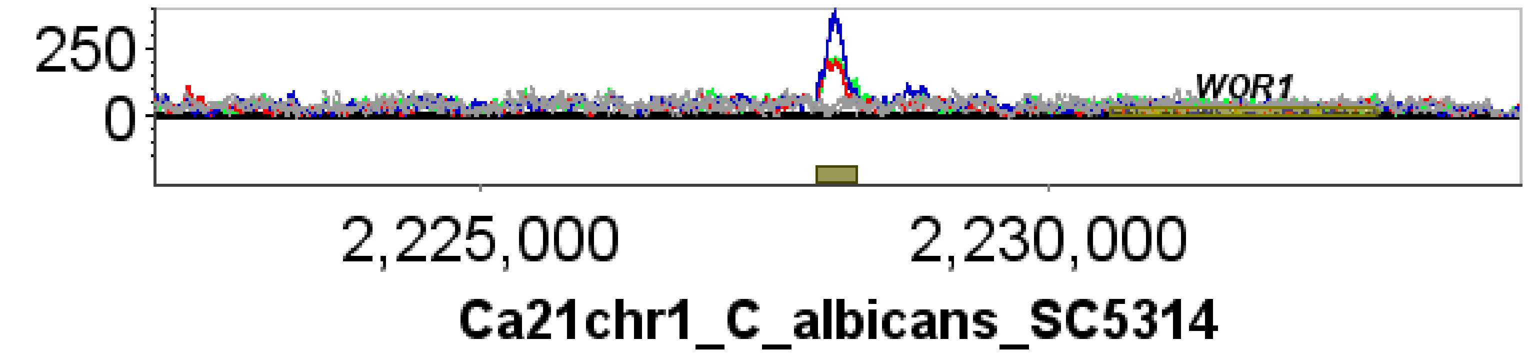

[3.625] Ca21chr2_C_albicans_SC5314:1740473-1752472 [+] [YWP1, GTR1, ERG9, orf19.3621]

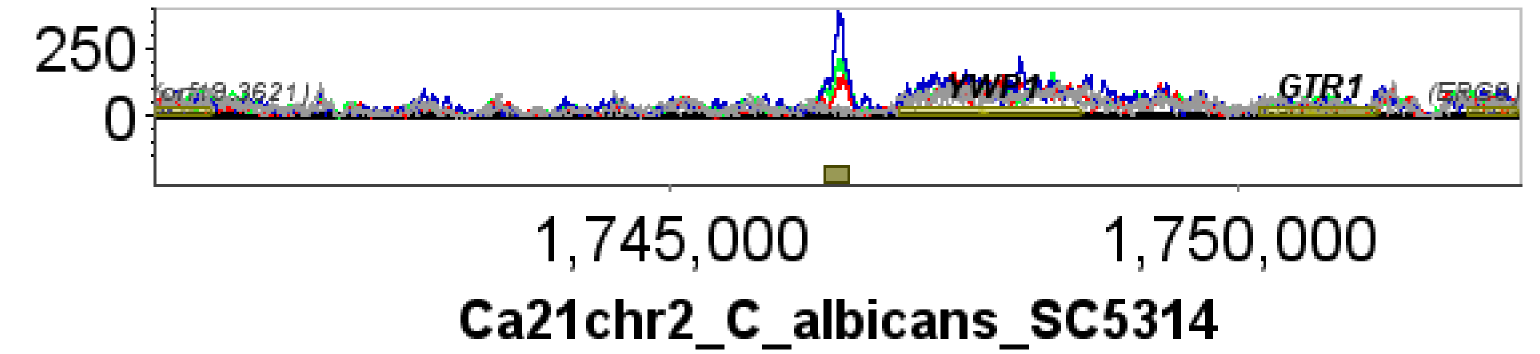

[3.38] Ca21chr1_C_albicans_SC5314:1076227-1088226 [+] [BRG1, orf19.450]

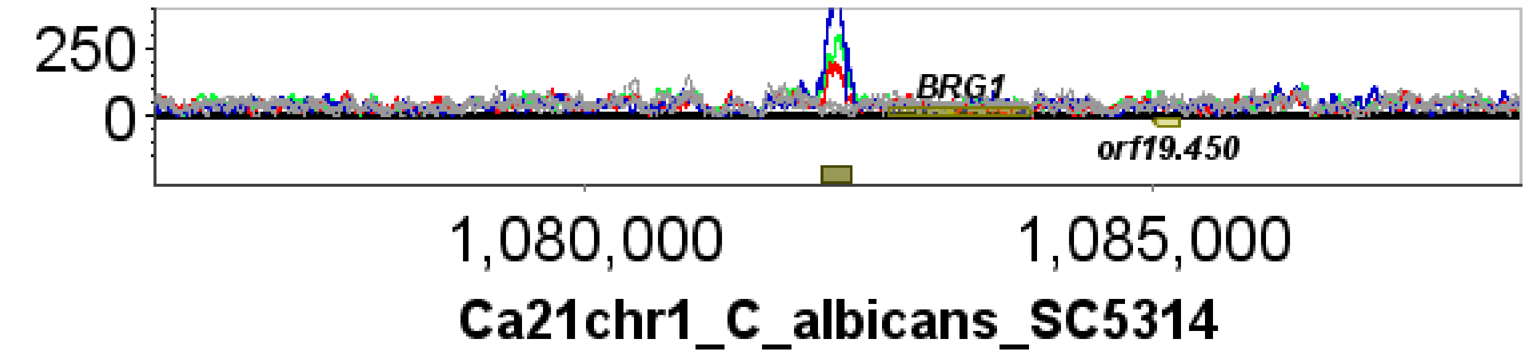

[3.165] Ca21chr3_C_albicans_SC5314:1216918-1228917 [+] [orf19.6984, orf19.6983]

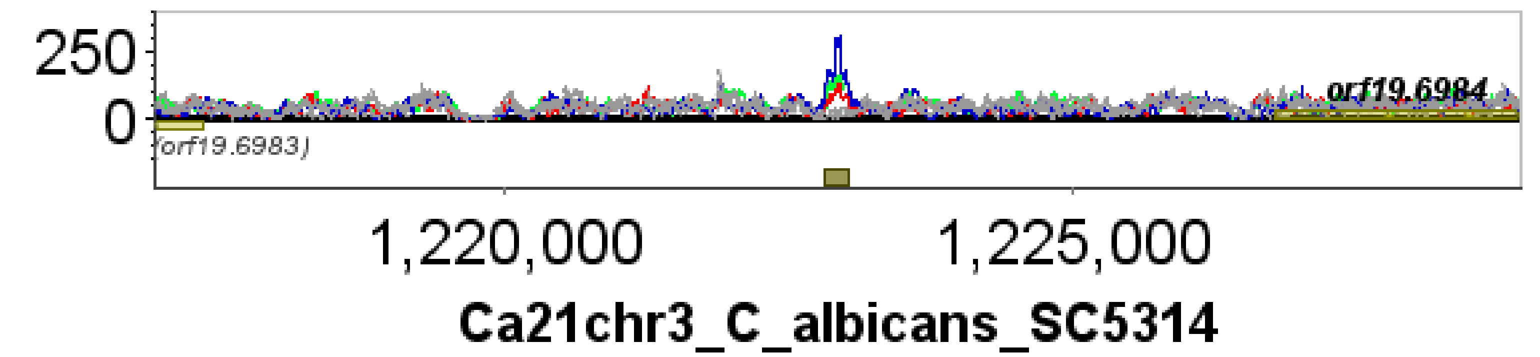

[3.15] Ca21chr3_C_albicans_SC5314:1723010-1735009 [+] [TCC1, orf19.6732, orf19.6731.1, orf19.6731, orf19.6730]

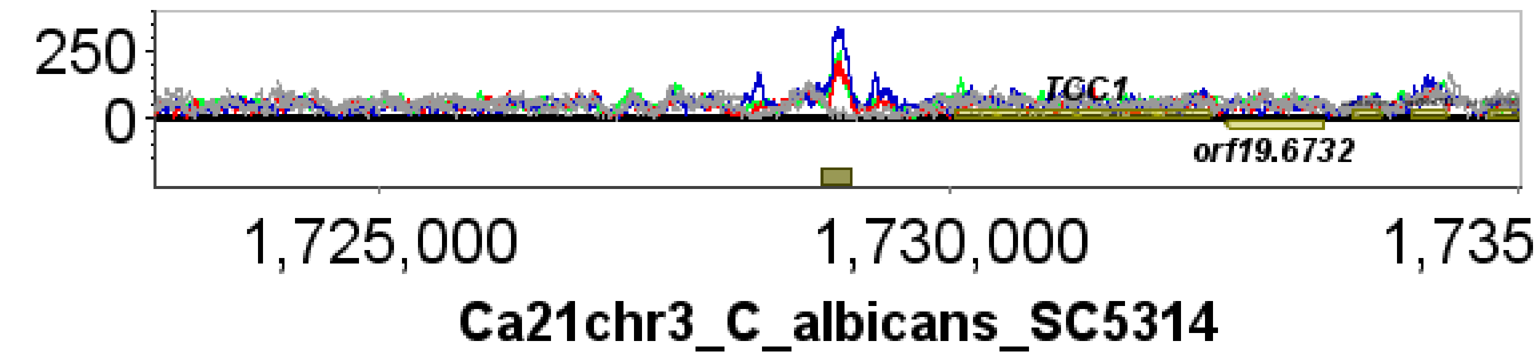

[2.99] Ca21chrR_C_albicans_SC5314:2163047-2175046 [+] [orf19.7590, FAA4, orf19.7589, orf19.7588, CHT3]

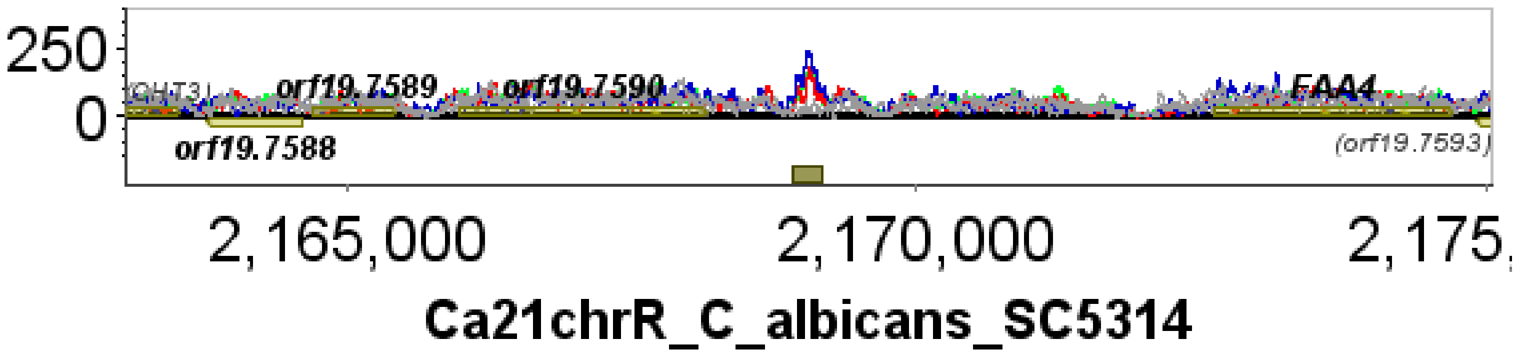

[2.94] Ca21chr3_C_albicans_SC5314:567990-579989 [+] [orf19.257, ZCF1, orf19.258, orf19.259, orf19.254]

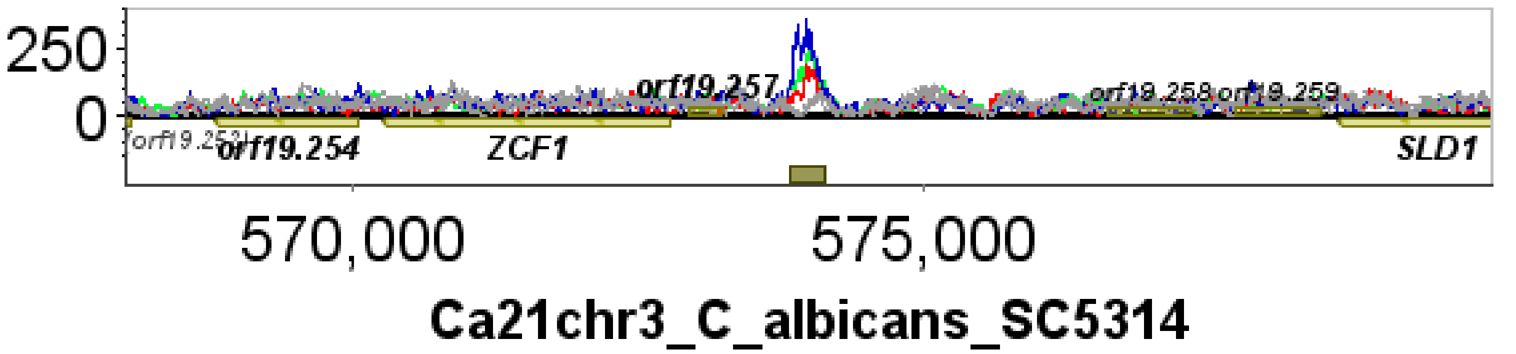

[2.695] Ca21chr2_C_albicans_SC5314:198245-210244 [+] [orf19.2002, HNM1, orf19.2001, REG1, orf19.2000]

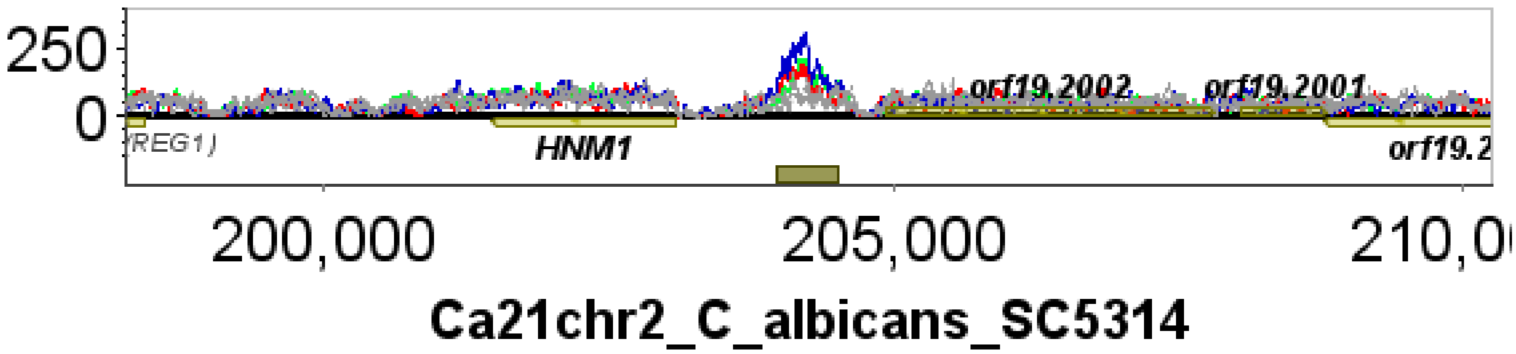

[2.59] Ca21chrR_C_albicans_SC5314:1515627-1527626 [+] [URA2, CRZ2]

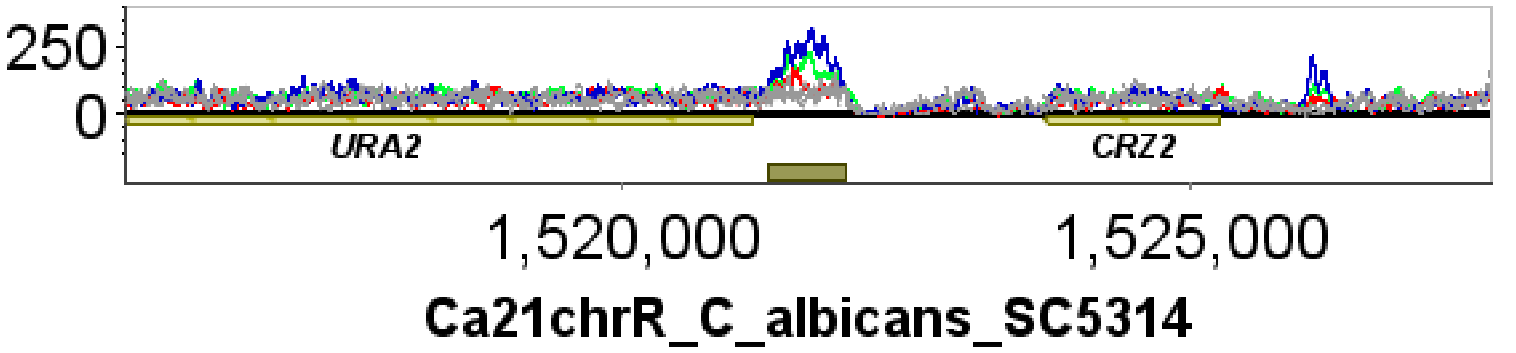

Full genome chromatin immunoprecipitation (ChIP-seq) mapping of Wor4 binding sites in opaque cells.

Plots of 12kb regions centered on the set of 244 Wor4 binding sites in opaque cells. Read counts for the Wor4-GFP strain repeats are shown in red, blue, and green. Read counts for the untagged strain repeats are shown in grey. The called peaks of Wor4 binding are indicated by the brown boxes in the lower track in each image. Peaks are arranged in order of decreasing Wor4 enrichment. Read counts are indicated on the y-axis. Chromosomal locations and specific enrichment levels for the peak are indicated in the strip above each panel; when multiple peaks are present the enrichment value corresponds to the peak at the center of the plot. Yellow boxes correspond to genes. Genes plotted above the bold line read in the sense direction; genes plotted below the line read in the antisense direction. Plots produced using the SnapShot Function in MochiView v1.46.

[16.98] Ca21chr1_C_albicans_SC5314:2218499-2230498 [+] [orf19.4883]

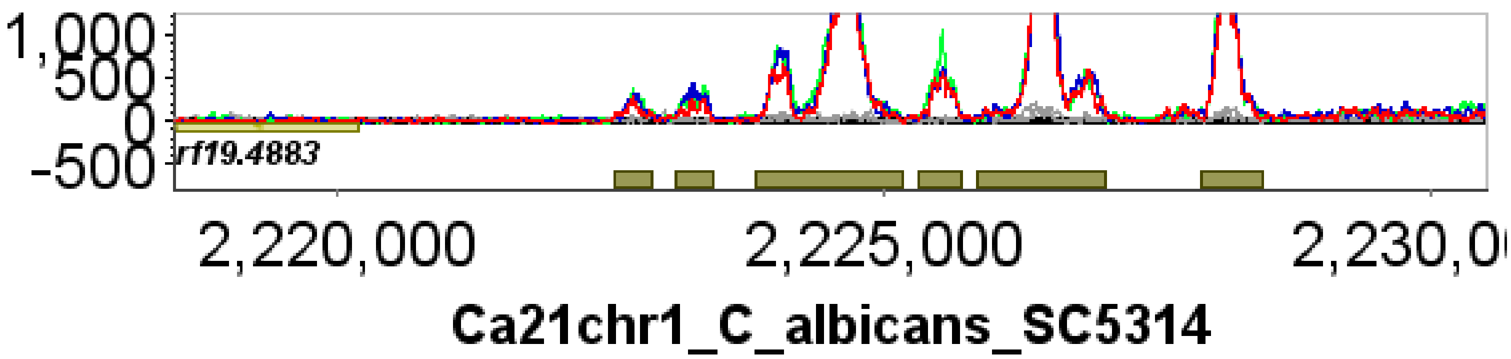

[15.76] Ca21chr1_C_albicans_SC5314:1323855-1335854 [+] [snR5b, snR44a, orf19.1821]

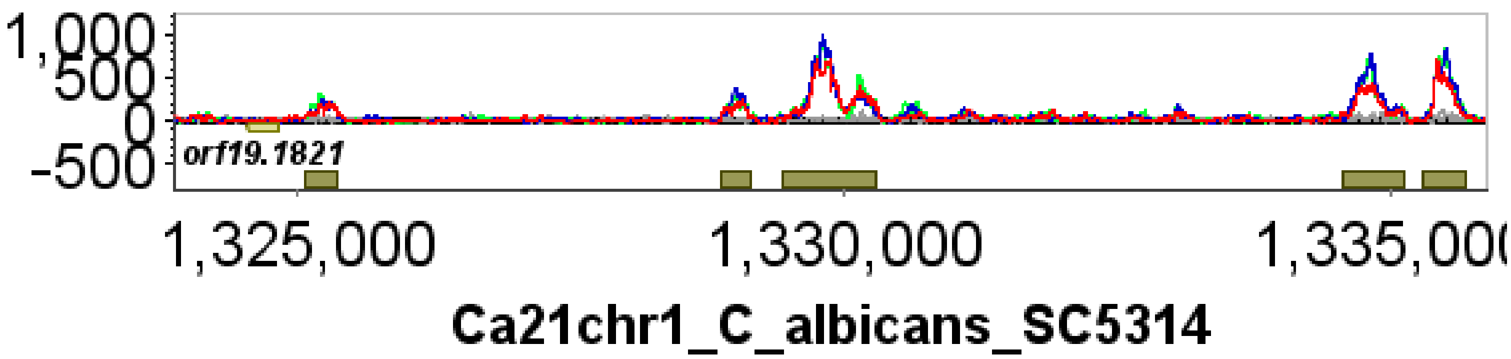

[14.83] Ca21chr4_C_albicans_SC5314:1517358-1529357 [+] [CZF1, orf19.3128, CCT6]

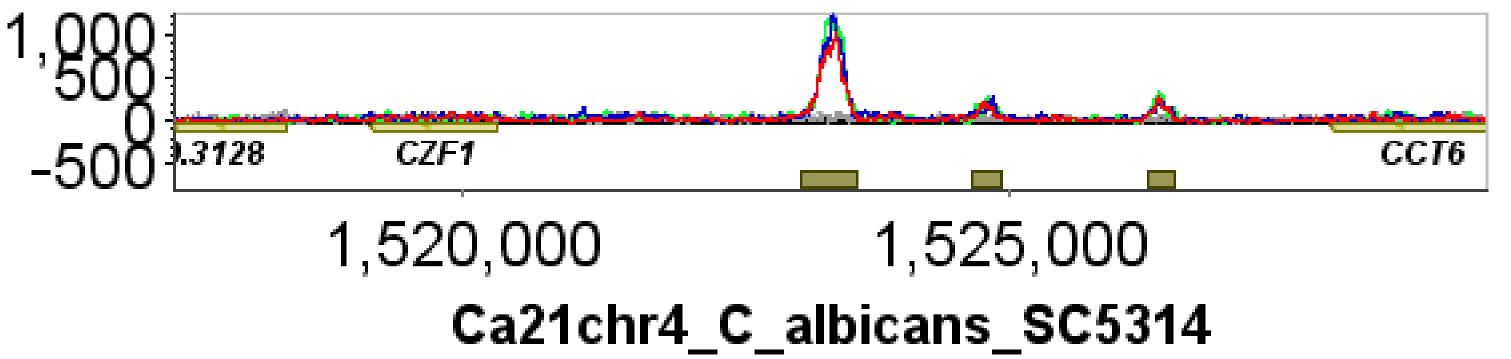

[14.66] Ca21chrR_C_albicans_SC5314:591915-603914 [+] [RFG1]

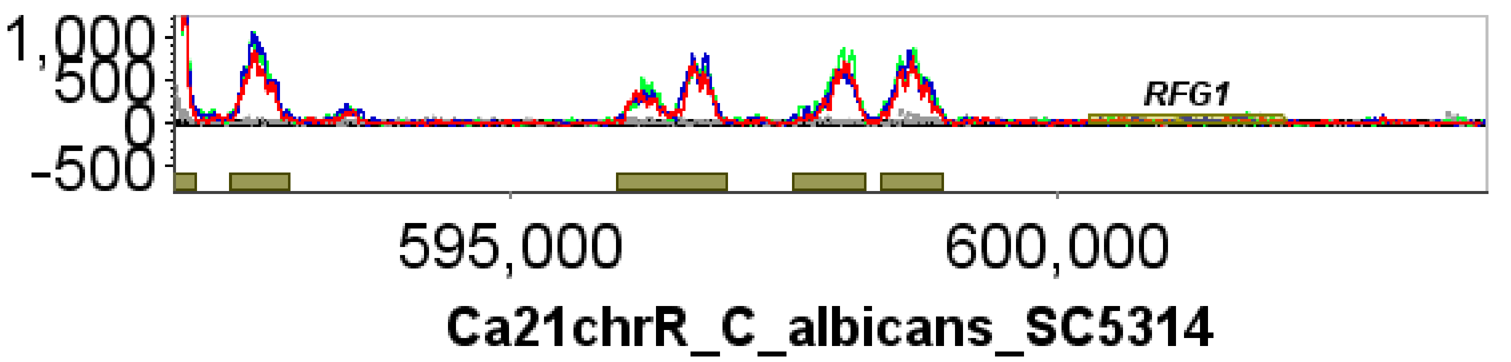

[14.5] Ca21chr7_C_albicans_SC5314:106342-118341 [+] [orf19.7054, GAC1, orf19.7055]

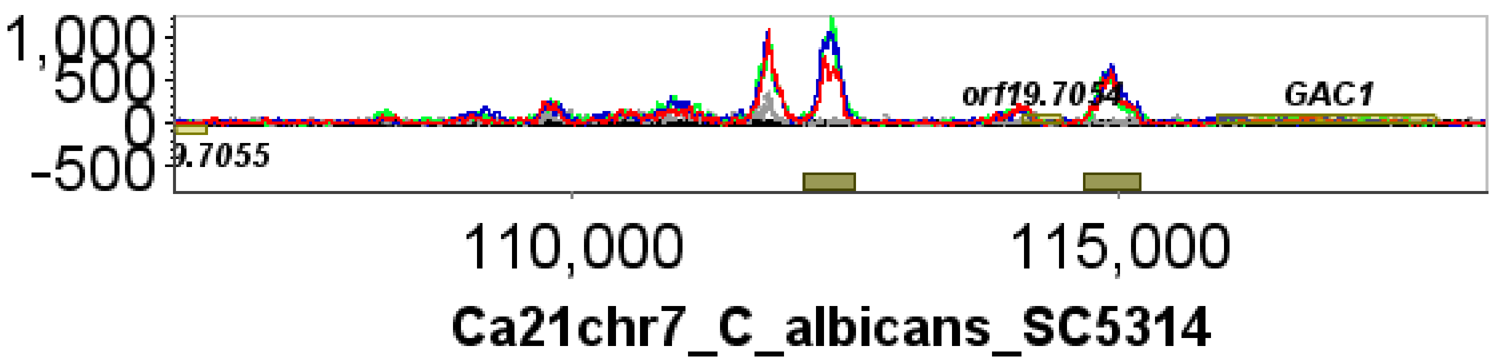

[14.16] Ca21chr3_C_albicans_SC5314:1718532-1730531 [+] [orf19.6736, TCC1]

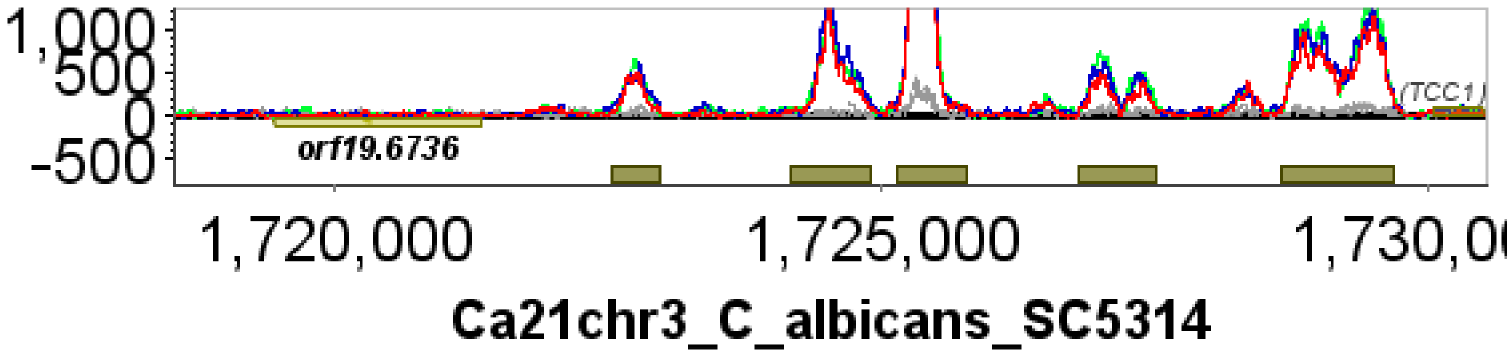

[13.71] Ca21chr3_C_albicans_SC5314:1110337-1122336 [+] [WOR2]

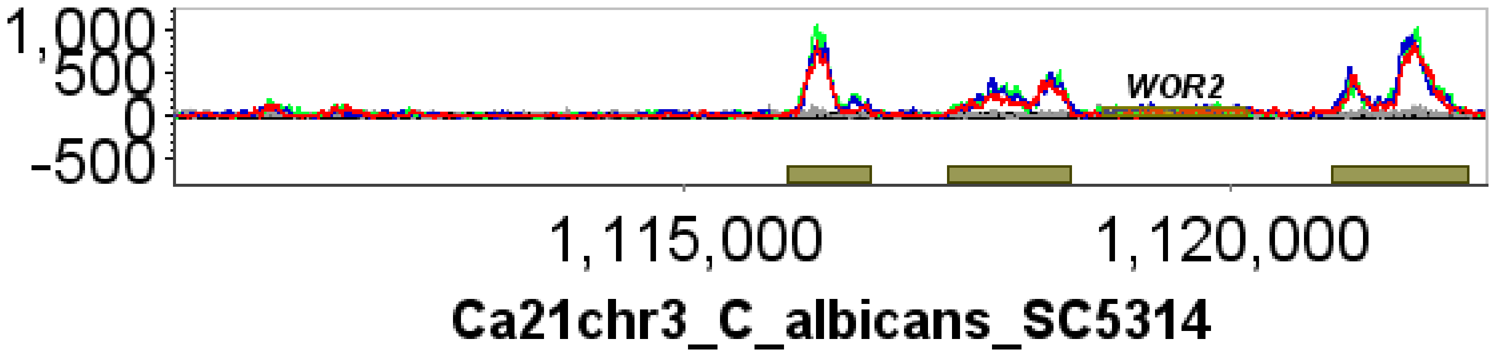

[13.57] Ca21chr1_C_albicans_SC5314:1084095-1096094 [+] [orf19.449, orf19.450, CUE5]

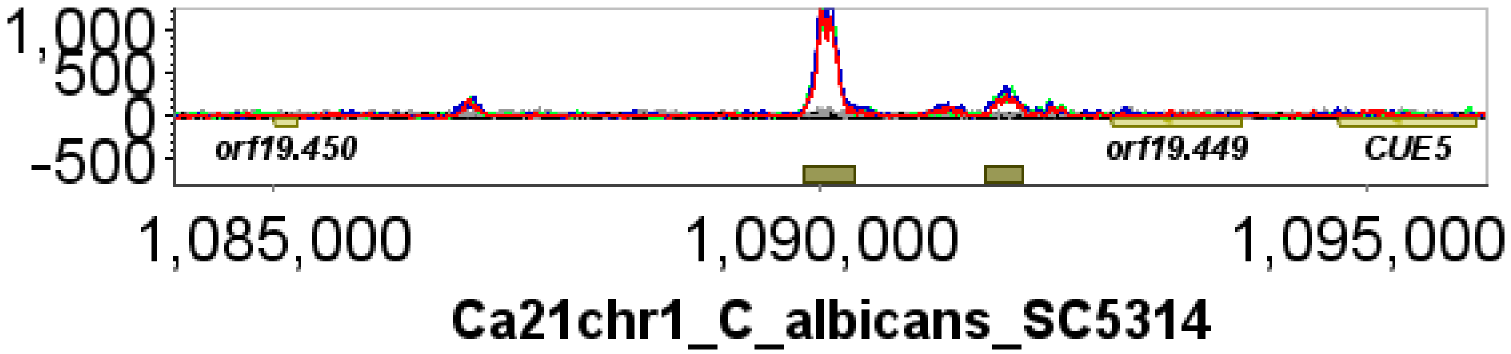

[13.34] Ca21chrR_C_albicans_SC5314:586703-598702 [+]

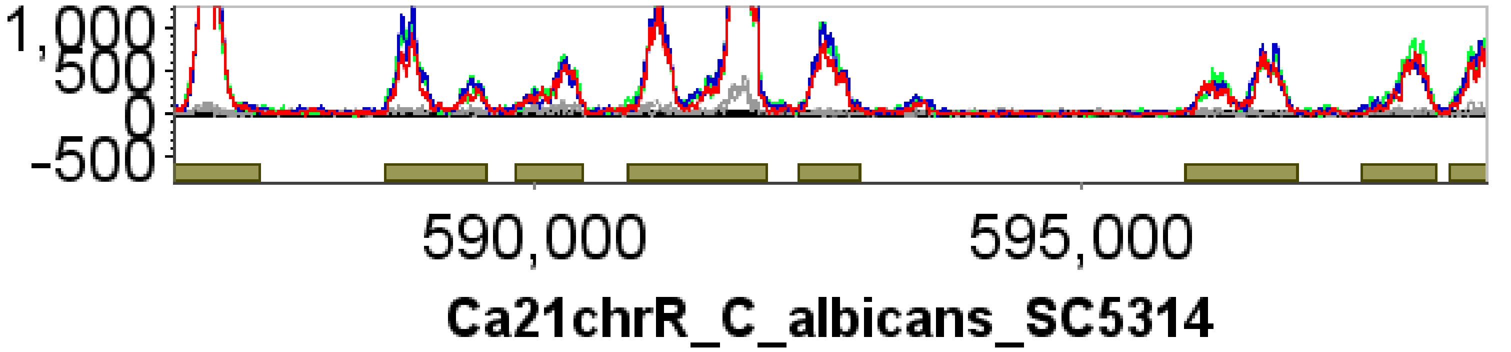

[13.25] Ca21chr3_C_albicans_SC5314:1752927-1764926 [+] [orf19.6715, orf19.6713, ABD1, orf19.6717, orf19.6718]

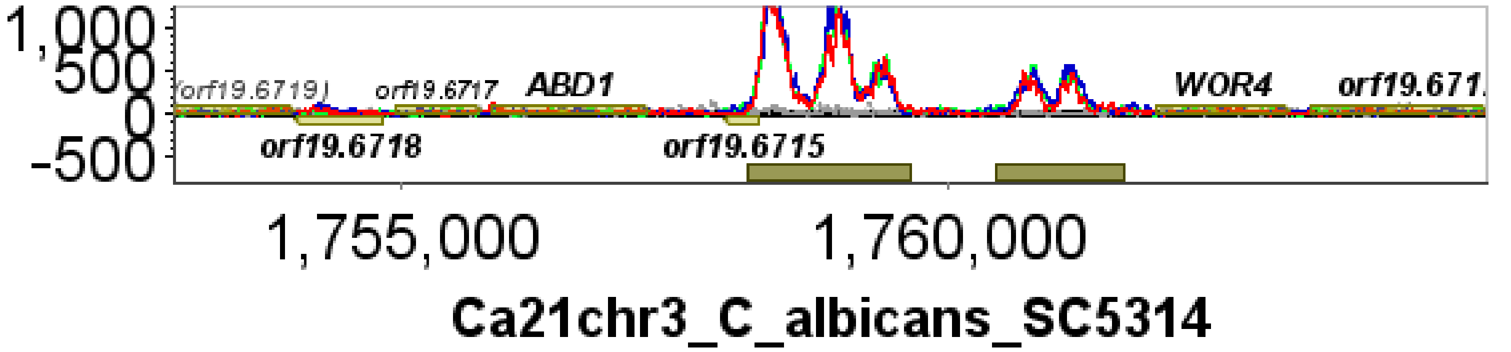

[13.18] Ca21chrR_C_albicans_SC5314:590476-602475 [+] [RFG1]

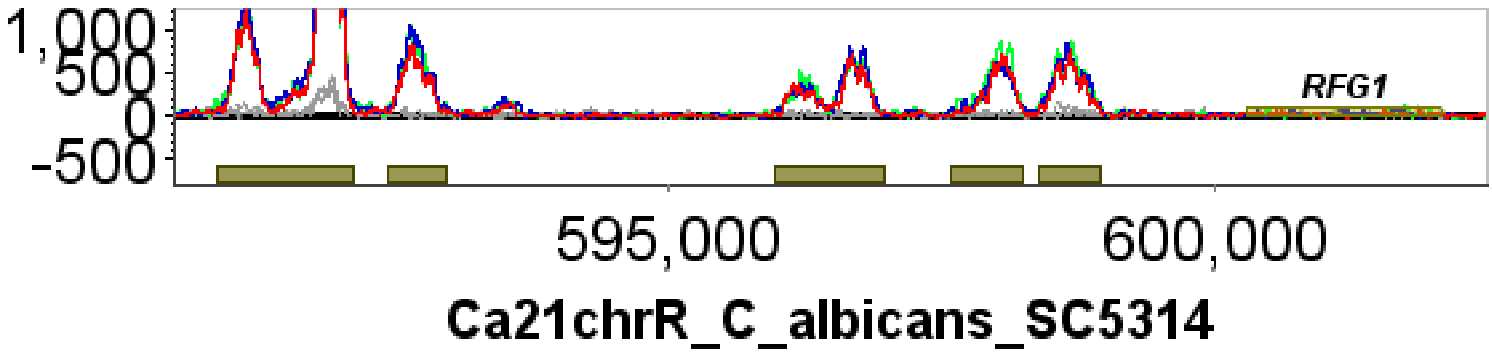

[12.97] Ca21chr2_C_albicans_SC5314:1799280-1811279 [+] [BUD20, PIR1, orf19.217, orf19.223]

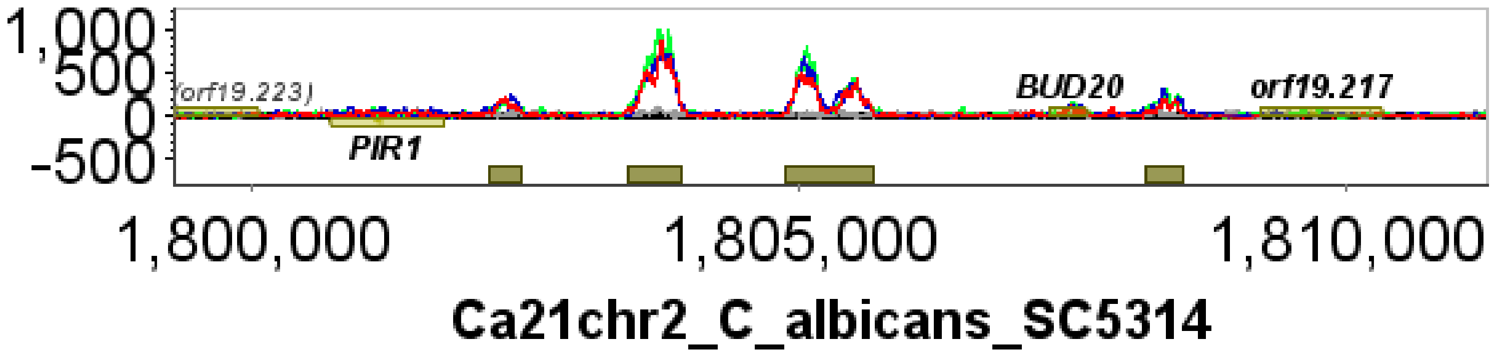

[12.79] Ca21chrR_C_albicans_SC5314:1715891-1727890 [+] [EFG1, orf19.609, orf19.607]

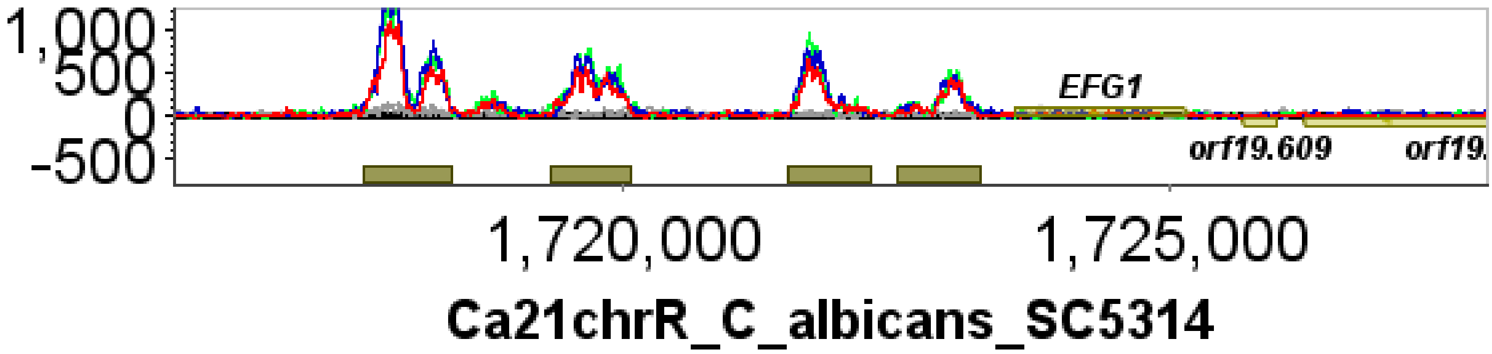

[12.58] Ca21chr3_C_albicans_SC5314:567776-579775 [+] [orf19.257, ZCF1, orf19.258, orf19.254, orf19.259]

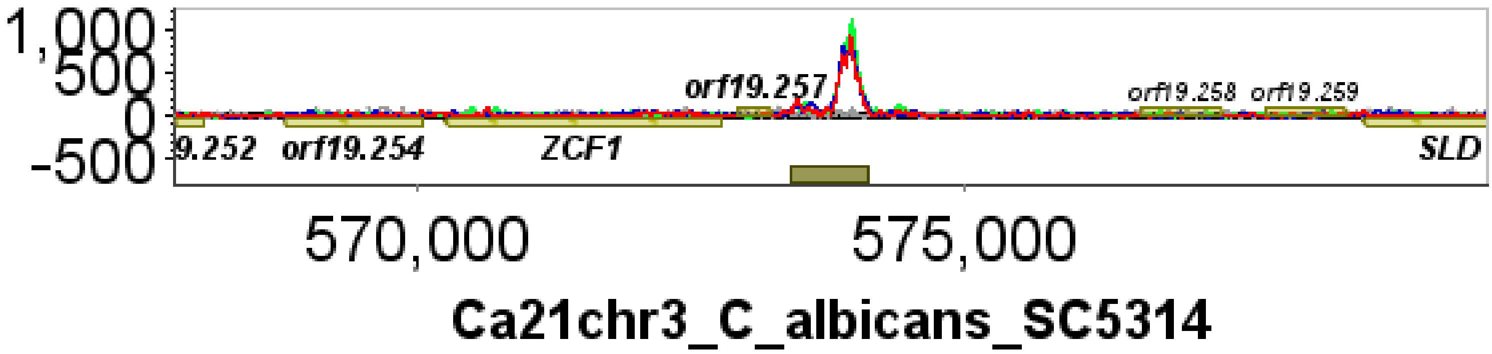

[12.48] Ca21chr7_C_albicans_SC5314:917847-929846 [+] [orf19.7149, TPO2, NRG1]

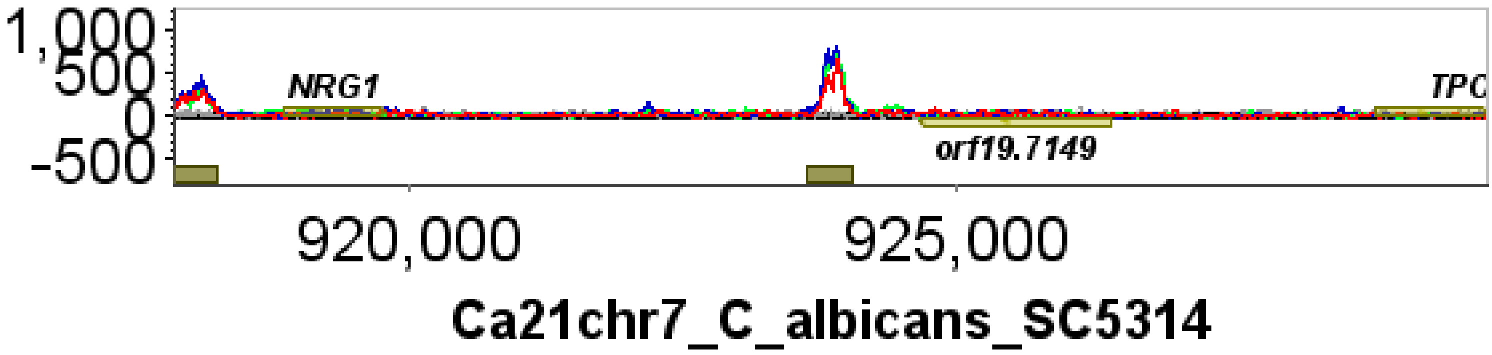

[12.25] Ca21chrR_C_albicans_SC5314:1307642-1319641 [+] [orf19.3868, orf19.3869, RPL7, ADE13]

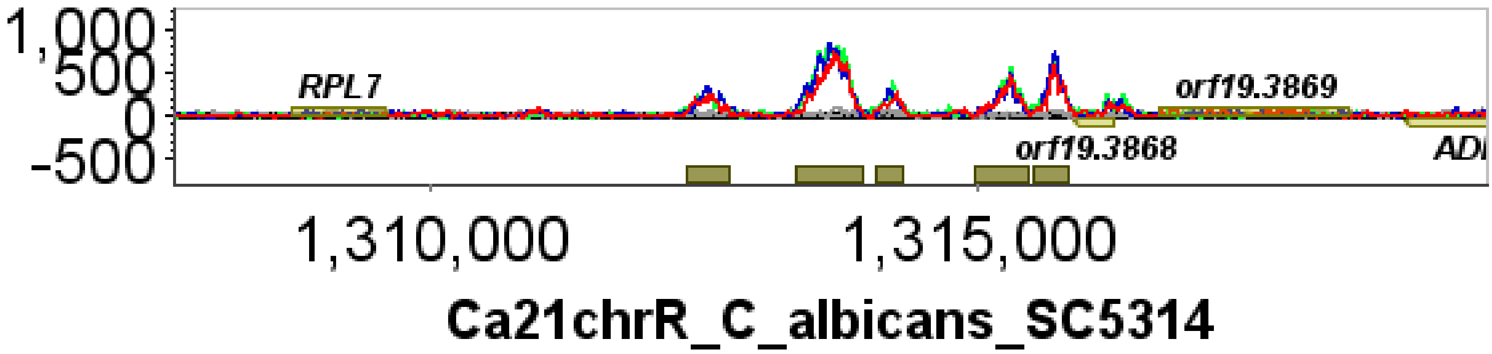

[12.25] Ca21chr3_C_albicans_SC5314:1642504-1654503 [+] [BMT1, ZFU2, MET8, PRO2]

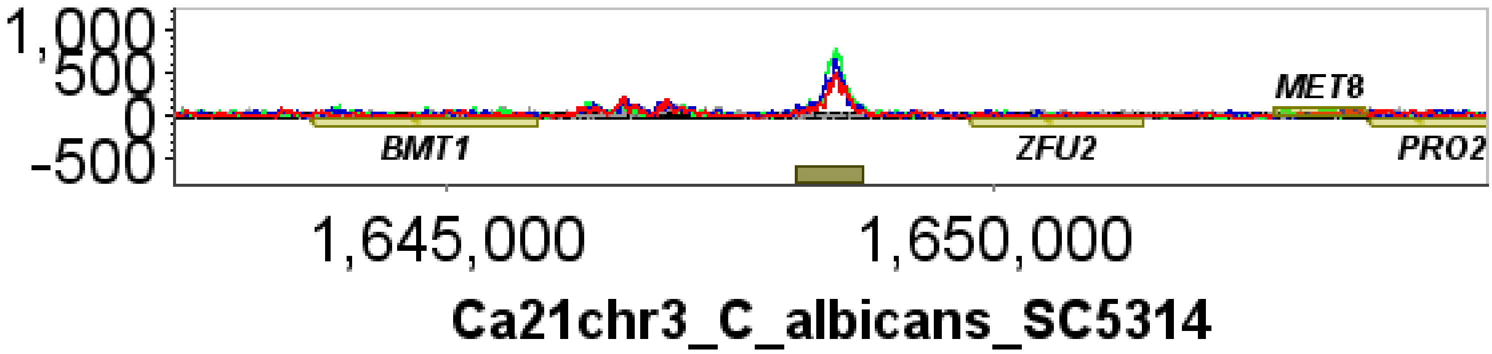

[12.22] Ca21chr5_C_albicans_SC5314:889617-901616 [+] [orf19.1286, orf19.1285, orf19.1287, FGR42]

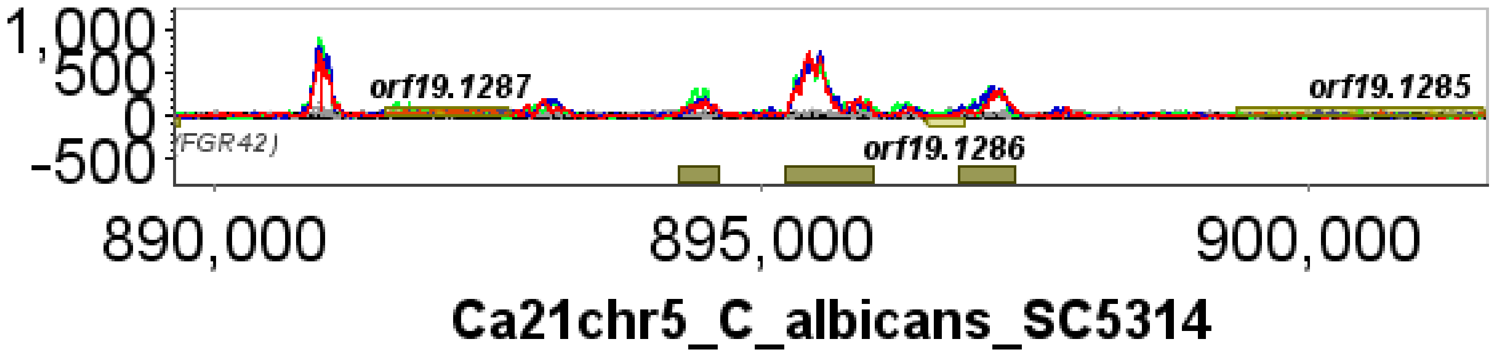

[12.15] Ca21chr2_C_albicans_SC5314:1866334-1878333 [+] [IFF6, MET10]

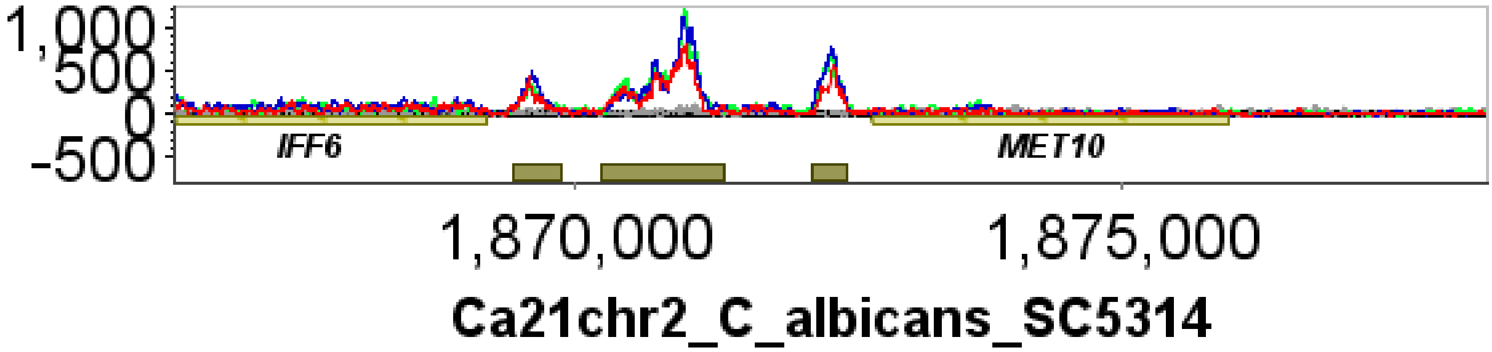

[12.05] Ca21chr5_C_albicans_SC5314:110766-122765 [+] [orf19.934, orf19.933, AGA1]

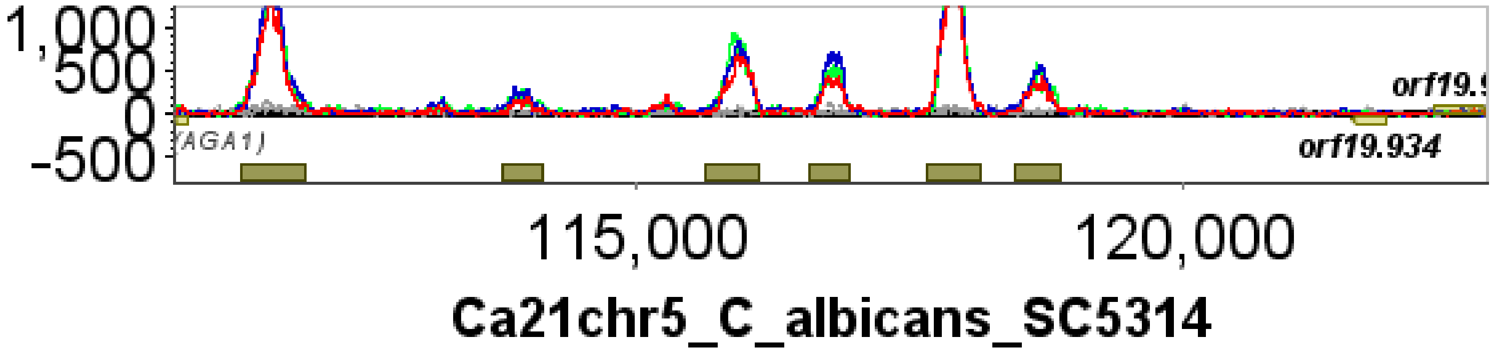

[11.98]   Ca21chr1_C_albicans_SC5314:2219512-2231511 [+]   [WOR1, orf19.4883]

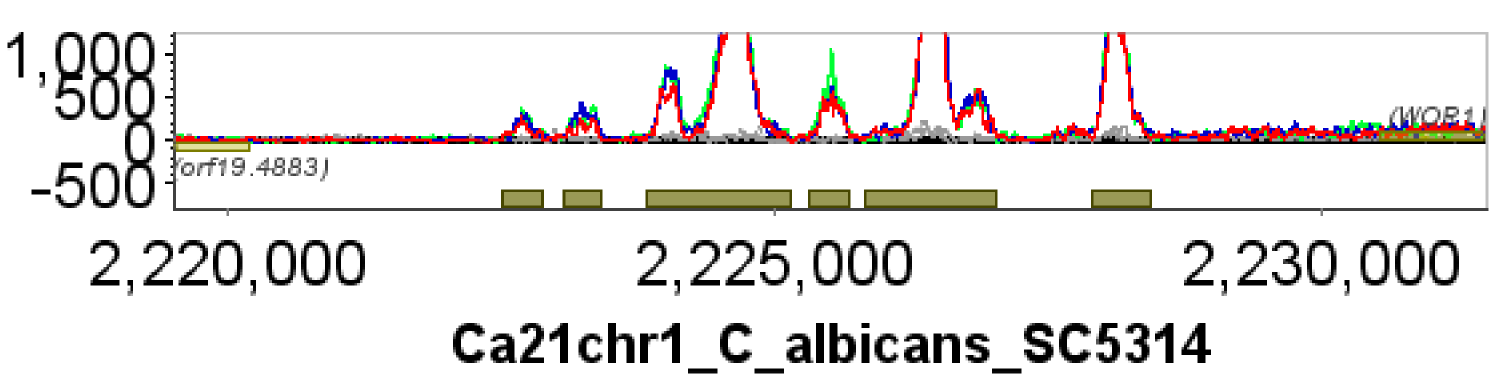

[11.89]   Ca21chr2_C_albicans_SC5314:1740357-1752356 [+]   [YWP1, GTR1, ERG9, orf19.3621]

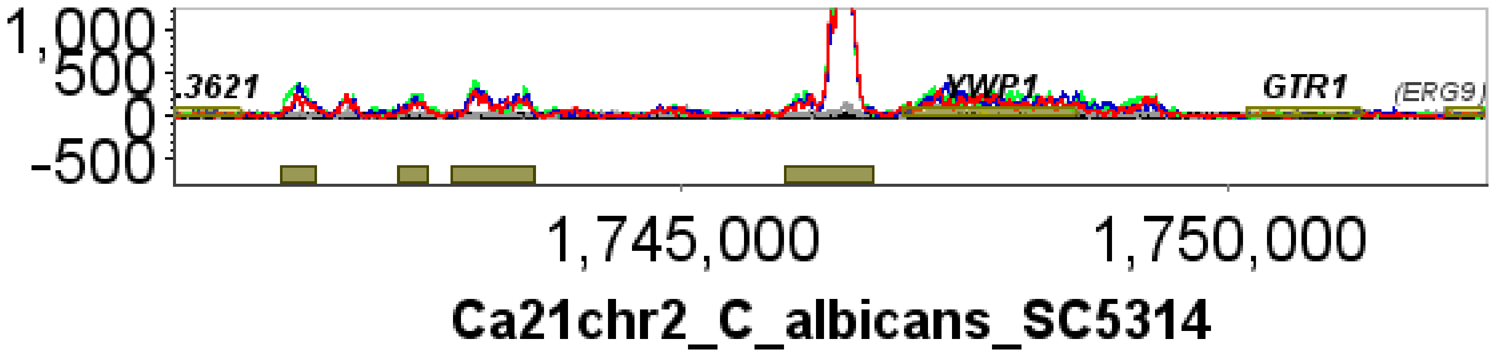

[11.75]   Ca21chrR_C_albicans_SC5314:29002-41001 [+]   [orf19.7522, REP1, MKC1, POT1, orf19.7519]

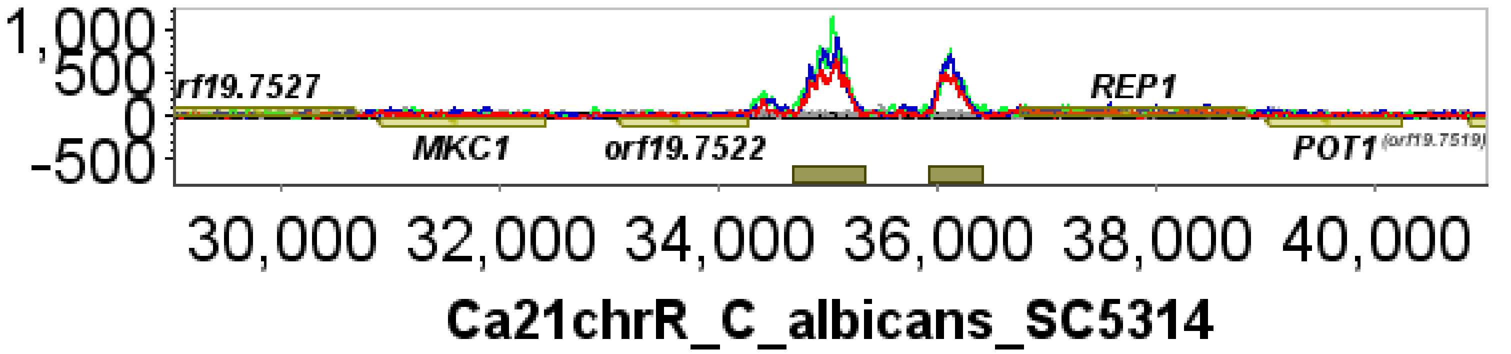

[11.7]   Ca21chr2_C_albicans_SC5314:729732-741731 [+]   [ADAEC, orf19.871]

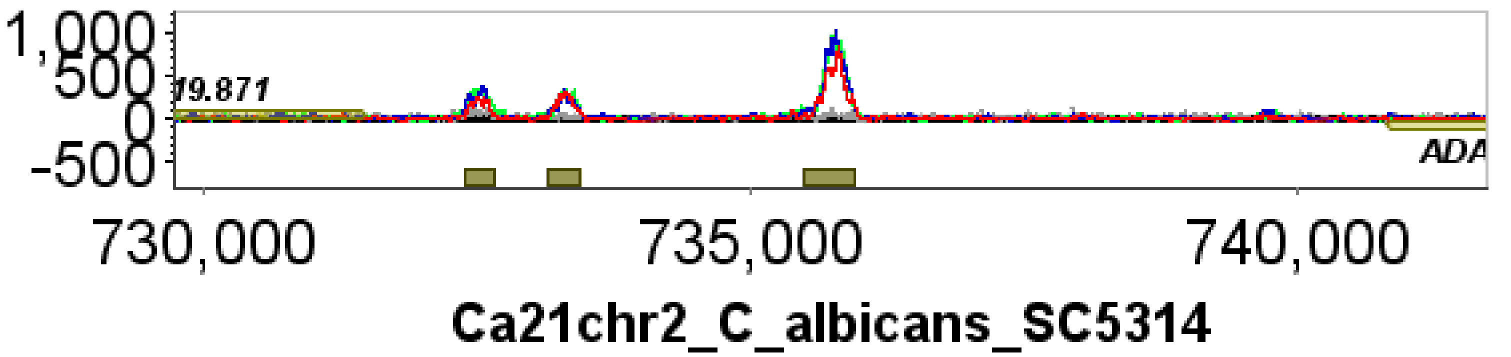

[11.66]   Ca21chr2_C_albicans_SC5314:738245-750244 [+]   [ADAEC, orf19.867, RAD32]

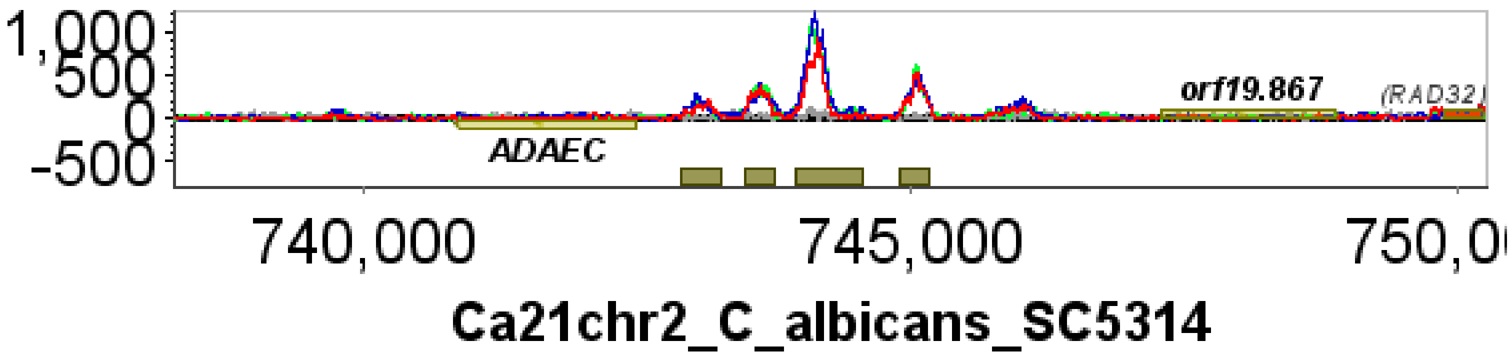

[11.63] Ca21chrR_C_albicans_SC5314:867038-879037 [+] [WOR3, IFF9]

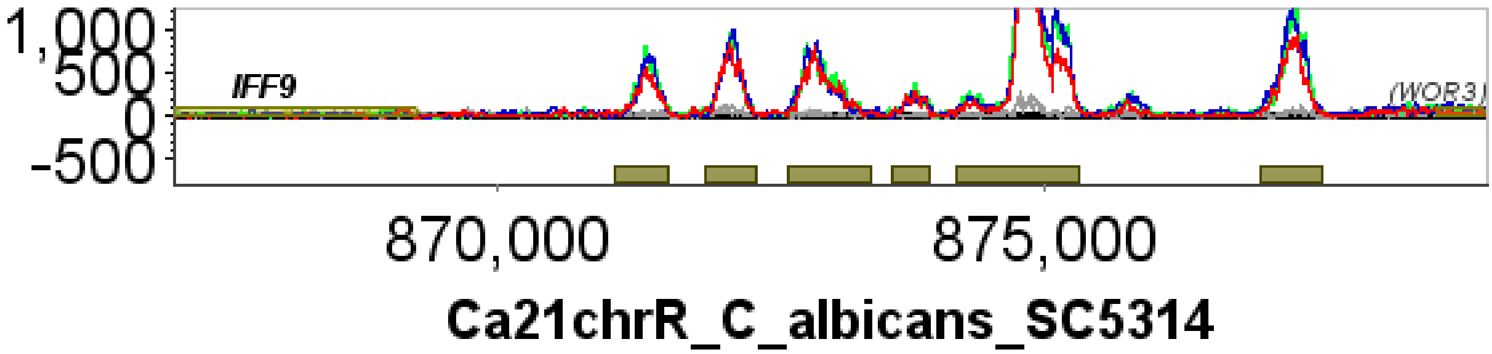

[11.6] Ca21chrR_C_albicans_SC5314:581093-593092 [+] [orf19.2822, orf19.2821, orf19.2820, orf19.2819, orf19.2818]

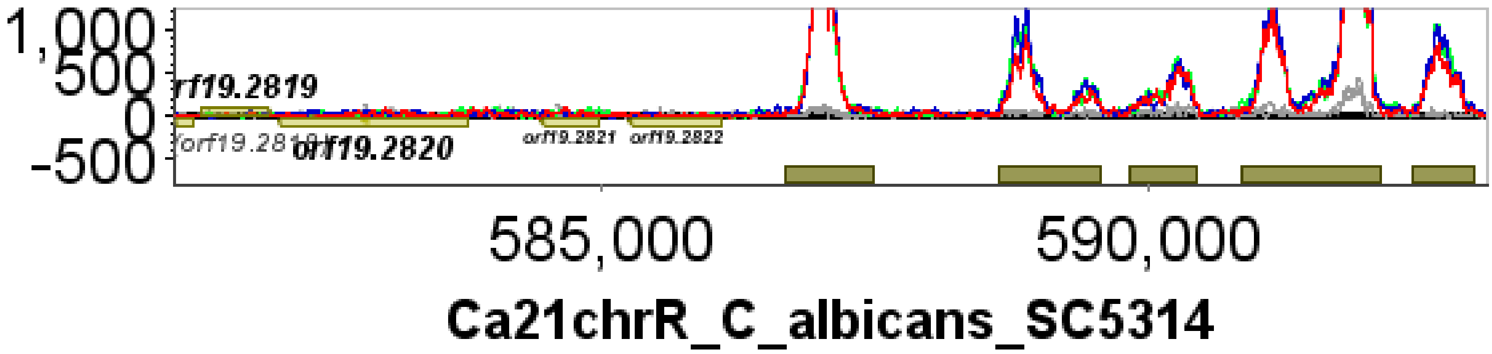

[11.56] Ca21chrR_C_albicans_SC5314:1520090-1532089 [+] [CRZ2, URA2]

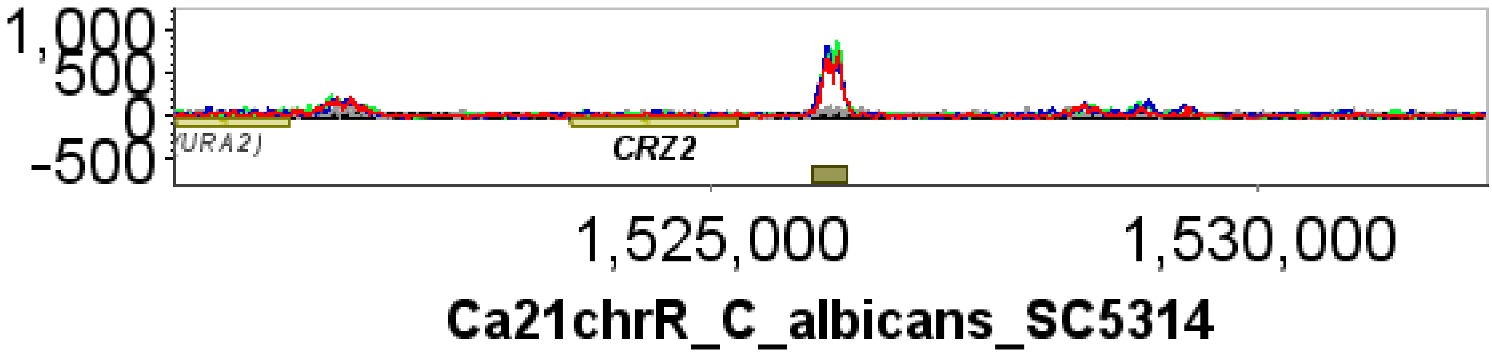

[11.49] Ca21chr7_C_albicans_SC5314:906330-918329 [+] [orf19.7151, orf19.7152, orf19.7153]

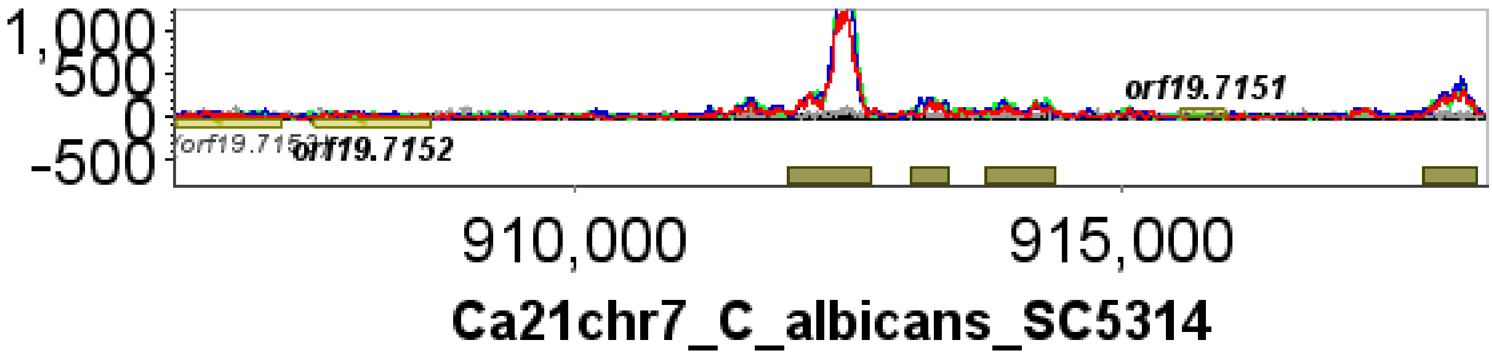

[11.44] Ca21chr1_C_albicans_SC5314:2341654-2353653 [+] [snR62, orf19.1831, FCY23, orf19.1830, orf19.1833]

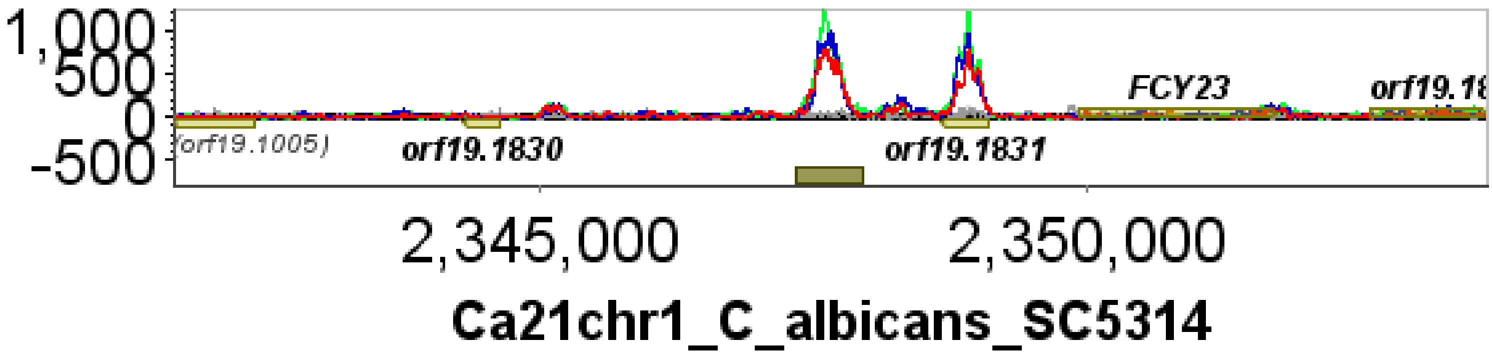

[11.14]   Ca21chr3_C_albicans_SC5314:1755025-1767024 [+]   [orf19.6713, orf19.6712, orf19.6715, UTP9, ABD1]

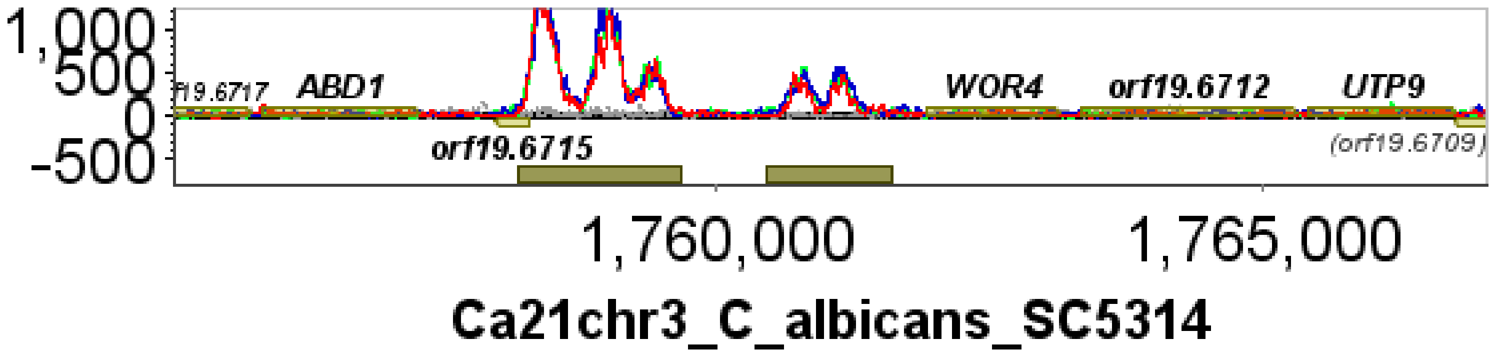

[10.72]   Ca21chrR_C_albicans_SC5314:30166-42165 [+]   [REP1, orf19.7522, MKC1, POT1, orf19.7519]

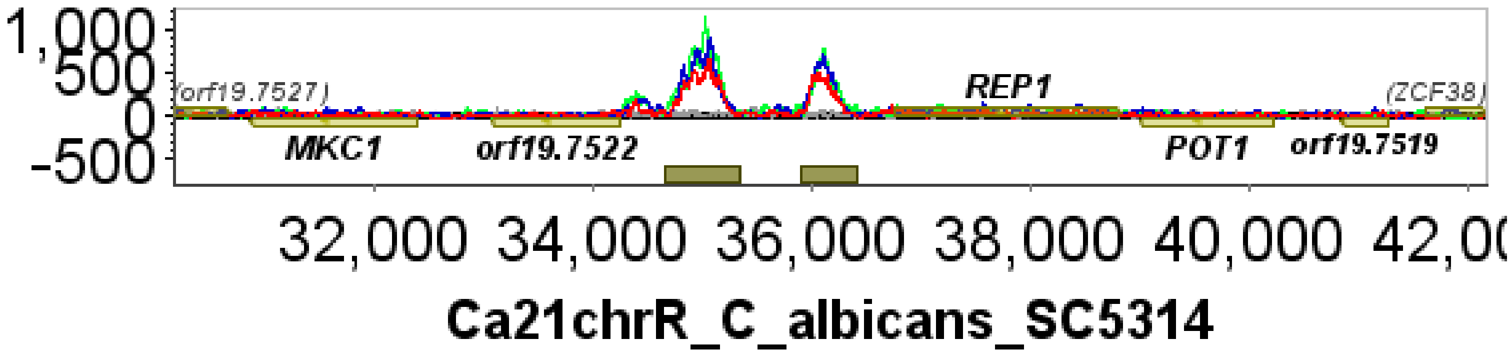

[10.7]   Ca21chr2_C_albicans_SC5314:1864817-1876816 [+]   [IFF6, MET10]

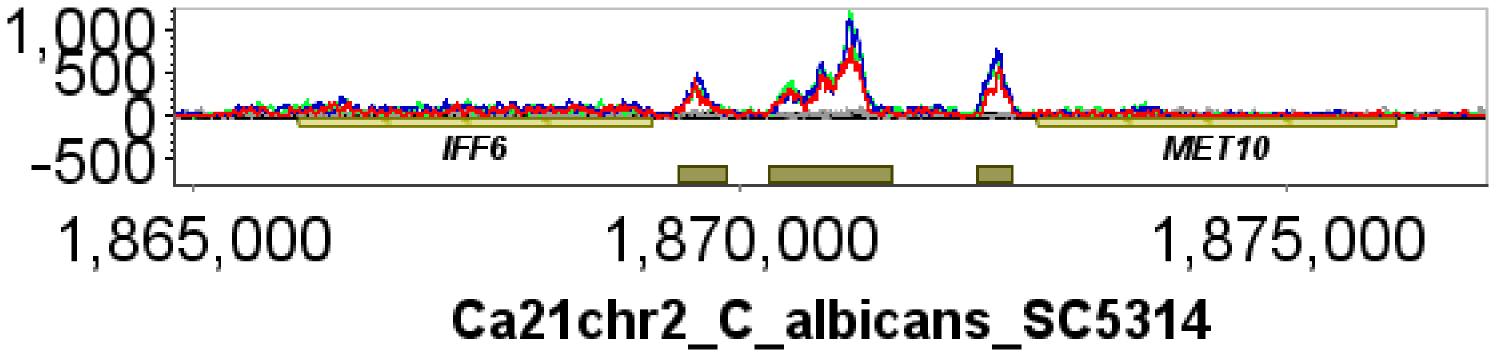

[10.69]   Ca21chr2_C_albicans_SC5314:779462-791461 [+]   [orf19.849, orf19.850, PGA16, tR(UCU)2, tF(GAA)2]

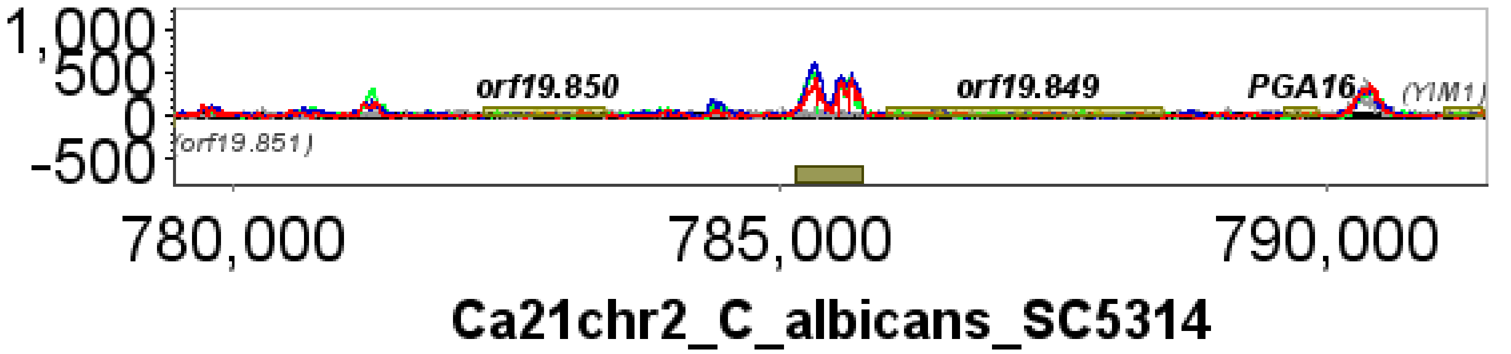

[10.64]   Ca21chr1_C_albicans_SC5314:141526-153525 [+]   [orf19.6027]

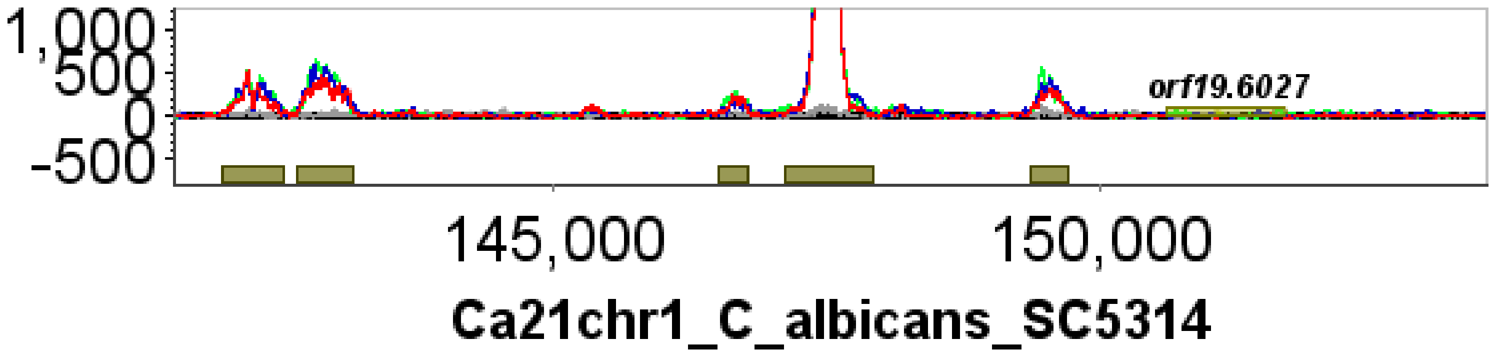

[10.38] Ca21chrR_C_albicans_SC5314:871252-883251 [+] [WOR3]

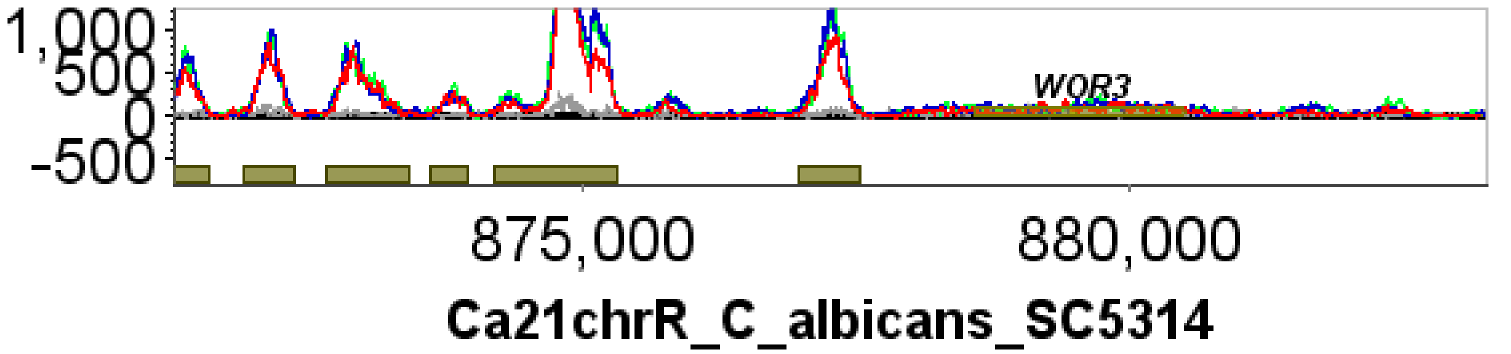

[10.36] Ca21chr5_C_albicans_SC5314:111904-123903 [+] [orf19.934, orf19.933]

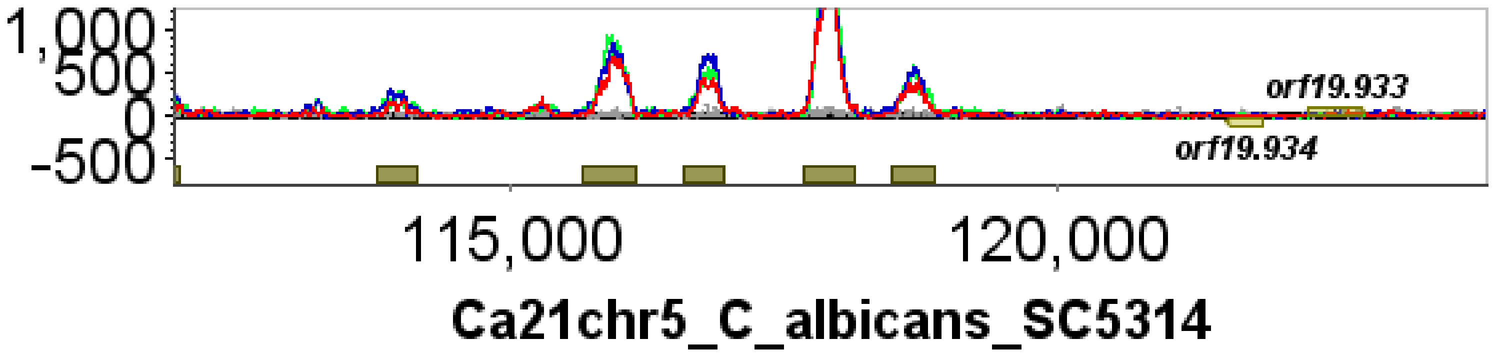

[10.31] Ca21chr3_C_albicans_SC5314:256778-268777 [+] [tR(CCU)1, orf19.1728, PMC1, orf19.1729, orf19.1730]

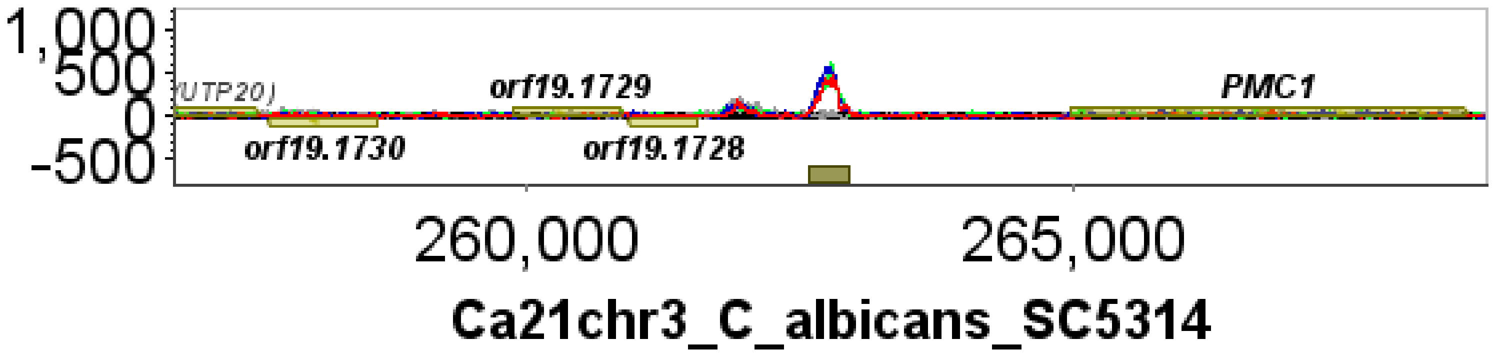

[10.3] Ca21chrR_C_albicans_SC5314:583107-595106 [+] [orf19.2822, orf19.2821, orf19.2820]

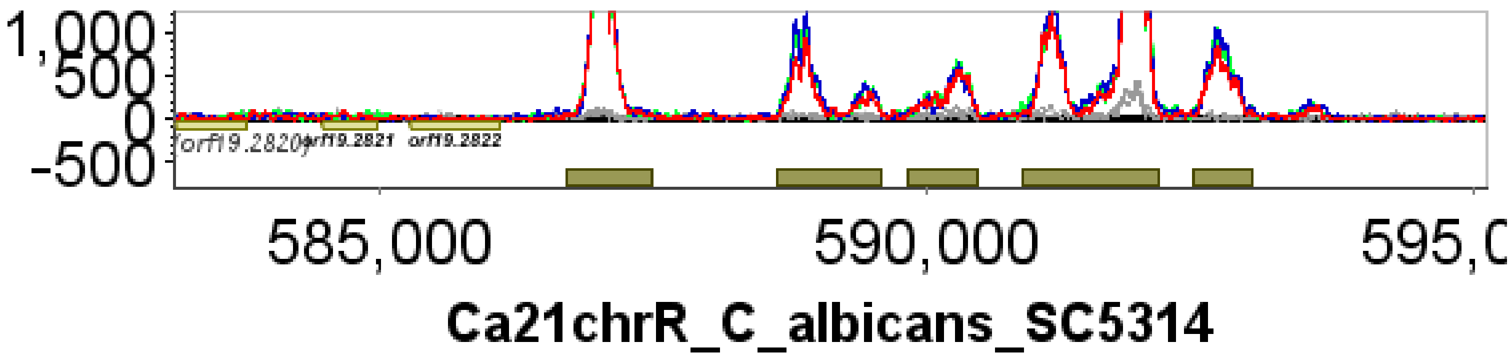

[10.18] Ca21chr1_C_albicans_SC5314:136914-148913 [+] [HGC1]

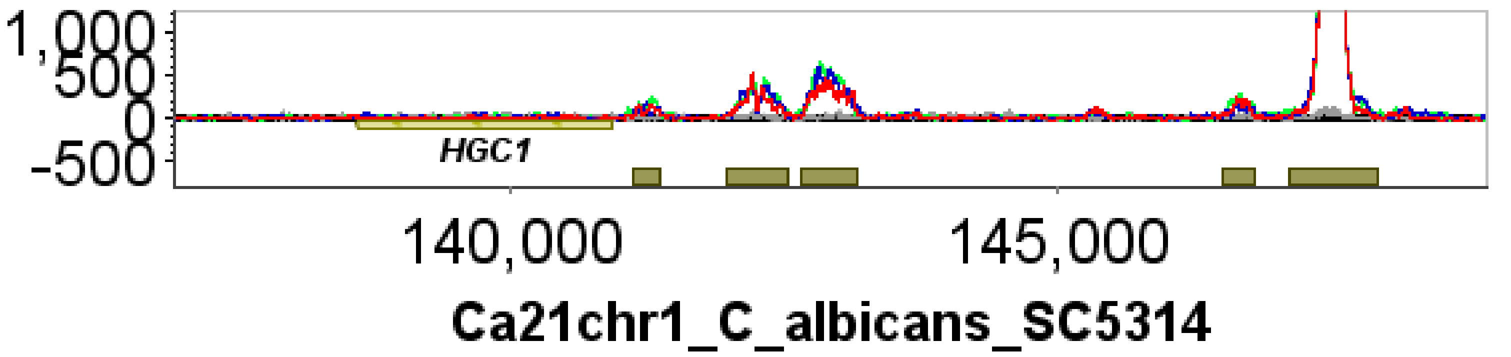

[10.06] Ca21chr1_C_albicans_SC5314:1516541-1528540 [+] [TAF145, GAP4, orf19.6194]

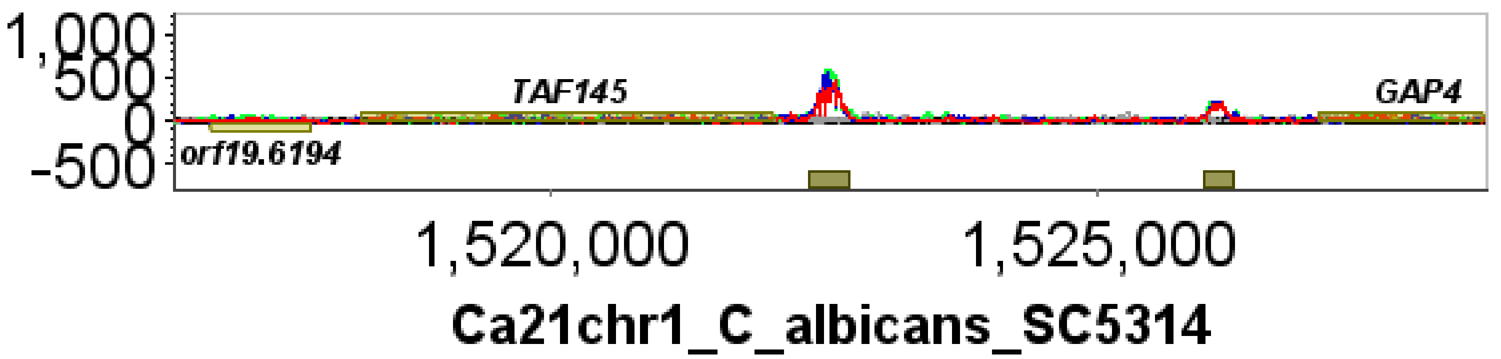

[10.04] Ca21chr5_C_albicans_SC5314:490226-502225 [+] [PTH2, orf19.4230, orf19.4229, orf19.4228, PTH1]

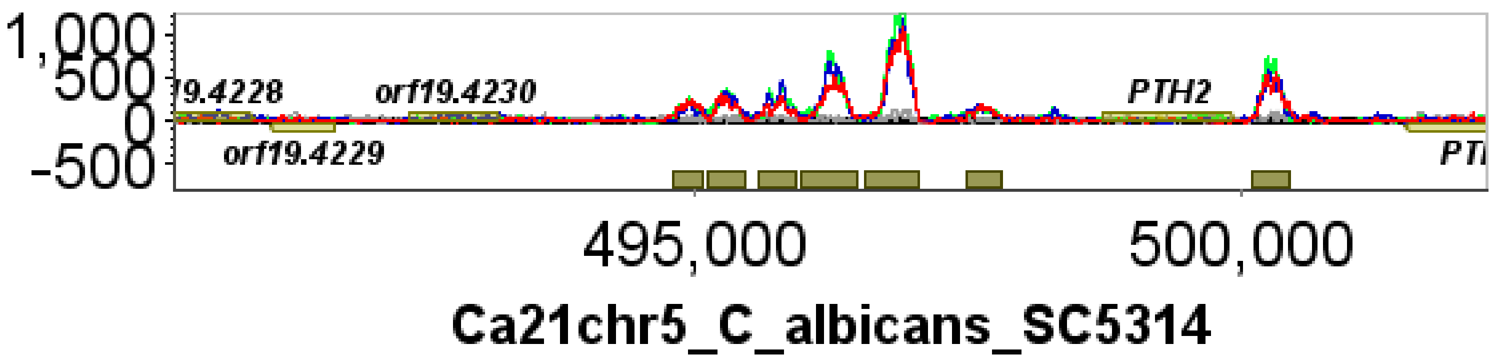

[10.02] Ca21chr1_C_albicans_SC5314:1329475-1341474 [+] [snR44a, snR5b, orf19.177, orf19.6280]

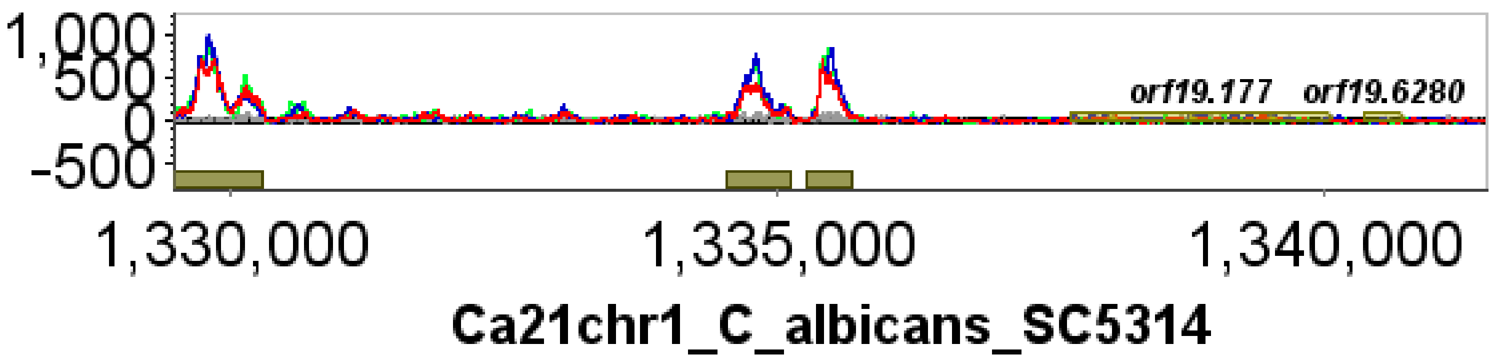

[9.83] Ca21chr3_C_albicans_SC5314:1723172-1735171 [+] [TCC1, orf19.6732, orf19.6731.1, orf19.6731, orf19.6730]

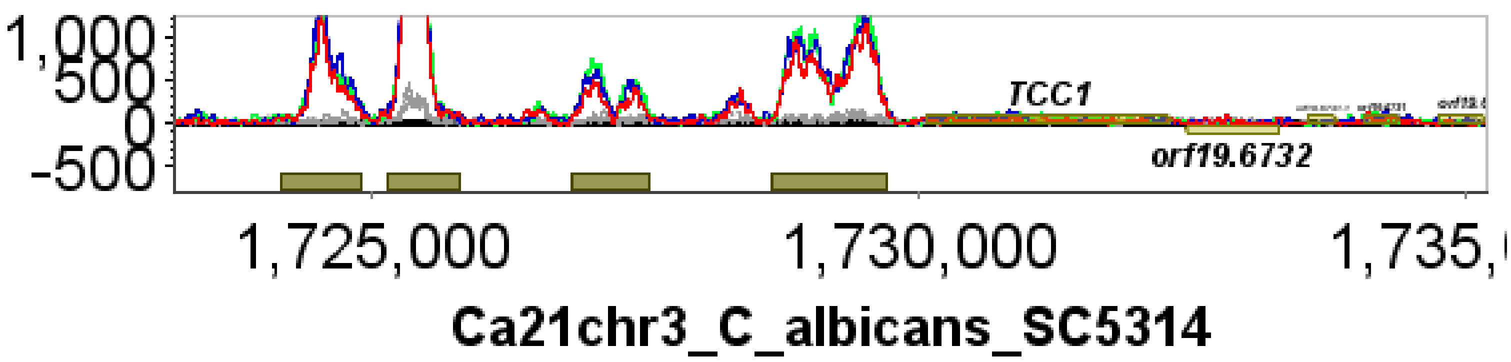

[9.82] Ca21chr1_C_albicans_SC5314:442096-454095 [+] [GAL1, orf19.3671, SHA3, GAL10]

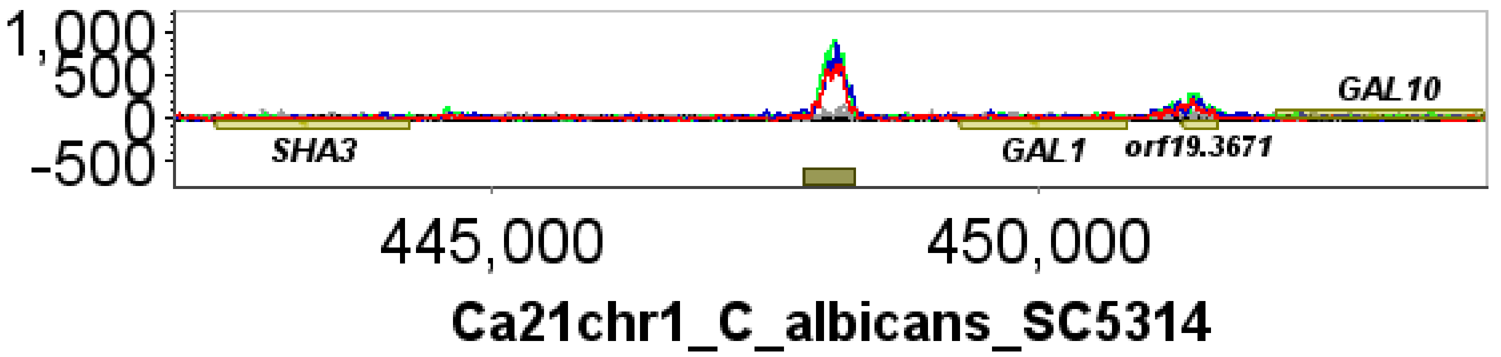

[9.79] Ca21chrR_C_albicans_SC5314:1713717-1725716 [+] [EFG1, tG(GCC)1, orf19.609]

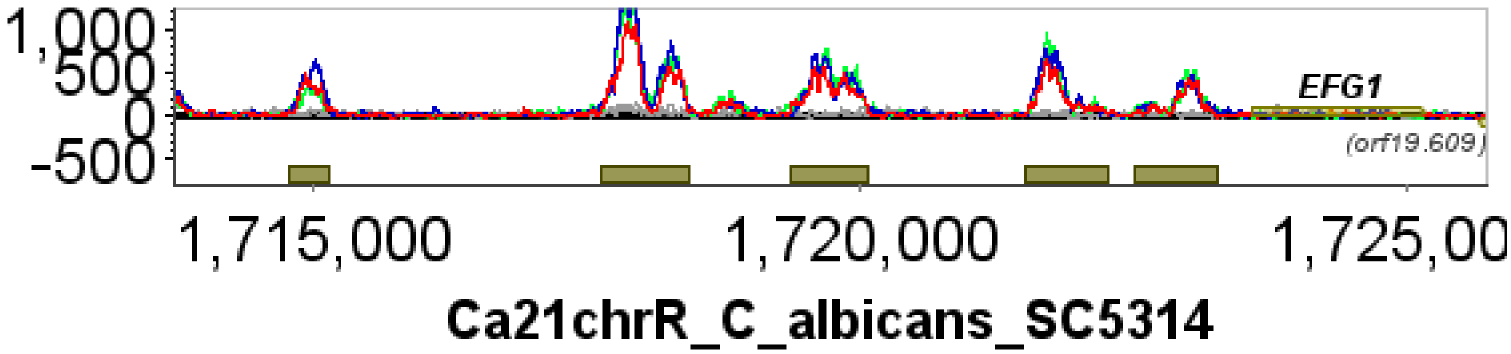

[9.78] Ca21chr6_C_albicans_SC5314:149554-161553 [+] [SUN41, orf19.3643, CAN3]

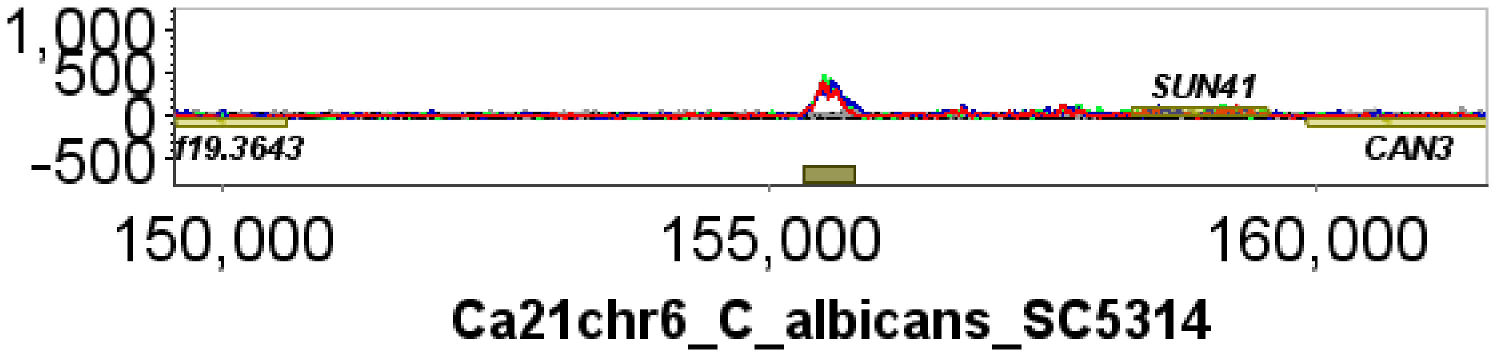

[9.78] Ca21chr3_C_albicans_SC5314:1111980-1123979 [+] [WOR2]

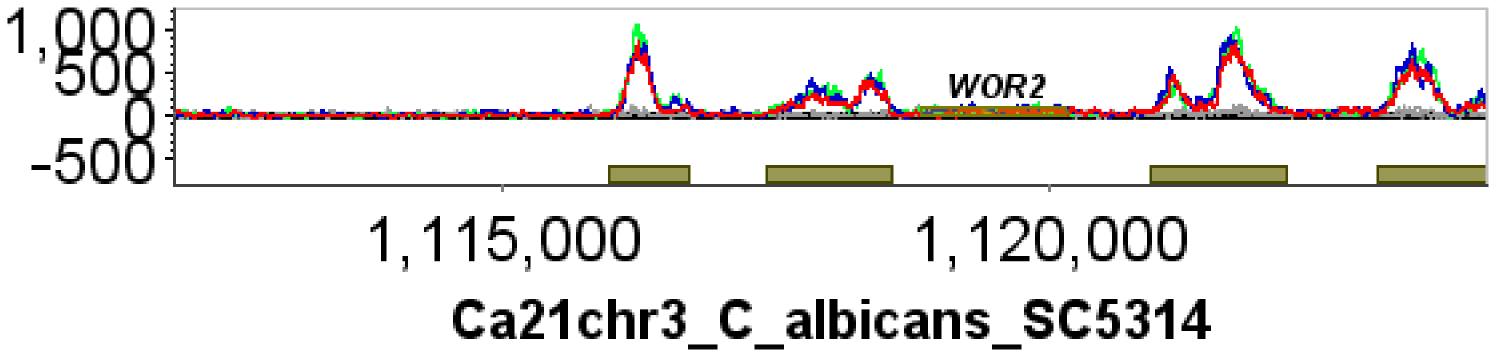

[9.77] Ca21chr1_C_albicans_SC5314:1328829-1340828 [+] [snR44a, snR5b, orf19.177, orf19.6280]

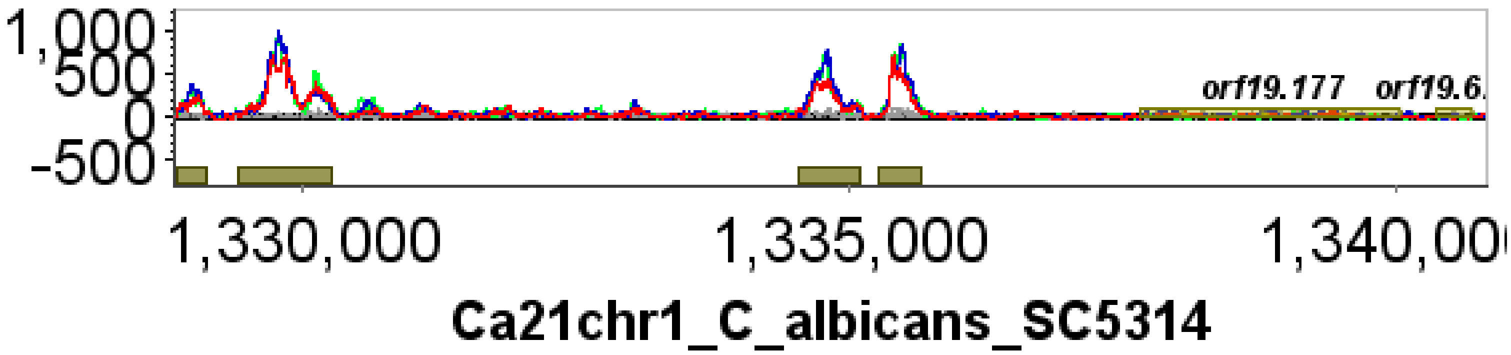

[9.76] Ca21chr1_C_albicans_SC5314:1076208-1088207 [+] [BRG1, orf19.450]

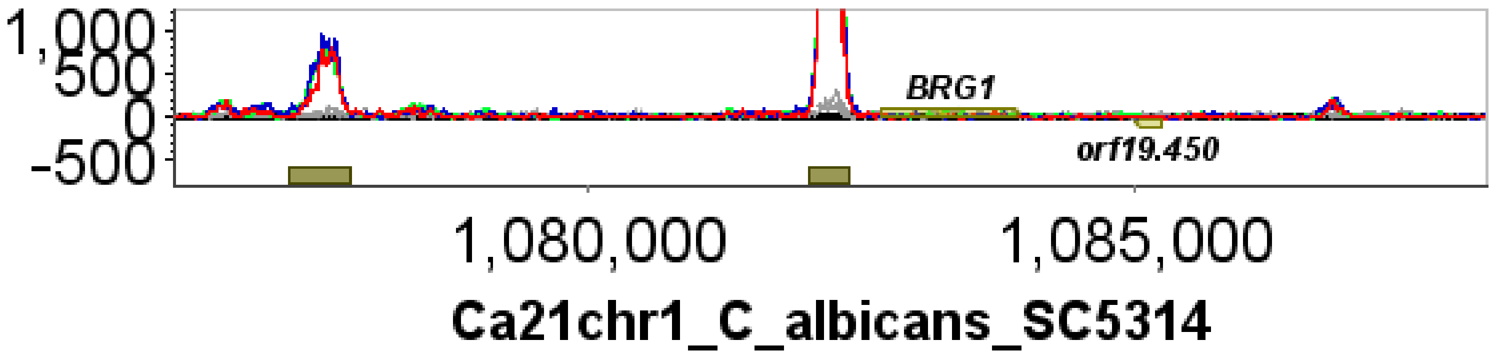

[9.73]   Ca21chr1_C_albicans_SC5314:2220439-2232438 [+]   [WOR1]

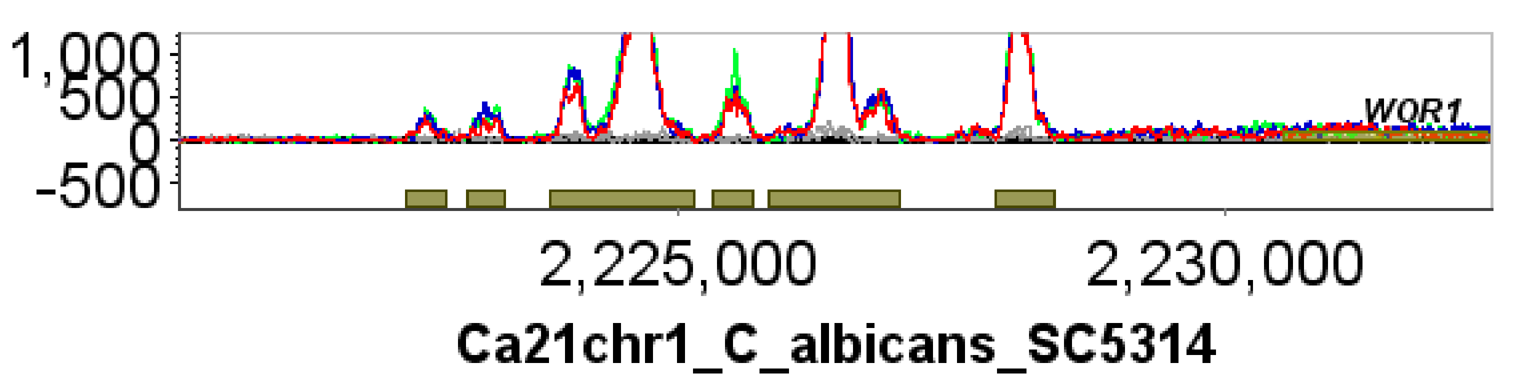

[9.71]   Ca21chrR_C_albicans_SC5314:1309672-1321671 [+]   [orf19.3868, orf19.3869, ADE13, DAD3]

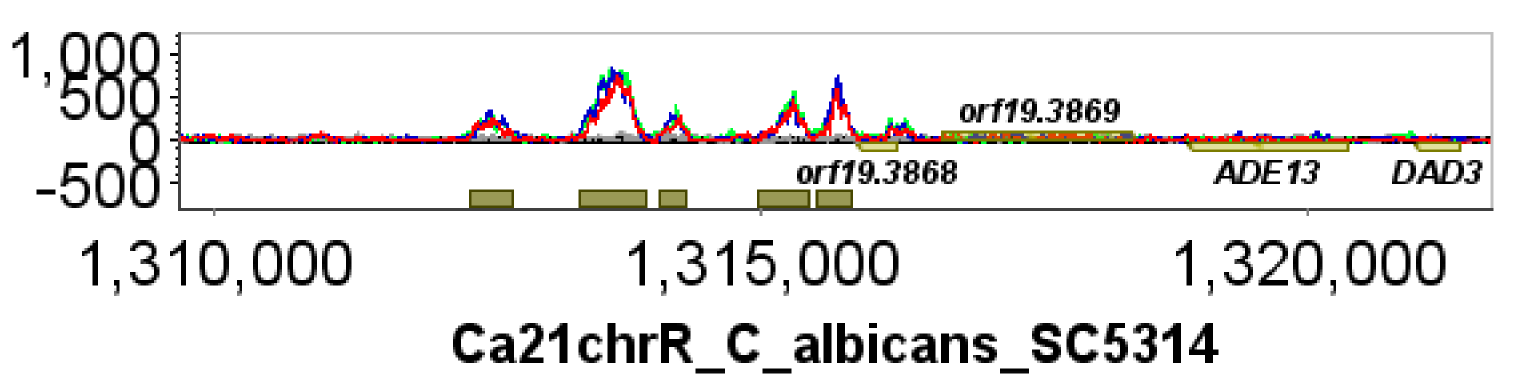

[9.68]   Ca21chr2_C_albicans_SC5314:739018-751017 [+]   [orf19.867, ADAEC, RAD32]

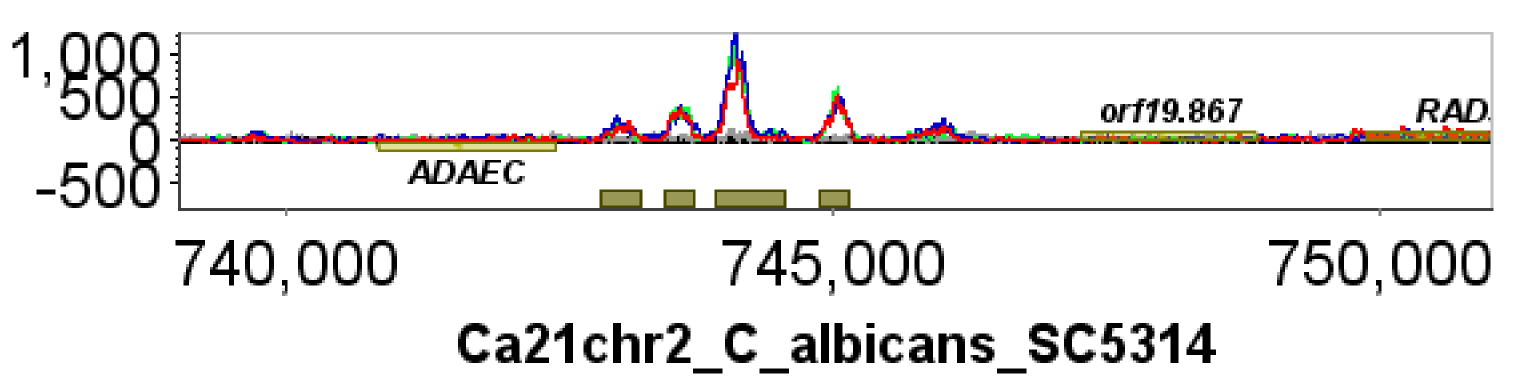

[9.64]   Ca21chrR_C_albicans_SC5314:2078812-2090811 [+]   [IFA14, ALO1]

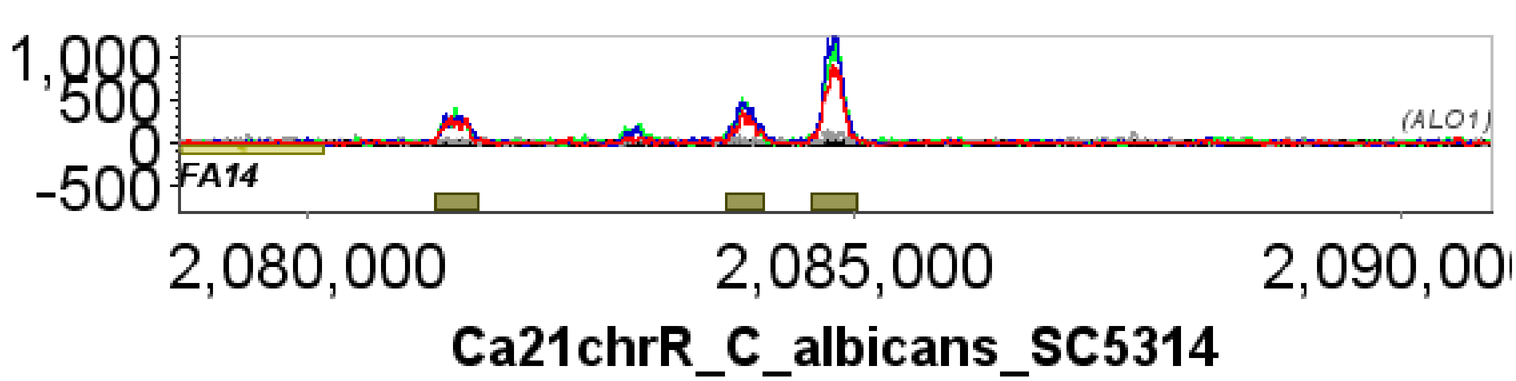

[9.54]   Ca21chrR_C_albicans_SC5314:592669-604668 [+]   [RFG1]

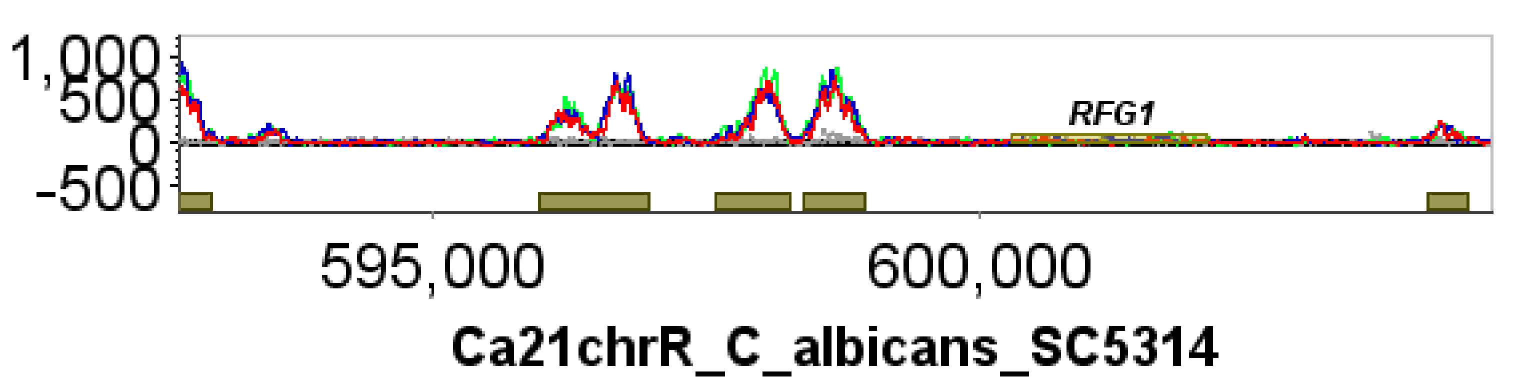

[9.52] Ca21chrR_C_albicans_SC5314:866133-878132 [+] [IFF9]

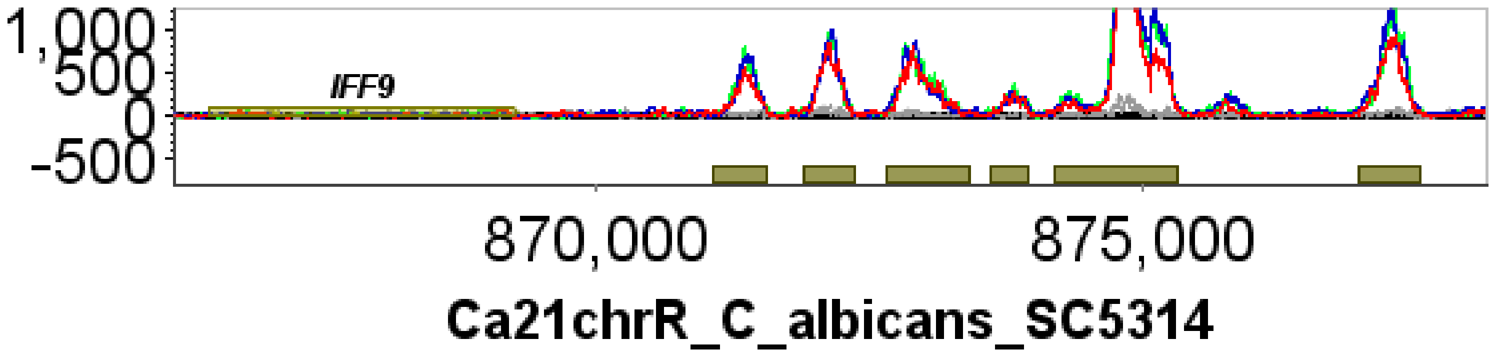

[9.49] Ca21chr7_C_albicans_SC5314:108927-120926 [+] [orf19.7054, GAC1]

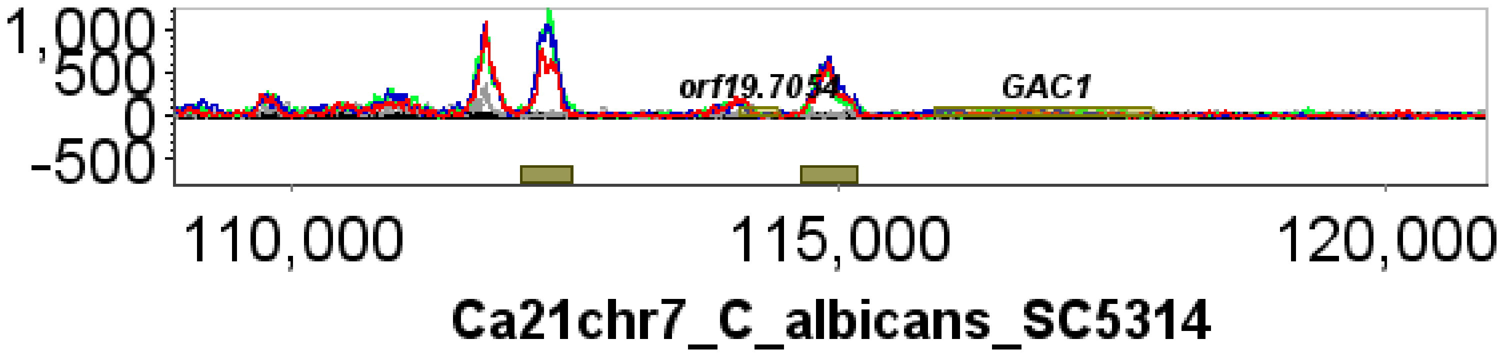

[9.47] Ca21chr5_C_albicans_SC5314:490793-502792 [+] [PTH2, orf19.4230, orf19.4229, PTH1, orf19.4228]

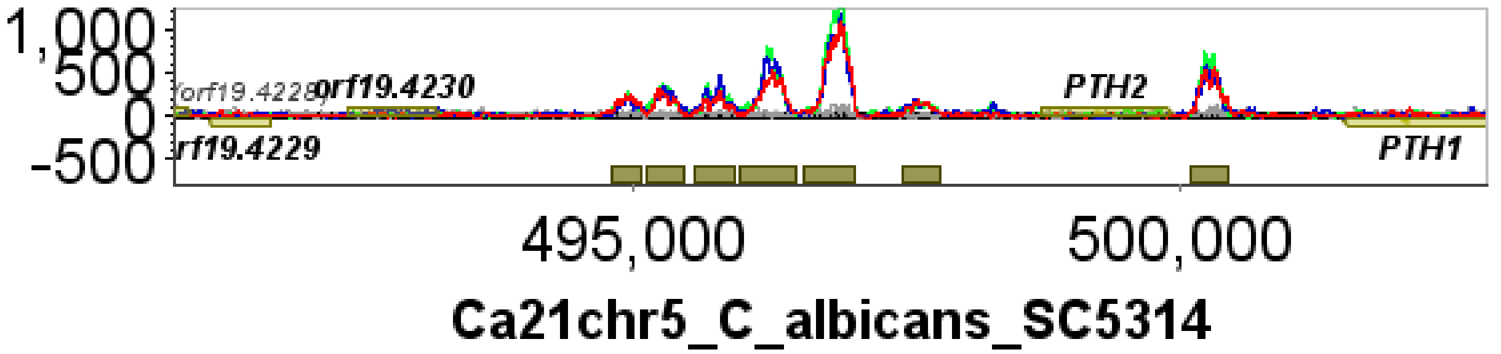

[9.42] Ca21chr5_C_albicans_SC5314:109881-121880 [+] [AGA1, orf19.934]

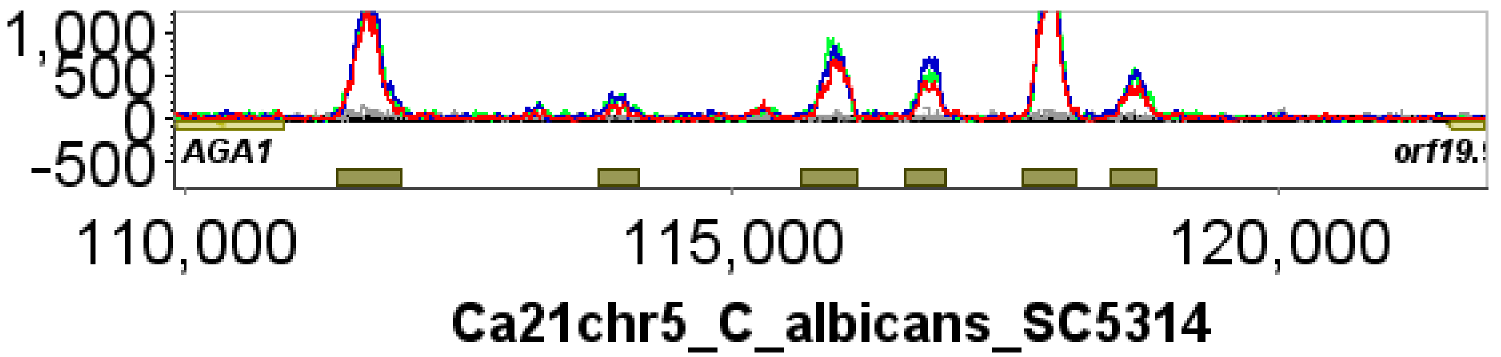

[9.4] Ca21chr3_C_albicans_SC5314:1582785-1594784 [+] [orf19.6805, orf19.6807, orf19.6809, orf19.6810]

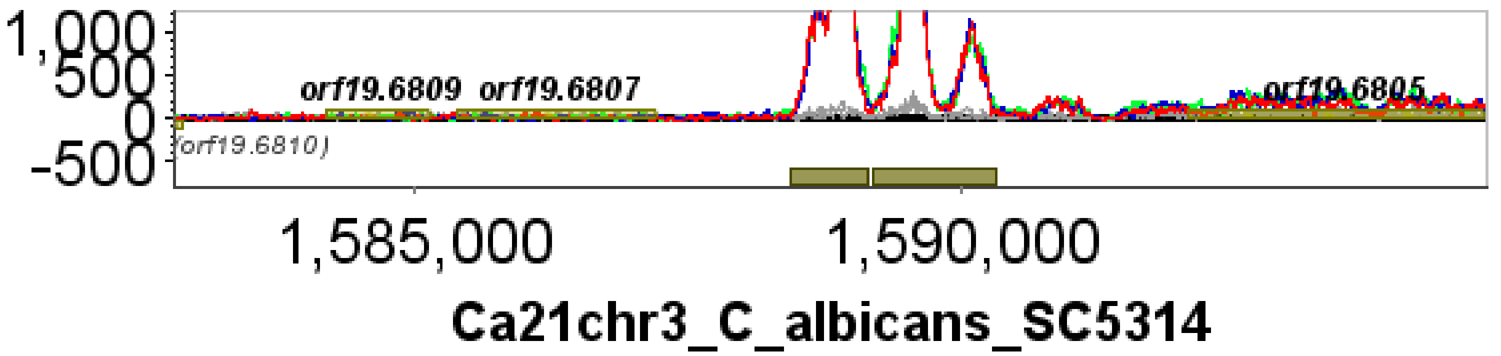

[9.35] Ca21chr7_C_albicans_SC5314:436586-448585 [+] [HSP90, RAD14, orf19.6518]

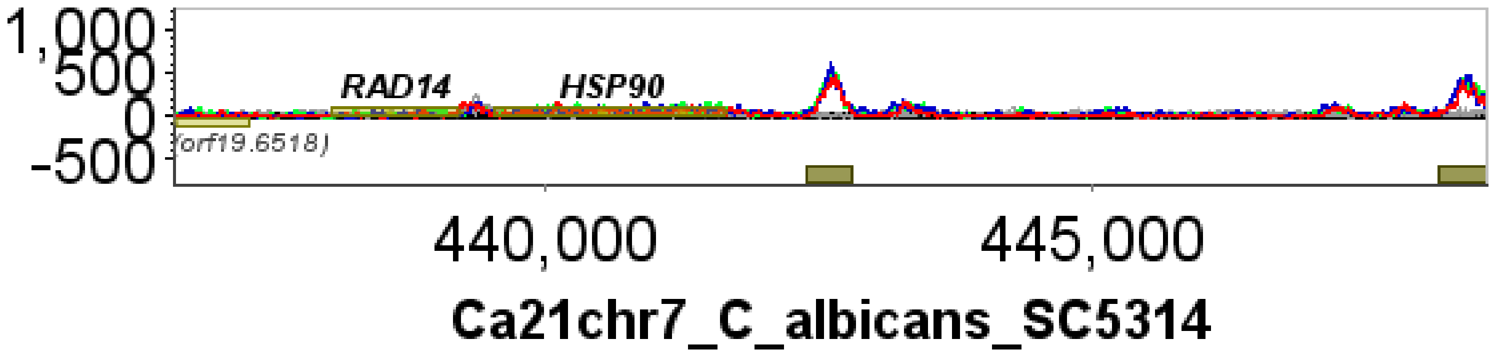

[9.24] Ca21chr3_C_albicans_SC5314:1115546-1127545 [+] [WOR2, RHB1]

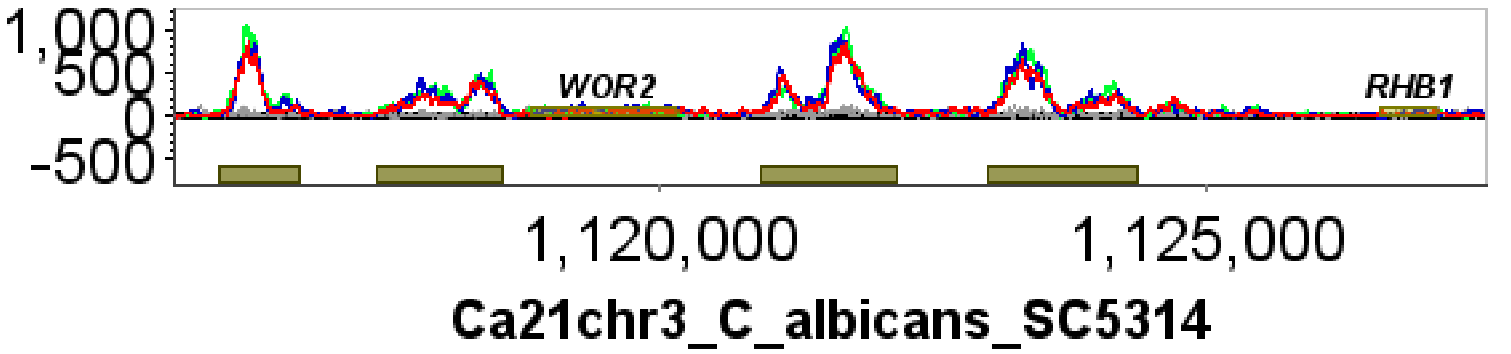

[9.2] Ca21chrR_C_albicans_SC5314:1708971-1720970 [+] [tG(GCC)1, orf19.612, ASF1]

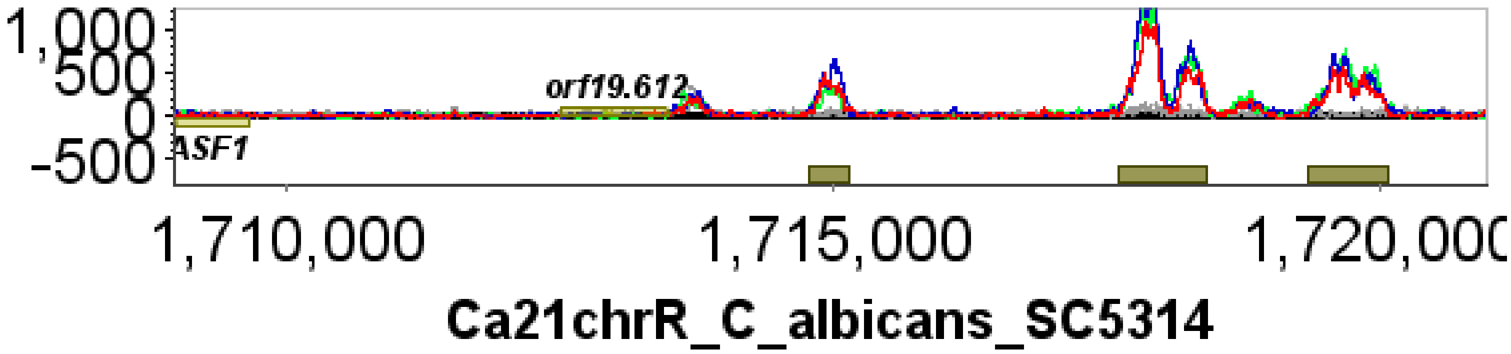

[9.12] Ca21chrR_C_albicans_SC5314:2105074-2117073 [+] [BET2, DEF1, DPB2]

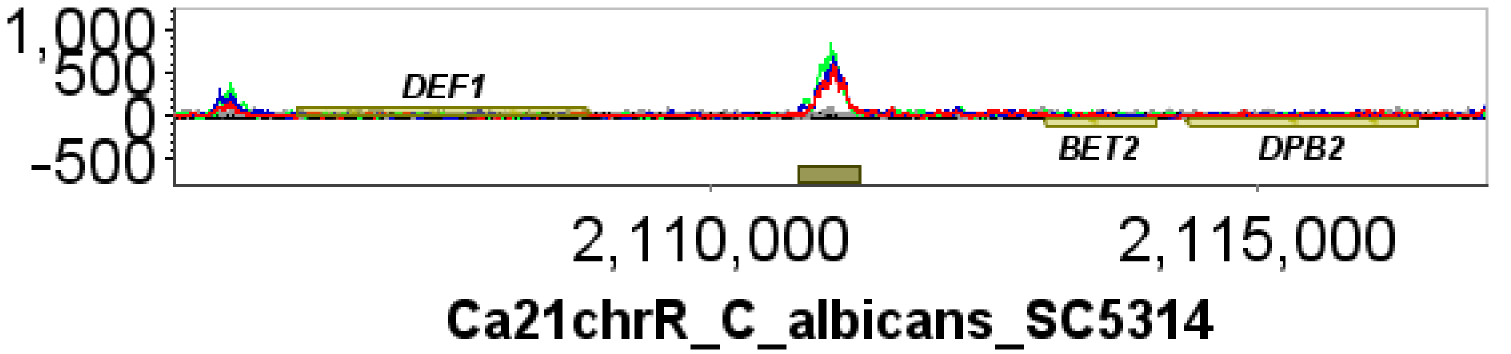

[9.12] Ca21chr3_C_albicans_SC5314:1583746-1595745 [+] [orf19.6805, orf19.6807, tY(GUA)3, orf19.6809]

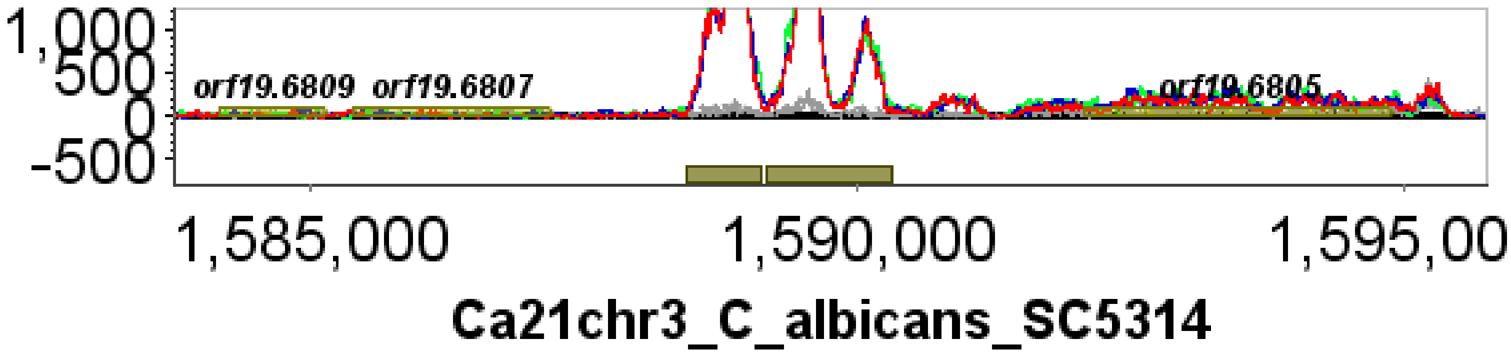

[9.01] Ca21chrR_C_albicans_SC5314:865320-877319 [+] [IFF9]

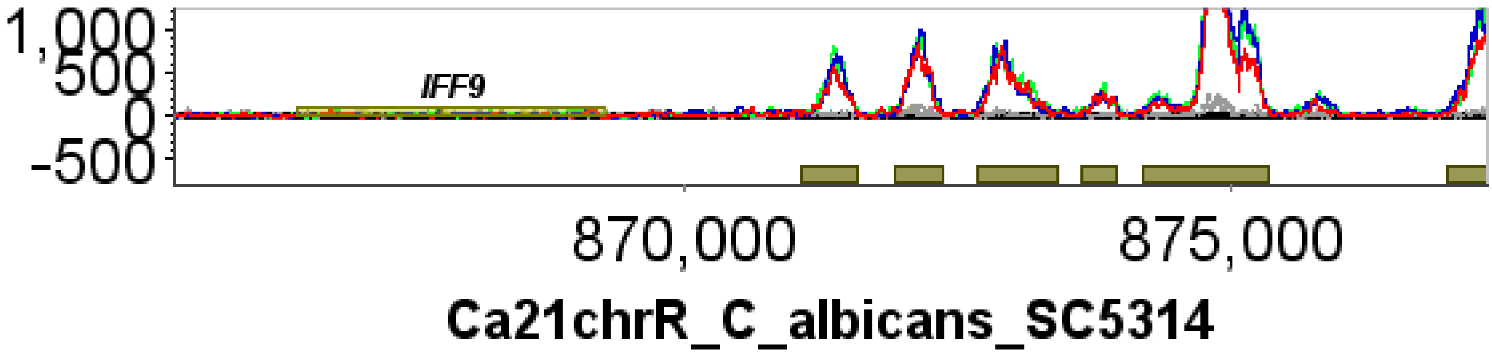

[8.94] Ca21chr6_C_albicans_SC5314:30982-42981 [+] [orf19.1189, CPH2]

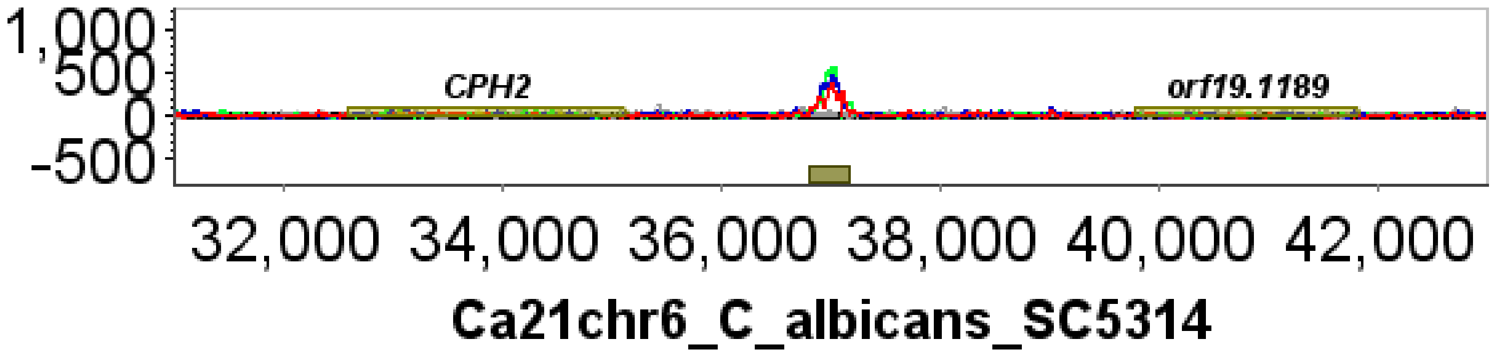

[8.93] Ca21chr5_C_albicans_SC5314:112664-124663 [+] [orf19.934, orf19.933]

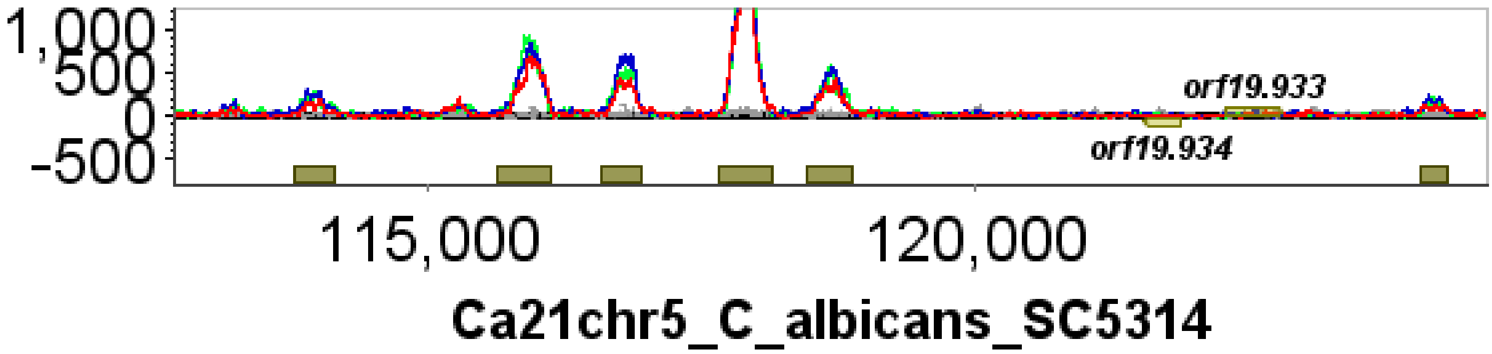

[8.88] Ca21chr3_C_albicans_SC5314:1716759-1728758 [+] [orf19.6736, orf19.6737]

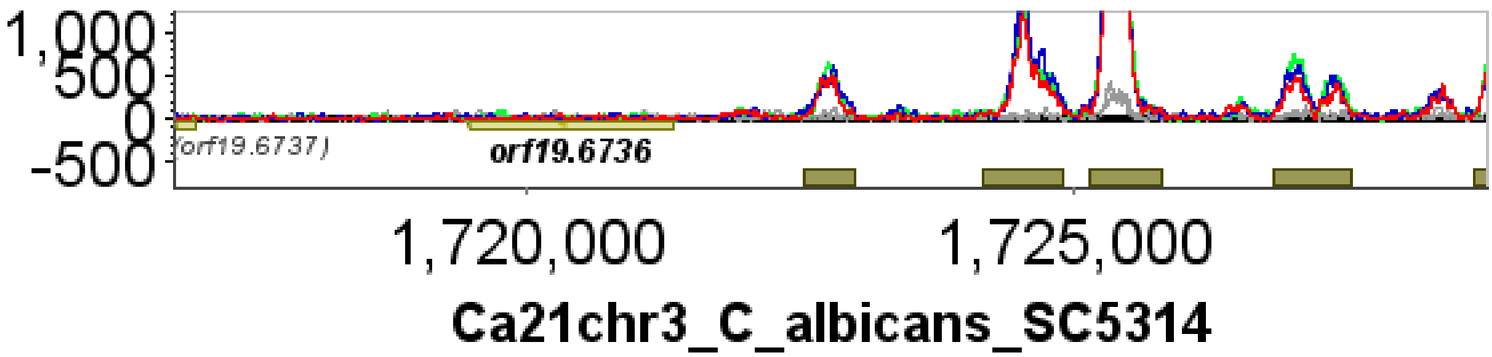

[8.8] Ca21chr5_C_albicans_SC5314:105674-117673 [+] [AGA1, orf19.936]

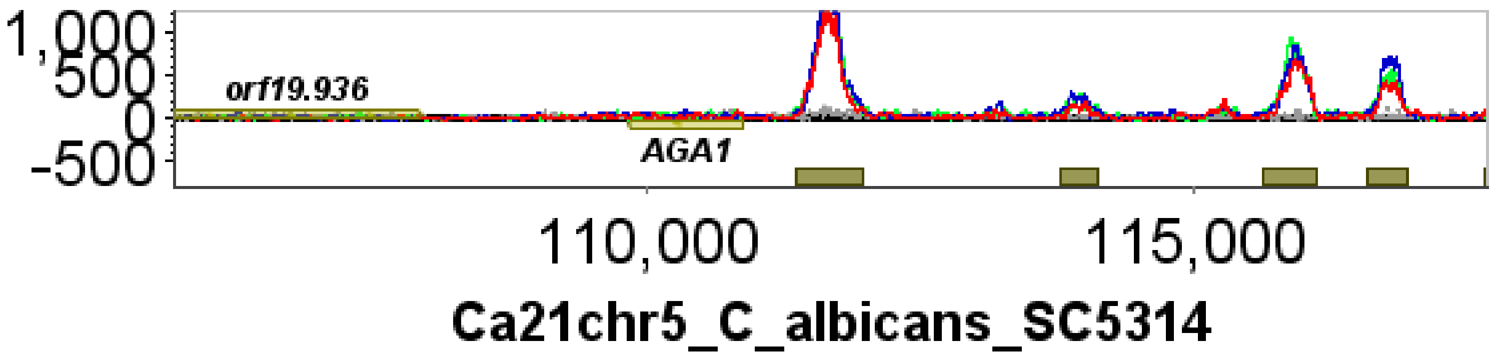

[8.75] Ca21chr5_C_albicans_SC5314:891056-903055 [+] [orf19.1286, orf19.1285, orf19.1287, MEC1]

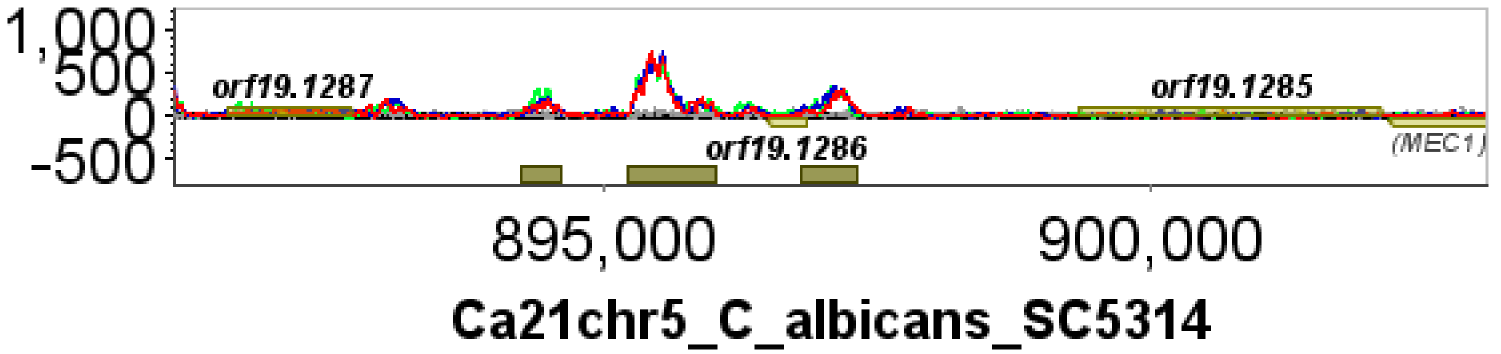

[8.61] Ca21chr1_C_albicans_SC5314:3104168-3116167 [+] [RBE1, RPL4B, FTR1, YPT52, orf19.7215.3]

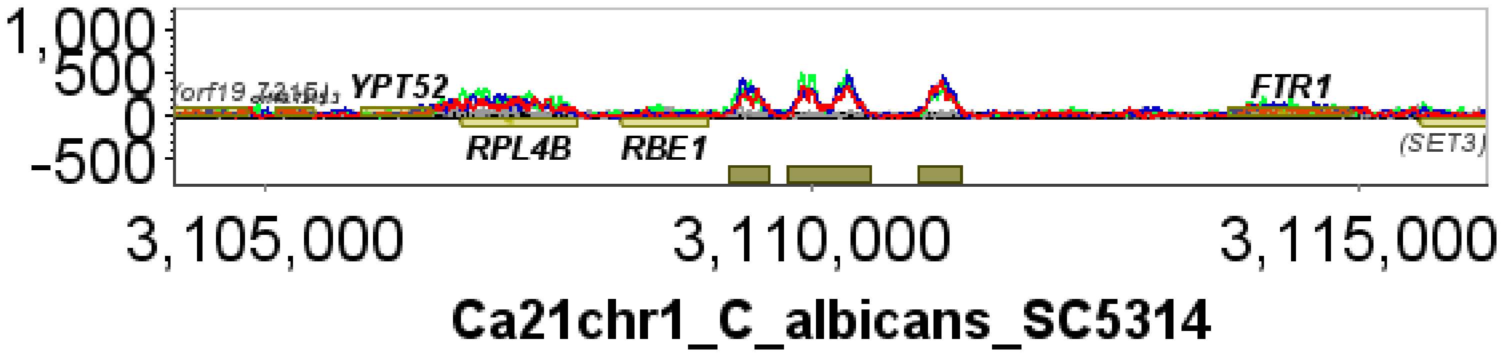

[8.57] Ca21chr7_C_albicans_SC5314:358970-370969 [+] [orf19.6556, orf19.6555, orf19.6554, orf19.6557]

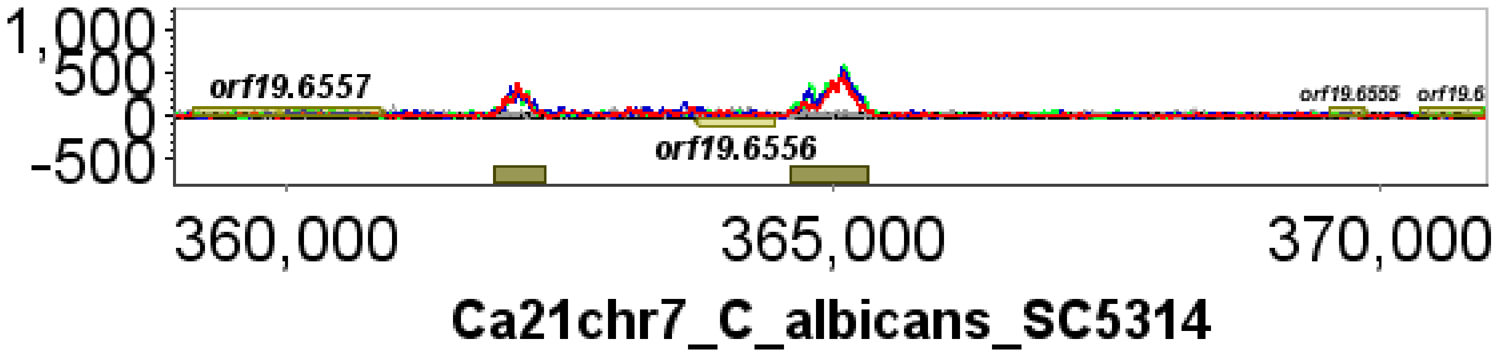

[8.53] Ca21chr1_C_albicans_SC5314:2222181-2234180 [+] [WOR1]

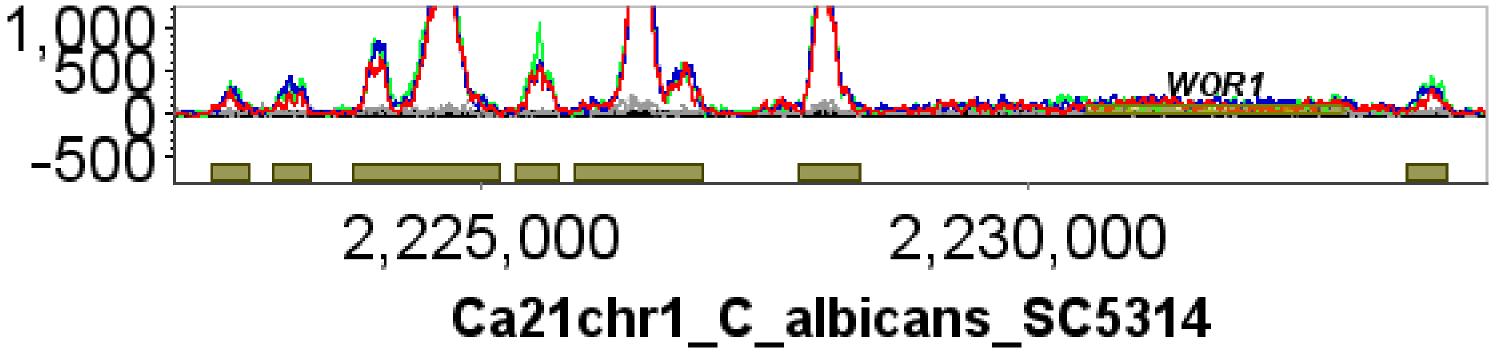

[8.51] Ca21chrR_C_albicans_SC5314:259354-271353 [+] [CTF18, orf19.3238, orf19.3237, ERG27]

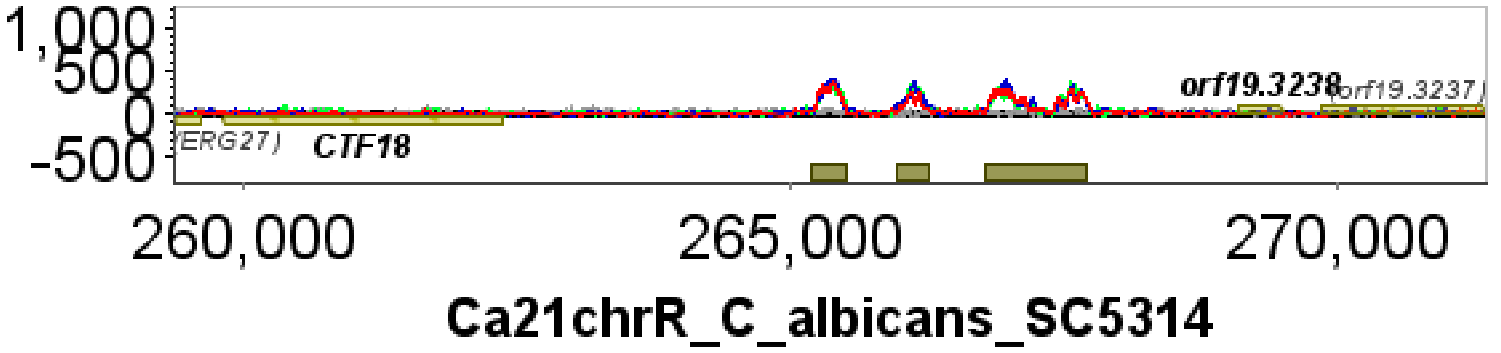

[8.51] Ca21chr1_C_albicans_SC5314:450215-462214 [+] [GAL102, GAL7, orf19.3673, ABP140, orf19.3677]

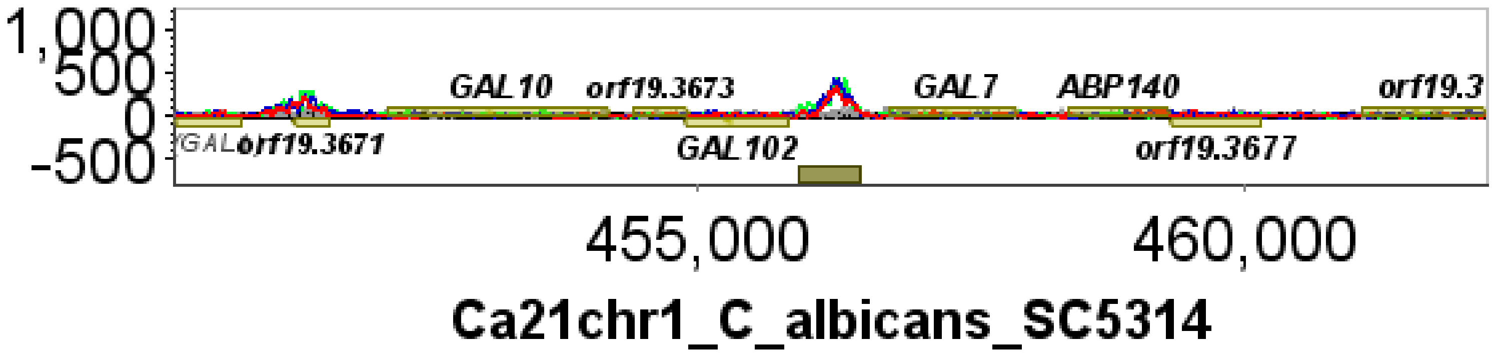

[8.47] Ca21chrR_C_albicans_SC5314:261246-273245 [+] [orf19.3238, orf19.3237, CTF18]

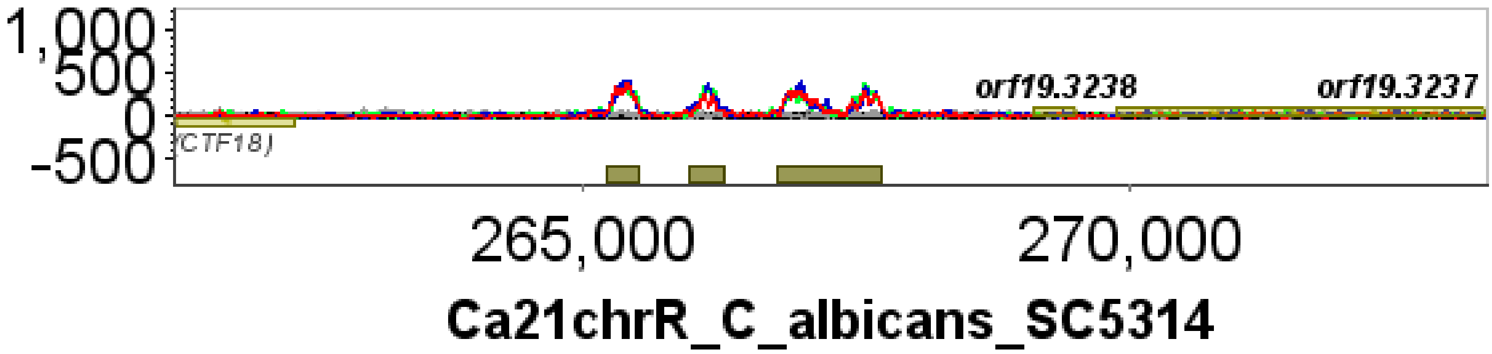

[8.45] Ca21chr1_C_albicans_SC5314:136251-148250 [+] [HGC1, ROT1]

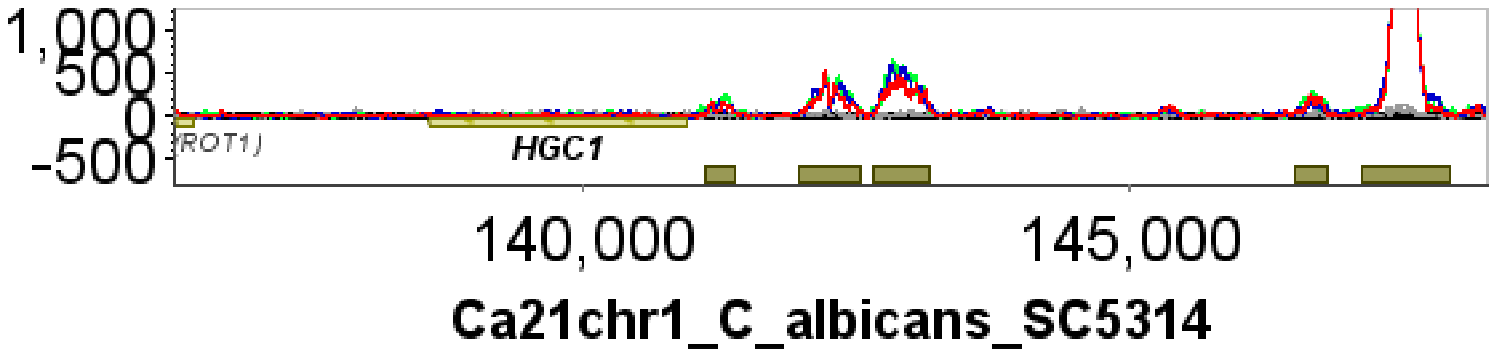

[8.35] Ca21chr5_C_albicans_SC5314:494266-506265 [+] [PTH2, PTH1, THR4, orf19.4234]

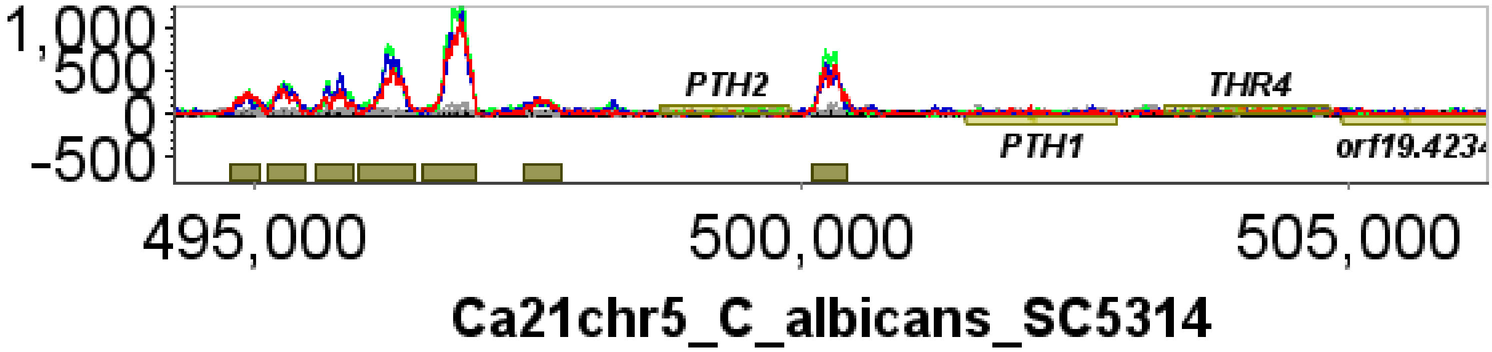

[8.31] Ca21chr5_C_albicans_SC5314:241227-253226 [+] [CLN3]

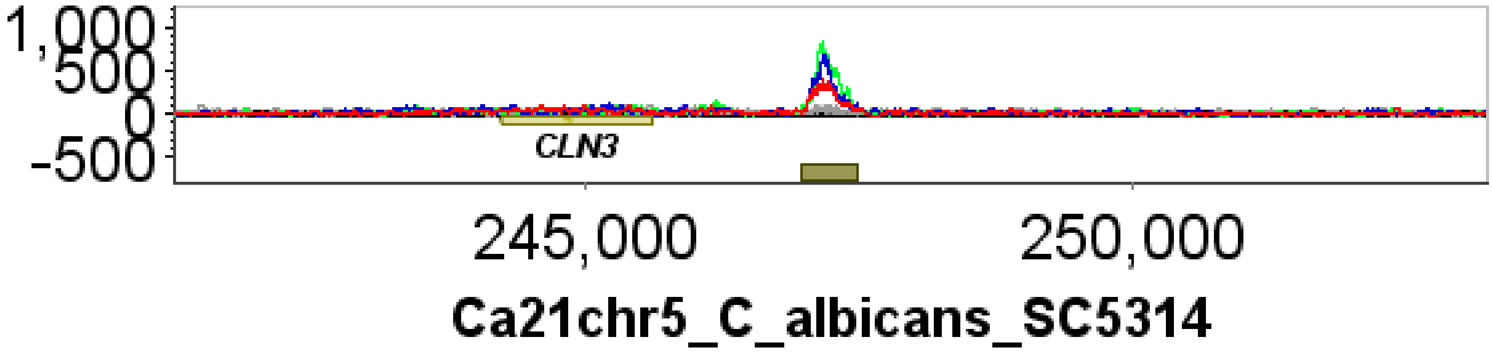

[8.26] Ca21chr3_C_albicans_SC5314:1117678-1129677 [+] [RHB1, WOR2, MCA1]

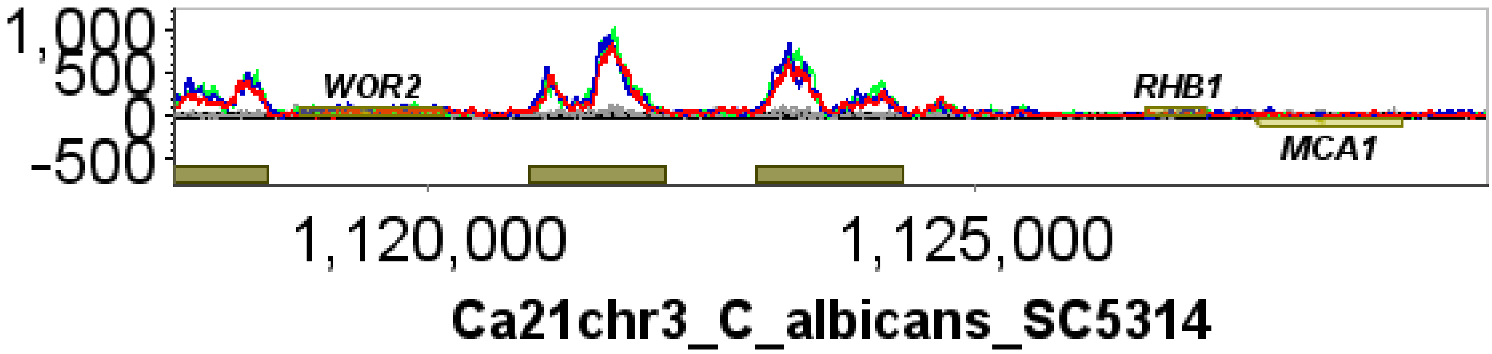

[8.13] Ca21chr4_C_albicans_SC5314:564213-576212 [+] [orf19.2724, orf19.2725]

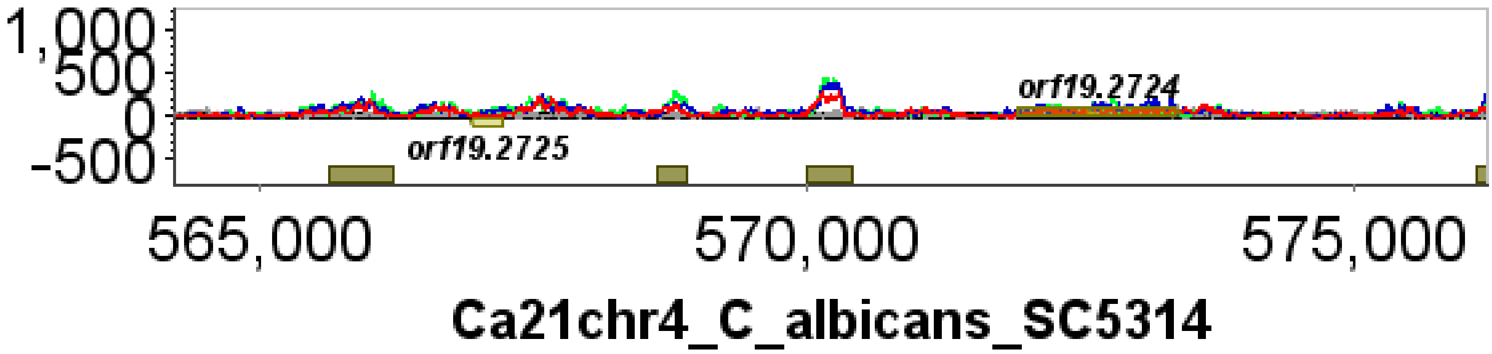

[8.11] Ca21chr1_C_albicans_SC5314:1071555-1083554 [+] [BRG1, orf19.4057, orf19.4059]

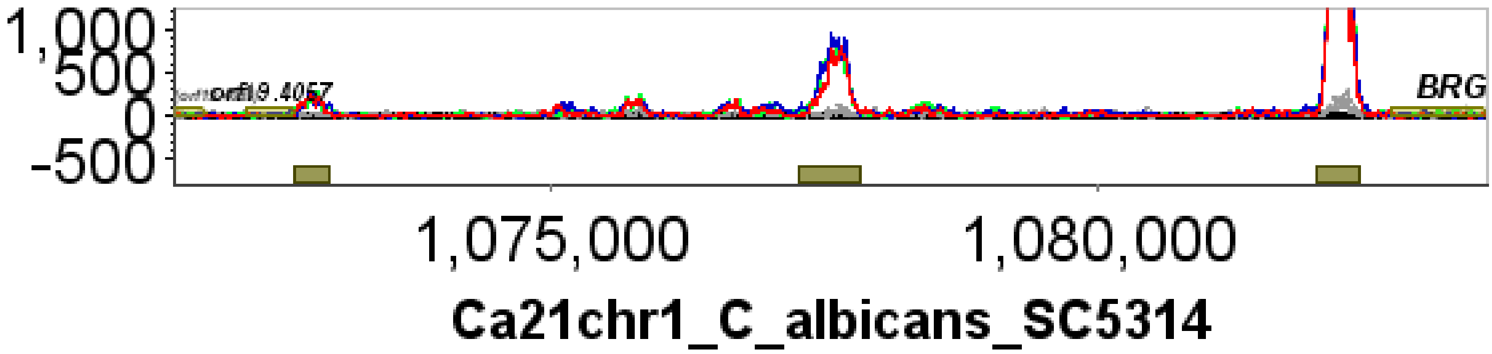

[8.1] Ca21chrR_C_albicans_SC5314:585497-597496 [+] [orf19.2822]

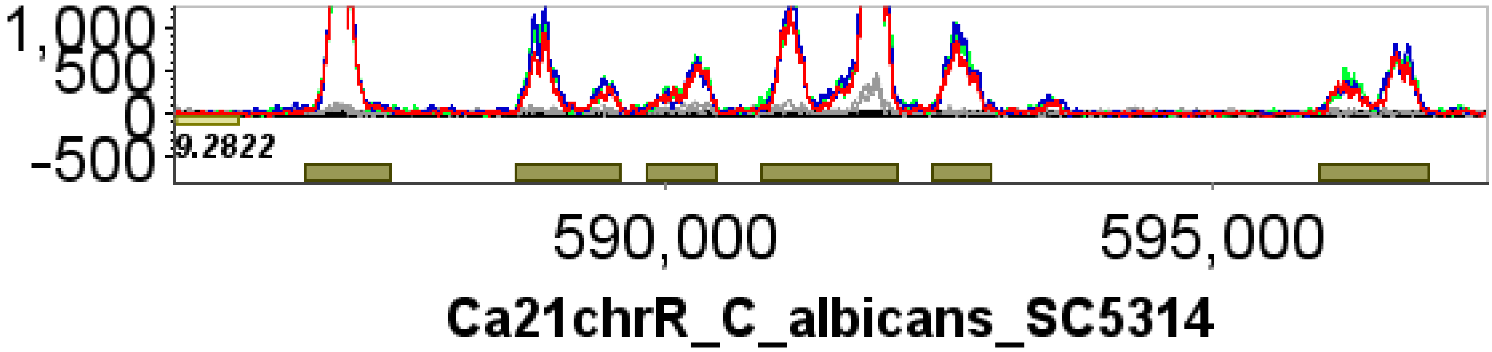

[8.02] Ca21chr5_C_albicans_SC5314:765189-777188 [+] [orf19.2638, tE(UUC)5, orf19.2639, orf19.2639.1, FUR1]

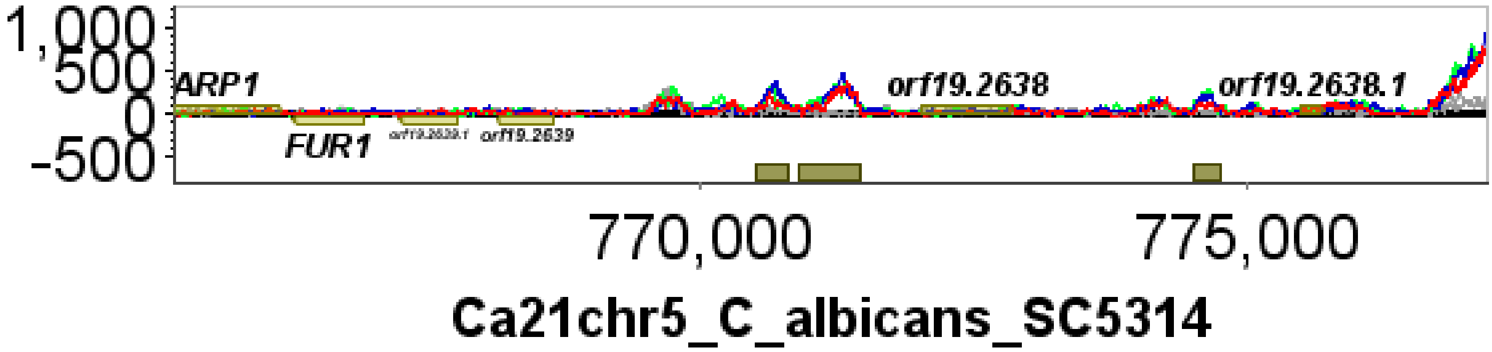

[7.96] Ca21chr1_C_albicans_SC5314:565996-577995 [+] [orf19.2962, orf19.2963, orf19.2961, FRS2, orf19.2964]

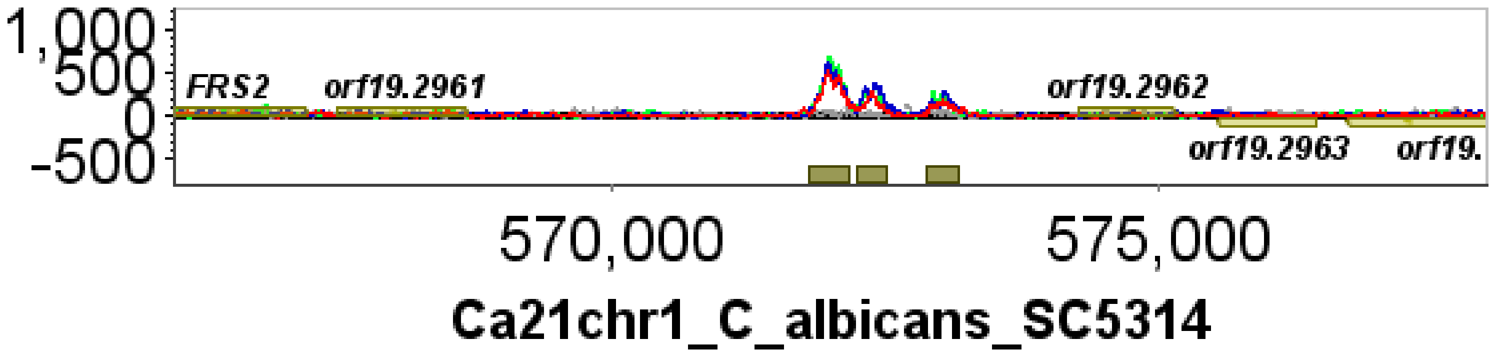

[7.93] Ca21chr1_C_albicans_SC5314:1984165-1996164 [+] [IPT1, orf19.4768, ZCF28, ARG81]

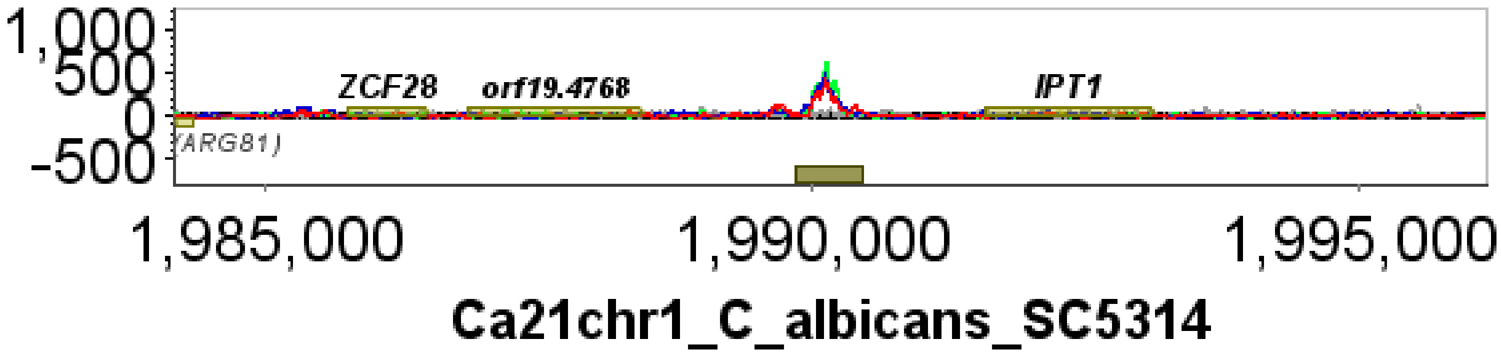

[7.8] Ca21chrR_C_albicans_SC5314:2077994-2089993 [+] [IFA14]

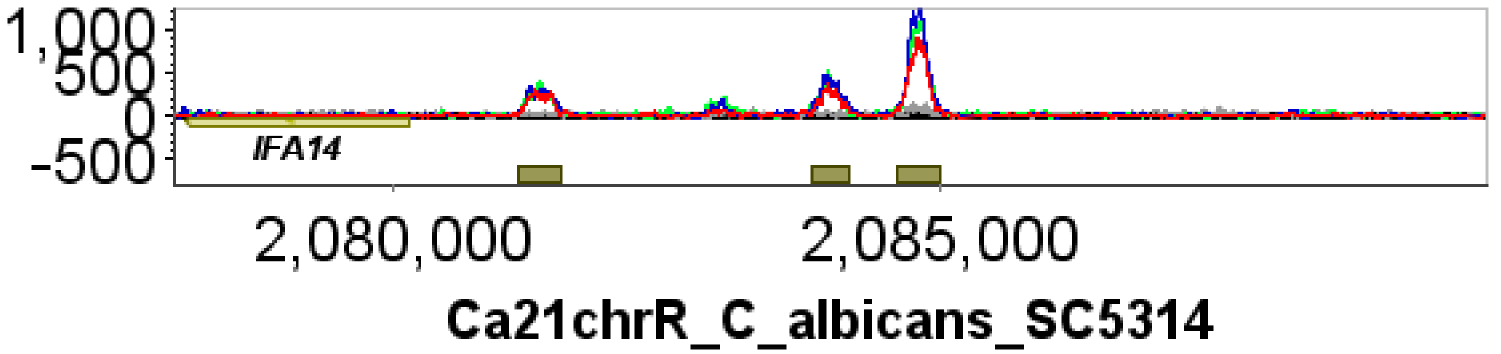

[7.8] Ca21chr7_C_albicans_SC5314:35245-47244 [+] [HGT12, orf19.7095, orf19.7096, HGT13, orf19.7092]

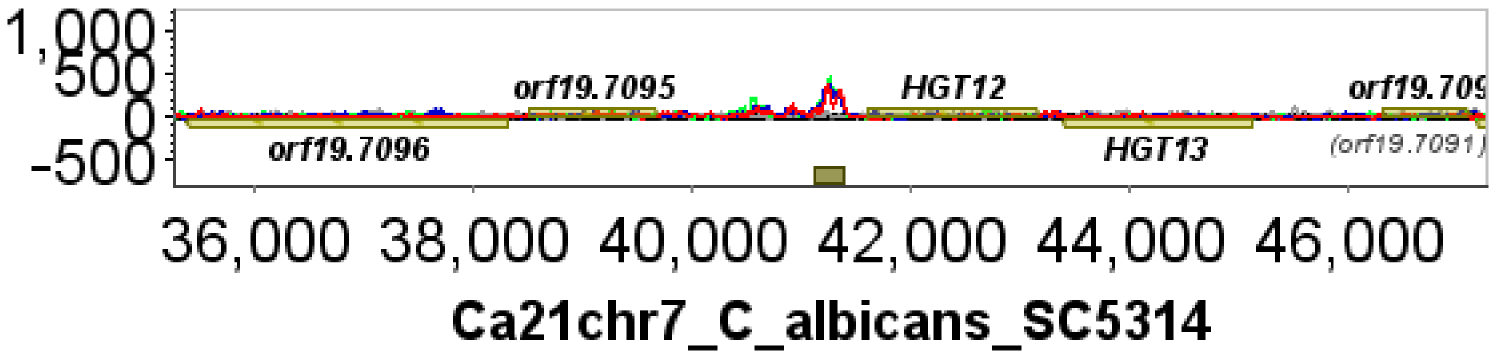

[7.78] Ca21chr1_C_albicans_SC5314:1219524-1231523 [+] [orf19.2459, orf19.2460, PRN4, PRN3, SIP5]

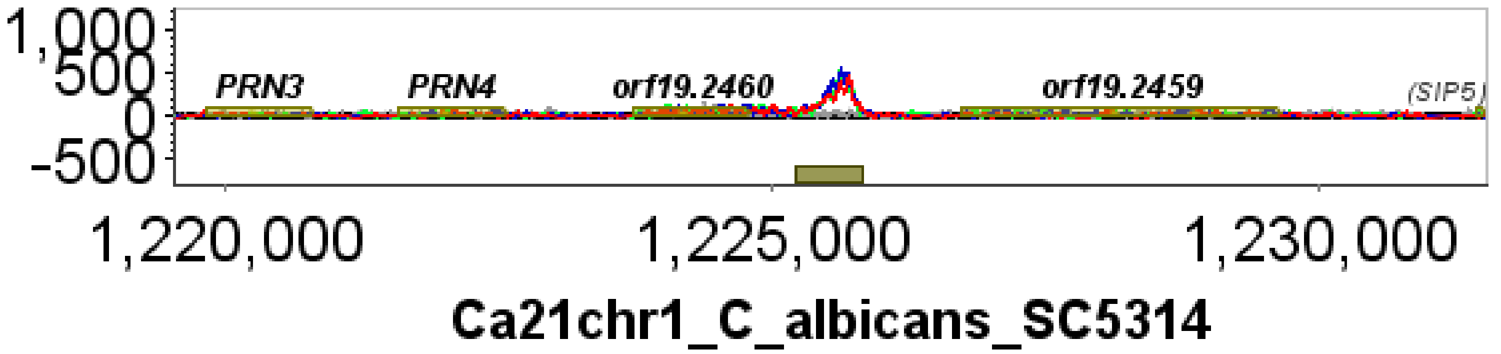

[7.77] Ca21chr2_C_albicans_SC5314:1737276-1749275 [+] [orf19.3621, YWP1, ANP1]

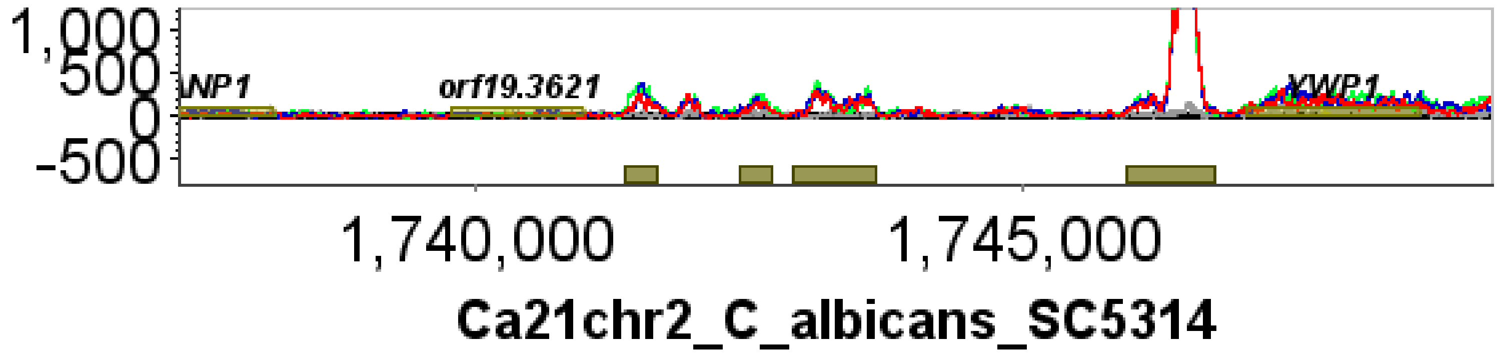

[7.72] Ca21chr5_C_albicans_SC5314:586870-598869 [+] [MNN1, orf19.4280, orf19.4278, IFH1]

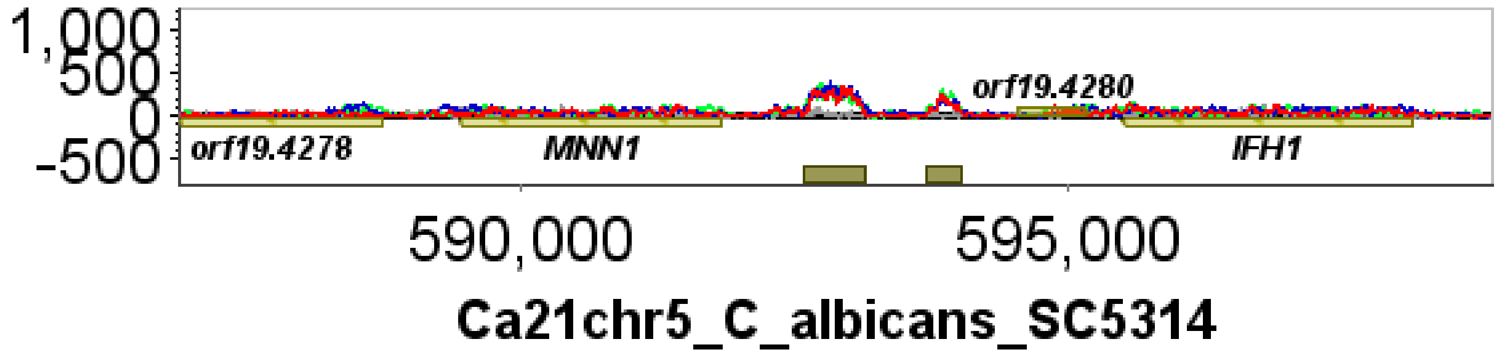

[7.71] Ca21chrR_C_albicans_SC5314:1716886-1728885 [+] [EFG1, orf19.609, orf19.607]

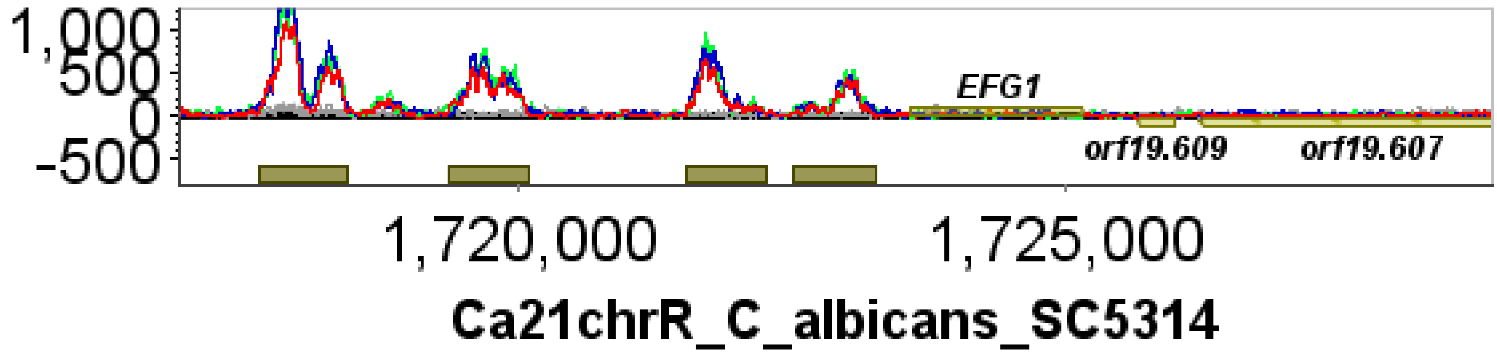

[7.71] Ca21chr6_C_albicans_SC5314:37320-49319 [+] [STV1, orf19.1189, orf19.1191]

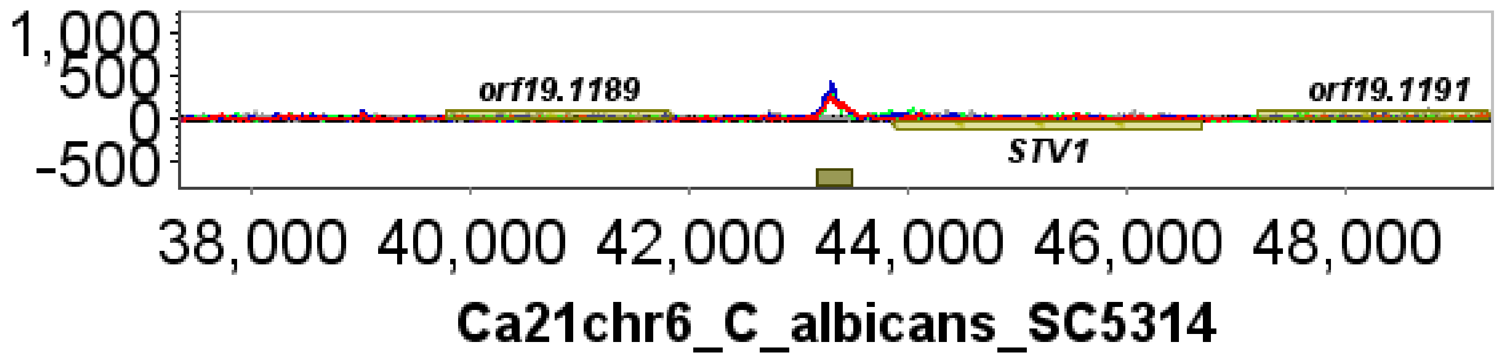

[7.71] Ca21chr5_C_albicans_SC5314:157787-169786 [+] [IFF8, SPE2, TFB3, RMT2]

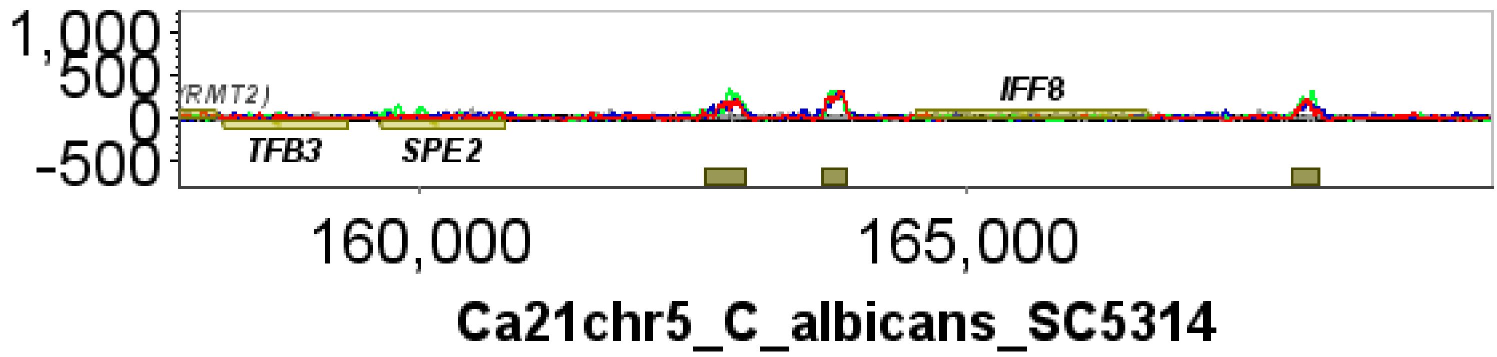

[7.7] Ca21chr3_C_albicans_SC5314:1359309-1371308 [+] [CAM1, AHR1, MNN9, NOG1]

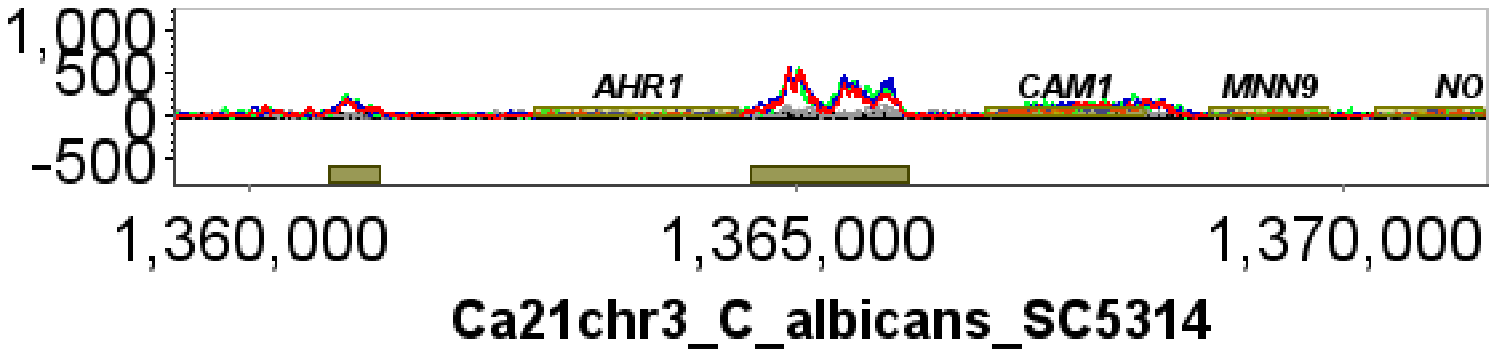

[7.68] Ca21chr2_C_albicans_SC5314:1310708-1322707 [+] [RTA2, RTA3, orf19.25, orf19.26, tI(AAU)1]

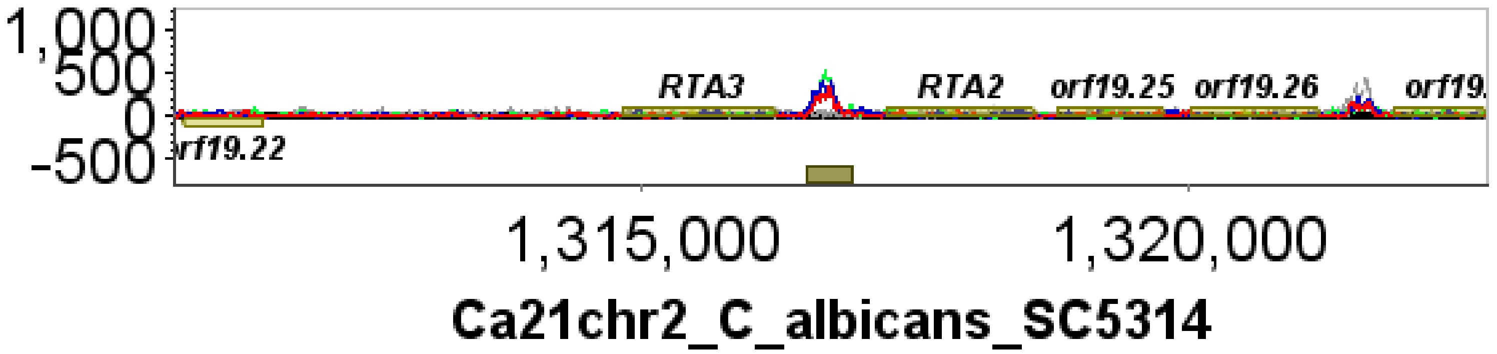

[7.66] Ca21chrR_C_albicans_SC5314:1185801-1197800 [+] [tC(GCA)1, tC(GCA)2, orf19.5844, RNR3, SRR1]

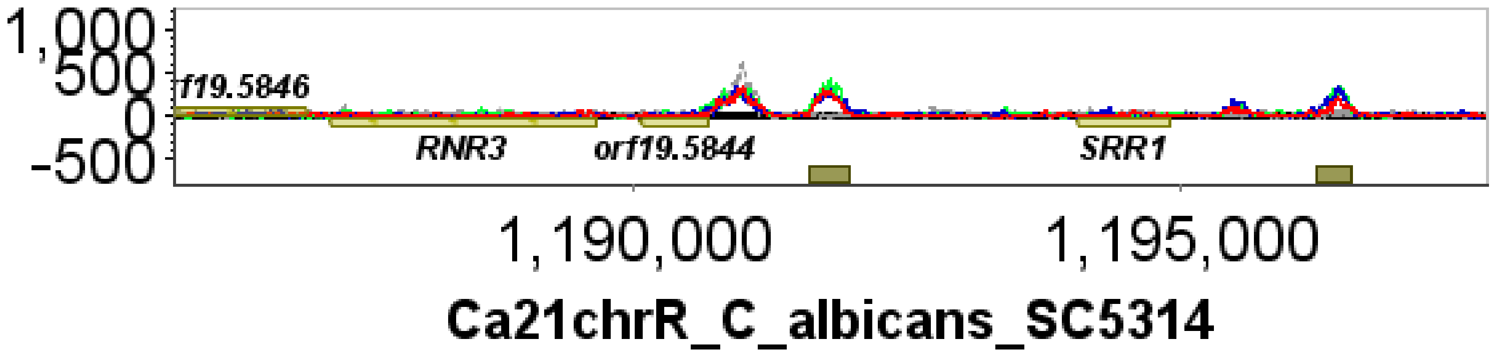

[7.65] Ca21chr4_C_albicans_SC5314:870133-882132 [+] [PGA31, orf19.5300, ECM1, PGA30, orf19.5299.1]

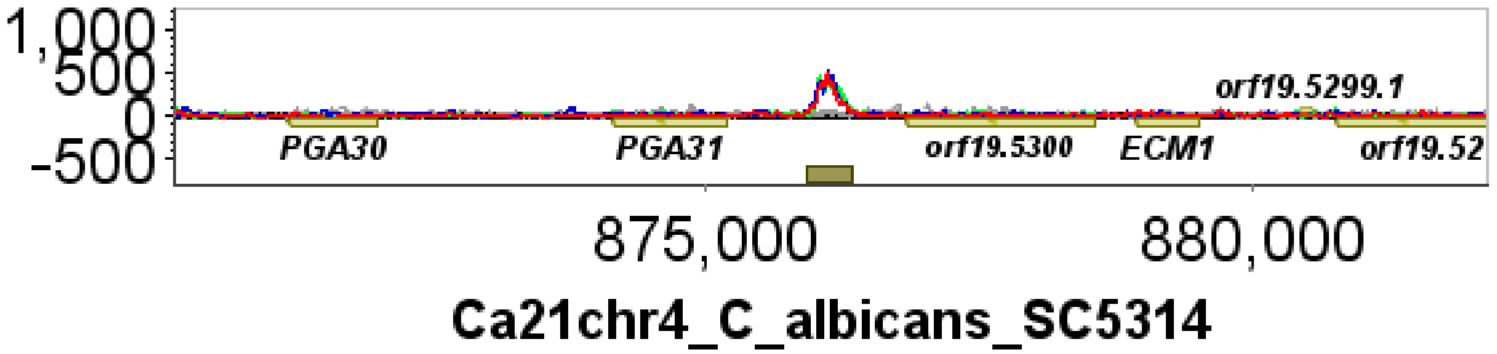

[7.64] Ca21chrR_C_albicans_SC5314:1309212-1321211 [+] [orf19.3868, orf19.3869, ADE13, DAD3, RPL7]

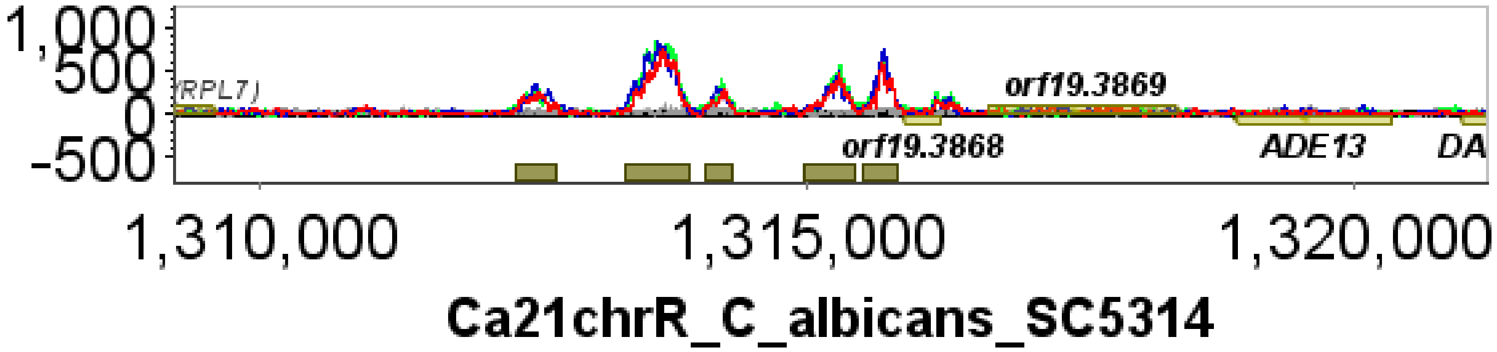

[7.64] Ca21chr3_C_albicans_SC5314:997012-1009011 [+] [orf19.5933, orf19.5934, orf19.5932, orf19.5935, SEN1]

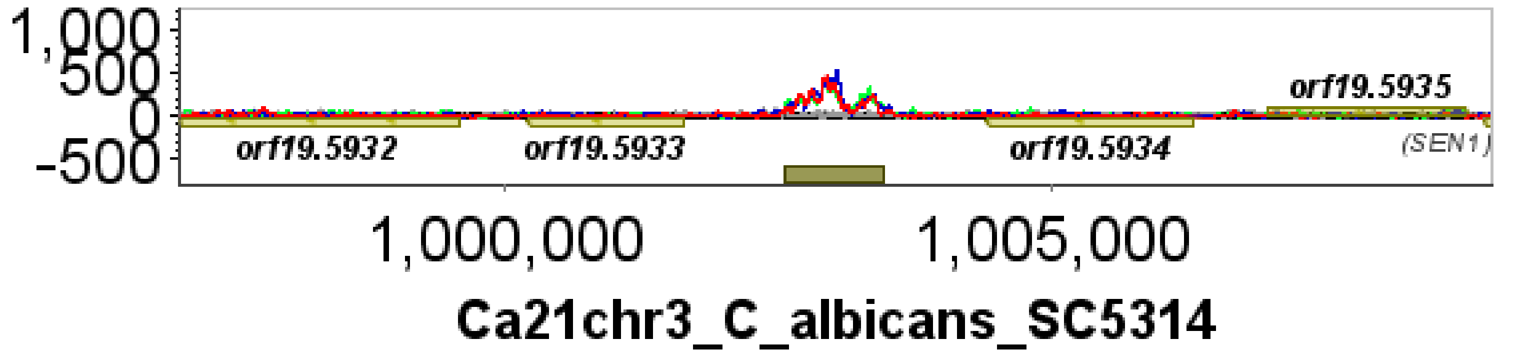

[7.64] Ca21chr1_C_albicans_SC5314:1085694-1097693 [+] [orf19.449, CUE5, orf19.446.2]

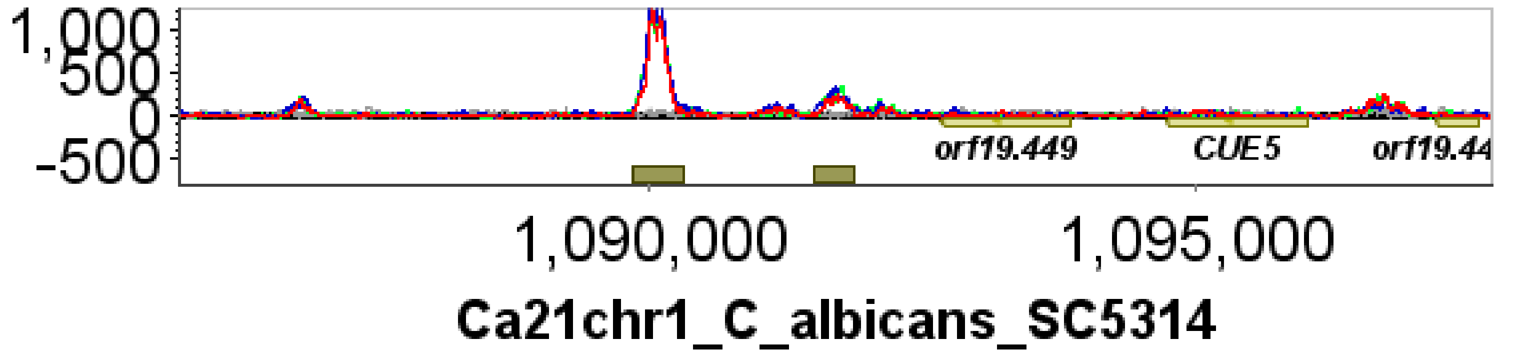

[7.63] Ca21chr1_C_albicans_SC5314:3105184-3117183 [+] [RBE1, FTR1, RPL4B, YPT52, orf19.7215.3]

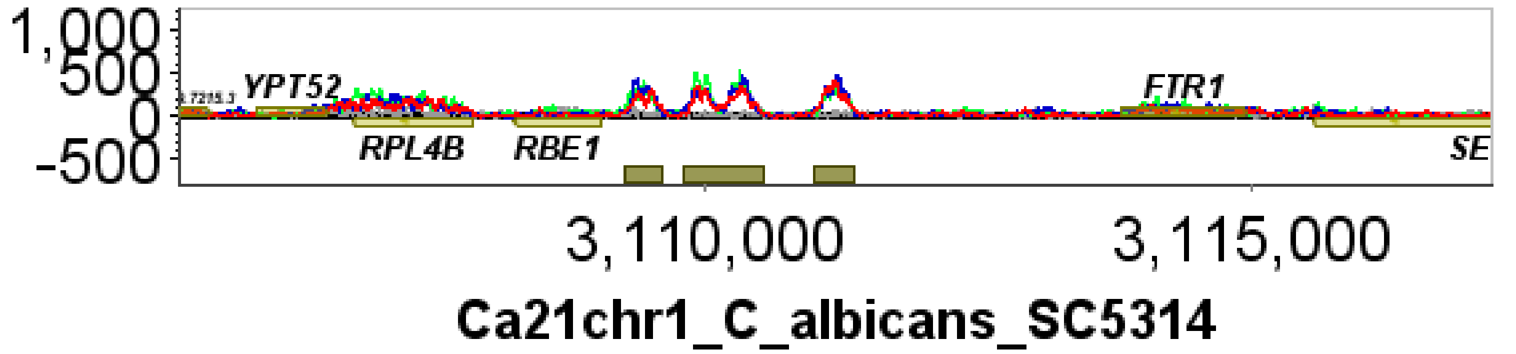

[7.52] Ca21chrR_C_albicans_SC5314:1712027-1724026 [+] [tG(GCC)1, orf19.612, EFG1]

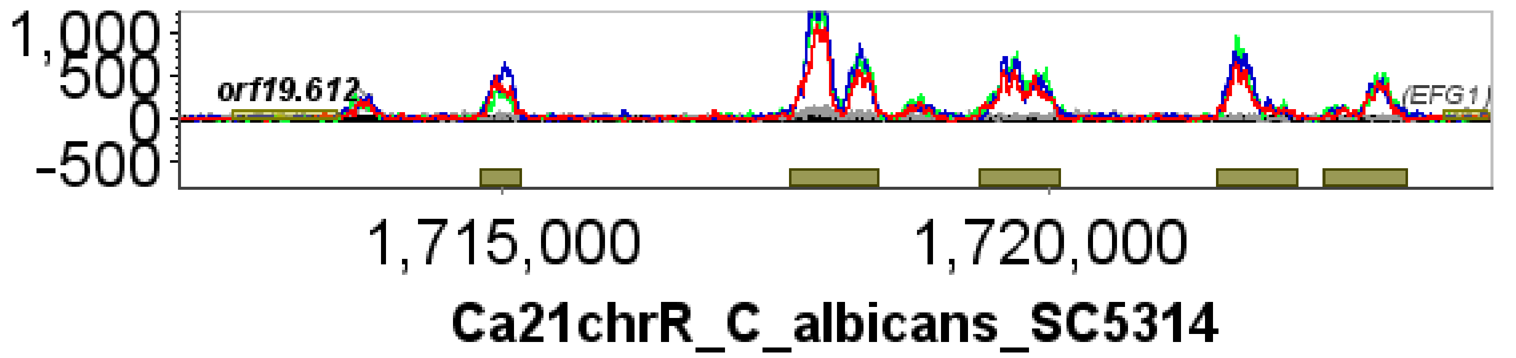

[7.45] Ca21chrR_C_albicans_SC5314:868755-880754 [+] [WOR3, IFF9]

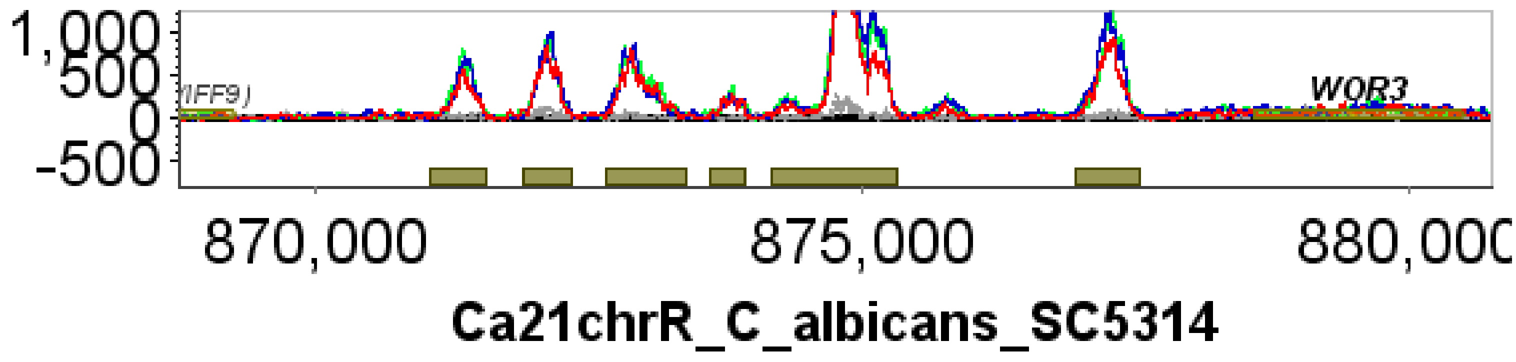

[7.43] Ca21chrR_C_albicans_SC5314:2075353-2087352 [+] [IFA14, PMT5]

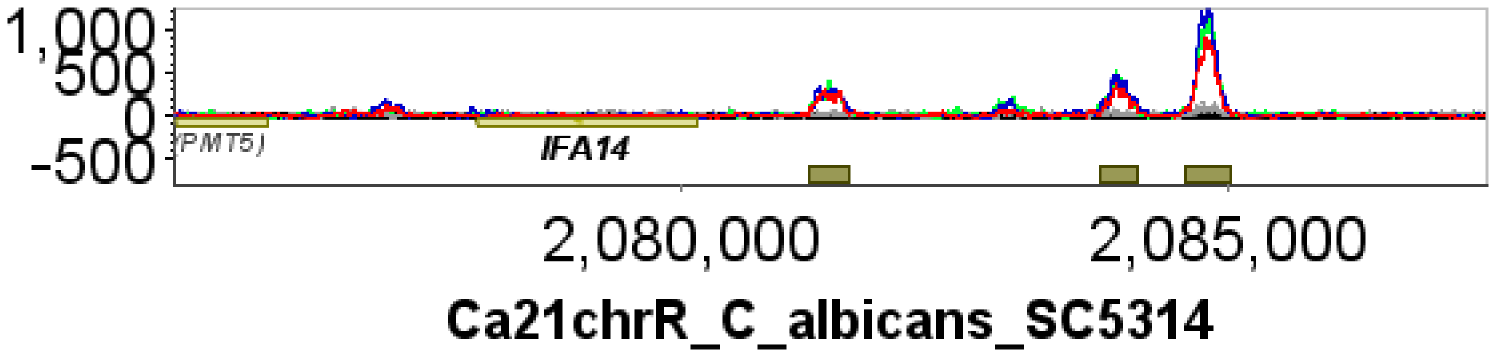

[7.42] Ca21chr5_C_albicans_SC5314:918203-930202 [+] [orf19.3897, CHT2, orf19.3898, SDC1, orf19.3900]

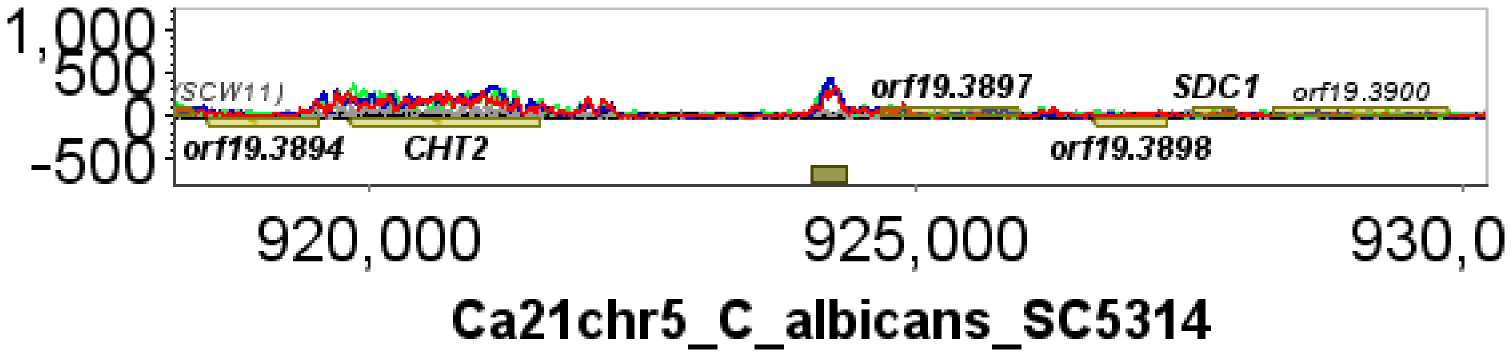

[7.41] Ca21chr1_C_albicans_SC5314:293017-305016 [+] [orf19.3337, orf19.3336]

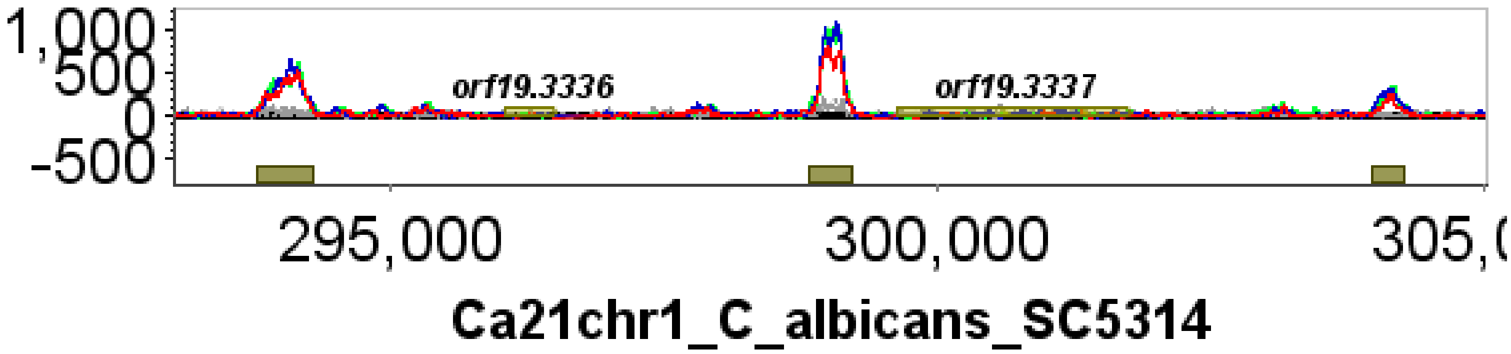

[7.35] Ca21chr1_C_albicans_SC5314:2227652-2239651 [+] [MIR1, WOR1, orf19.4886]

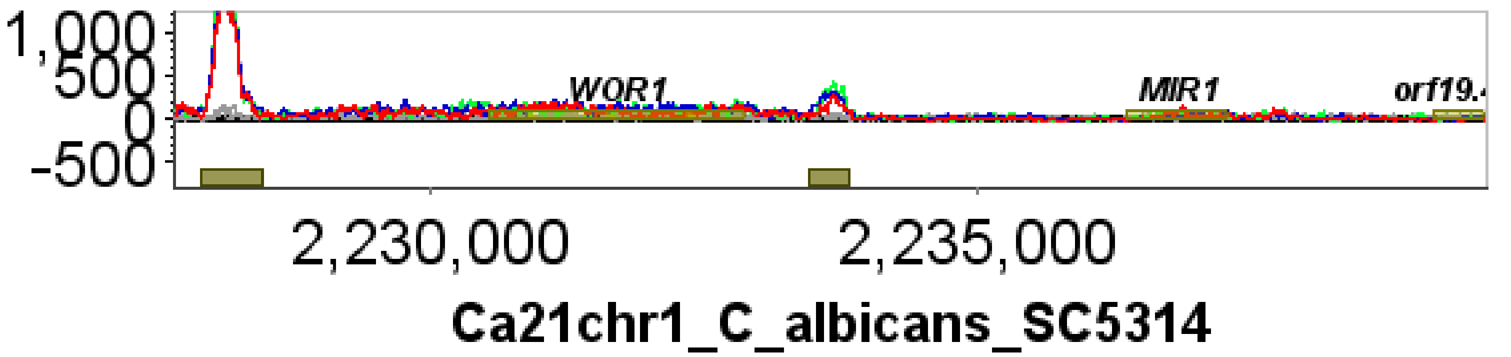

[7.34] Ca21chr1_C_albicans_SC5314:3103434-3115433 [+] [RBE1, RPL4B, YPT52, orf19.7215.3, FTR1]

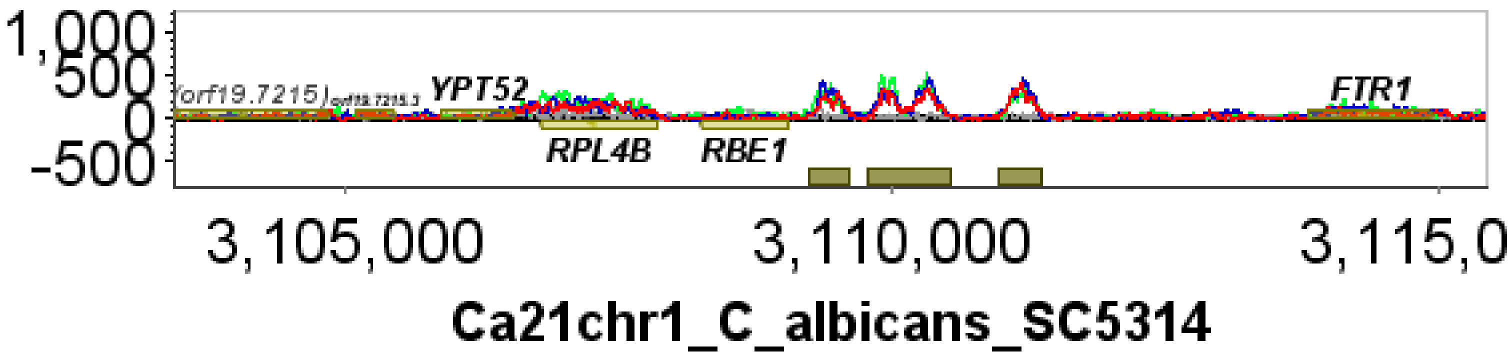

[7.34] Ca21chr1_C_albicans_SC5314:2268697-2280696 [+] [MNN12, GCA1, orf19.4898, orf19.4901]

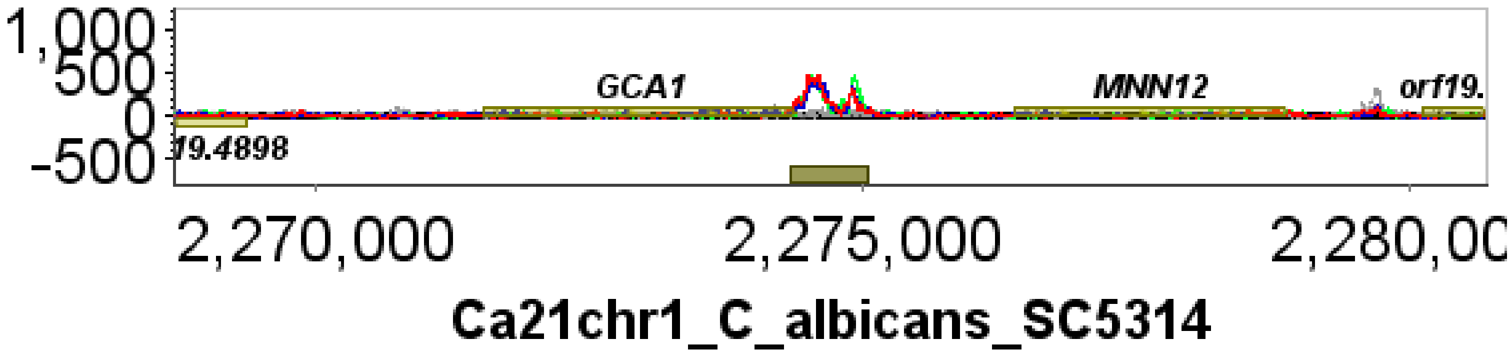

[7.31] Ca21chr1_C_albicans_SC5314:2217258-2229257 [+] [orf19.4883, snR10a, orf19.4882]

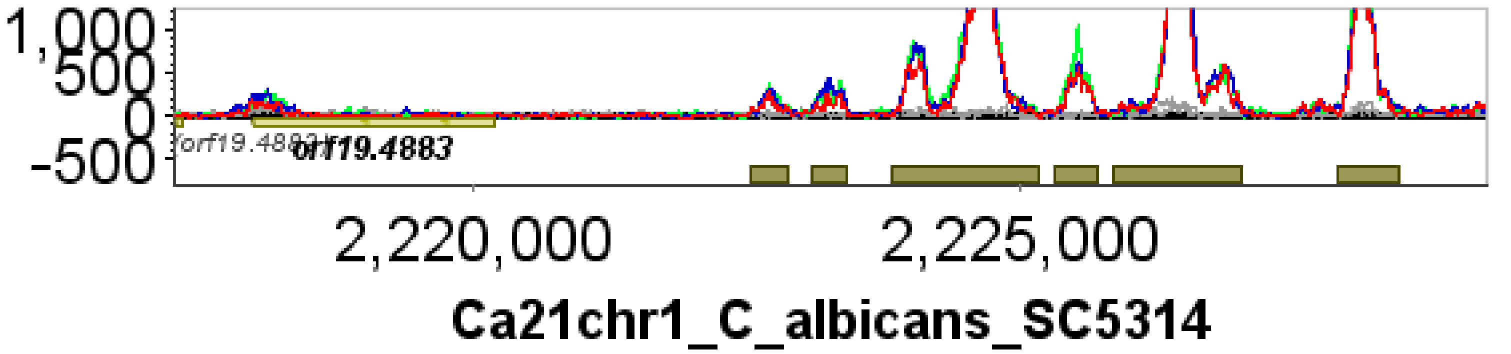

[7.25] Ca21chr3_C_albicans_SC5314:1169376-1181375 [+] [orf19.6968, USO6, orf19.6966]

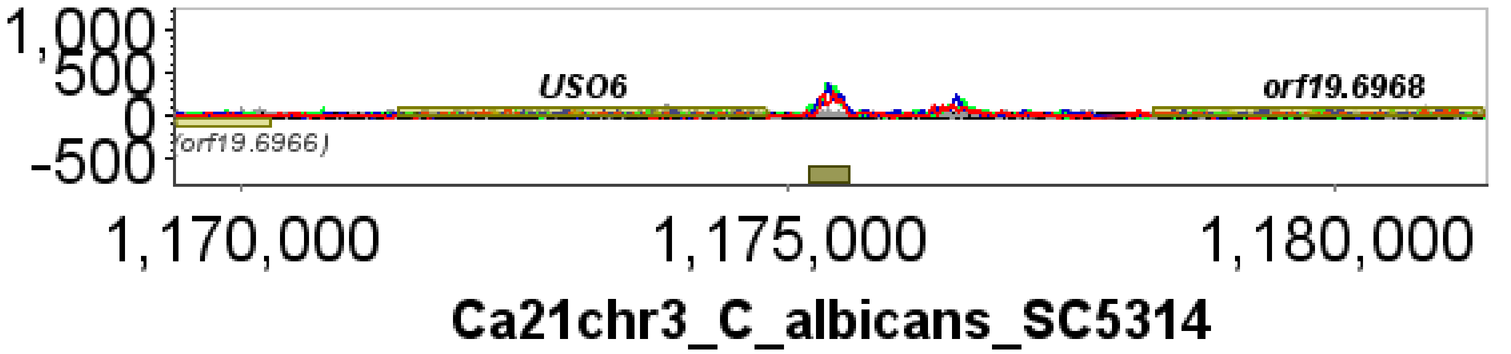

[7.19] Ca21chrR_C_albicans_SC5314:1405022-1417021 [+] [RGS2, orf19.693, snR5d]

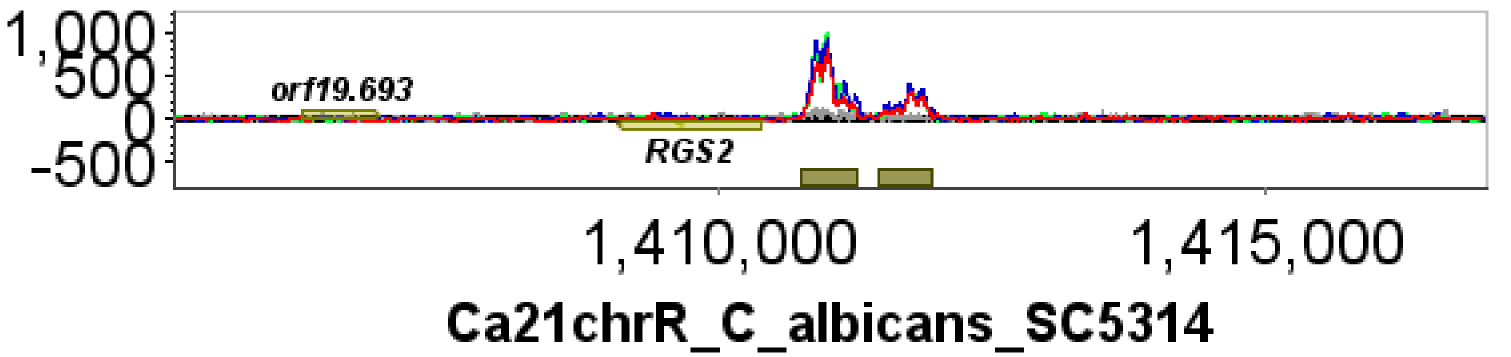

[7.17] Ca21chr2_C_albicans_SC5314:1797688-1809687 [+] [PIR1, BUD20, orf19.217, orf19.223]

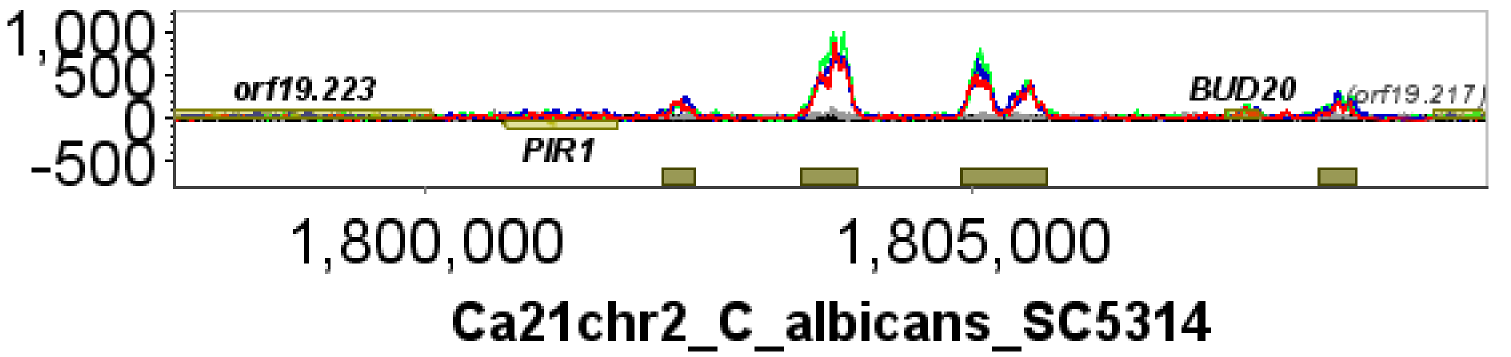

[7.15] Ca21chr5_C_albicans_SC5314:251056-263055 [+] [orf19.1958, orf19.1959, CYC3, orf19.1956]

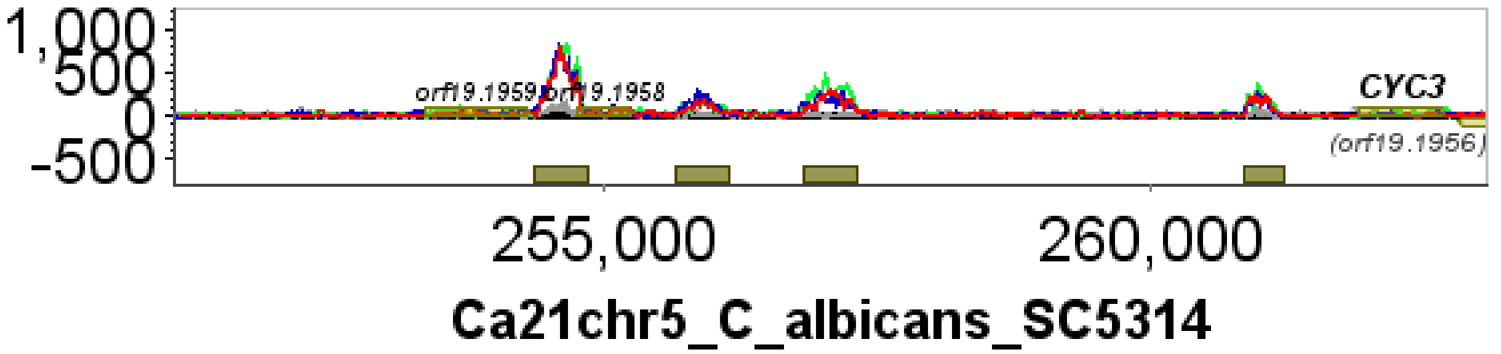

[7.13] Ca21chrR_C_albicans_SC5314:1405720-1417719 [+] [RGS2, snR5d, orf19.693]

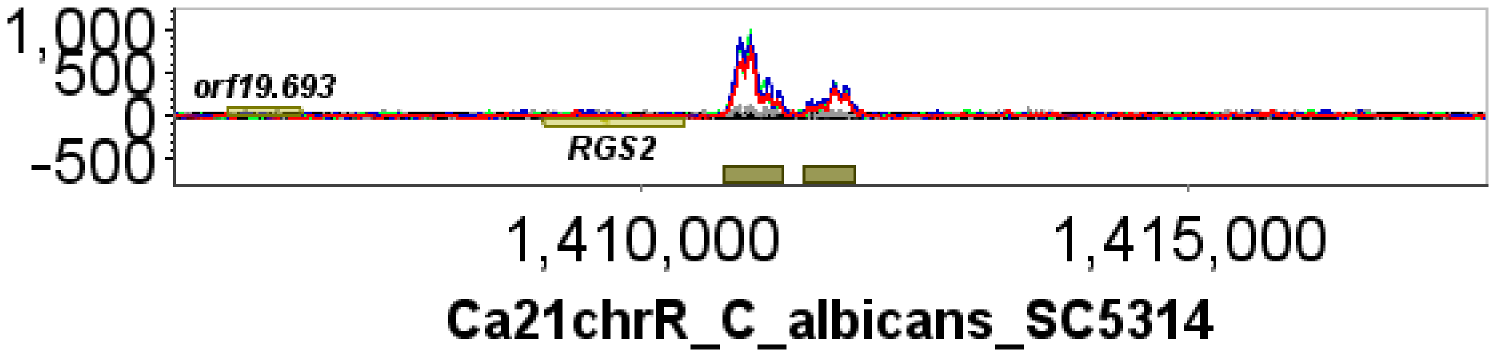

[7.06] Ca21chr5_C_albicans_SC5314:1109813-1121812 [+] [GRF10, orf19.4001, orf19.3999]

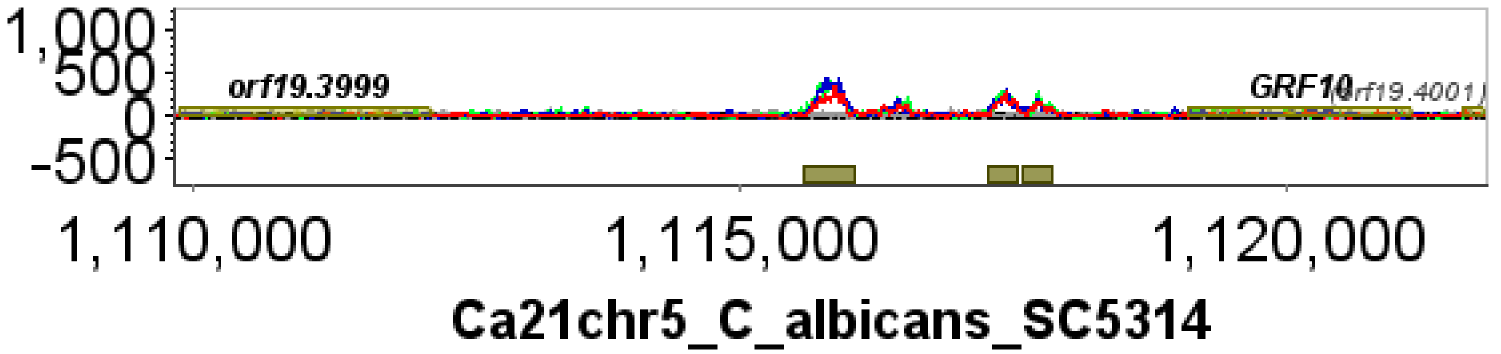

[7.06] Ca21chr5_C_albicans_SC5314:489298-501297 [+] [orf19.4230, PTH2, orf19.4229, orf19.4228, orf19.4227]

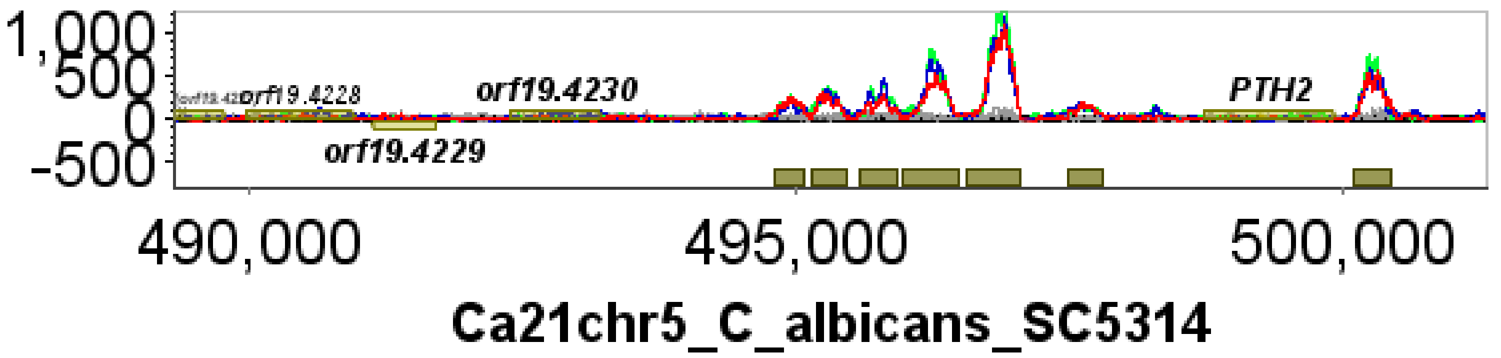

[7.06] Ca21chr1_C_albicans_SC5314:1323005-1335004 [+] [orf19.1821, snR5b, snR44a]

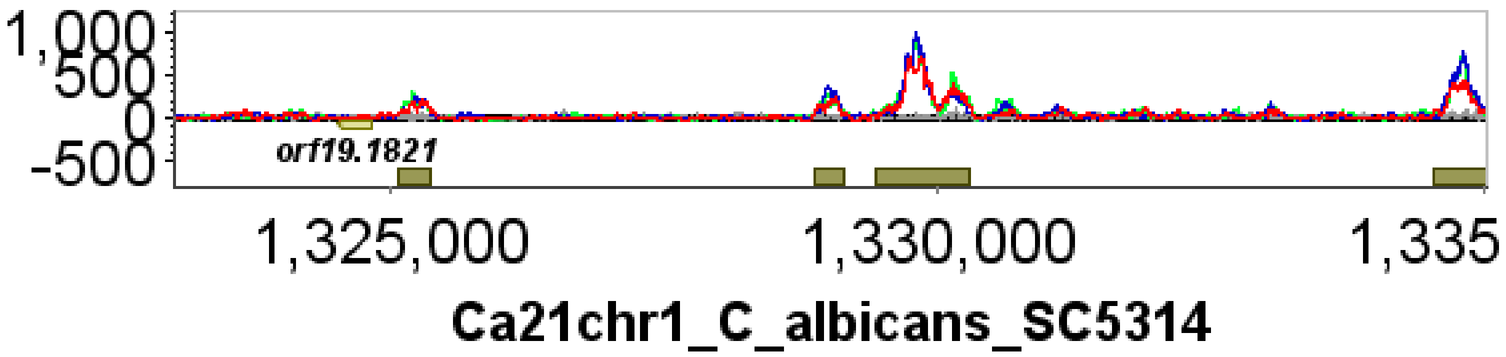

[7.01] Ca21chr2_C_albicans_SC5314:737606-749605 [+] [ADAEC, orf19.867]

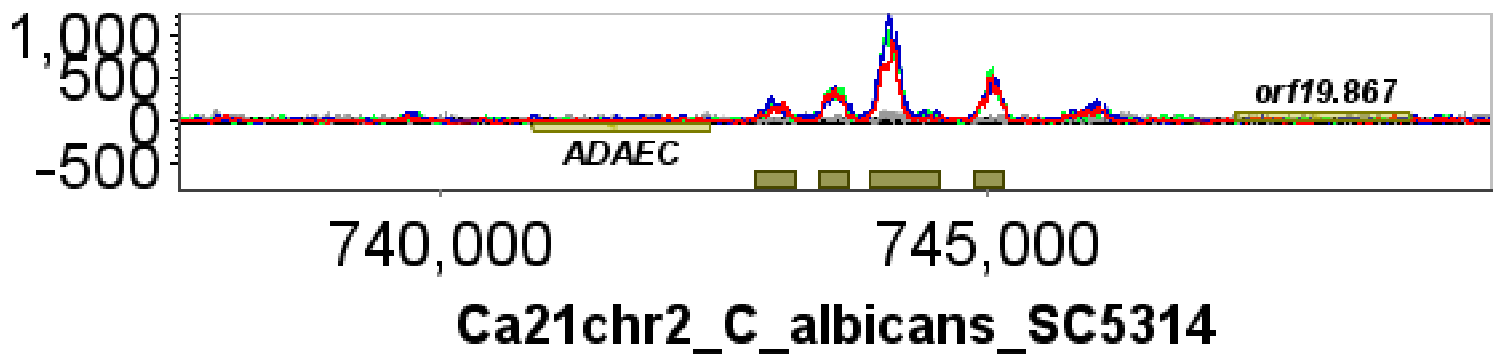

[7.0] Ca21chr2_C_albicans_SC5314:727302-739301 [+] [orf19.871]

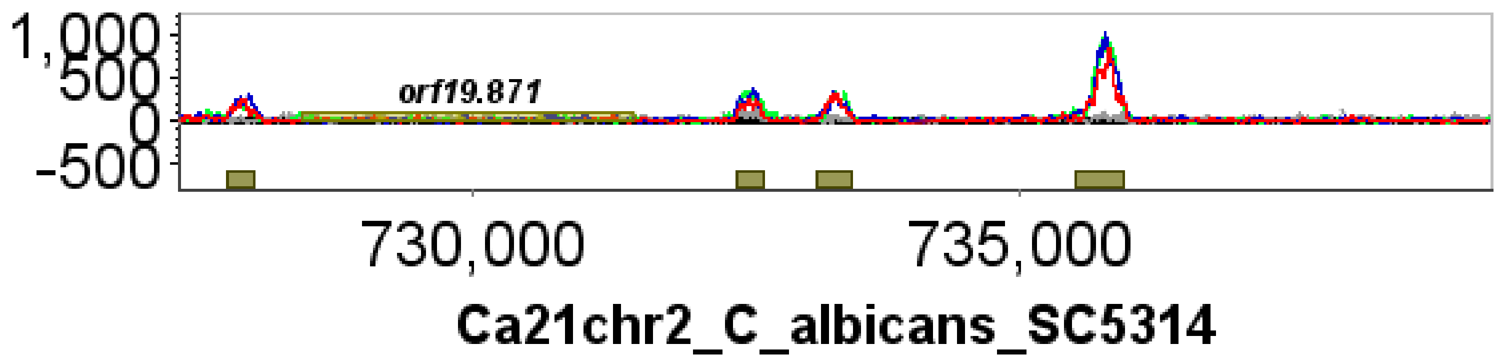

[6.98] Ca21chr3_C_albicans_SC5314:1469203-1481202 [+] [AAF1, ECM15, tH(GUG)3]

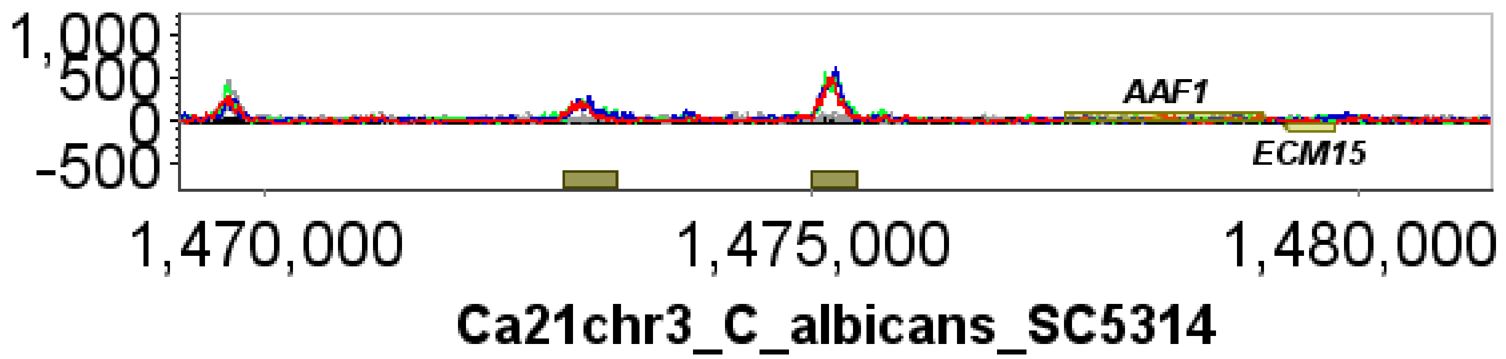

[6.96] Ca21chr1_C_albicans_SC5314:3065406-3077405 [+] [SIM1, SSK1, orf19.5033, YBP1, DOS2]

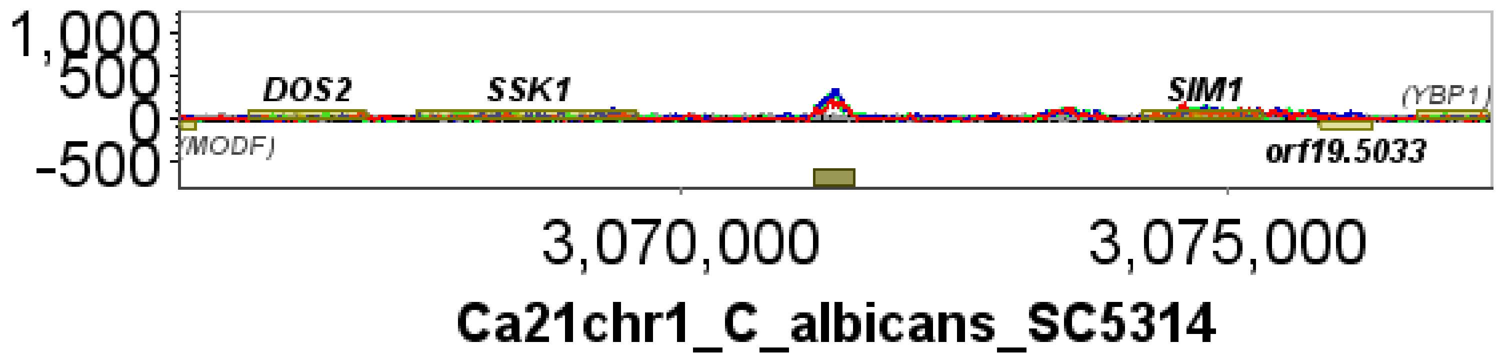

[6.92] Ca21chr1_C_albicans_SC5314:2617458-2629457 [+] [orf19.5267, MED10, orf19.5266, orf19.5269, orf19.5269.1]

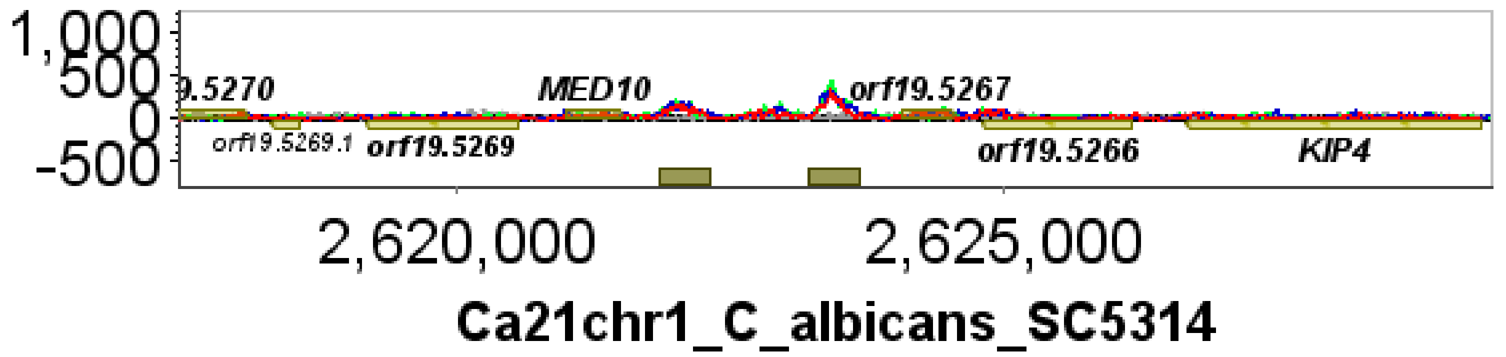

[6.87] Ca21chr1_C_albicans_SC5314:491484-503483 [+] [orf19.3694, orf19.2925, orf19.2926, PGA5, MNN11]

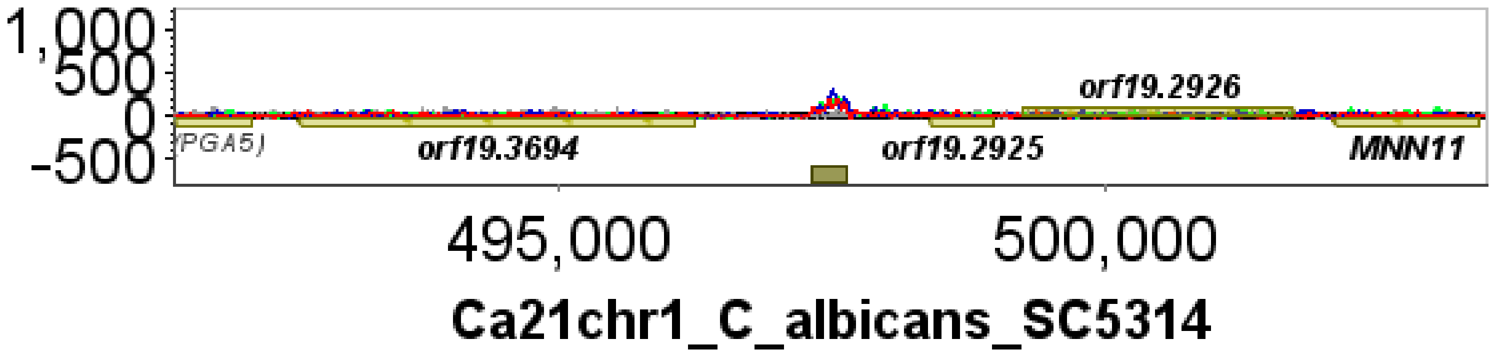

[6.86] Ca21chr7_C_albicans_SC5314:442406-454405 [+] [CUP9]

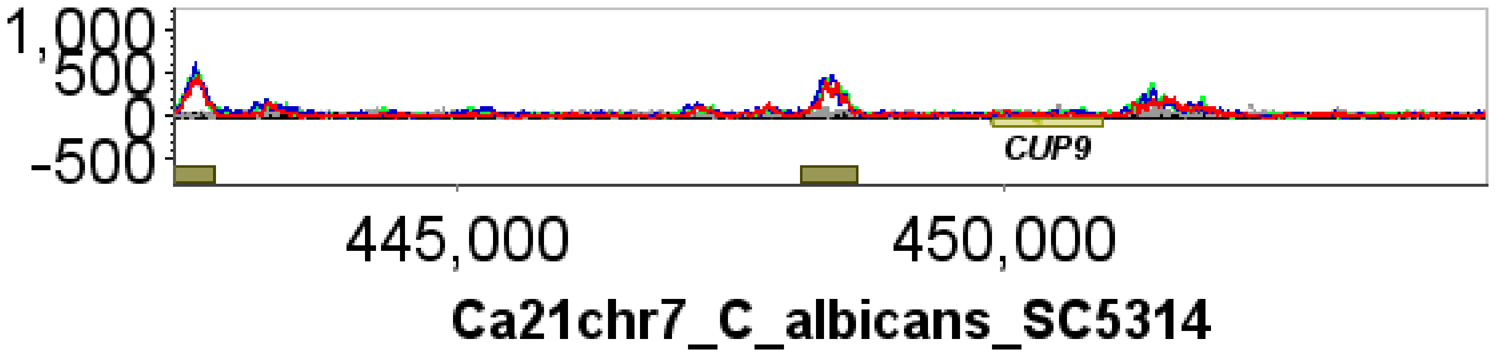

[6.84] Ca21chr2_C_albicans_SC5314:12413-24412 [+] [orf19.1906, EMC9, NDT80, LEU5, orf19.1910]

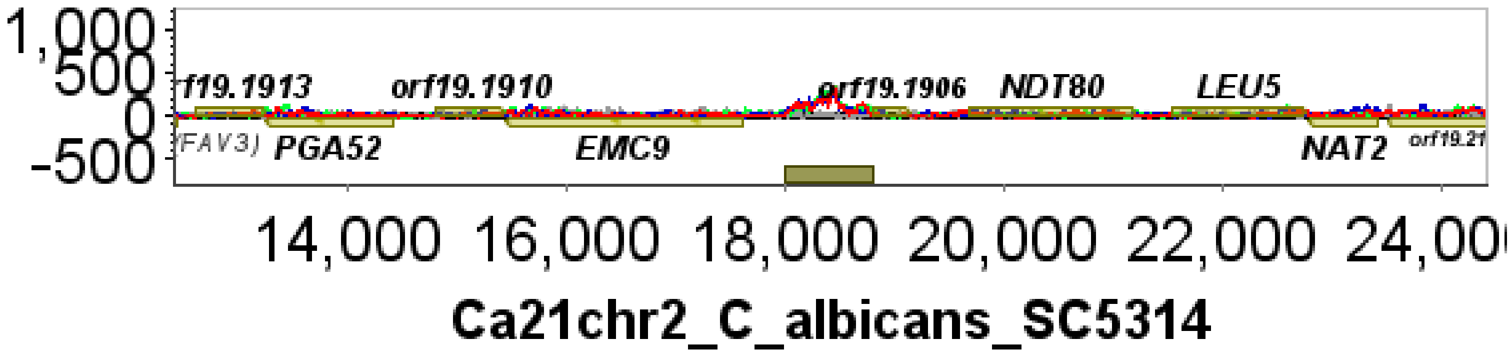

[6.75] Ca21chr1_C_albicans_SC5314:533560-545559 [+] [PUT4, HNM4, orf19.2943.5, tR(UCU)4, SNZ1]

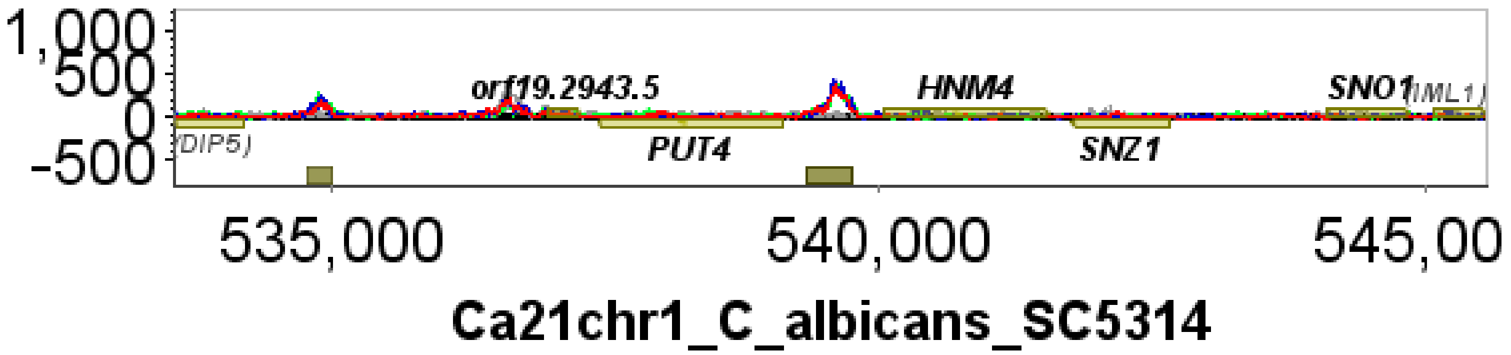

[6.72] Ca21chr2_C_albicans_SC5314:1139405-1151404 [+] [tS(AGA)4, KTI11, RPS8A, snR56, orf19.6874]

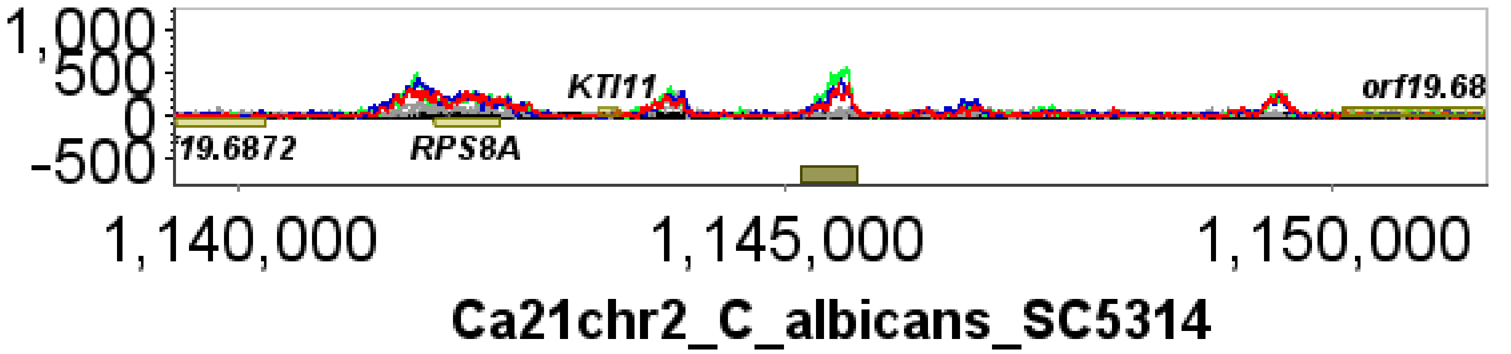

[6.7] Ca21chr1_C_albicans_SC5314:2935297-2947296 [+] [HYR1, orf19.4972]

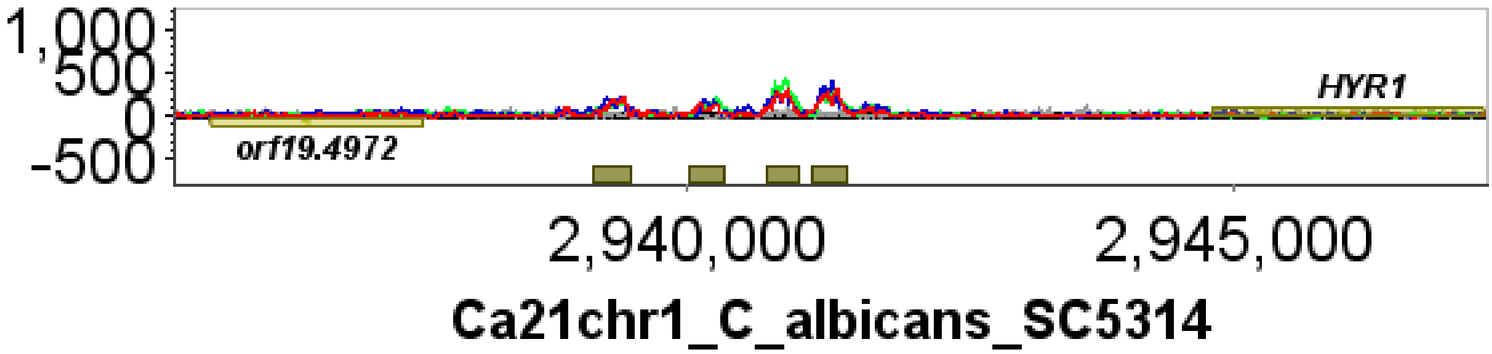

[6.67] Ca21chr1_C_albicans_SC5314:1319217-1331216 [+] [orf19.1821]

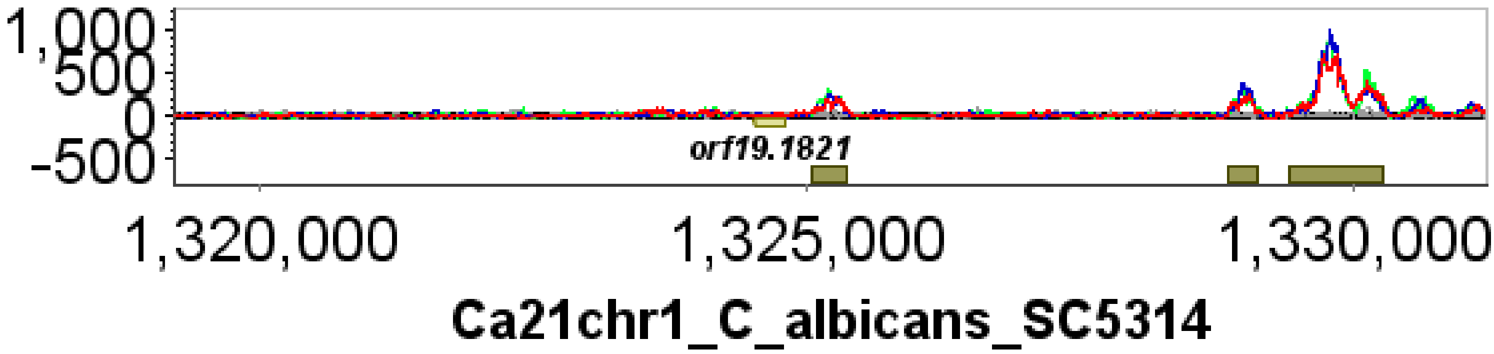

[6.64] Ca21chr4_C_albicans_SC5314:464214-476213 [+] [PGA59, AMS1]

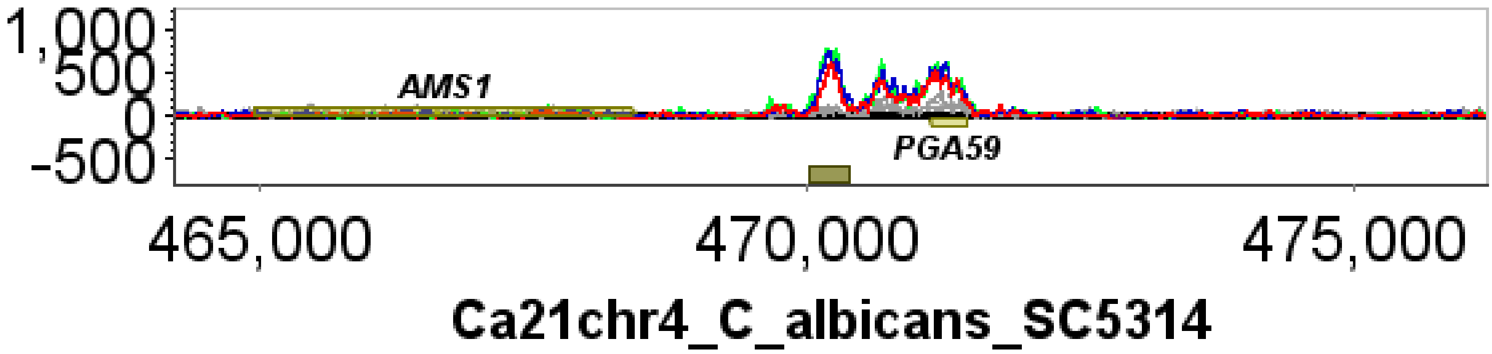

[6.64] Ca21chr3_C_albicans_SC5314:1721166-1733165 [+] [TCC1, orf19.6736, orf19.6732]

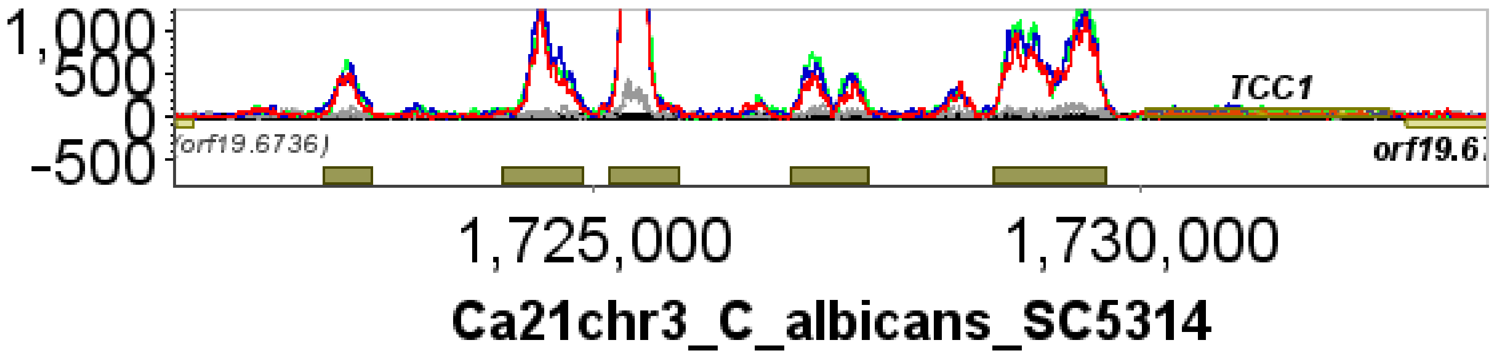

[6.63] Ca21chr2_C_albicans_SC5314:1735510-1747509 [+] [orf19.3621, ANP1, YWP1, SMC2]

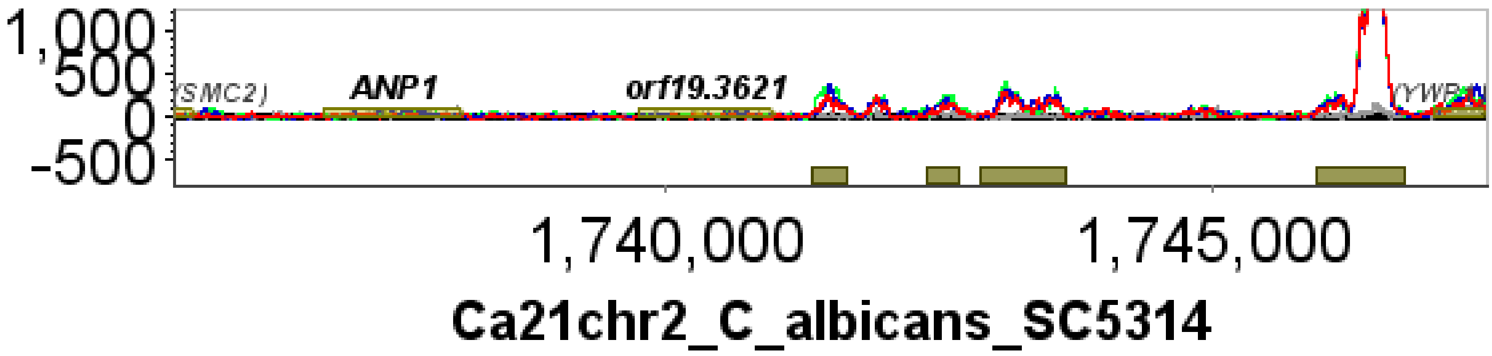

[6.63] Ca21chr1_C_albicans_SC5314:1670351-1682350 [+] [VRG4, ADE4, orf19.1229]

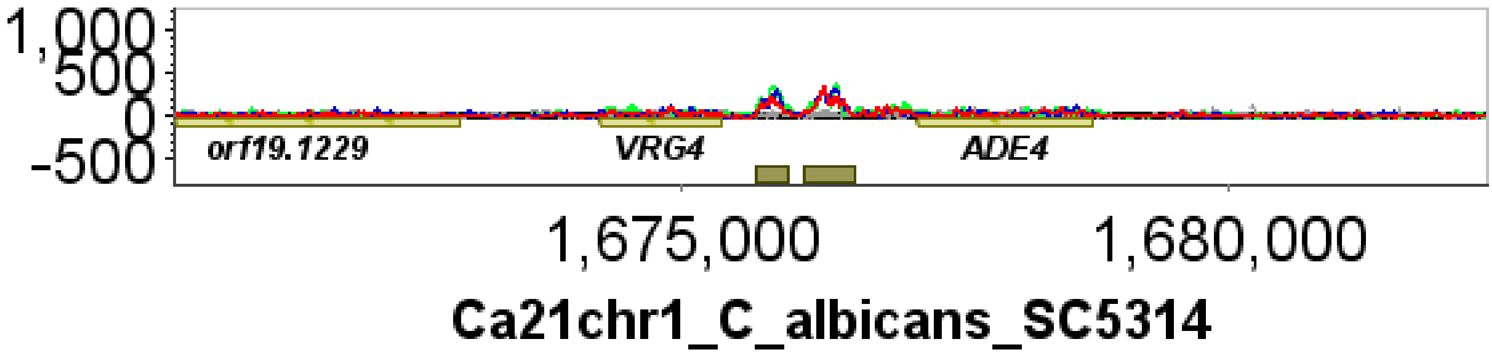

[6.59] Ca21chr4_C_albicans_SC5314:857880-869879 [+] [JEN2, orf19.5308, orf19.5306, RHD3, orf19.5311]

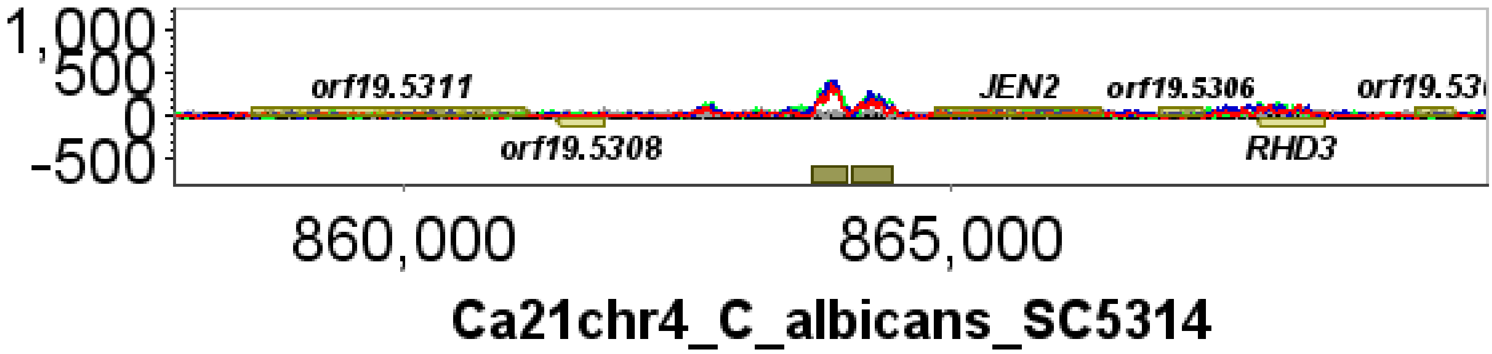

[6.52] Ca21chr7_C_albicans_SC5314:356132-368131 [+] [orf19.6556, orf19.6557, orf19.6558, orf19.6559]

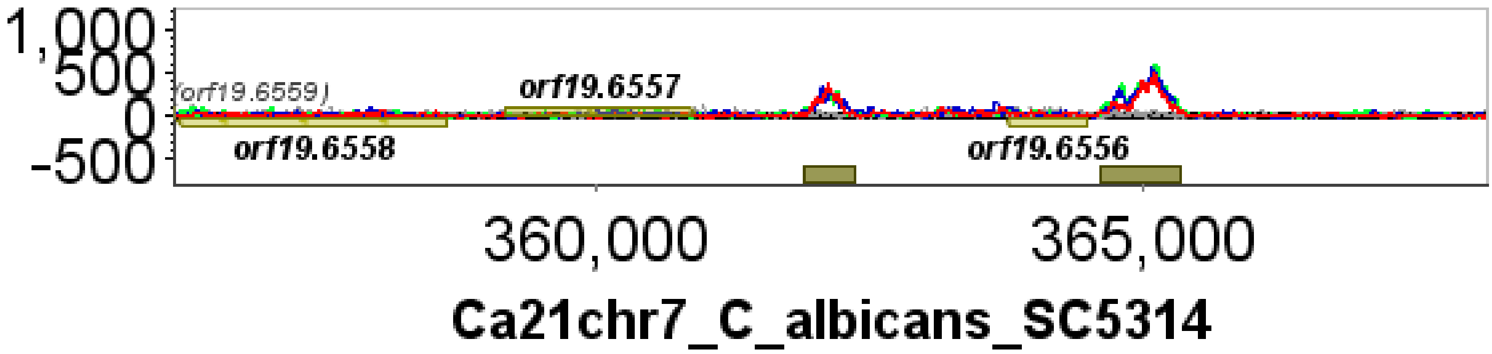

[6.49] Ca21chrR_C_albicans_SC5314:260119-272118 [+] [orf19.3238, orf19.3237, CTF18]

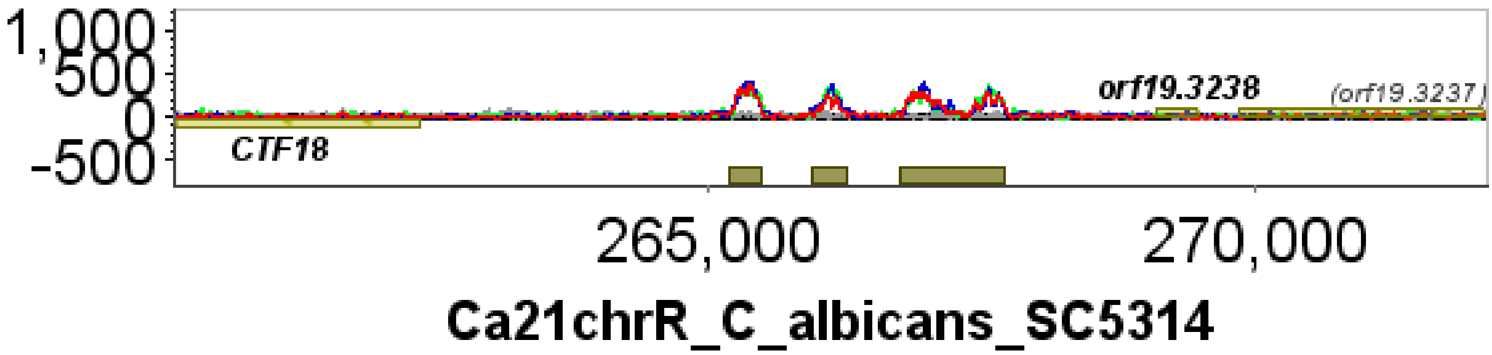

[6.48] Ca21chr2_C_albicans_SC5314:721877-733876 [+] [orf19.871, orf19.872, orf19.873, COX6, orf19.874]

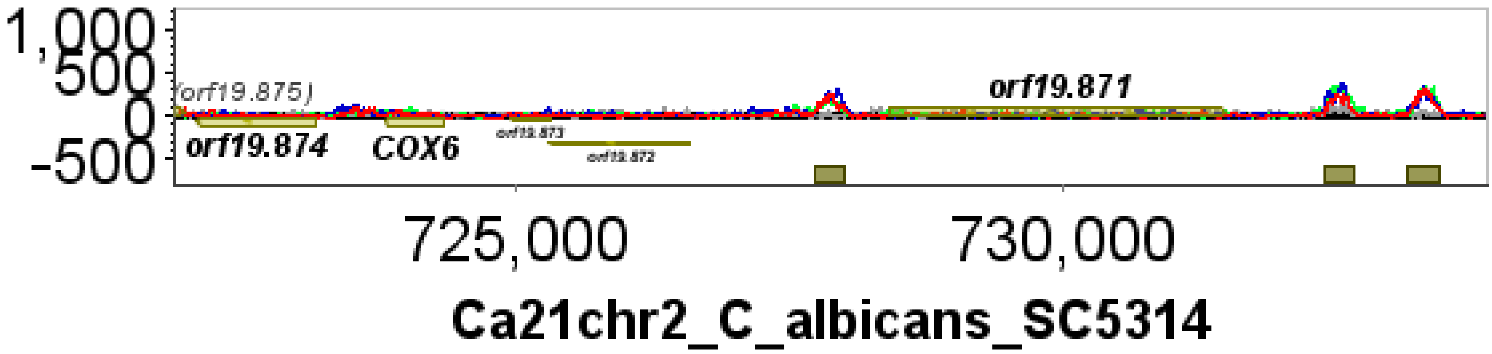

[6.41] Ca21chr7_C_albicans_SC5314:719367-731366 [+] [LIP8, RBR3]

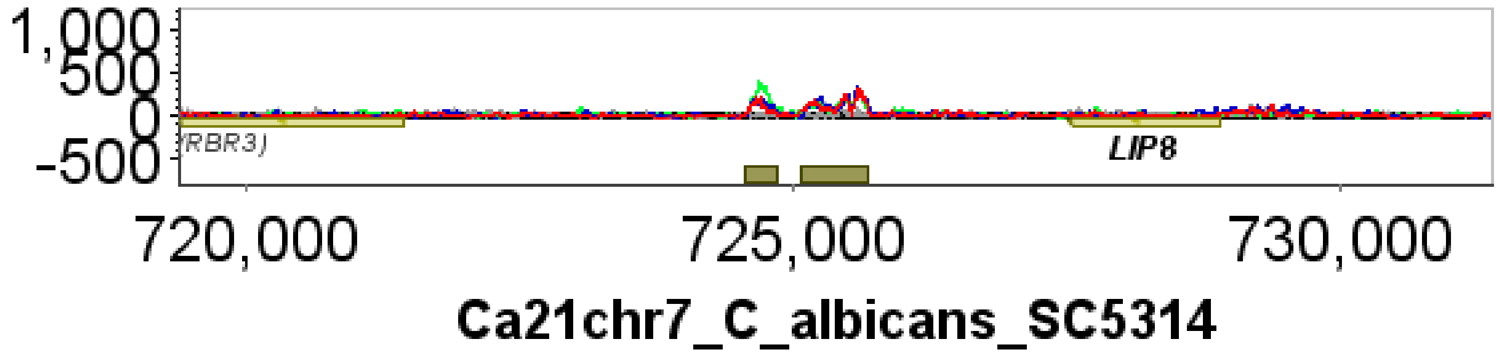

[6.39] Ca21chr5_C_albicans_SC5314:96145-108144 [+] [orf19.938, orf19.937, orf19.936, NAM7]

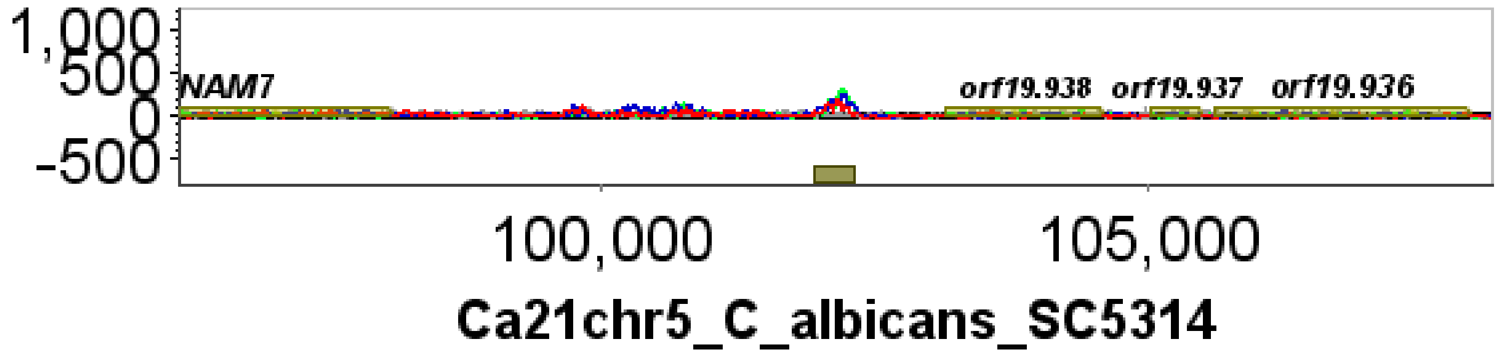

[6.34] Ca21chr1_C_albicans_SC5314:143542-155541 [+] [orf19.6027, ERG2, orf19.6025]

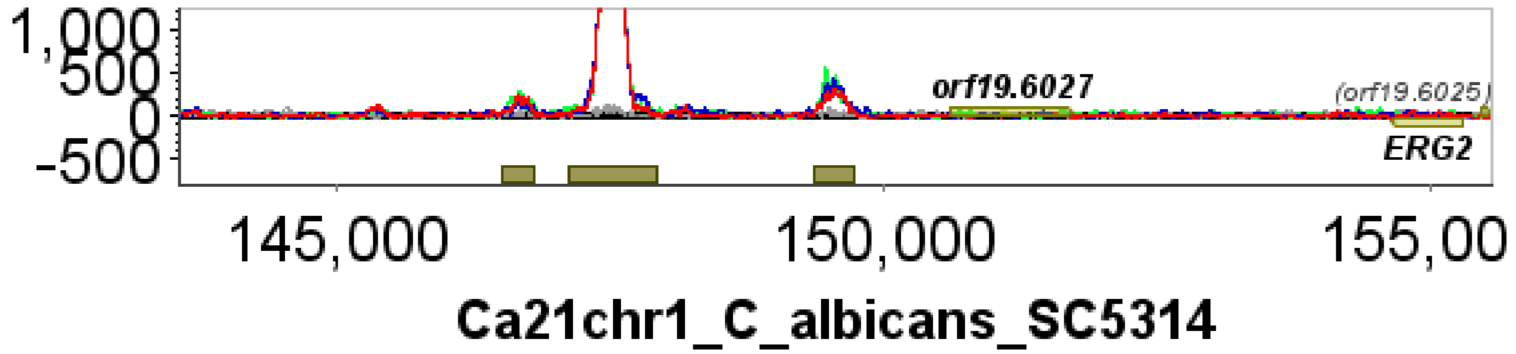

[6.33] Ca21chr4_C_albicans_SC5314:570277-582276 [+] [HIT1, CGR1, orf19.2721, orf19.2724, orf19.2720]

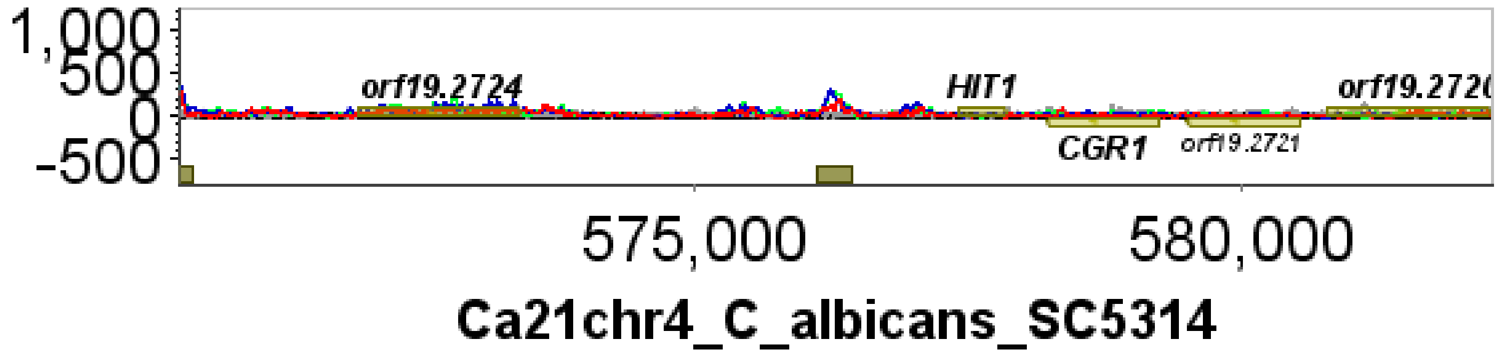

[6.24] Ca21chrR_C_albicans_SC5314:867779-879778 [+] [WOR3, IFF9]

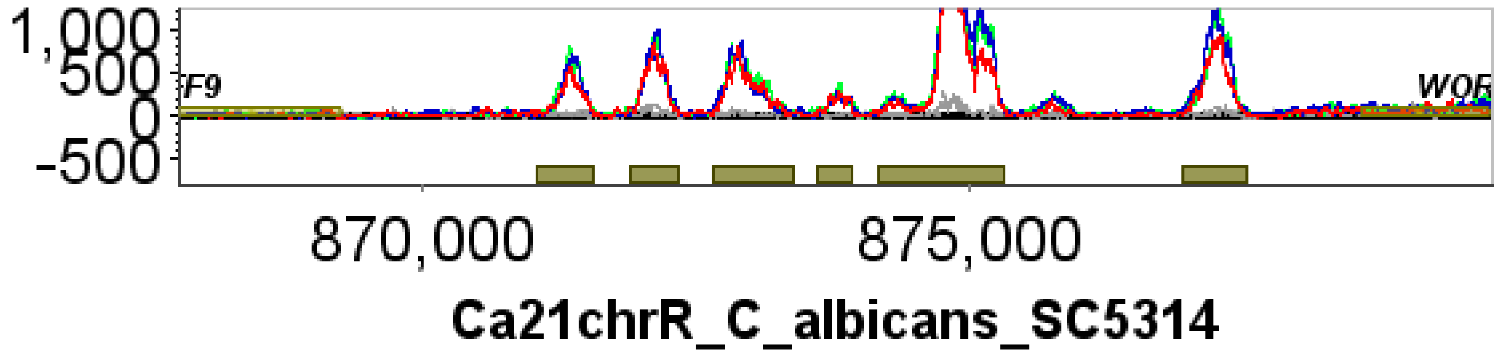

[6.24] Ca21chr1_C_albicans_SC5314:288042-300041 [+] [orf19.3336, orf19.3335, RPS21, orf19.3337, orf19.3333]

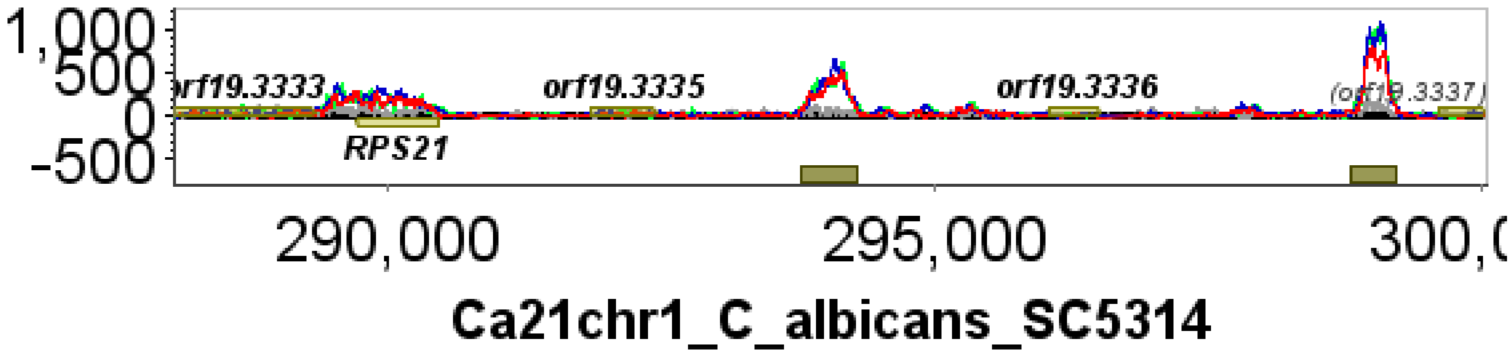

[6.23] Ca21chr3_C_albicans_SC5314:1216946-1228945 [+] [orf19.6984, orf19.6983]

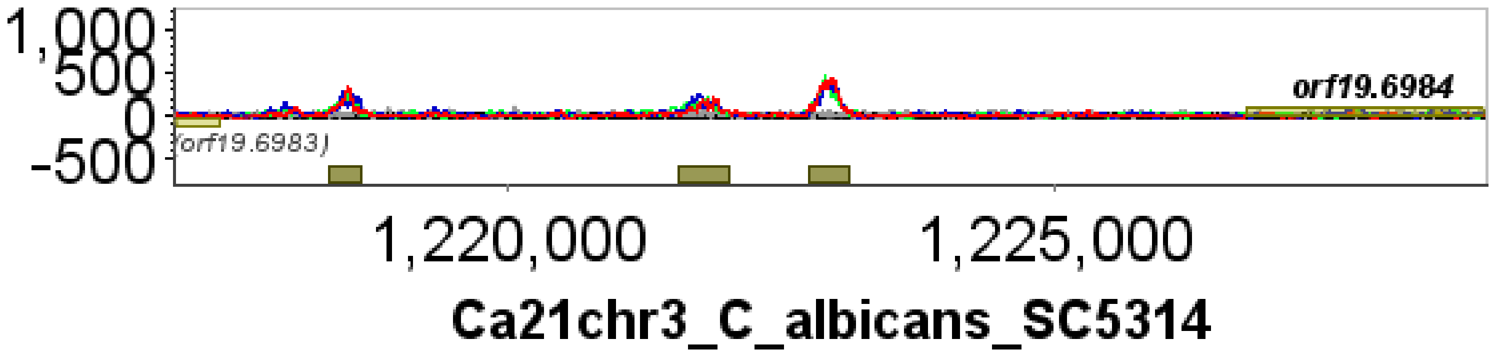

[6.21] Ca21chr1_C_albicans_SC5314:566386-578385 [+] [orf19.2962, orf19.2963, orf19.2961, FRS2, orf19.2964]

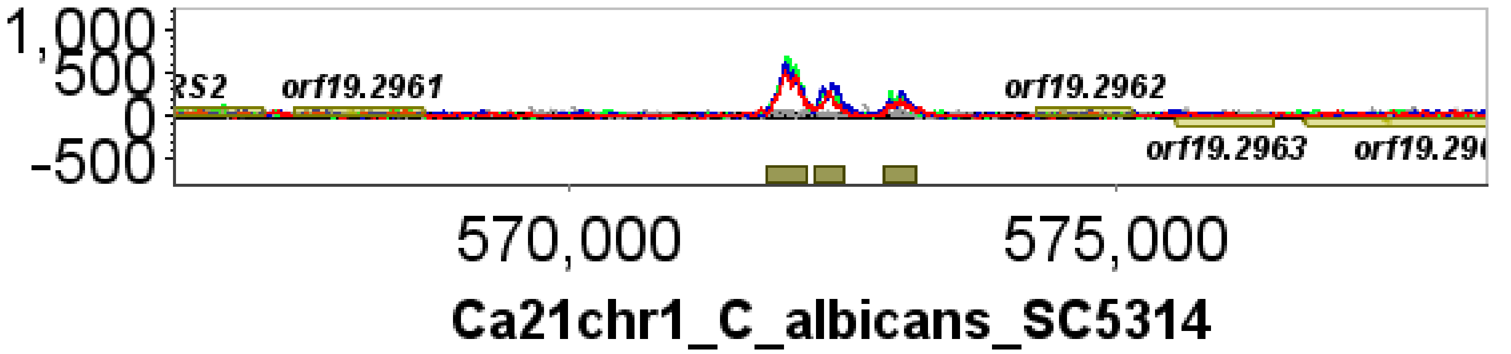

[6.21] Ca21chr1_C_albicans_SC5314:418664-430663 [+] [PHO91, orf19.3663.1, HSP31, orf19.3661, SET6]

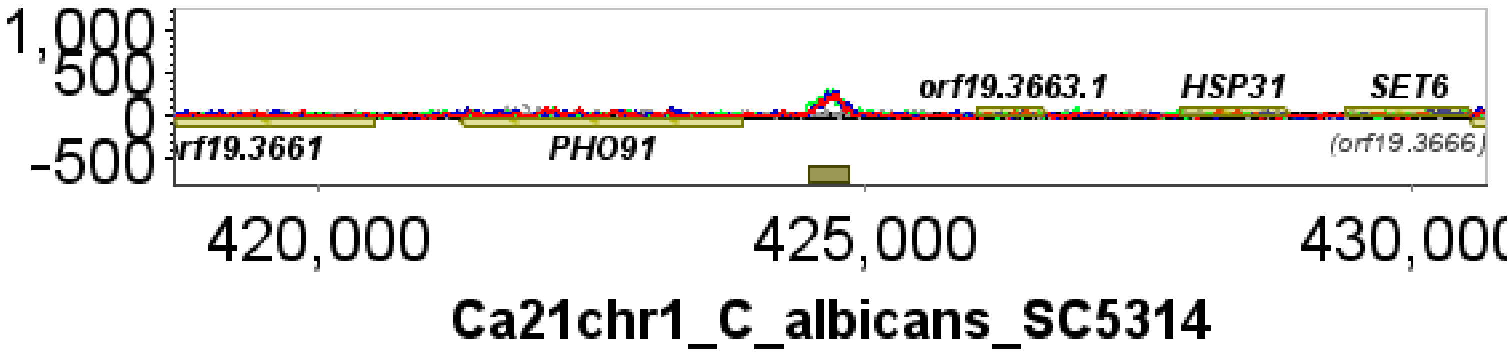

[6.2] Ca21chr1_C_albicans_SC5314:2095237-2107236 [+] [orf19.4818, orf19.4819, RAM2, snR31a, orf19.4816]

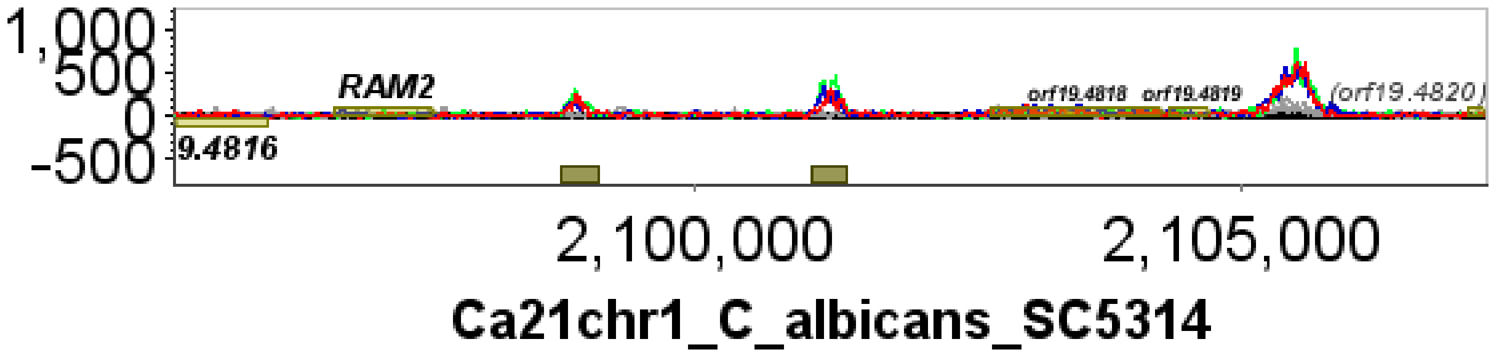

[6.18] Ca21chr2_C_albicans_SC5314:447464-459463 [+] [OPI1, orf19.1544, HEX3, orf19.1545, orf19.1546]

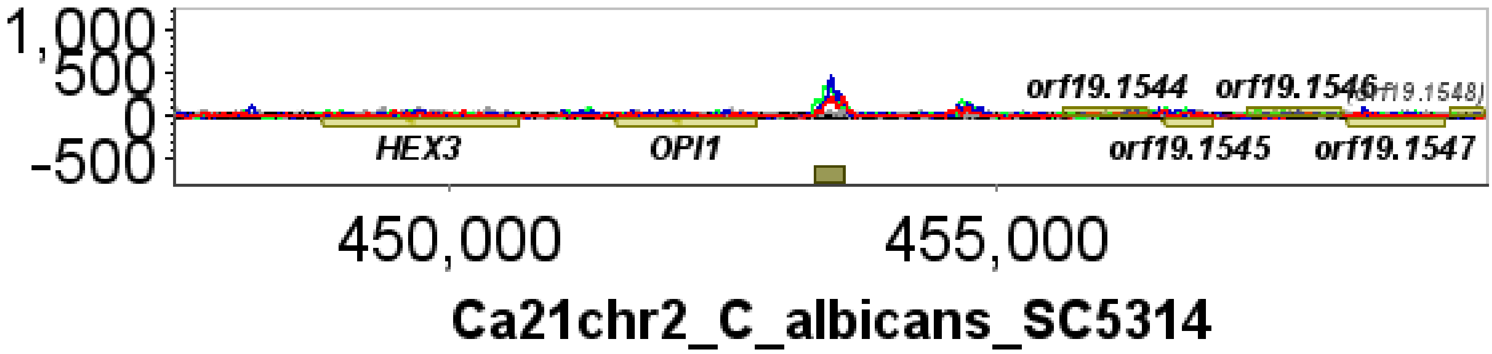

[6.18] Ca21chr1_C_albicans_SC5314:2515889-2527888 [+] [PHO84, DPP1, BTA1]

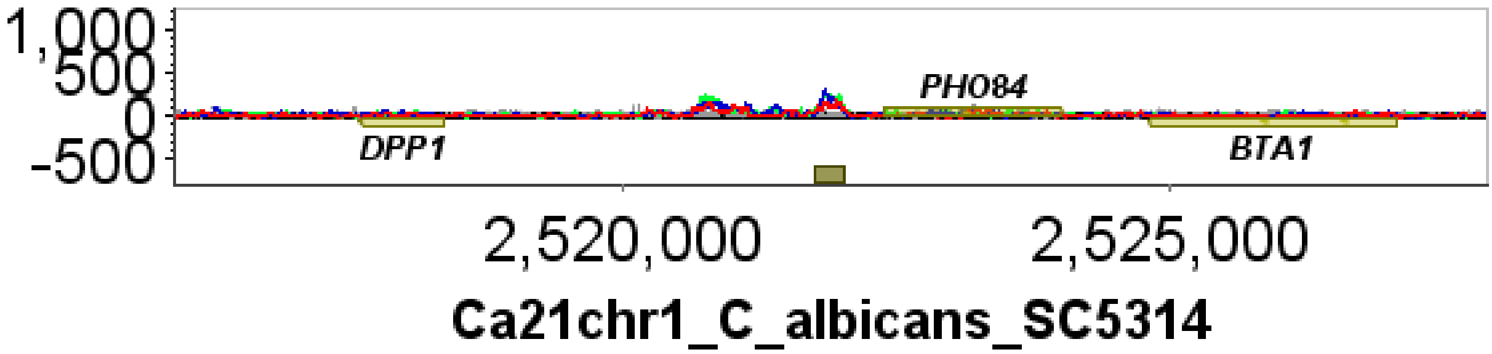

[6.17] Ca21chrR_C_albicans_SC5314:584139-596138 [+] [orf19.2822, orf19.2821]

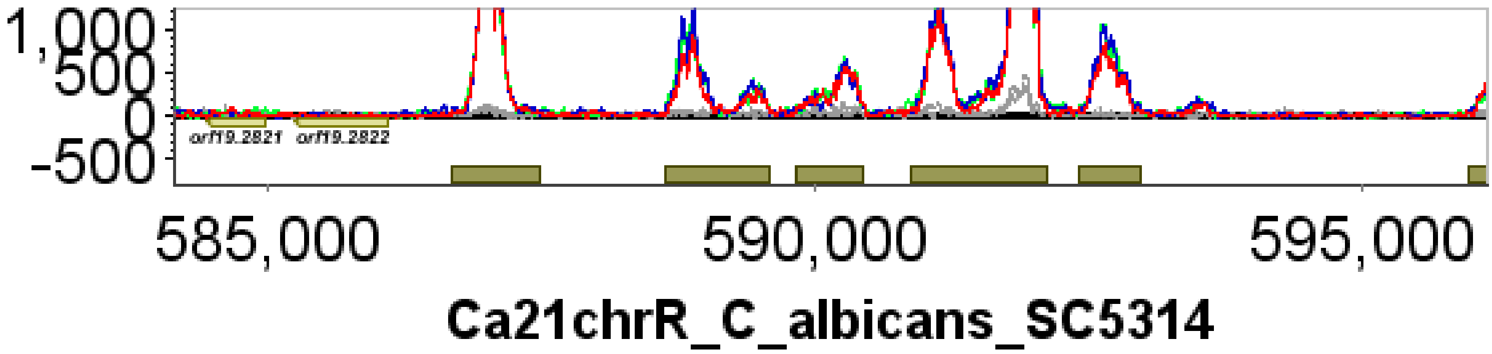

[6.08] Ca21chrR_C_albicans_SC5314:1190412-1202411 [+] [SRR1, MTG2, DBP8, tC(GCA)1, tC(GCA)2]

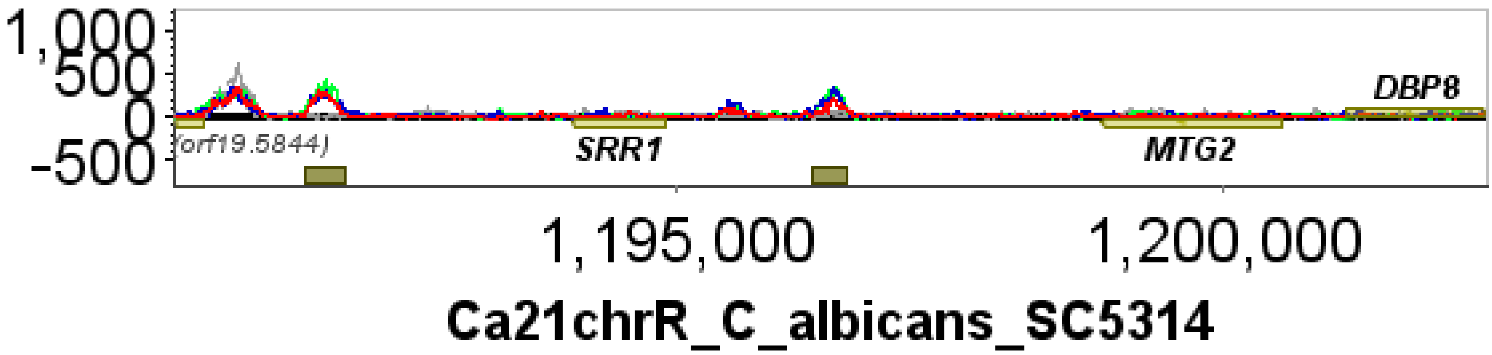

[6.08] Ca21chr3_C_albicans_SC5314:1666808-1678807 [+] [orf19.6769, orf19.6770, NOP13, UBI4, ECM29]

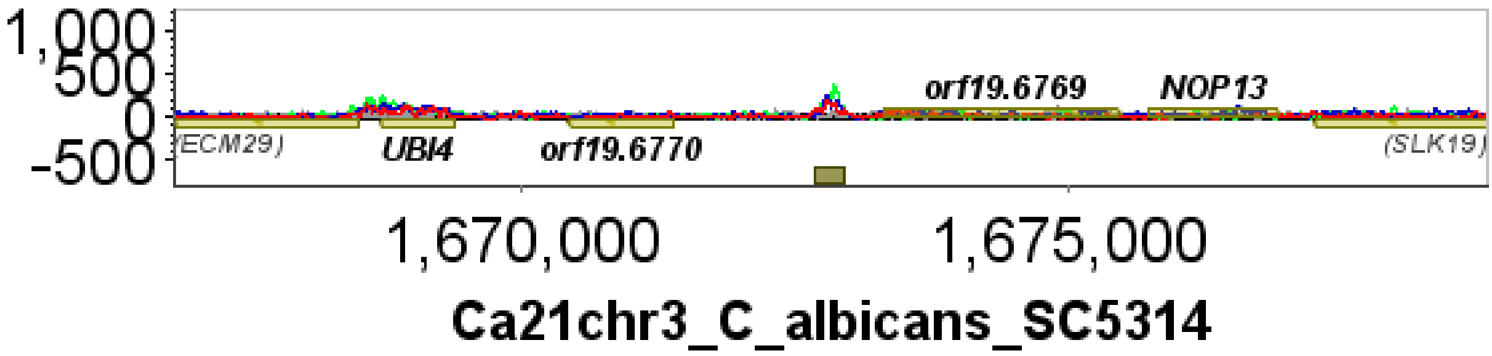

[6.08] Ca21chr1_C_albicans_SC5314:298128-310127 [+] [SOD2, orf19.3341, orf19.3337, orf19.3342]

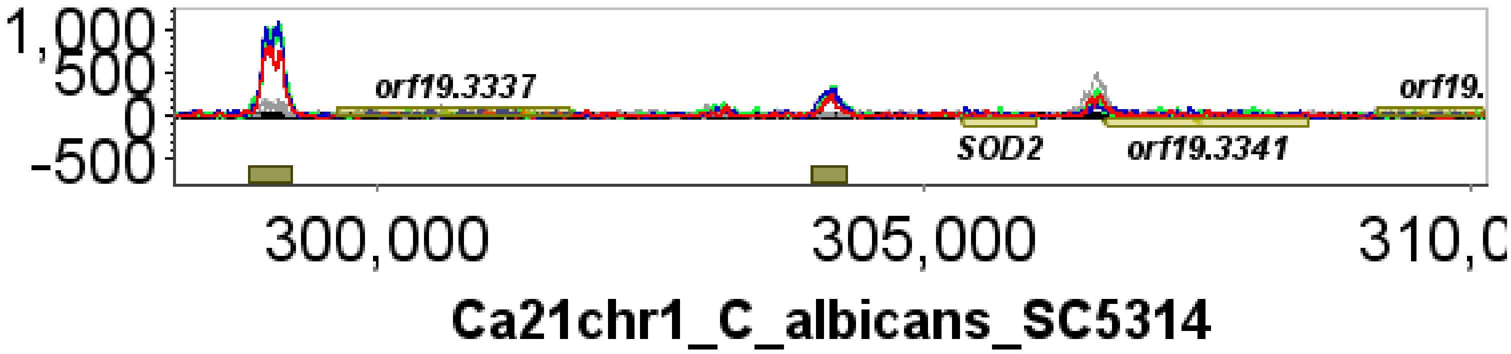

[6.04] Ca21chr5_C_albicans_SC5314:203391-215390 [+] [GIT2, URA4, TRX2, orf19.1975, TFS1]

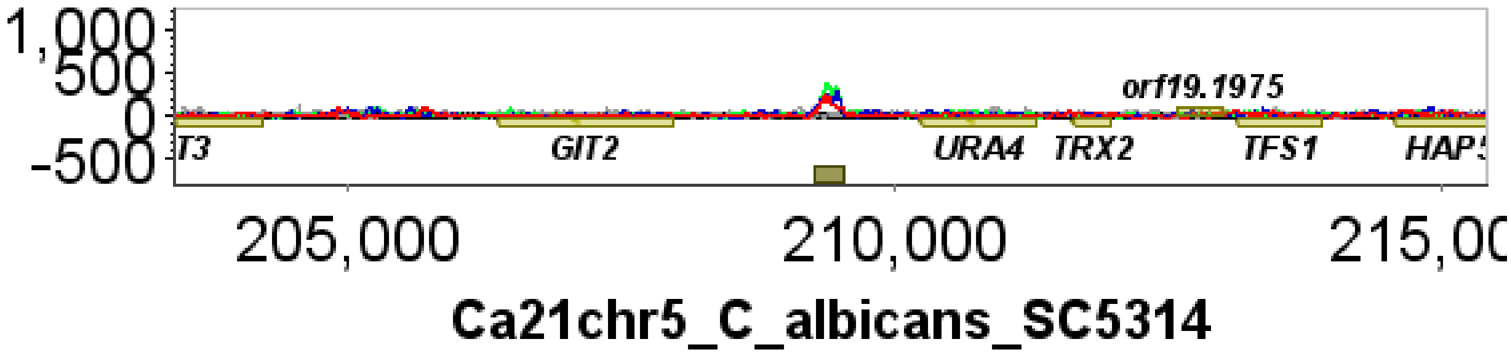

[6.04] Ca21chr1_C_albicans_SC5314:2934864-2946863 [+] [orf19.4972, HYR1]

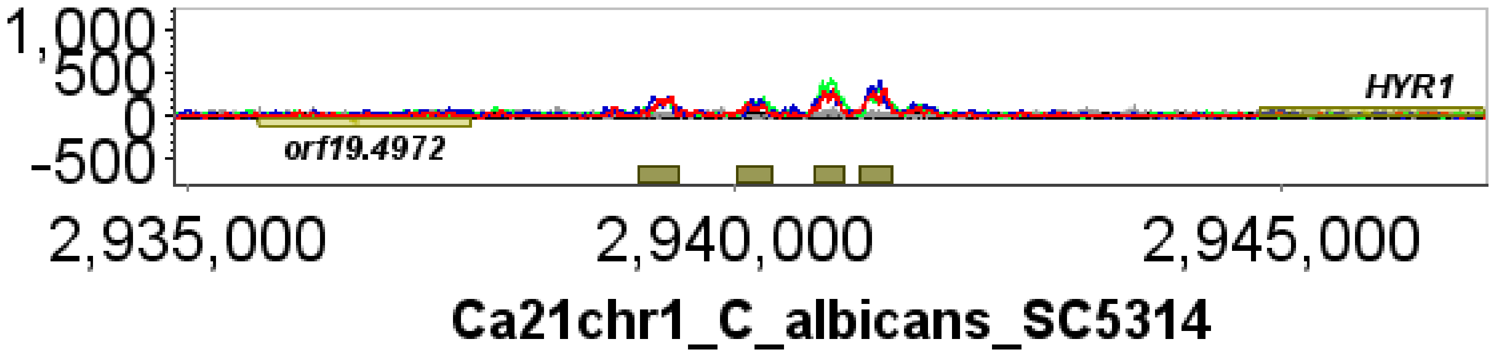

[6.03] Ca21chr5_C_albicans_SC5314:1111401-1123400 [+] [GRF10, orf19.4001, DUN1, orf19.3999]

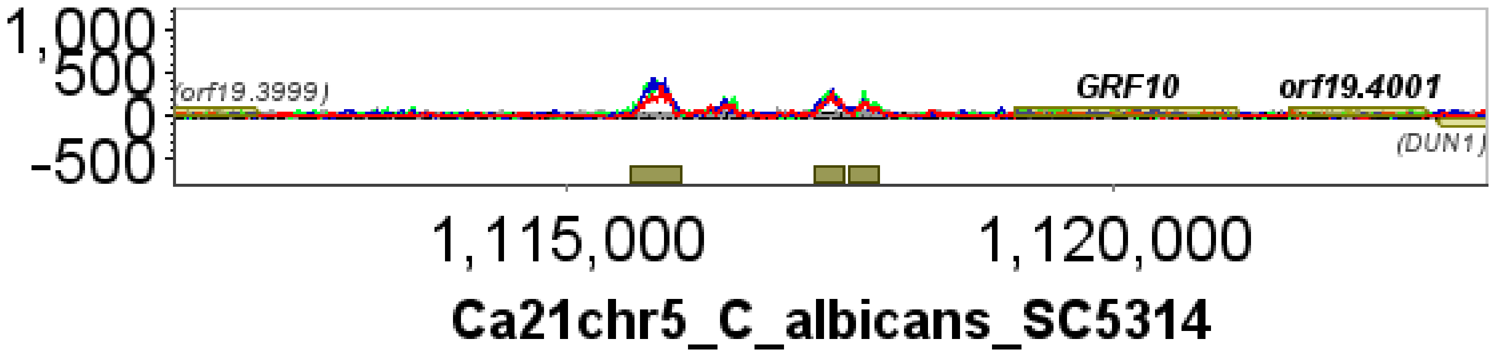

[6.03] Ca21chr4_C_albicans_SC5314:271463-283462 [+] [PGA53, orf19.4652, ILV6, orf19.4653, orf19.4654]

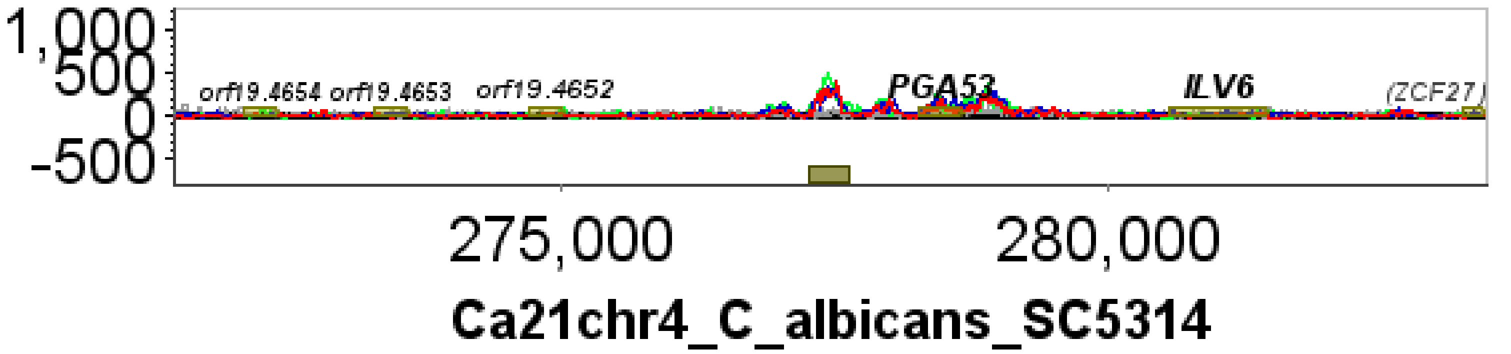

[6.0] Ca21chrR_C_albicans_SC5314:1306533-1318532 [+] [orf19.3868, RPL7, orf19.3869]

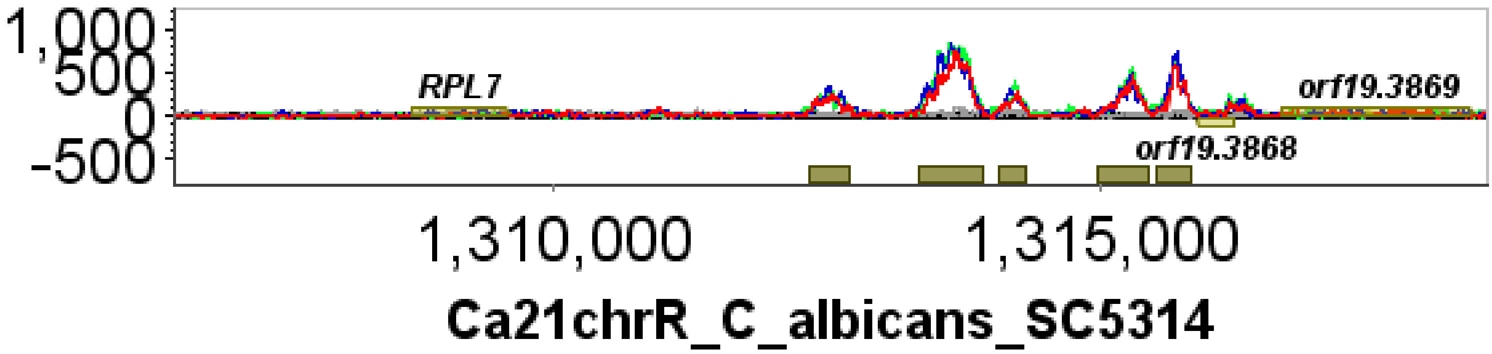

[6.0] Ca21chr3_C_albicans_SC5314:946922-958921 [+] [TEC1, orf19.5910]

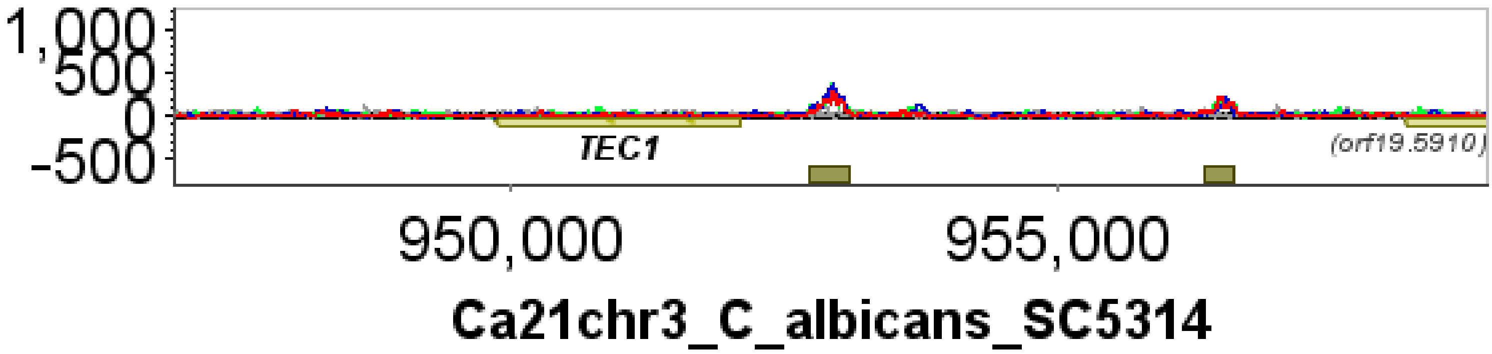

[5.98] Ca21chr1_C_albicans_SC5314:1669832-1681831 [+] [VRG4, orf19.1229, ADE4]

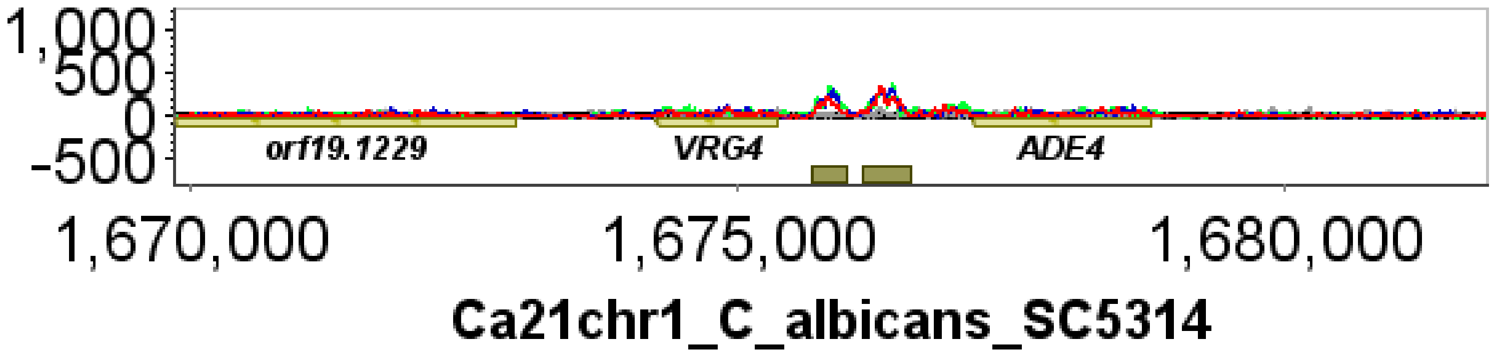

[5.93] Ca21chr5_C_albicans_SC5314:587870-599869 [+] [orf19.4280, MNN1, IFH1, orf19.4278]

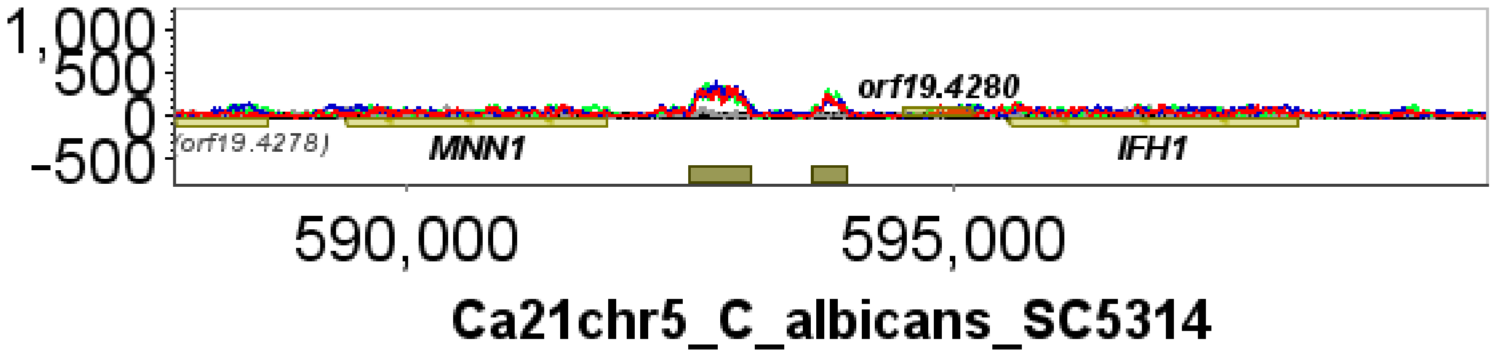

[5.93] Ca21chr5_C_albicans_SC5314:107954-119953 [+] [AGA1]

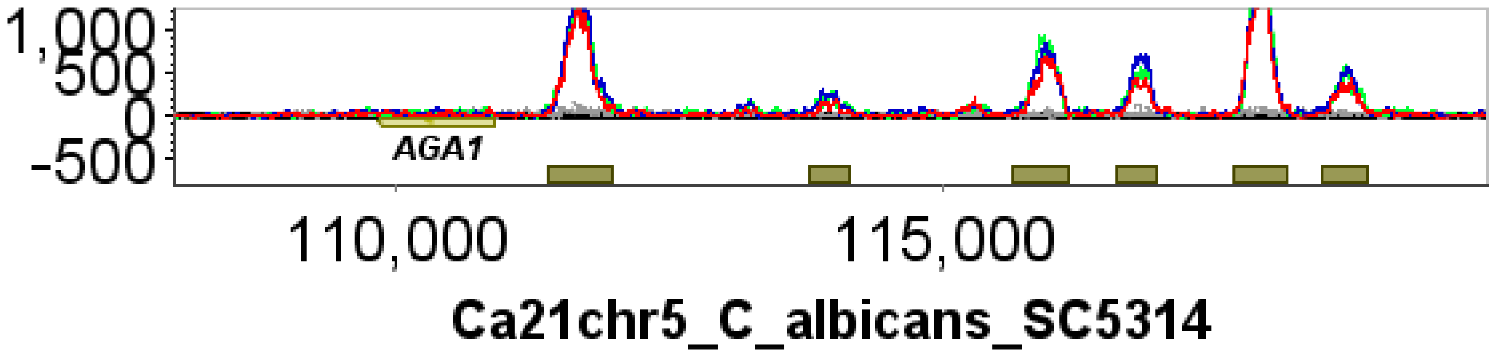

[5.86] Ca21chr7_C_albicans_SC5314:912005-924004 [+] [NRG1, orf19.7151]

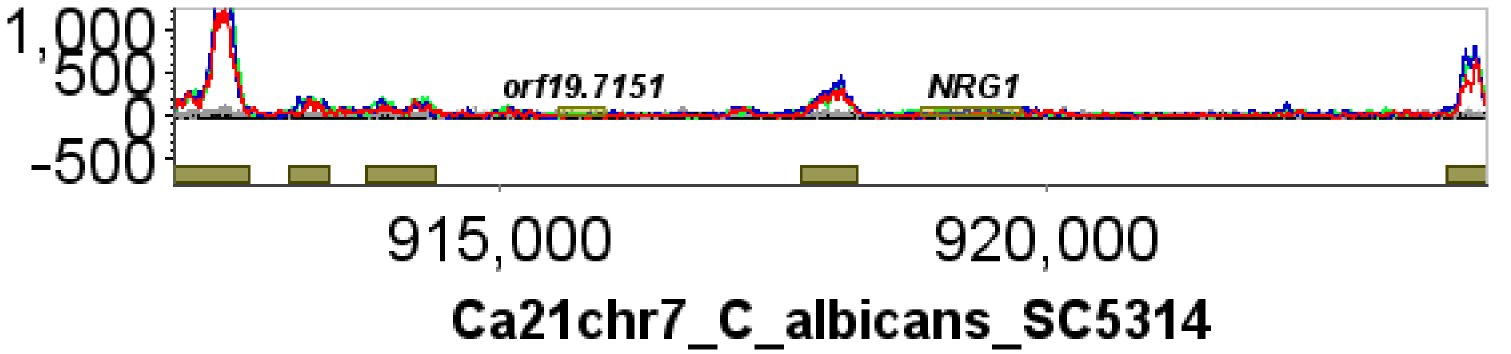

[5.86] Ca21chr6_C_albicans_SC5314:142078-154077 [+] [orf19.3643, orf19.3644]

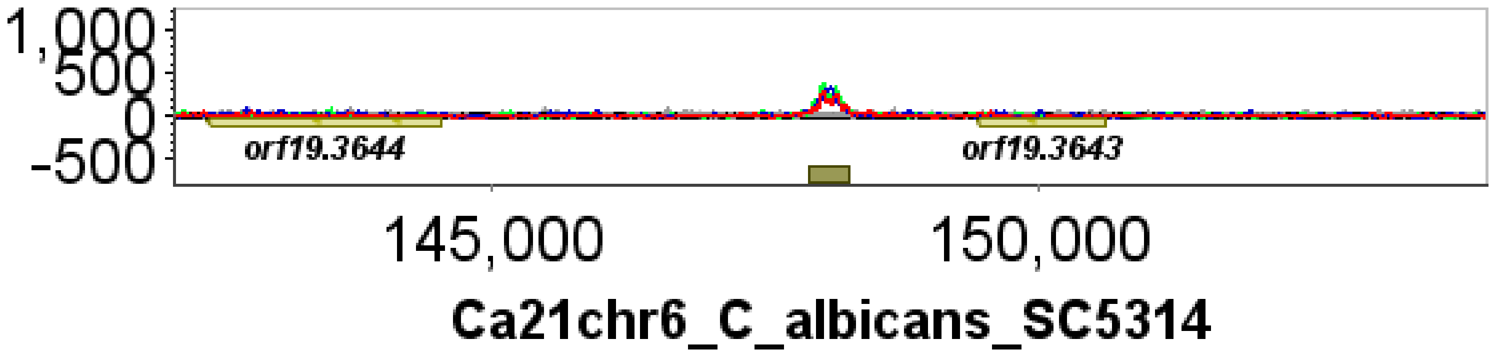

[5.86] Ca21chr4_C_albicans_SC5314:936386-948385 [+] [orf19.1409.3, LYS4, FGR3, orf19.1409.1]

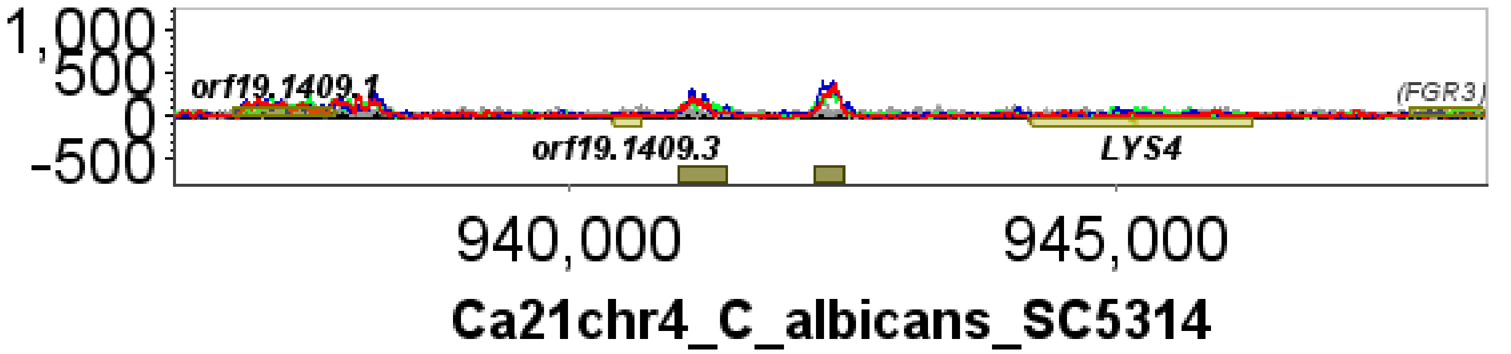

[5.85] Ca21chrR_C_albicans_SC5314:774011-786010 [+] [orf19.4394, CIT1, orf19.4395, orf19.4396, DEM1]

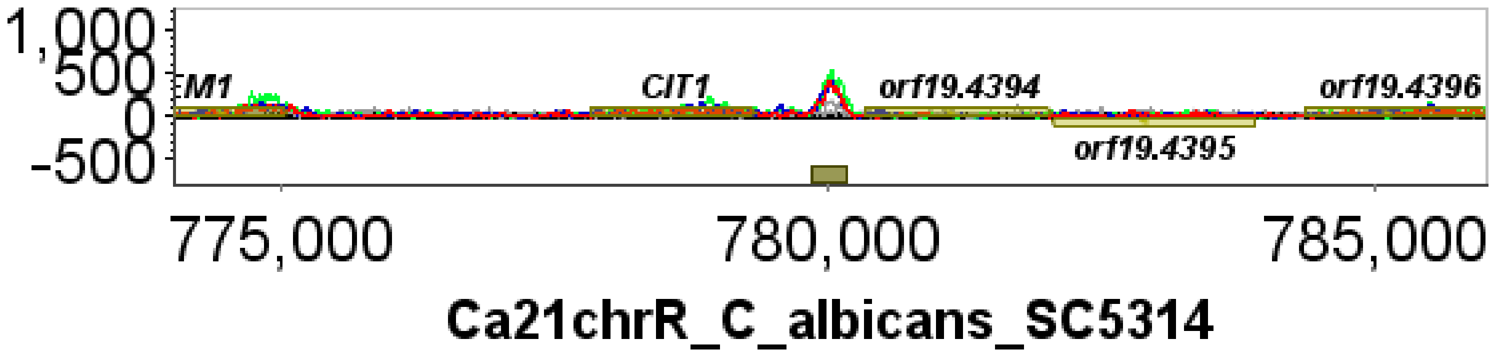

[5.85] Ca21chrR_C_albicans_SC5314:438817-450816 [+] [OPT1, orf19.2604, PRK1, HDA1]

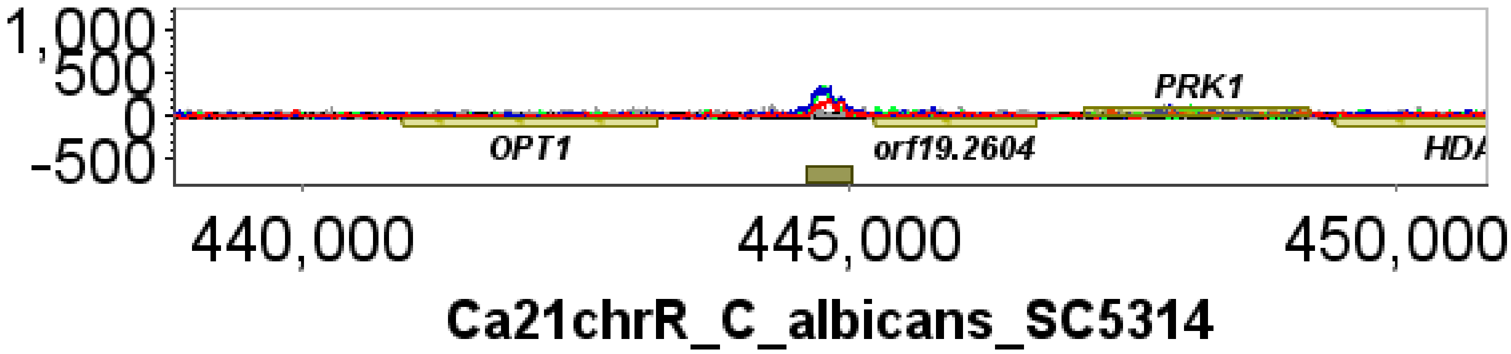

[5.84] Ca21chr3_C_albicans_SC5314:978657-990656 [+] [ZCF31, orf19.5921, orf19.5925, orf19.5920, ARG11]

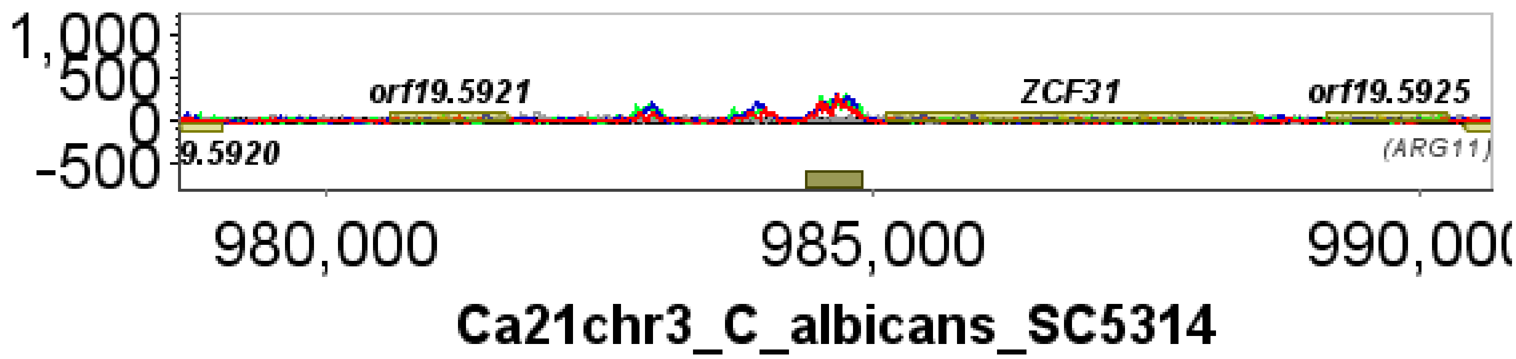

[5.83] Ca21chr1_C_albicans_SC5314:2216708-2228707 [+] [orf19.4883, snR10a, orf19.4882]

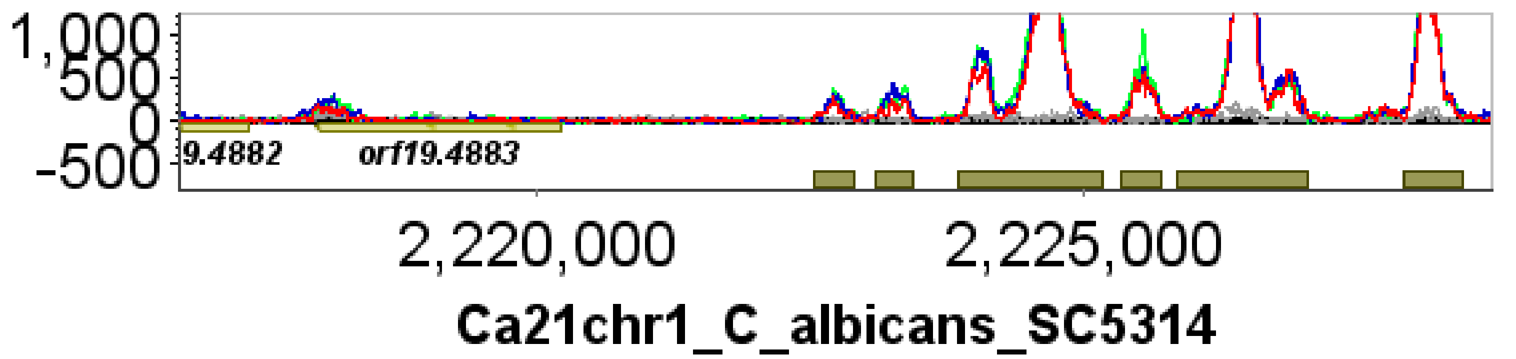

[5.79] Ca21chr2_C_albicans_SC5314:1863657-1875656 [+] [IFF6, orf19.4070, MET10]

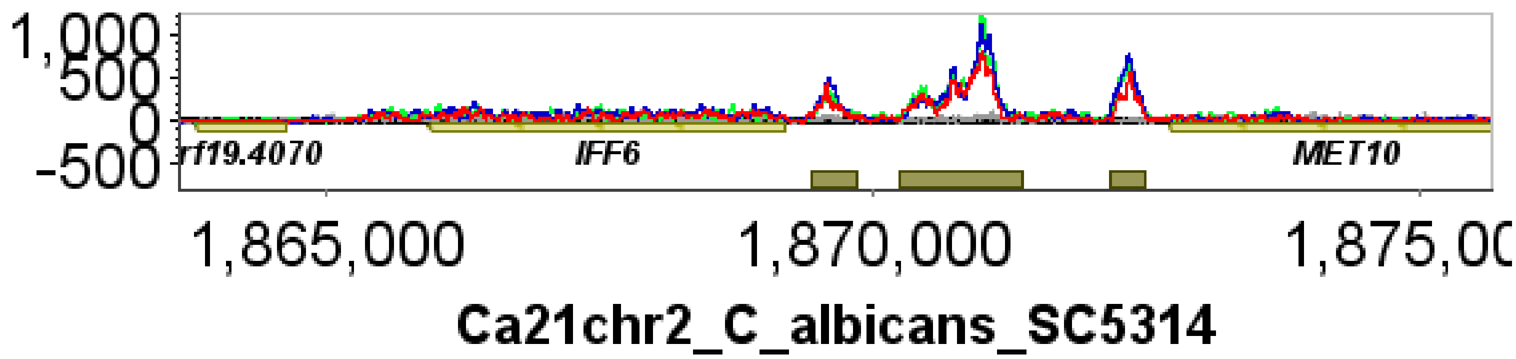

[5.71] Ca21chr3_C_albicans_SC5314:1212516-1224515 [+] [orf19.6983]

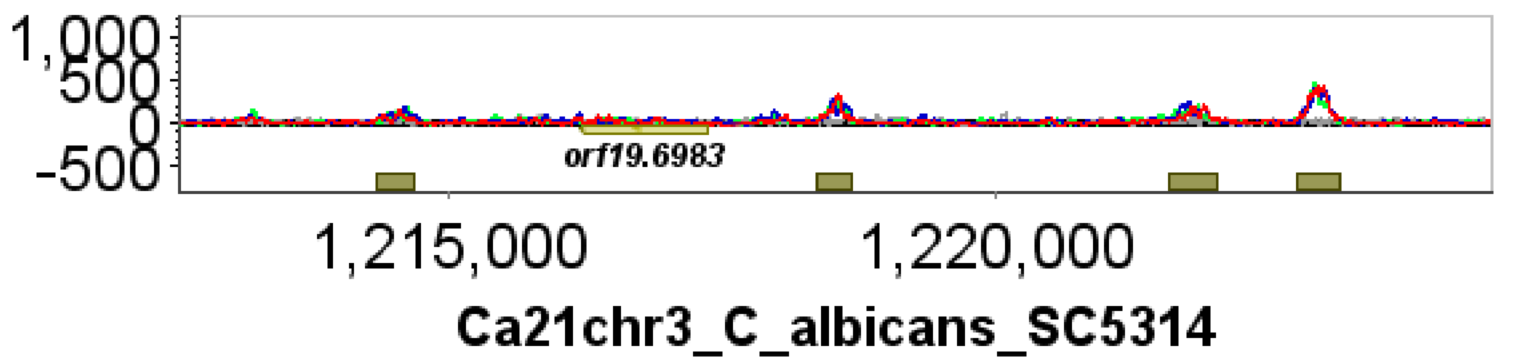

[5.68] Ca21chr1_C_albicans_SC5314:1066831-1078830 [+] [orf19.4057, orf19.4059, ARO4, orf19.4061, TRY2]

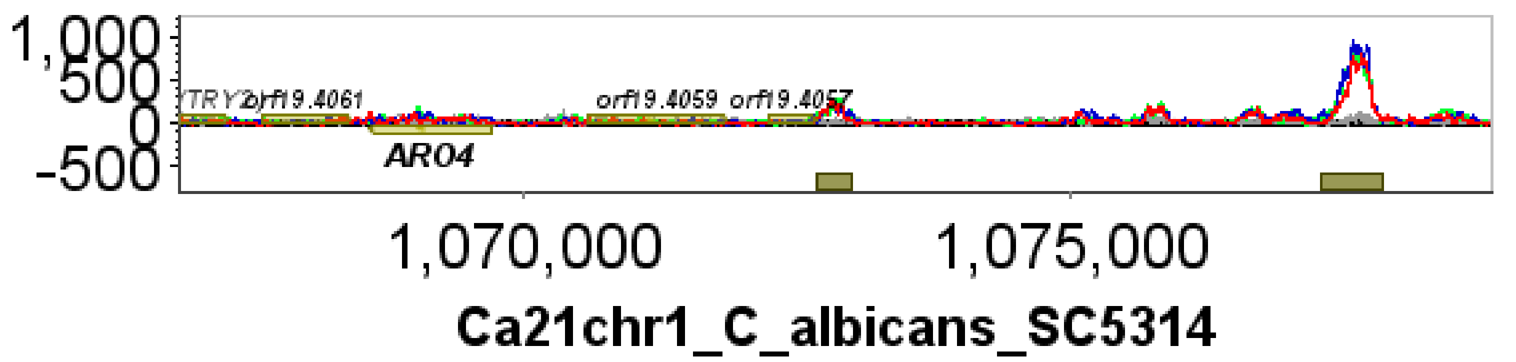

[5.61] Ca21chr1_C_albicans_SC5314:140652-152651 [+] [orf19.6027, HGC1]

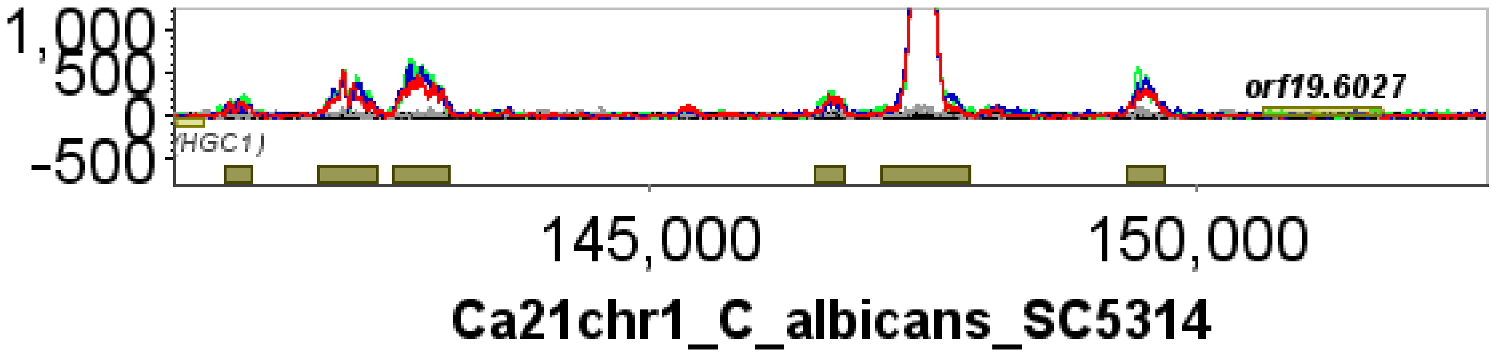

[5.61] Ca21chr5_C_albicans_SC5314:255022-267021 [+] [CYC3, orf19.1956, snR189c, snR69, orf19.1955]

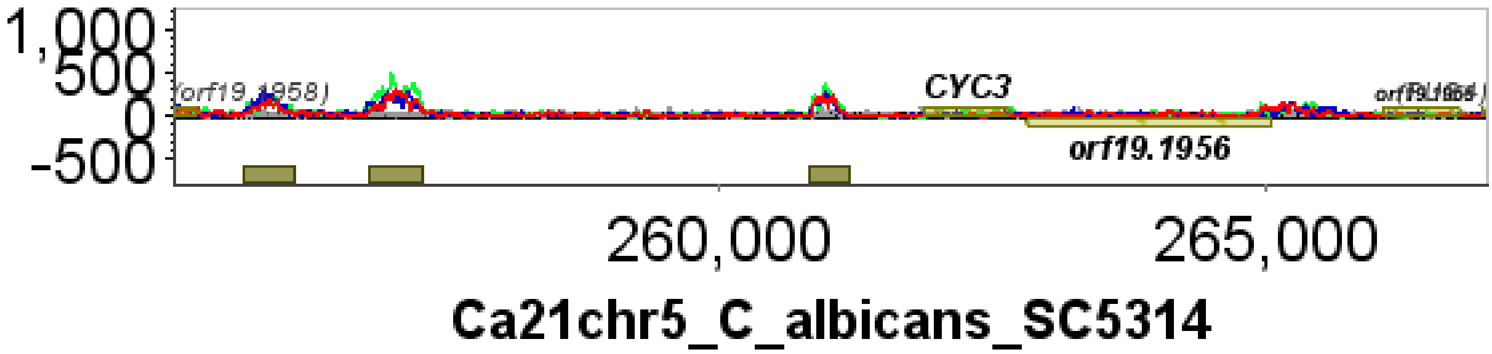

[5.49] Ca21chr5_C_albicans_SC5314:301556-313555 [+] [CFL5, CFL4, SNM1, SEF2]

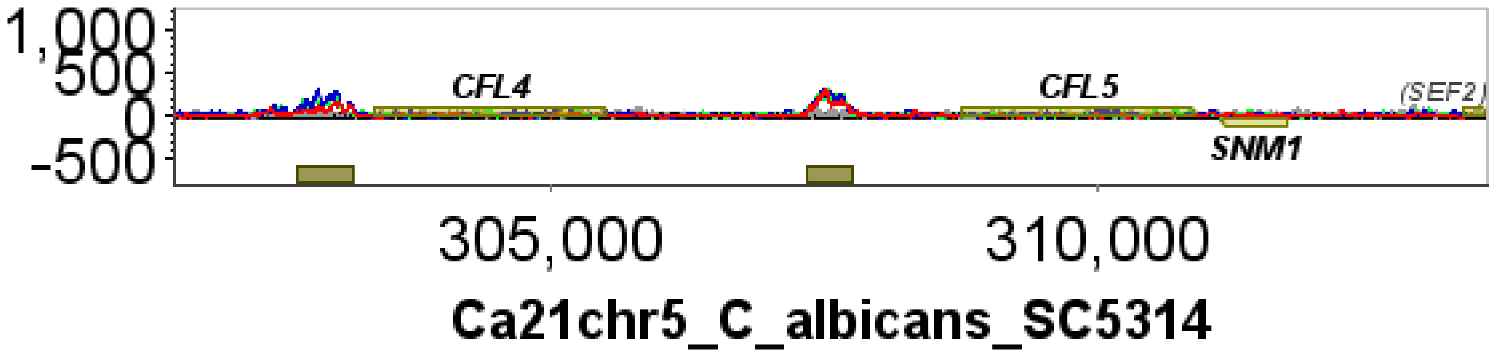

[5.48] Ca21chr1_C_albicans_SC5314:1279914-1291913 [+] [HAC1, orf19.2431, orf19.2433, NPL4, orf19.2430]

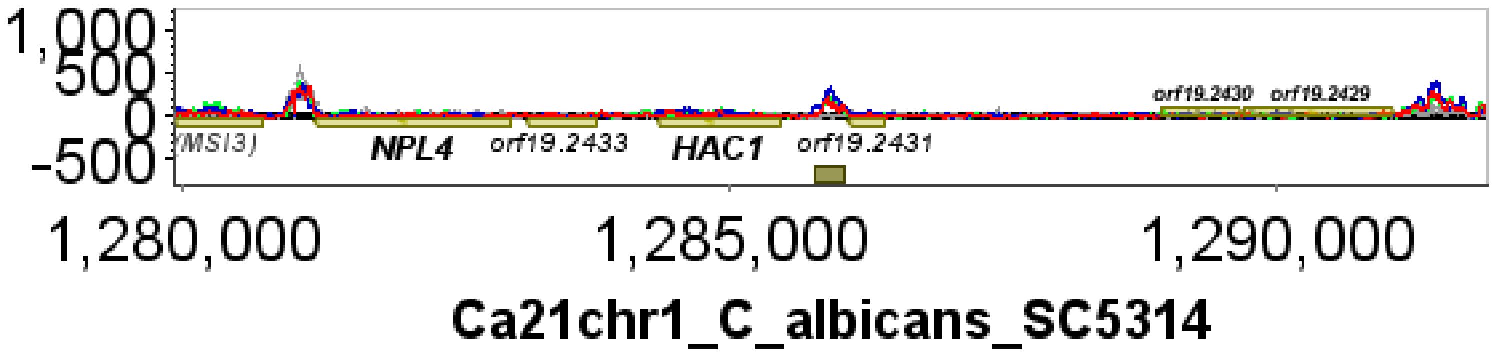

[5.46] Ca21chr5_C_albicans_SC5314:156784-168783 [+] [IFF8, SPE2, TFB3, RMT2]

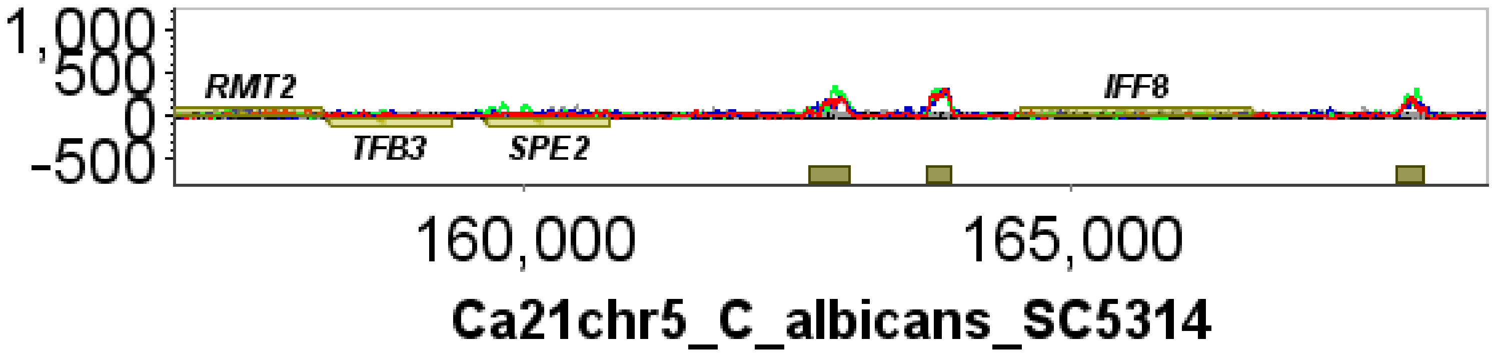

[5.45] Ca21chr6_C_albicans_SC5314:662597-674596 [+] [MDR1, BMT6, orf19.5605, orf19.5606, orf19.5601]

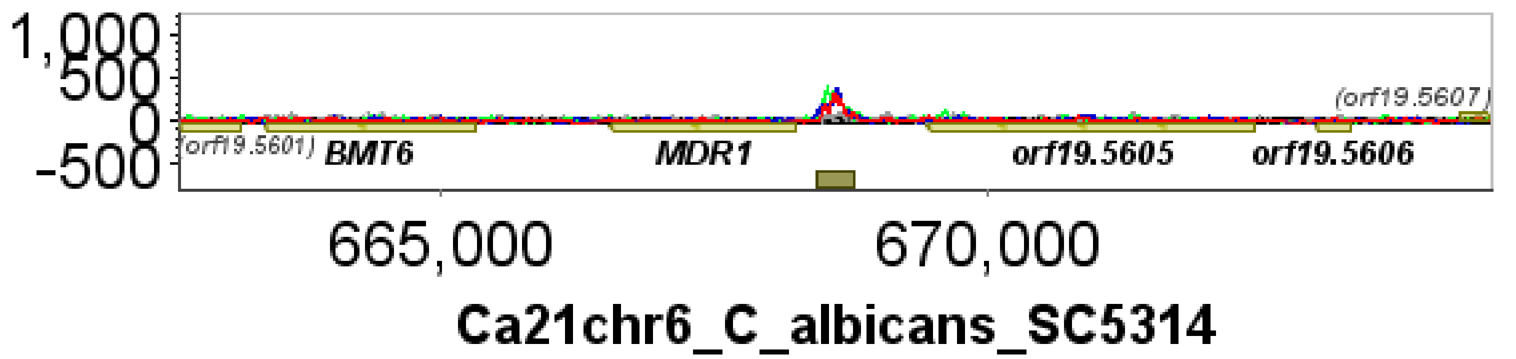

[5.43] Ca21chr5_C_albicans_SC5314:248604-260603 [+] [orf19.1958, orf19.1959]

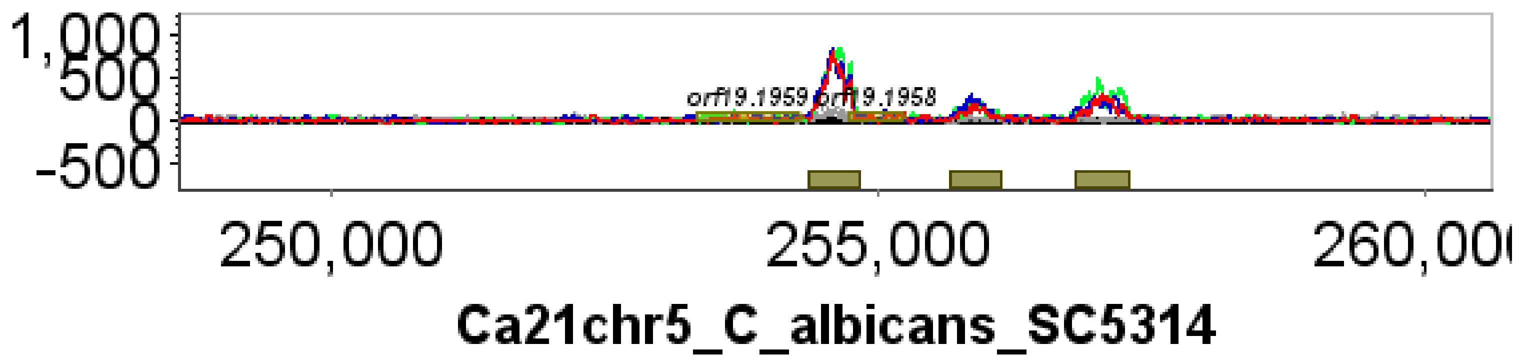

[5.41] Ca21chr6_C_albicans_SC5314:917847-929846 [+] [orf19.1075, orf19.73, SEC5, ATM1]

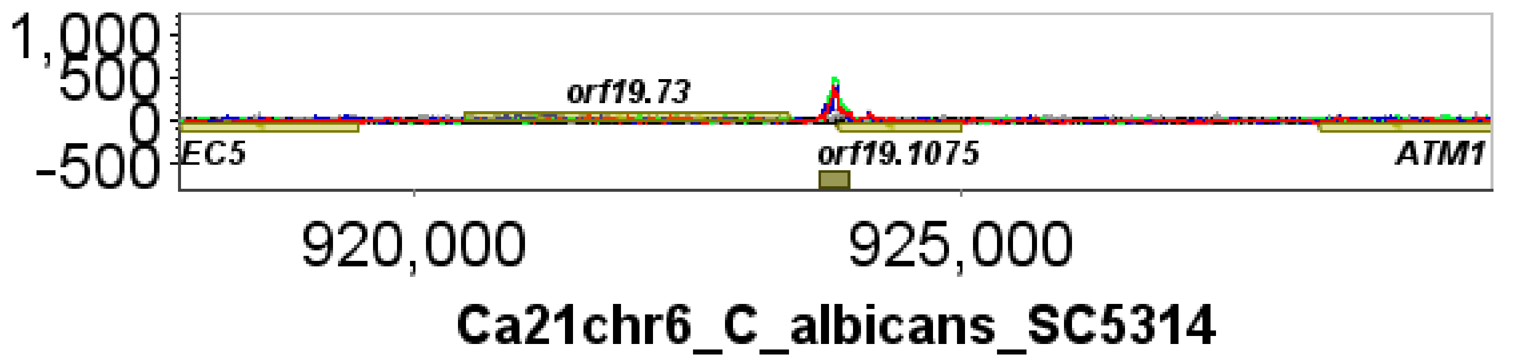

[5.39] Ca21chrR_C_albicans_SC5314:598272-610271 [+] [RFG1, DRE2]

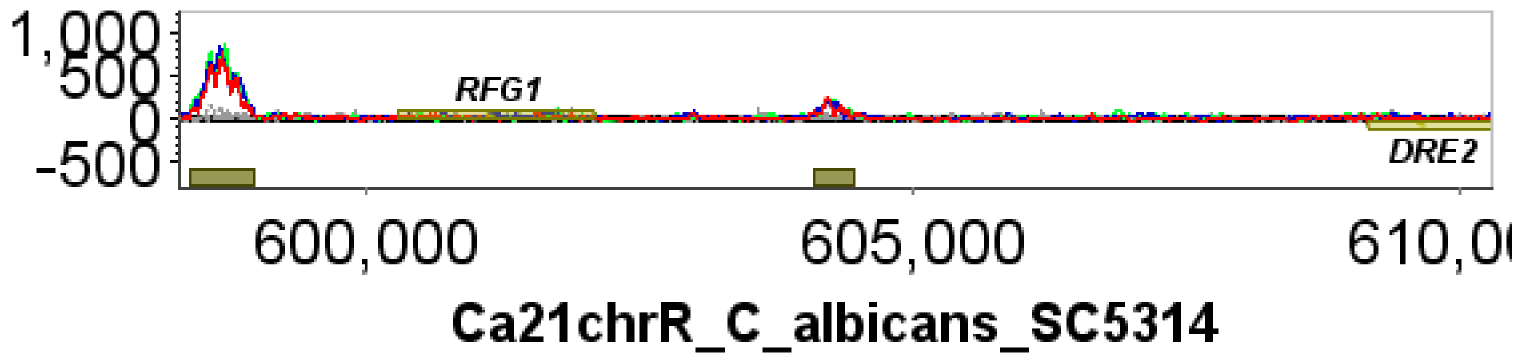

[5.39] Ca21chr7_C_albicans_SC5314:833788-845787 [+] [orf19.7204, MRP7, YAF9, orf19.7202, orf19.5502]

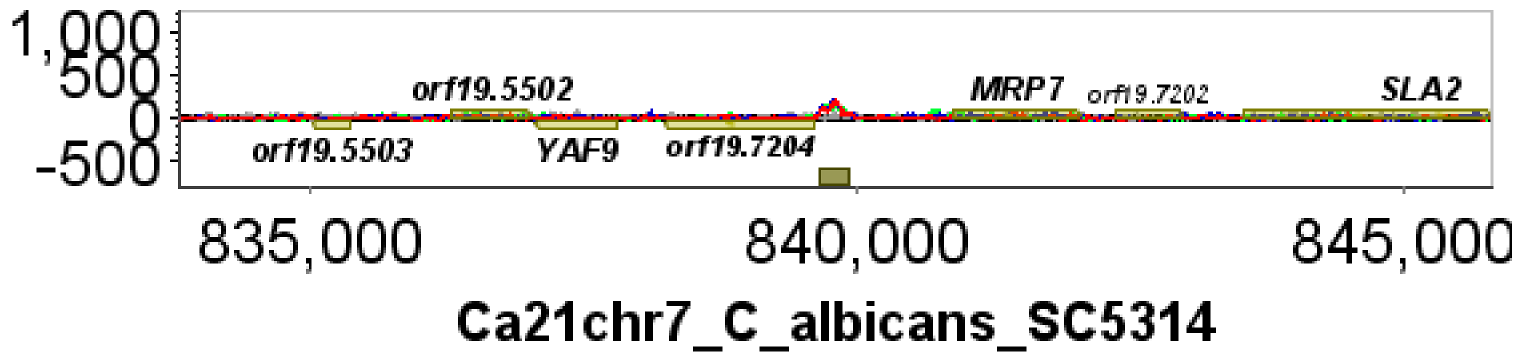

[5.38] Ca21chr5_C_albicans_SC5314:734797-746796 [+] [orf19.2653, TEF4, CAM1-1, orf19.2650.1, RMS1]

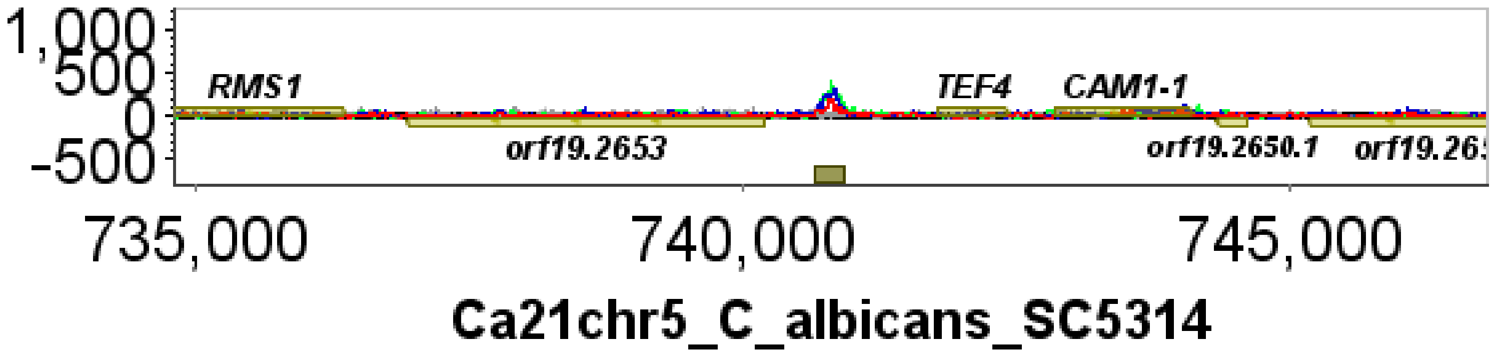

[5.38] Ca21chr5_C_albicans_SC5314:489745-501744 [+] [PTH2, orf19.4230, orf19.4229, orf19.4228, orf19.4227]

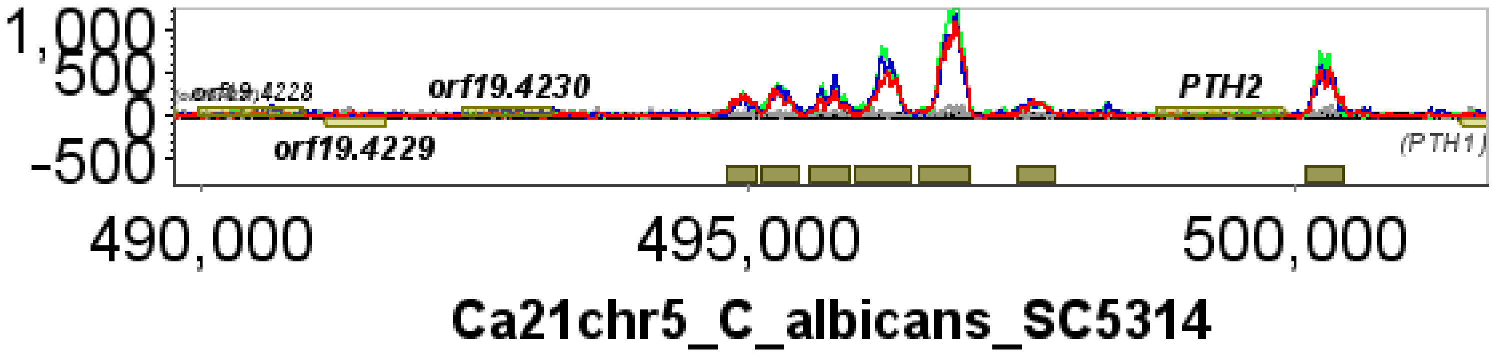

[5.37] Ca21chr5_C_albicans_SC5314:162099-174098 [+] [HYR3, IFF8]

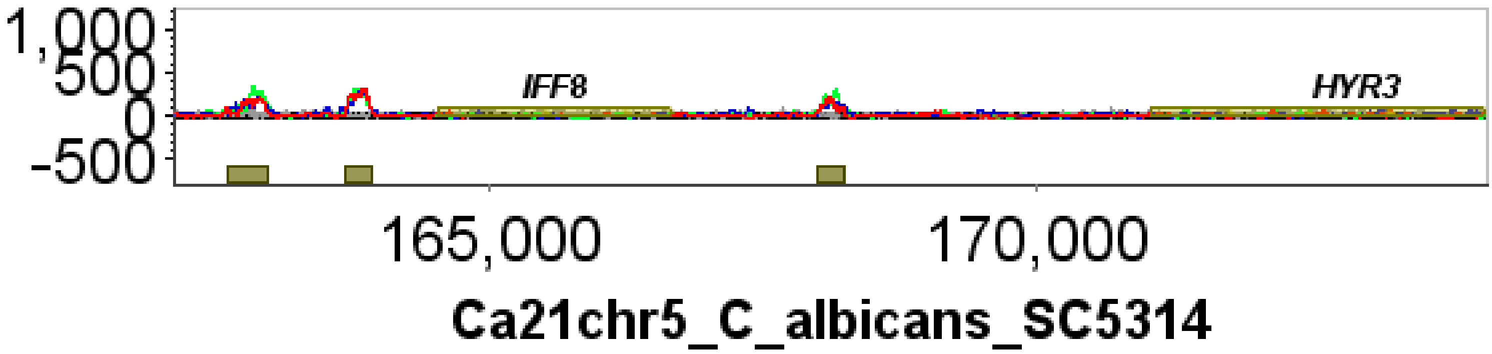

[5.37] Ca21chr3_C_albicans_SC5314:1354963-1366962 [+] [AHR1, CAM1]

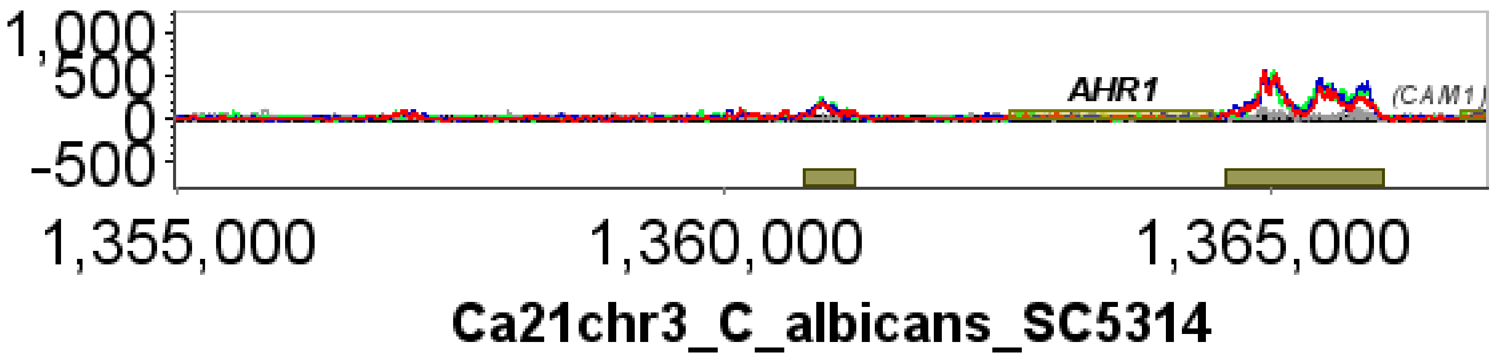

[5.37] Ca21chr1_C_albicans_SC5314:2092959-2104958 [+] [RAM2, orf19.4816, orf19.4818, orf19.4819, YTM1]

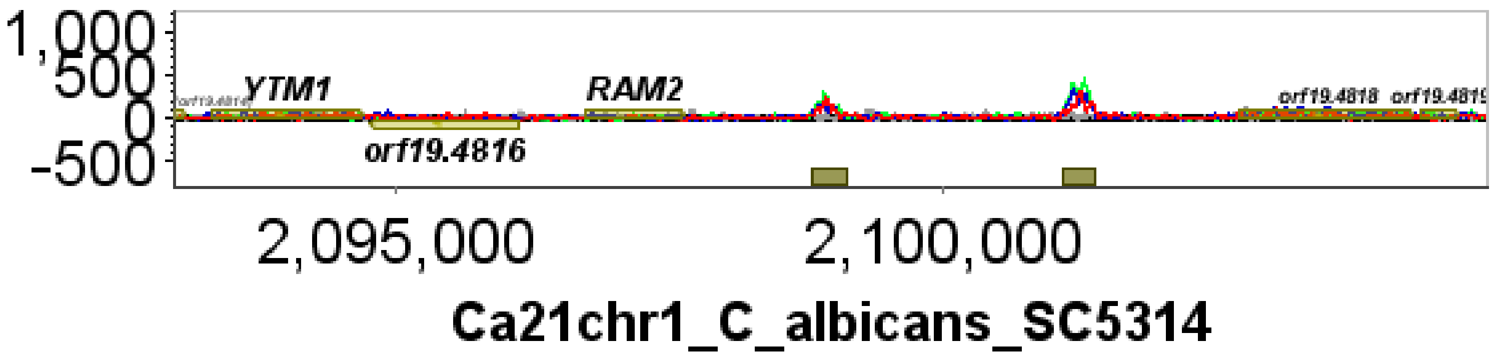

[5.32] Ca21chr5_C_albicans_SC5314:488932-500931 [+] [orf19.4230, orf19.4229, PTH2, orf19.4228, orf19.4227]

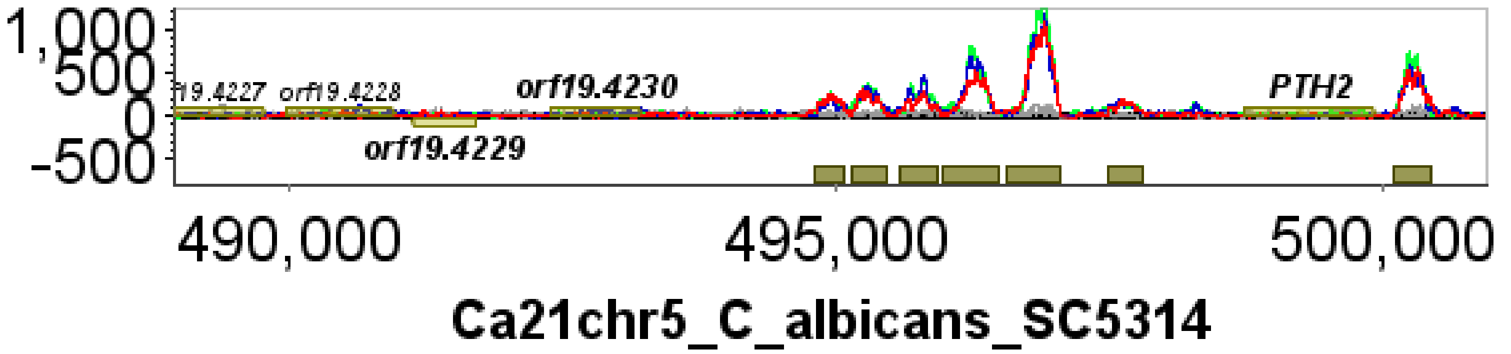

[5.29] Ca21chr5_C_albicans_SC5314:649644-661643 [+] [orf19.4321, orf19.4320, DAP2, MIG1, orf19.4323]

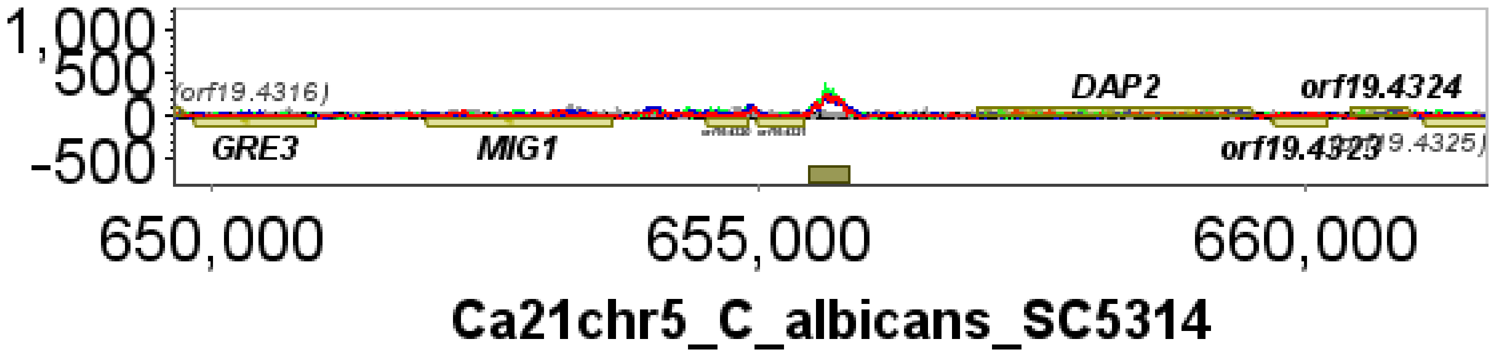

[5.28] Ca21chr5_C_albicans_SC5314:888420-900419 [+] [orf19.1286, orf19.1287, FGR42, orf19.1285]

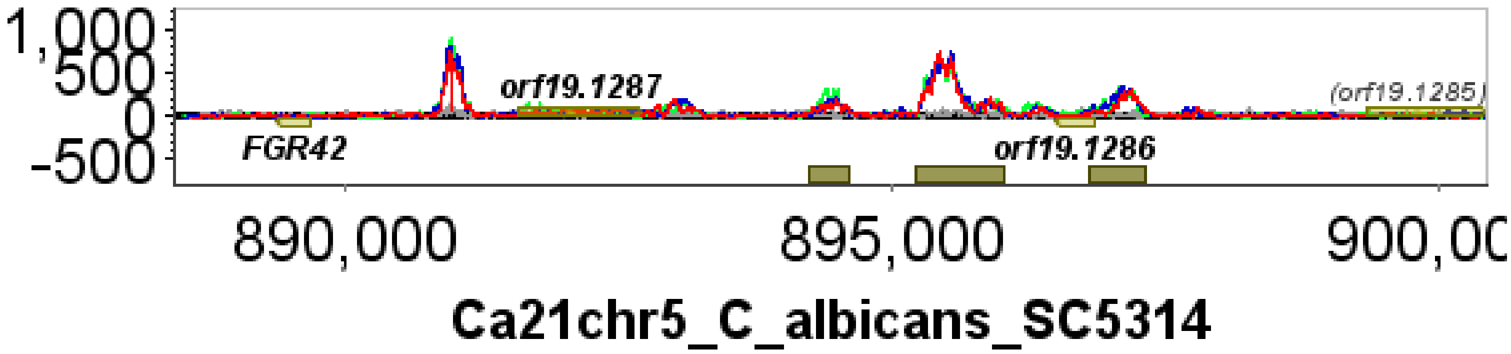

[5.24] Ca21chrR_C_albicans_SC5314:1253582-1265581 [+] [SFL1, orf19.457]

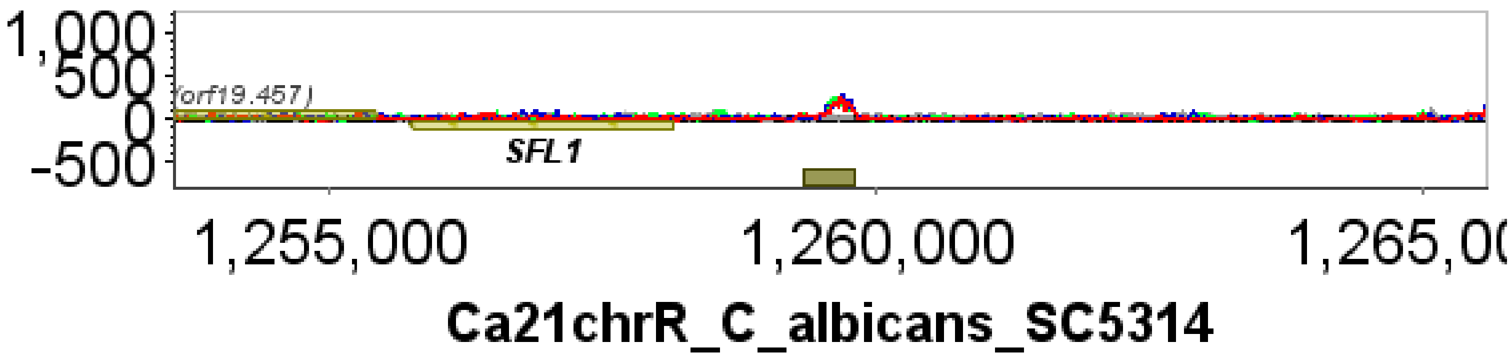

[5.23] Ca21chrR_C_albicans_SC5314:1366211-1378210 [+] [BCR1, orf19.725, orf19.721]

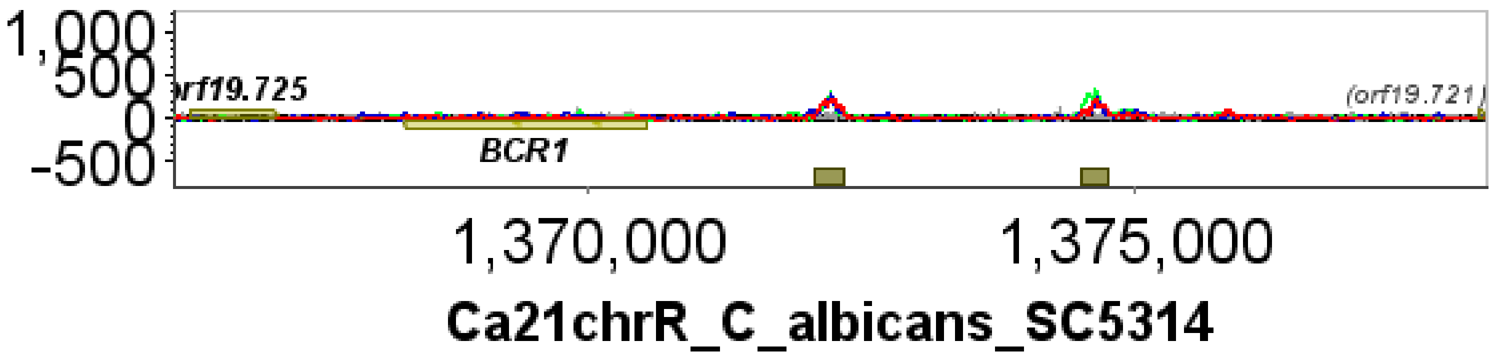

[5.22] Ca21chr3_C_albicans_SC5314:1466965-1478964 [+] [tH(GUG)3, AAF1, GLG2]

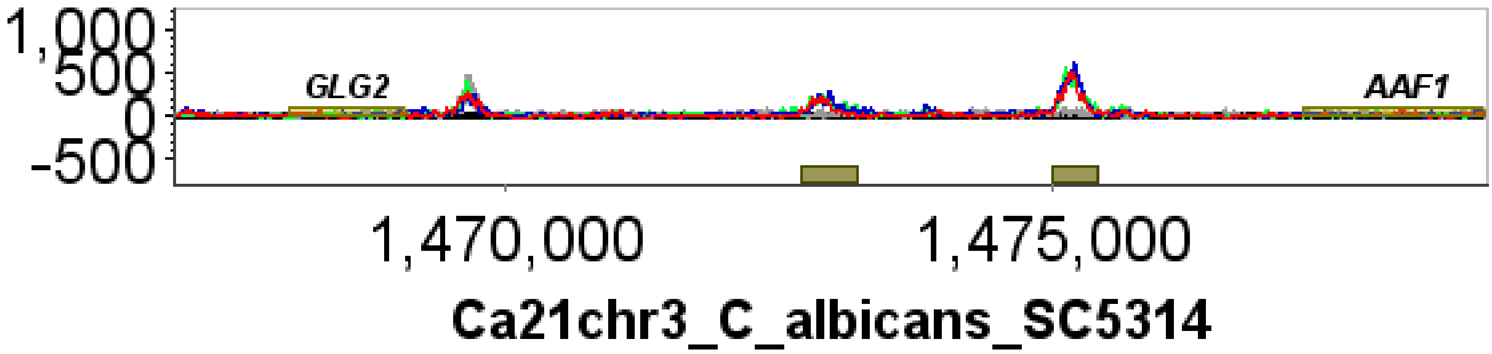

[5.18] Ca21chr2_C_albicans_SC5314:1802332-1814331 [+] [orf19.217, BUD20, orf19.216.1, orf19.216]

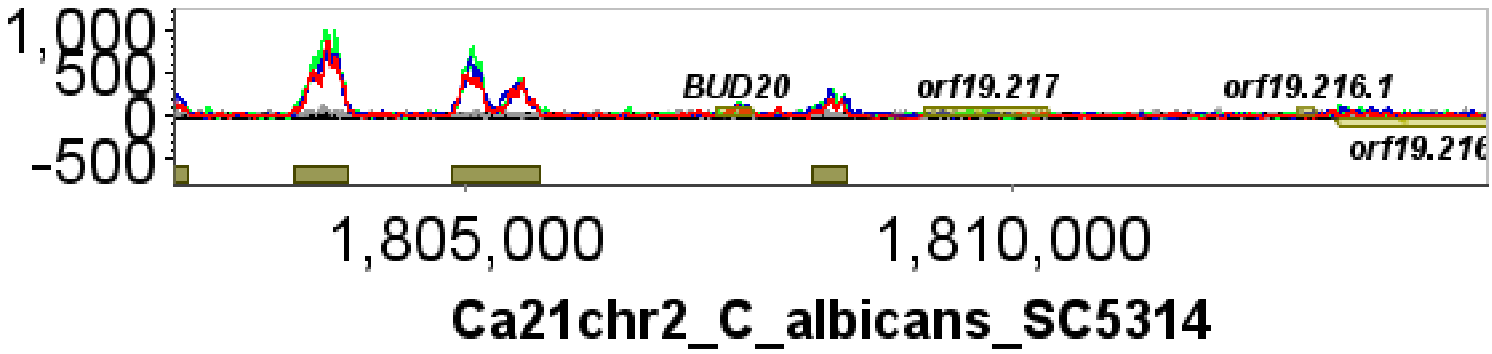

[5.17] Ca21chr6_C_albicans_SC5314:906572-918571 [+] [orf19.77.1, orf19.4553, SPB1, SEC5]

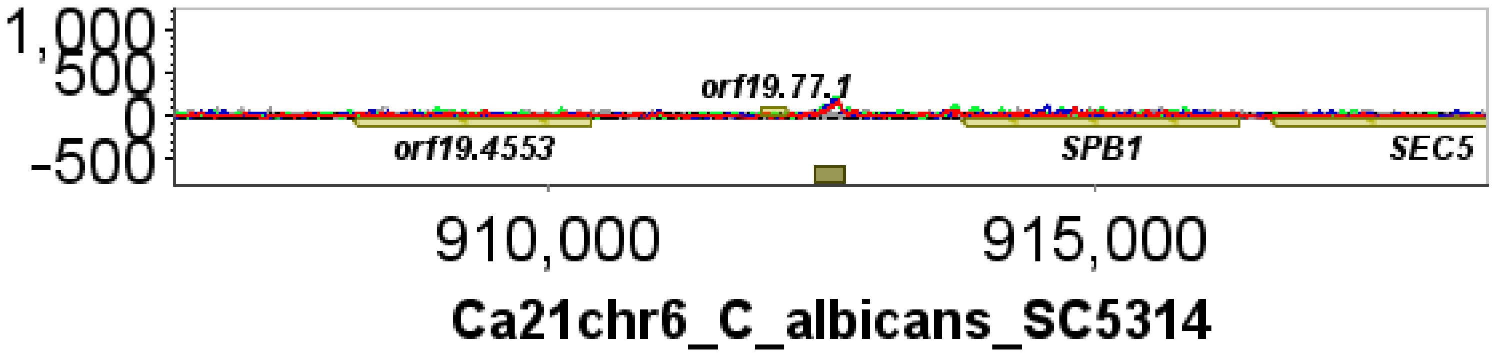

[5.17] Ca21chr5_C_albicans_SC5314:296947-308946 [+] [CFL4, orf19.1933, HST3, CFL5, orf19.1935]

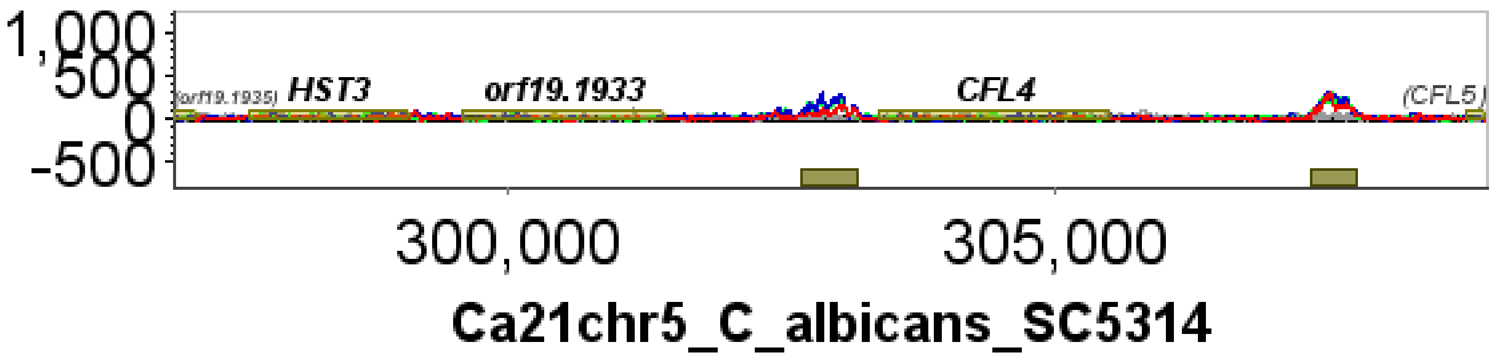

[5.15] Ca21chr2_C_albicans_SC5314:1736550-1748549 [+] [orf19.3621, YWP1, ANP1]

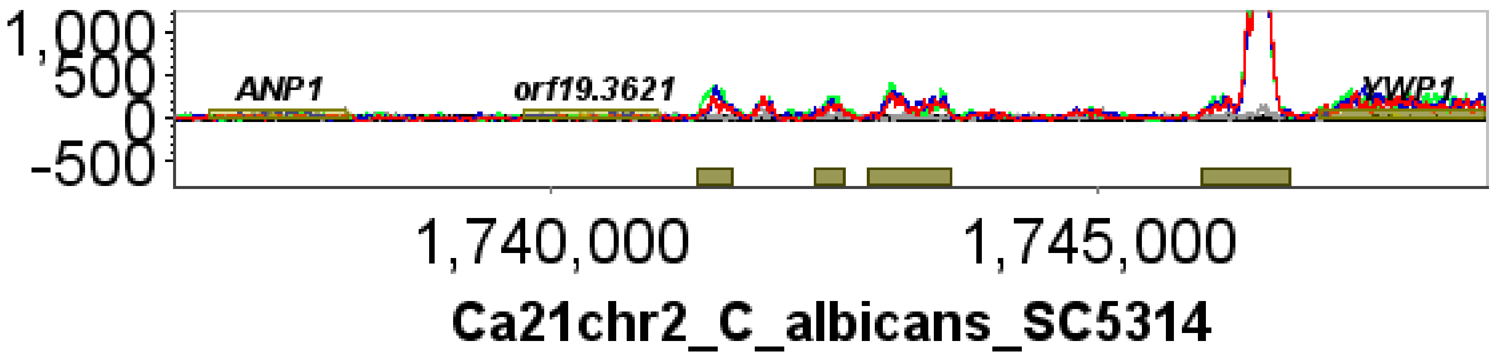

[5.13]   Ca21chr2_C_albicans_SC5314:1796313-1808312 [+]   [PIR1, orf19.223, BUD20, orf19.225]

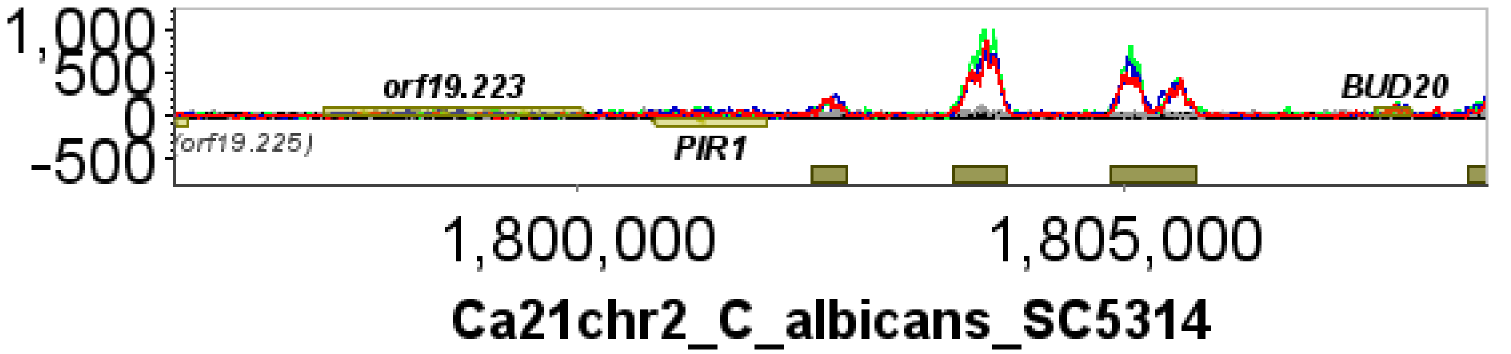

[5.09]   Ca21chr5_C_albicans_SC5314:714858-726857 [+]   [orf19.4349, orf19.2657, MED14]

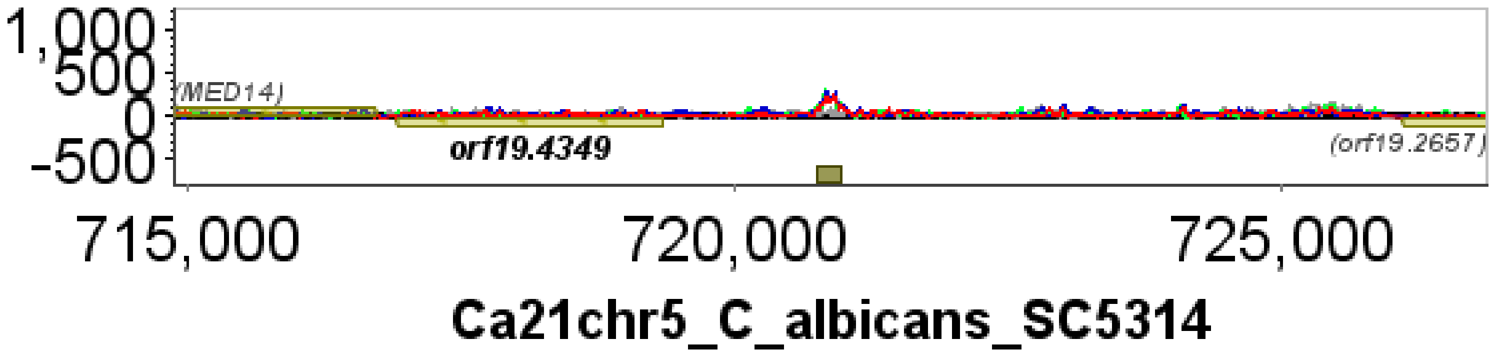

[5.09]   Ca21chr3_C_albicans_SC5314:950491-962490 [+]   [orf19.5910, TEC1, CMK1]

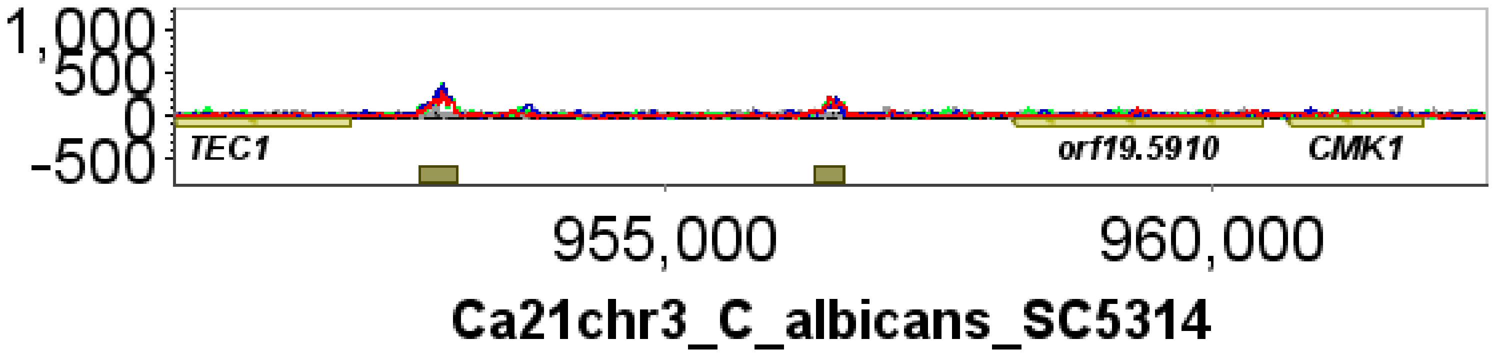

[5.09]   Ca21chr1_C_albicans_SC5314:3052954-3064953 [+]   [orf19.5026, tA(AGC)7, LCB2, MODF, MET3]

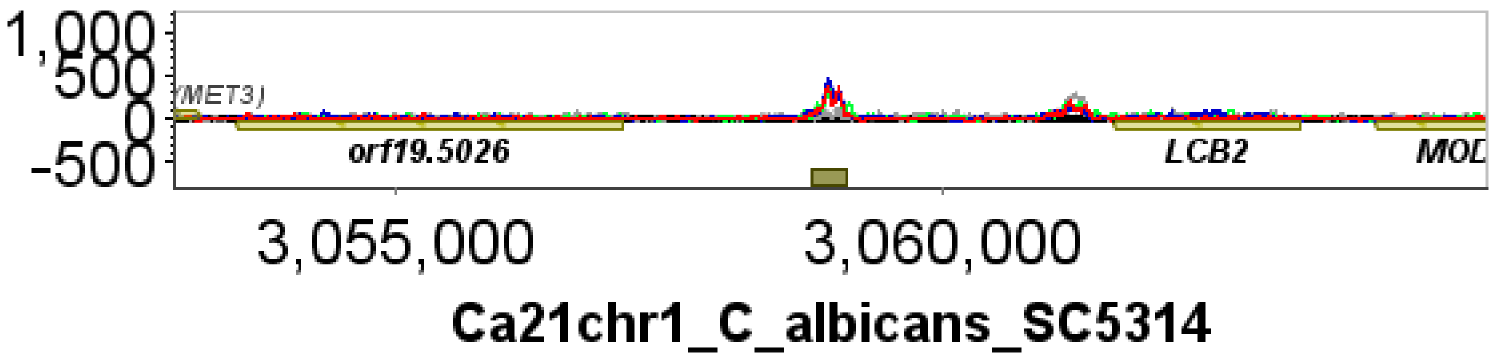

[5.08]   Ca21chr7_C_albicans_SC5314:718694-730693 [+]   [RBR3, LIP8]

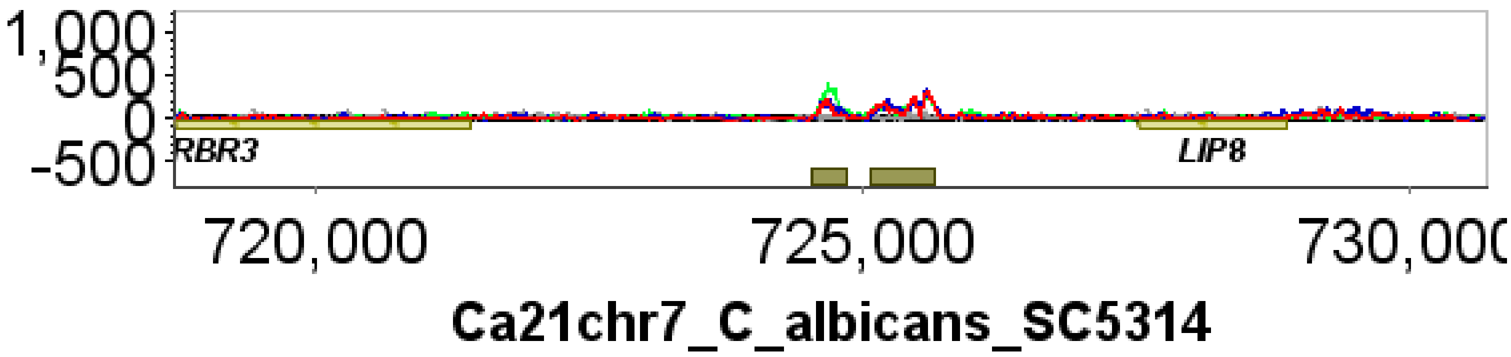

[5.08] Ca21chr1_C_albicans_SC5314:135249-147248 [+] [HGC1, ROT1]

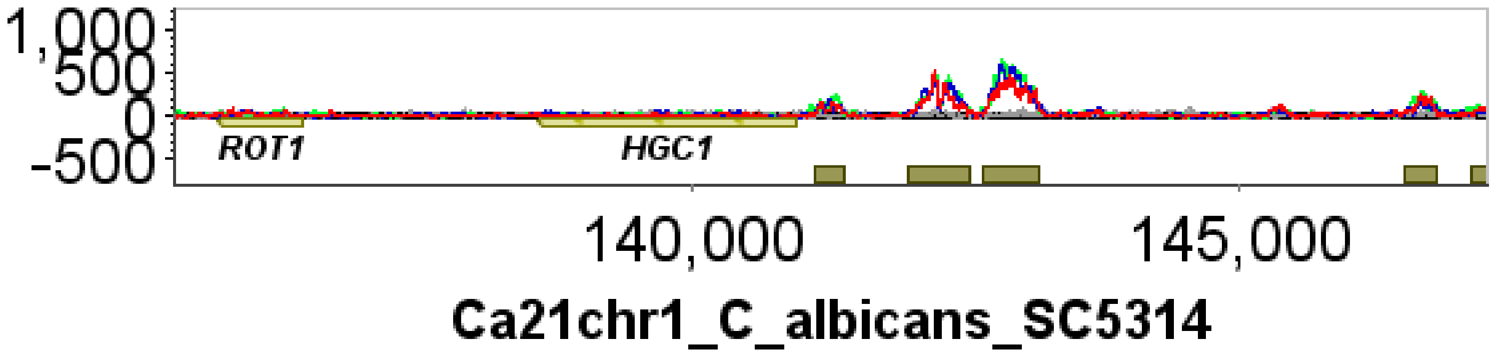

[5.06] Ca21chr3_C_albicans_SC5314:1719468-1731467 [+] [orf19.6736, TCC1]

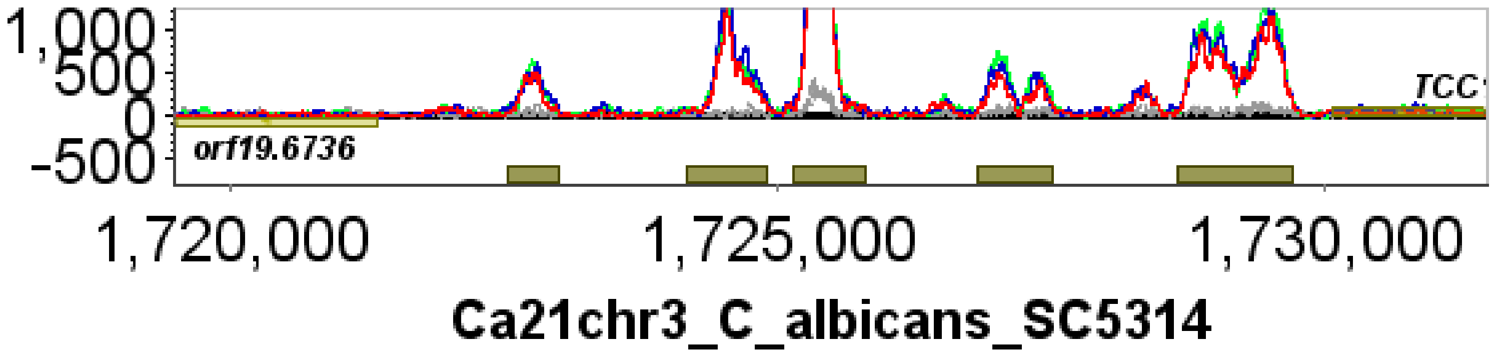

[4.99] Ca21chr5_C_albicans_SC5314:249894-261893 [+] [orf19.1958, orf19.1959, CYC3]

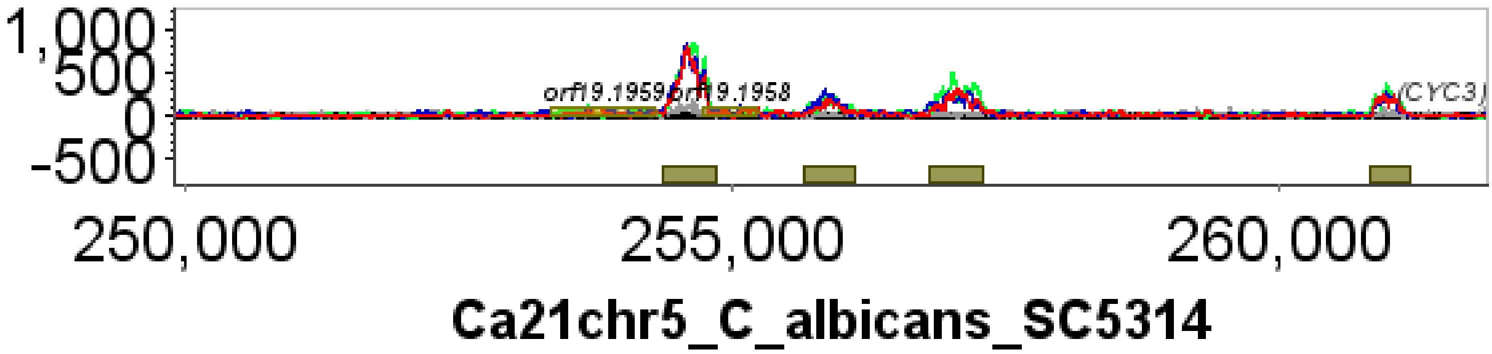

[4.98] Ca21chr5_C_albicans_SC5314:1111716-1123715 [+] [GRF10, orf19.4001, DUN1, orf19.3999]

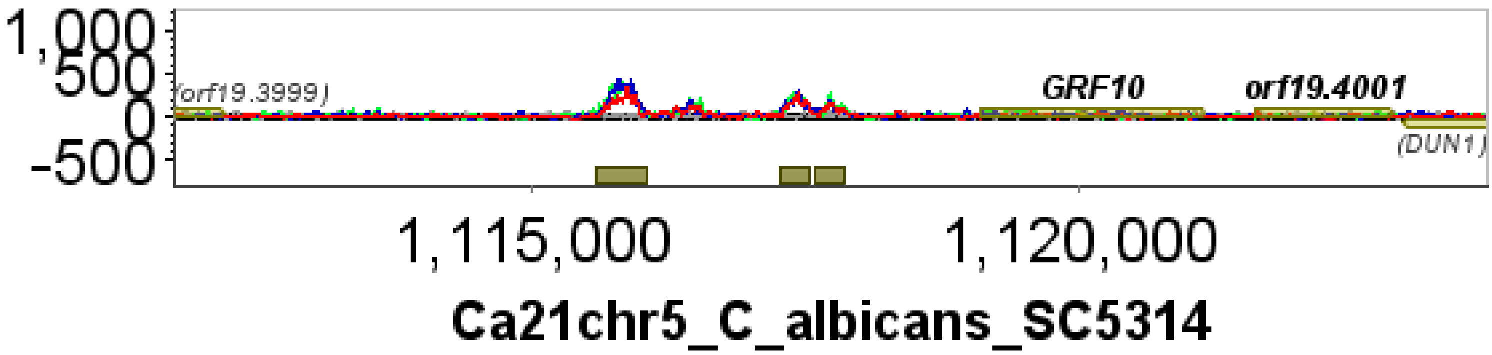

[4.9] Ca21chr1_C_albicans_SC5314:2933303-2945302 [+] [orf19.4972, orf19.4970, HYR1]

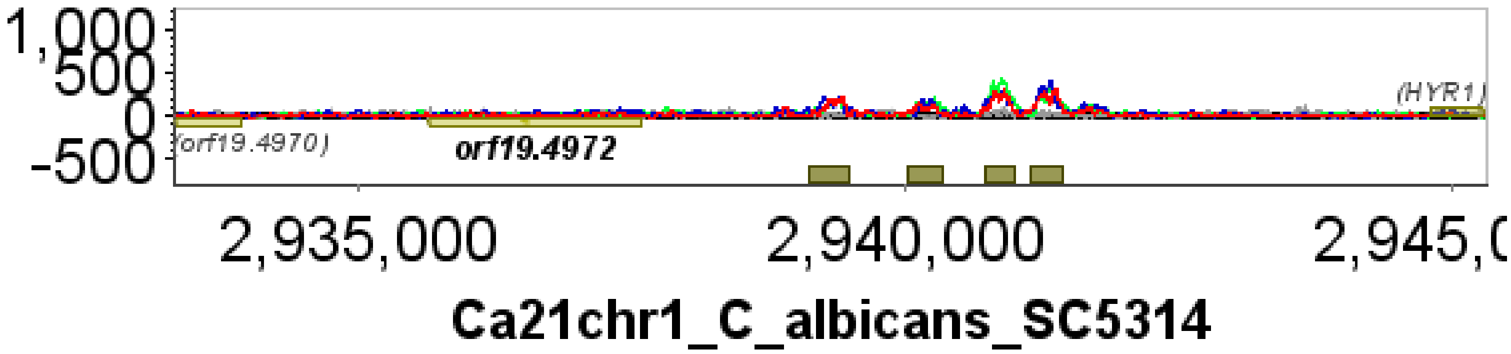

[4.89] Ca21chrR_C_albicans_SC5314:1368638-1380637 [+] [orf19.721, BCR1]

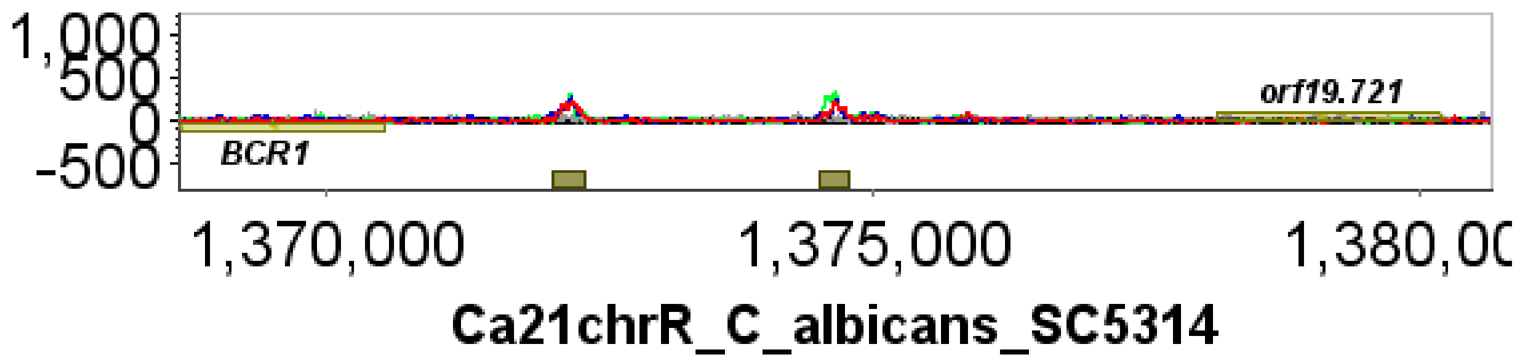

[4.86] Ca21chr5_C_albicans_SC5314:491638-503637 [+] [PTH2, orf19.4230, PTH1, THR4, orf19.4229]

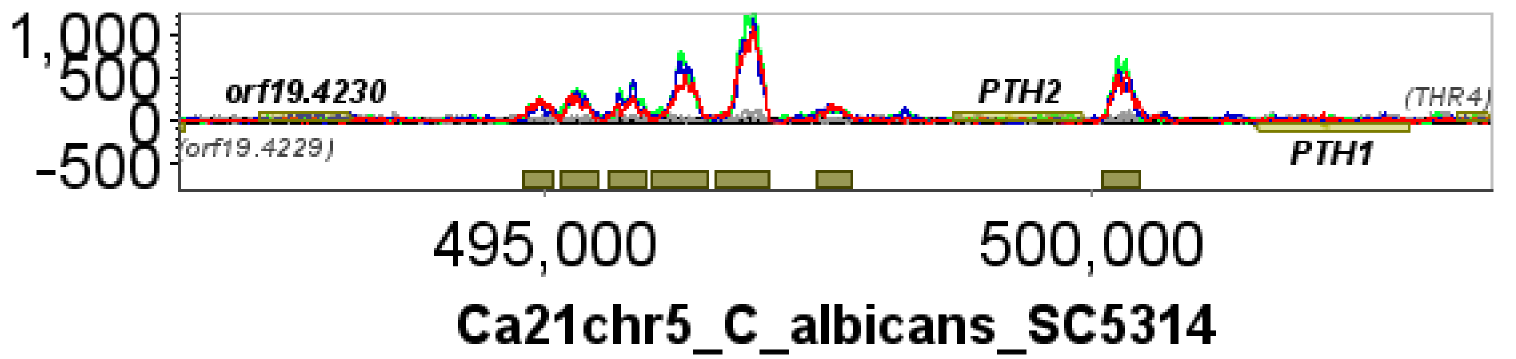

[4.84] Ca21chr4_C_albicans_SC5314:858281-870280 [+] [JEN2, orf19.5308, orf19.5306, RHD3, orf19.5304]

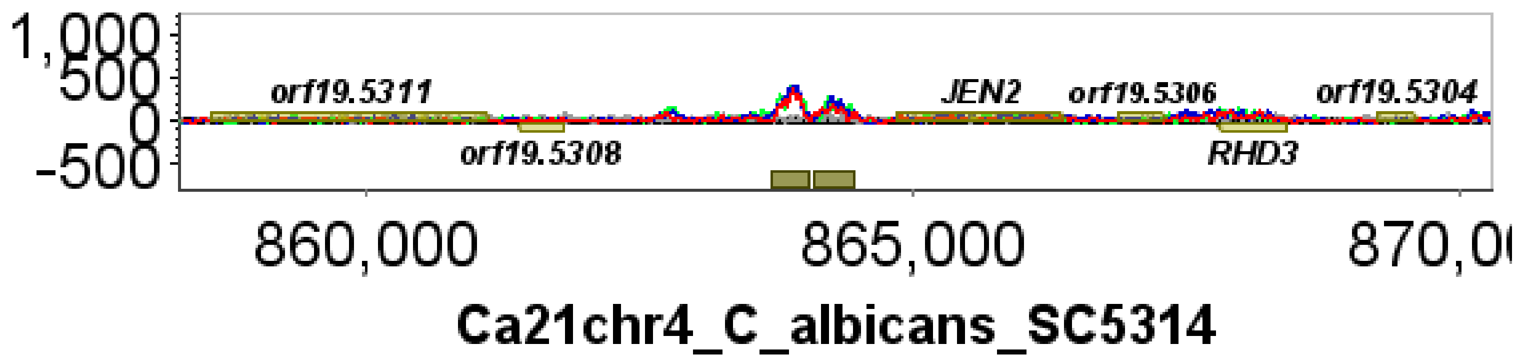

[4.8] Ca21chr1_C_albicans_SC5314:1352374-1364373 [+] [PBR1, orf19.6273, orf19.6272, orf19.6275, orf19.6271]

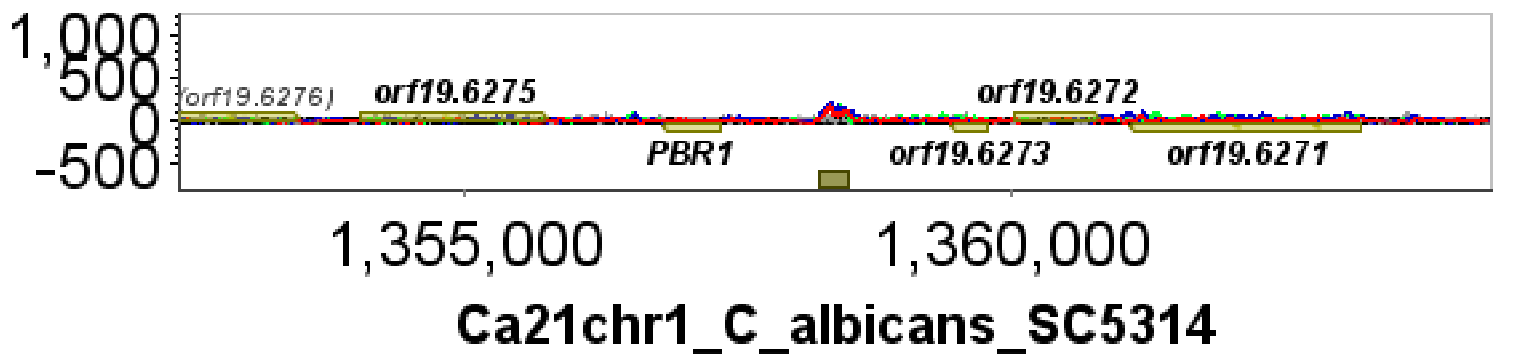

[4.76] Ca21chr1_C_albicans_SC5314:2048535-2060534 [+] [orf19.4793, orf19.4792, orf19.4795, orf19.4791]

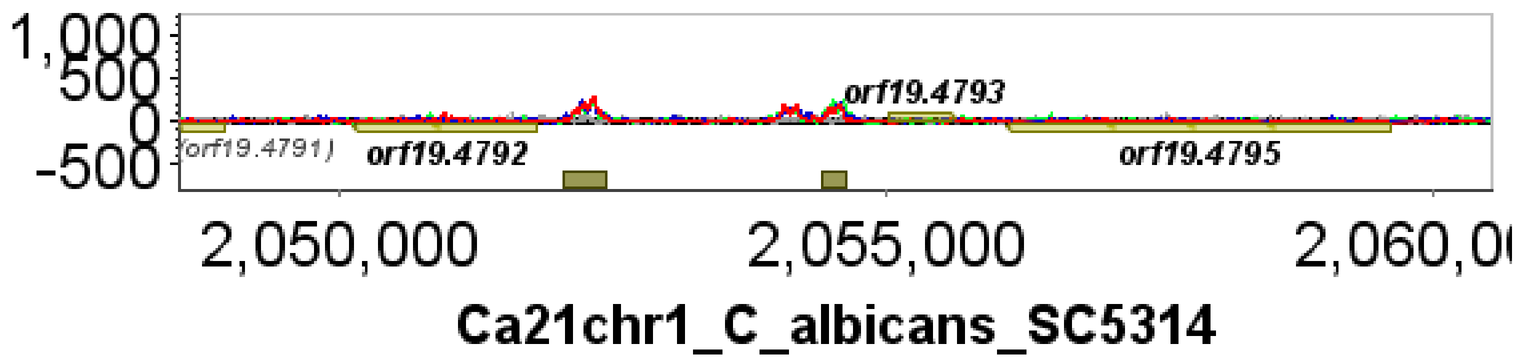

[4.72] Ca21chr5_C_albicans_SC5314:118191-130190 [+] [orf19.932, orf19.933, orf19.934]

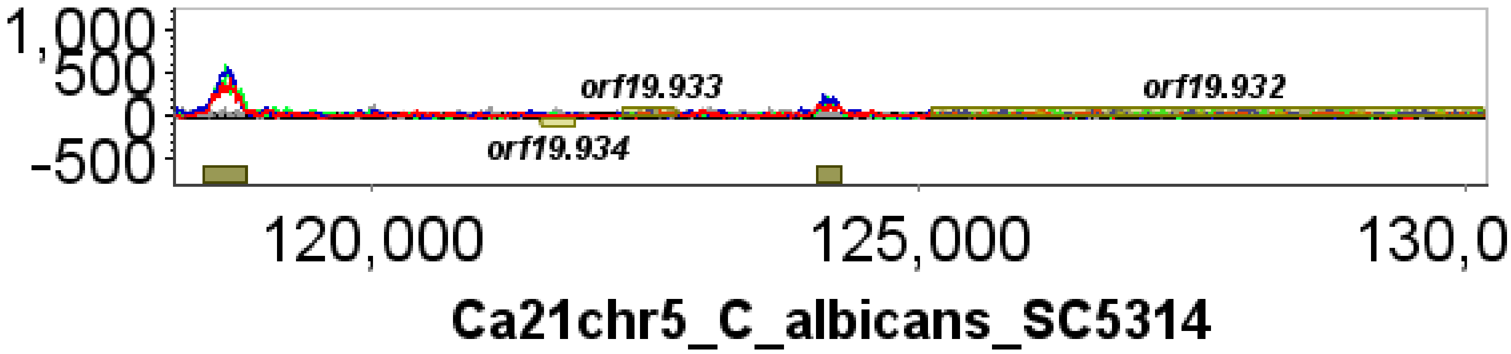

[4.71] Ca21chr1_C_albicans_SC5314:2046254-2058253 [+] [orf19.4792, orf19.4793, orf19.4791, orf19.4795]

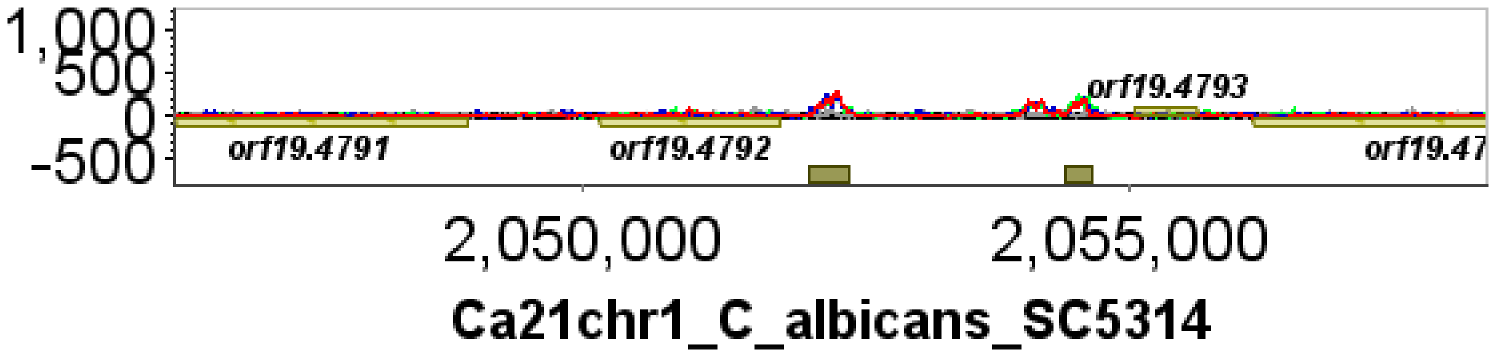

[4.69] Ca21chrR_C_albicans_SC5314:1308199-1320198 [+] [orf19.3868, orf19.3869, RPL7, ADE13]

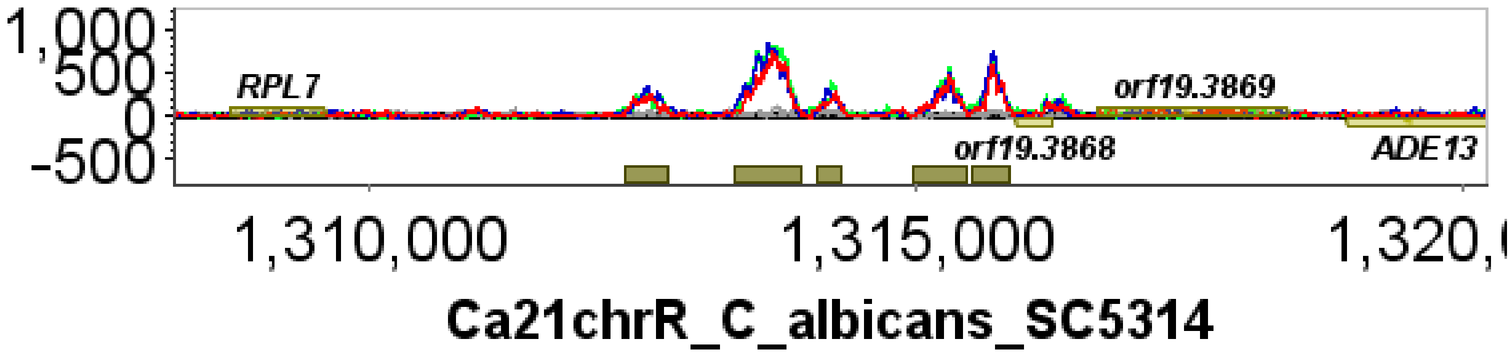

[4.61] Ca21chr1_C_albicans_SC5314:1520108-1532107 [+] [GAP4, orf19.4455, TAF145, RIA1]

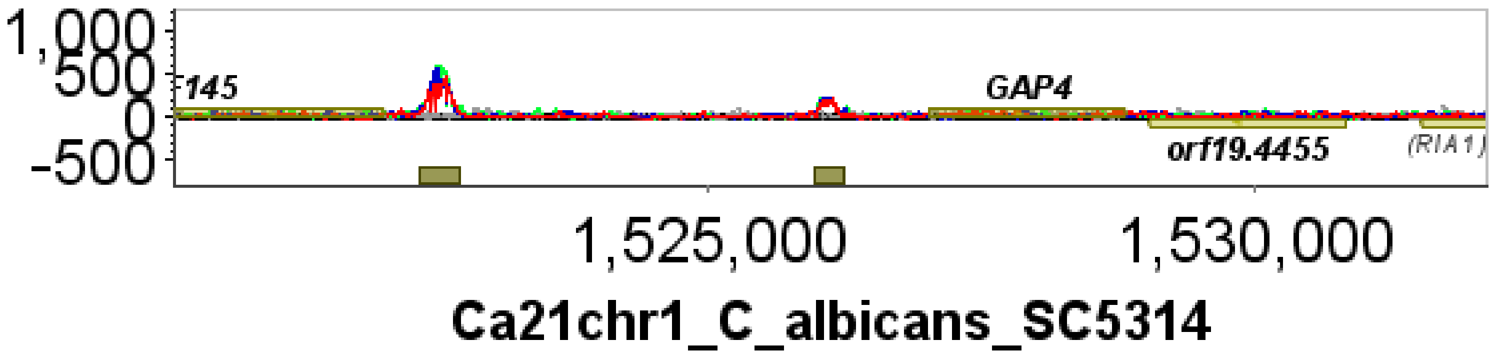

[4.56] Ca21chr5_C_albicans_SC5314:850079-862078 [+] [orf19.1105.3, PGA56, orf19.1105, orf19.3220, orf19.1106]

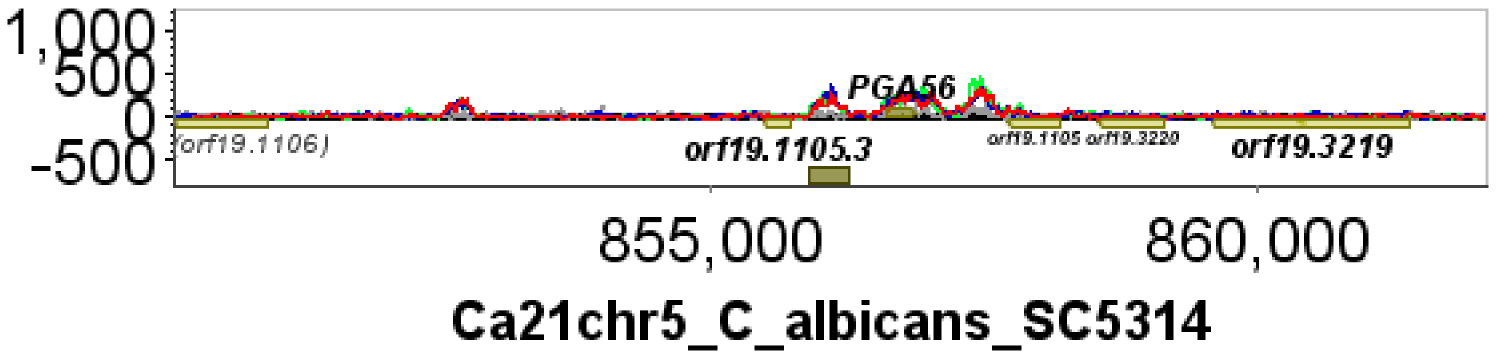

[4.53] Ca21chr7_C_albicans_SC5314:907247-919246 [+] [orf19.7151, orf19.7152, NRG1, orf19.7153]

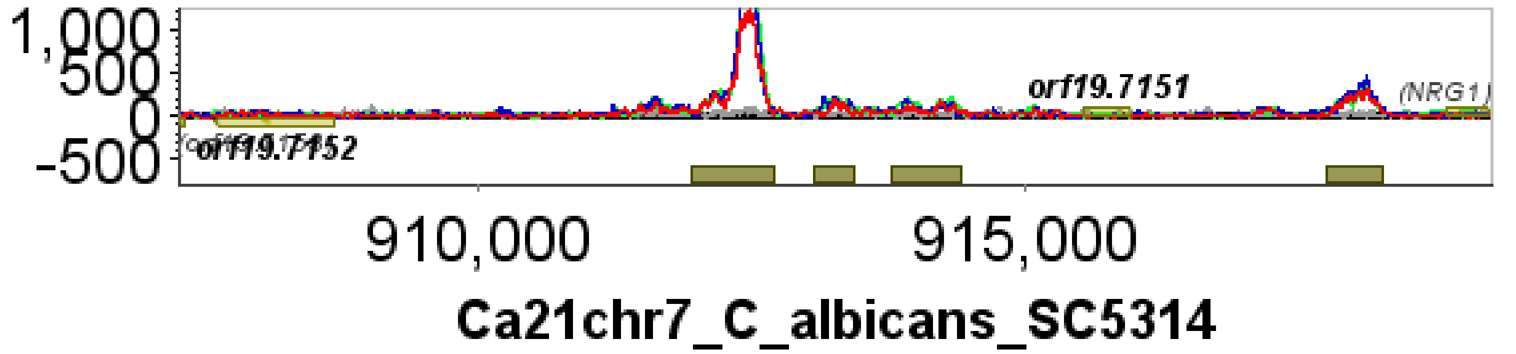

[4.52] Ca21chr3_C_albicans_SC5314:1208497-1220496 [+] [orf19.6983, orf19.6982, orf19.6981]

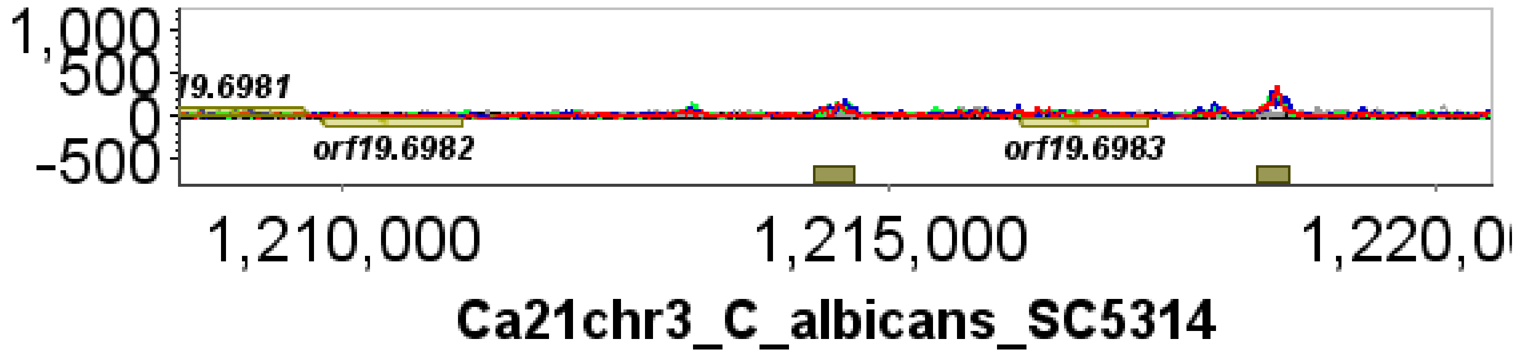

[4.52] Ca21chr2_C_albicans_SC5314:737072-749071 [+] [ADAEC, orf19.867]

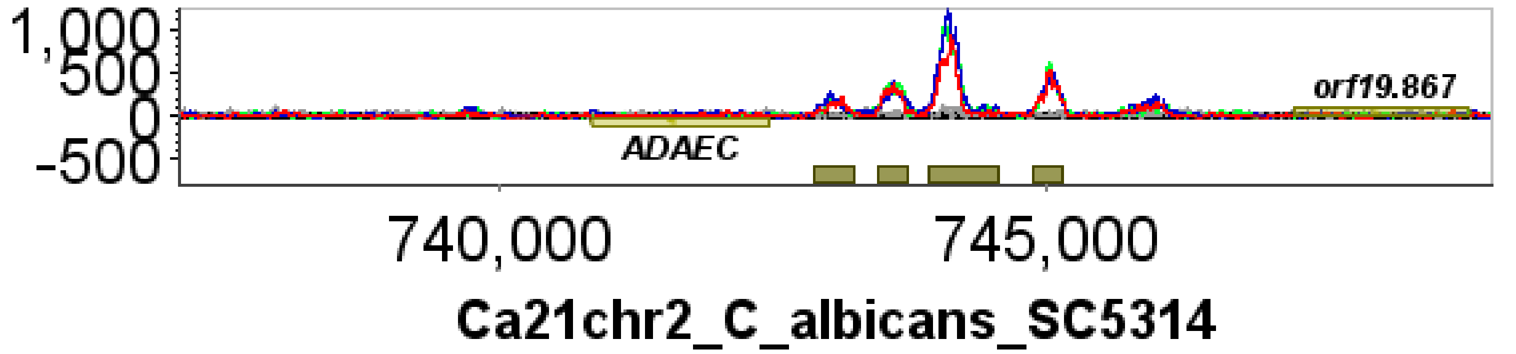

[4.52] Ca21chr1_C_albicans_SC5314:2934177-2946176 [+] [orf19.4972, HYR1]

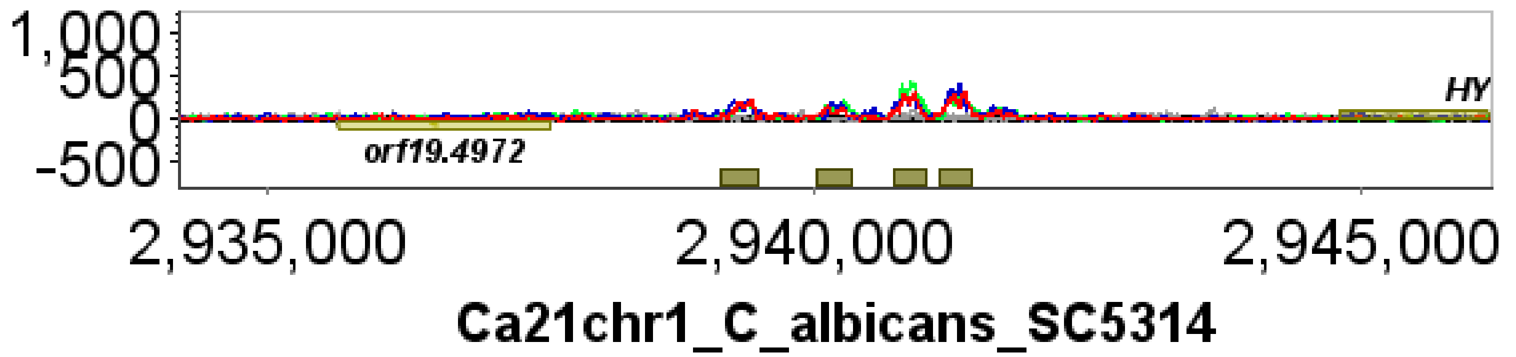

[4.52] Ca21chr1_C_albicans_SC5314:2616085-2628084 [+] [MED10, orf19.5269, orf19.5267, orf19.5269.1, orf19.5266]

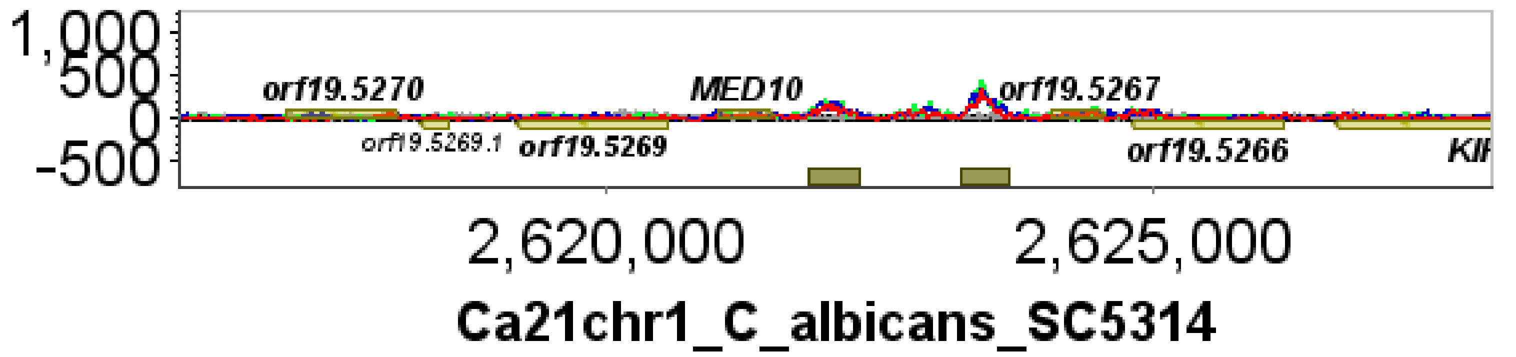

[4.5] Ca21chr5_C_albicans_SC5314:764656-776655 [+] [tE(UUC)5, orf19.2638, orf19.2639, orf19.2639.1, FUR1]

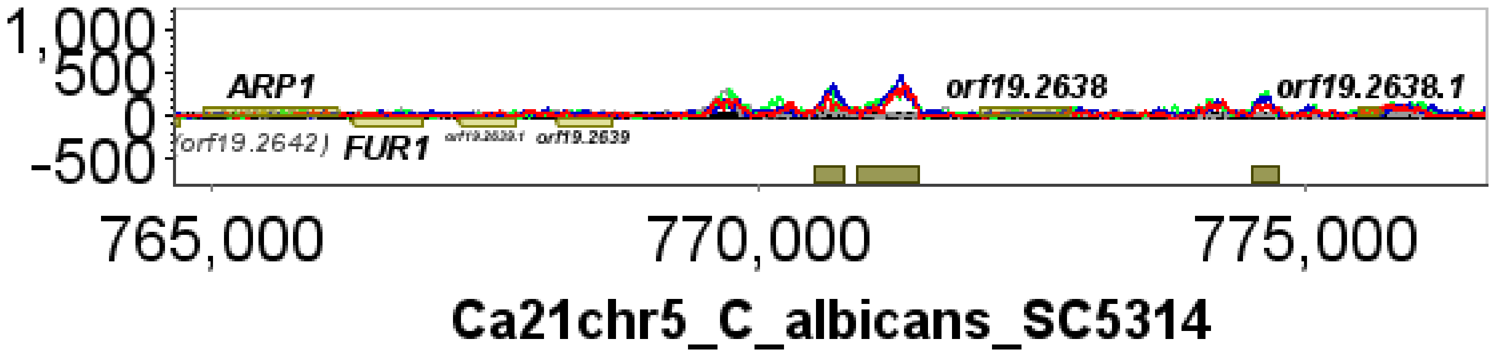

[4.48] Ca21chr7_C_albicans_SC5314:908085-920084 [+] [orf19.7151, NRG1, orf19.7152]

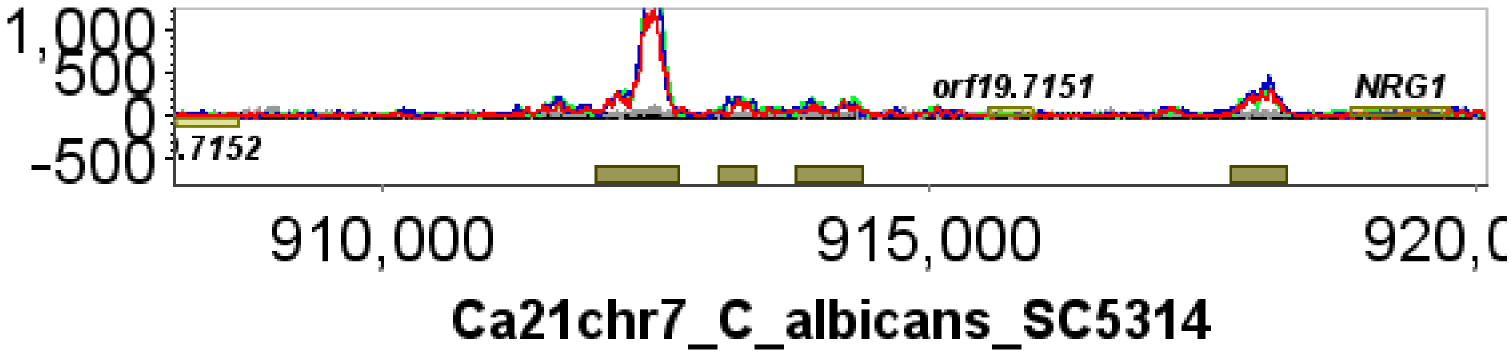

[4.46] Ca21chr4_C_albicans_SC5314:1518794-1530793 [+] [CZF1, CCT6, orf19.3125, orf19.3124]

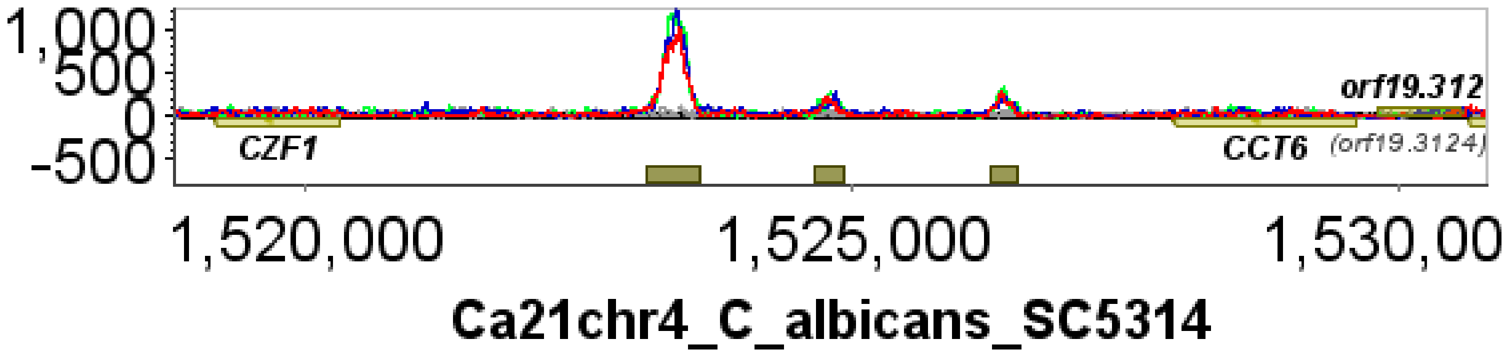

[4.38] Ca21chr2_C_albicans_SC5314:1570737-1582736 [+] [YVC1, orf19.2210, orf19.2208, orf19.2211, orf19.2212]

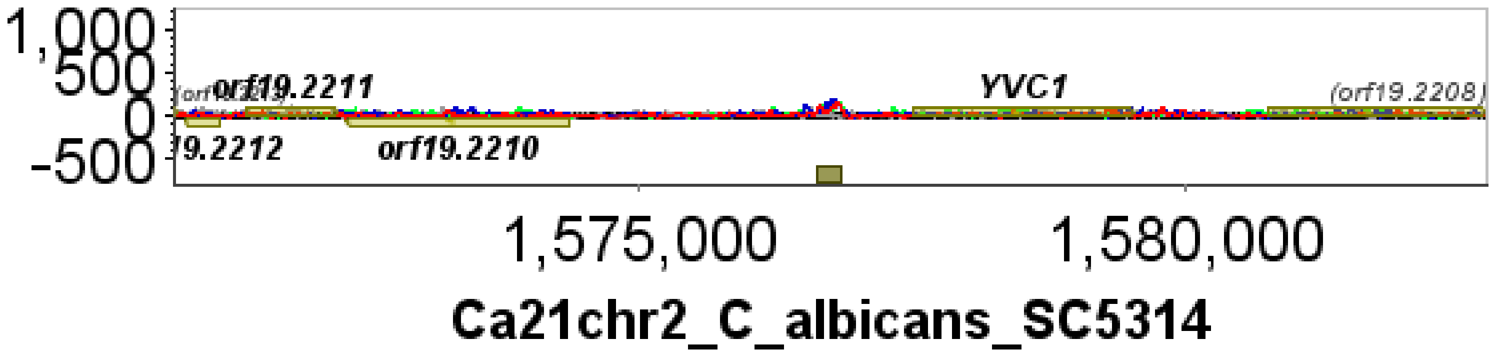

[4.36] Ca21chr5_C_albicans_SC5314:768644-780643 [+] [orf19.2638.1, snR40, orf19.2638, orf19.2637, tE(UUC)5]

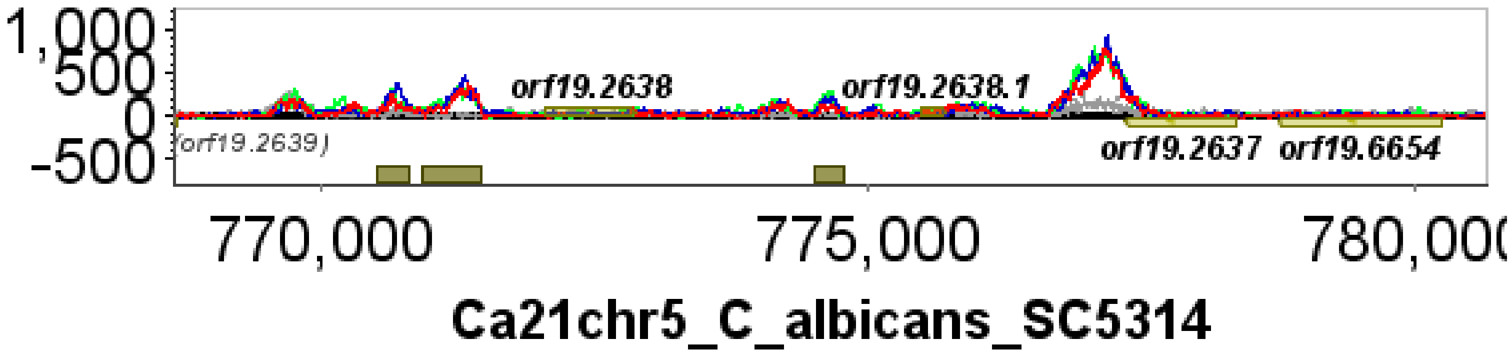

[4.31] Ca21chr2_C_albicans_SC5314:1416335-1428334 [+] [orf19.2247, ARE2, orf19.2246, YPT72, orf19.2249]

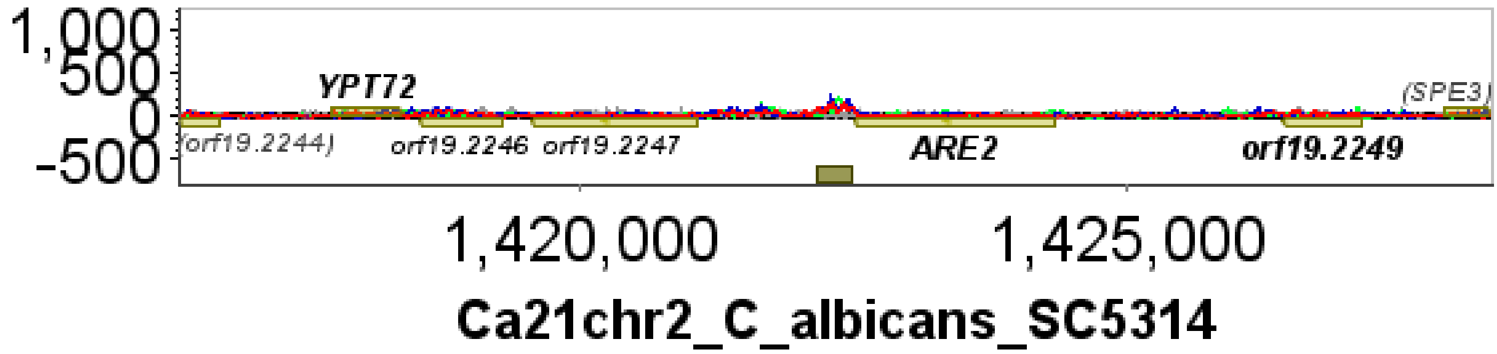

[4.29] Ca21chr1_C_albicans_SC5314:1228295-1240294 [+] [orf19.2457, SIP5, orf19.2459, orf19.2455]

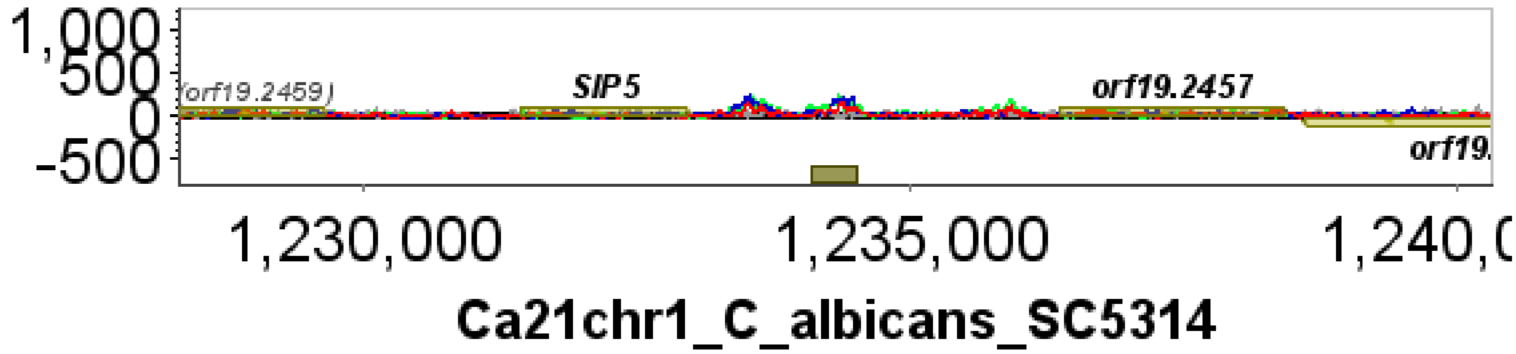

[4.29] Ca21chr1_C_albicans_SC5314:567030-579029 [+] [orf19.2962, orf19.2963, orf19.2961, orf19.2964, FRS2]

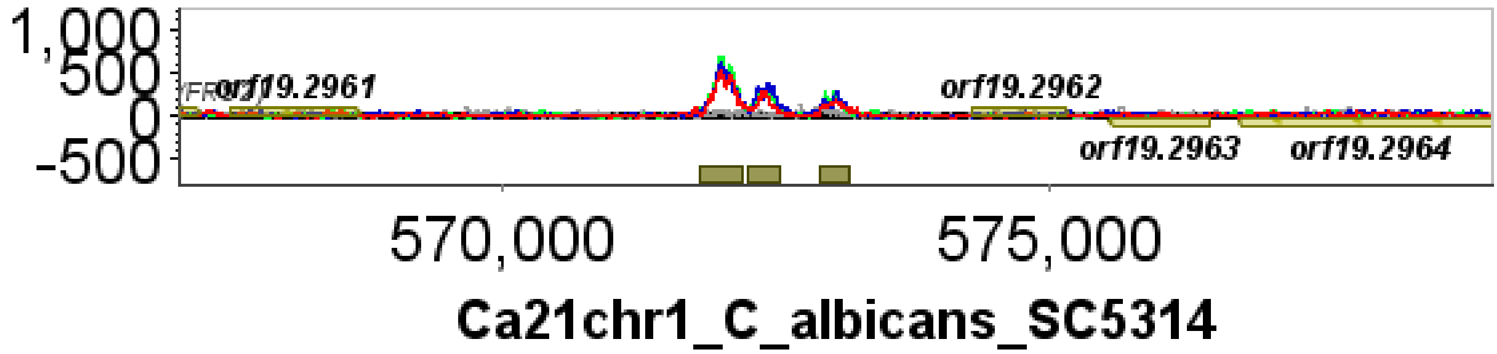

[4.17] Ca21chr2_C_albicans_SC5314:1075817-1087816 [+] [BUD14, AAT1, tQ(UUG)3, RPF2, orf19.3556]

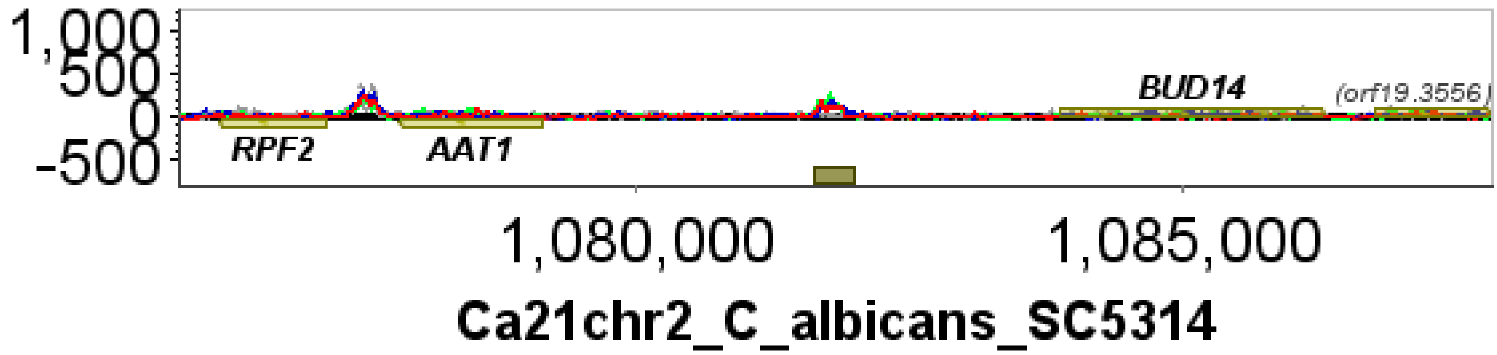

[4.16] Ca21chr4_C_albicans_SC5314:559924-571923 [+] [orf19.2725, orf19.2724]

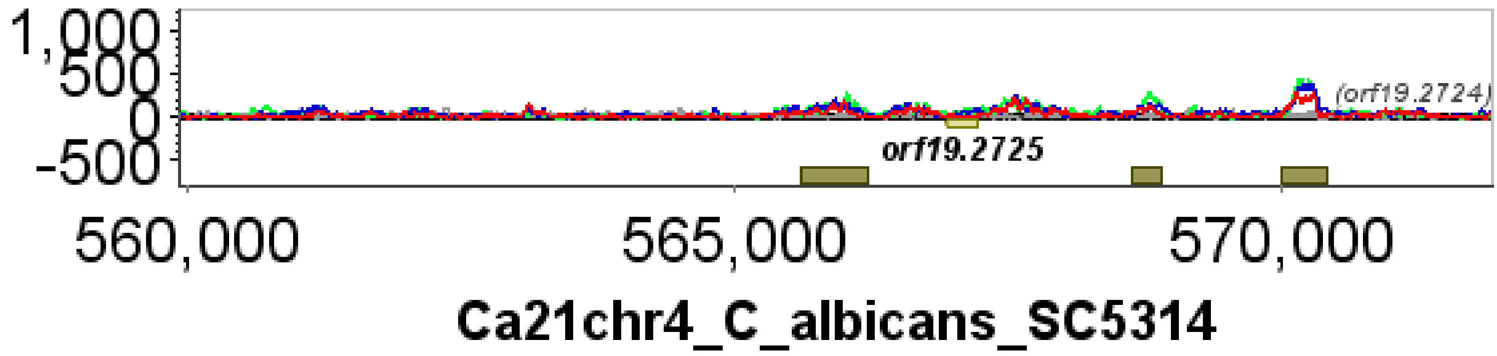

[4.16] Ca21chr3_C_albicans_SC5314:1215798-1227797 [+] [orf19.6983, orf19.6984]

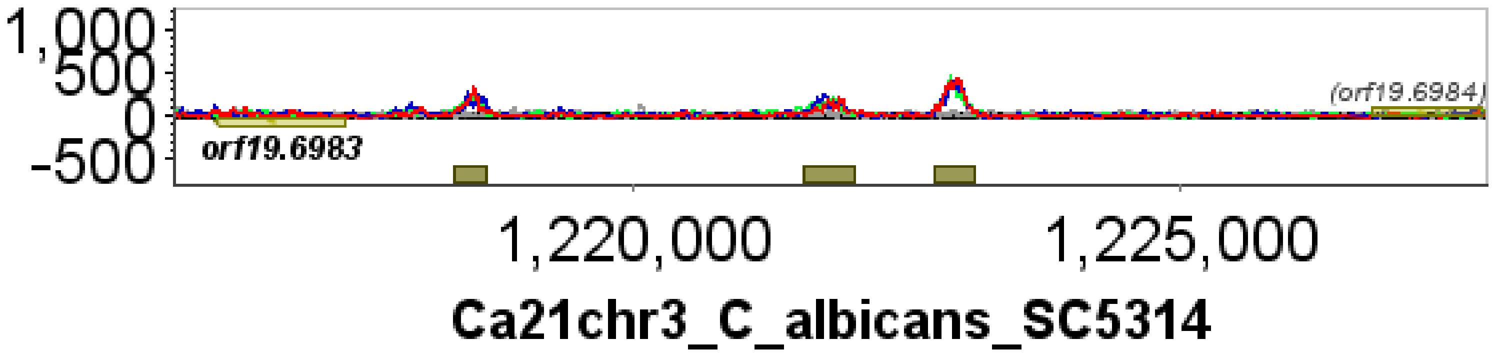

[4.14] Ca21chr4_C_albicans_SC5314:1520396-1532395 [+] [CCT6, orf19.3125, orf19.3124, orf19.3123.2]

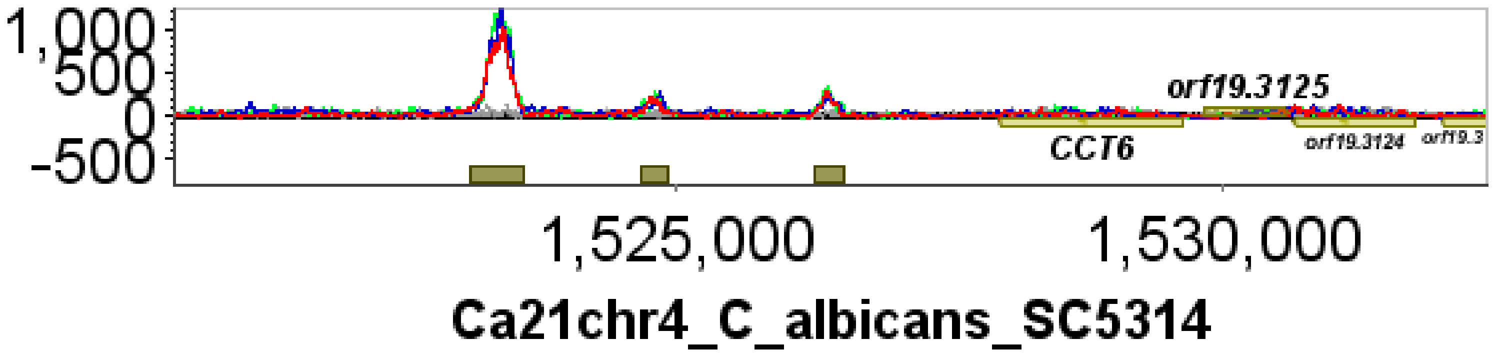

[4.1] Ca21chr2_C_albicans_SC5314:726533-738532 [+] [orf19.871, orf19.872]

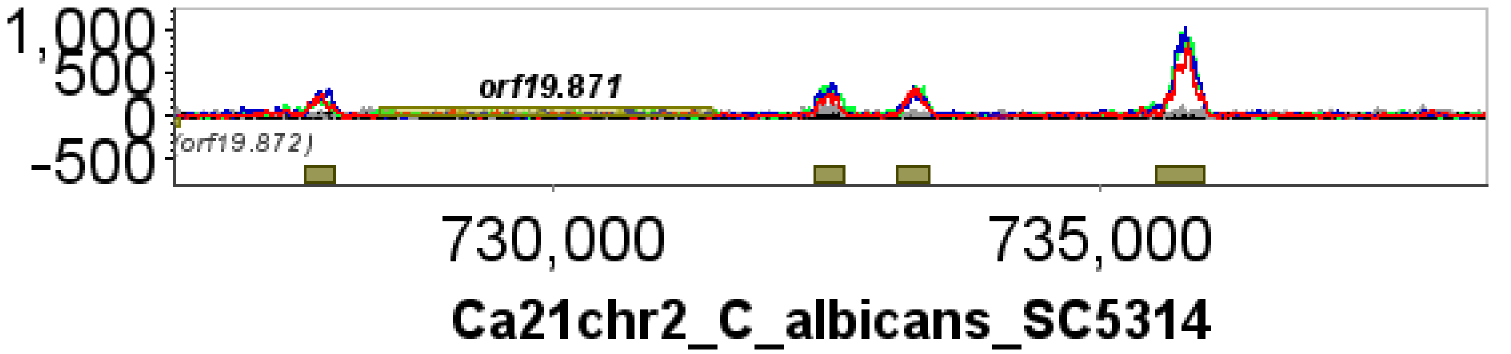

[4.08] Ca21chr5_C_albicans_SC5314:689413-701412 [+] [SUT1, orf19.4341, orf19.4340.1, orf19.4340, orf19.4346]

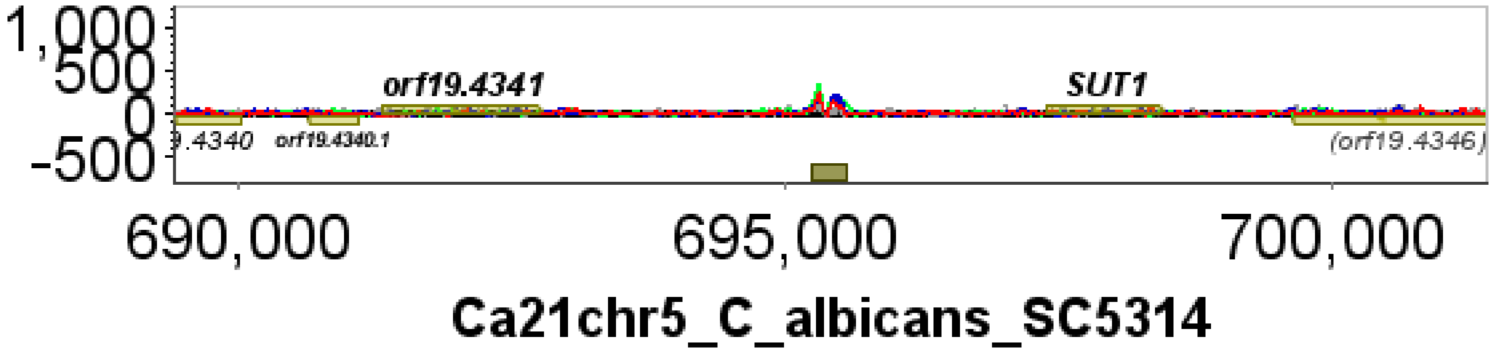

[4.01] Ca21chr1_C_albicans_SC5314:528905-540904 [+] [DIP5, tR(UCU)4, orf19.2943.5, PUT4, SCW4]

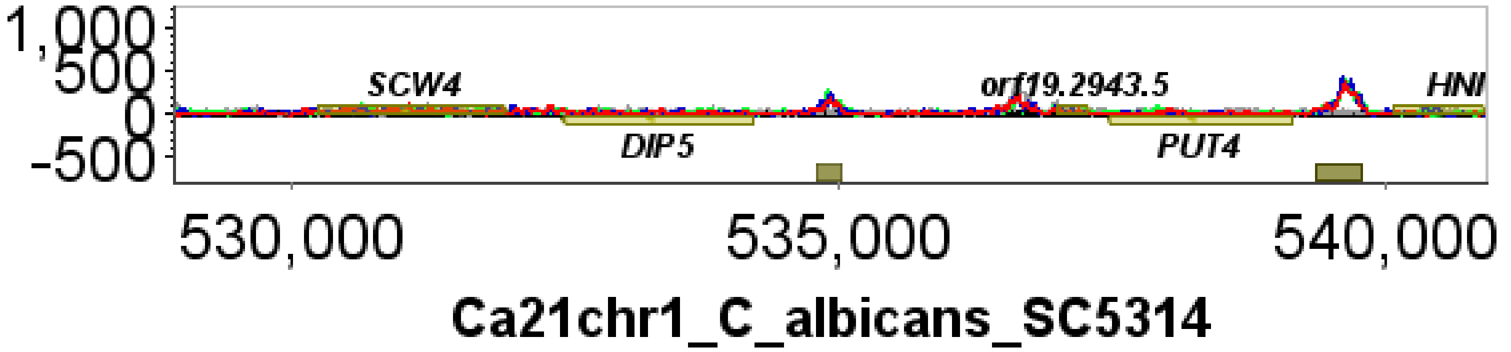

[3.99] Ca21chr4_C_albicans_SC5314:562778-574777 [+] [orf19.2725, orf19.2724]

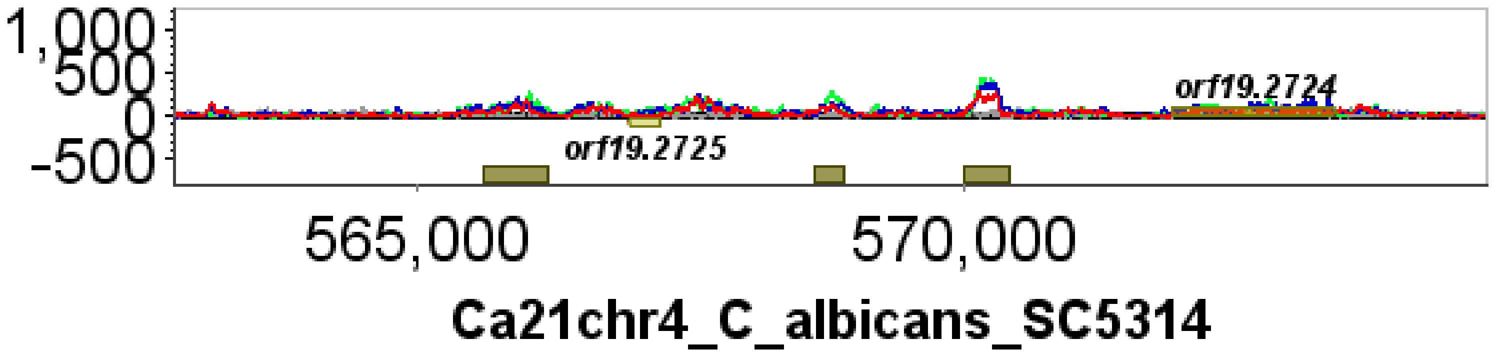

[3.8] Ca21chr4_C_albicans_SC5314:935223-947222 [+] [orf19.1409.3, orf19.1409.1, LYS4, orf19.1409.2]

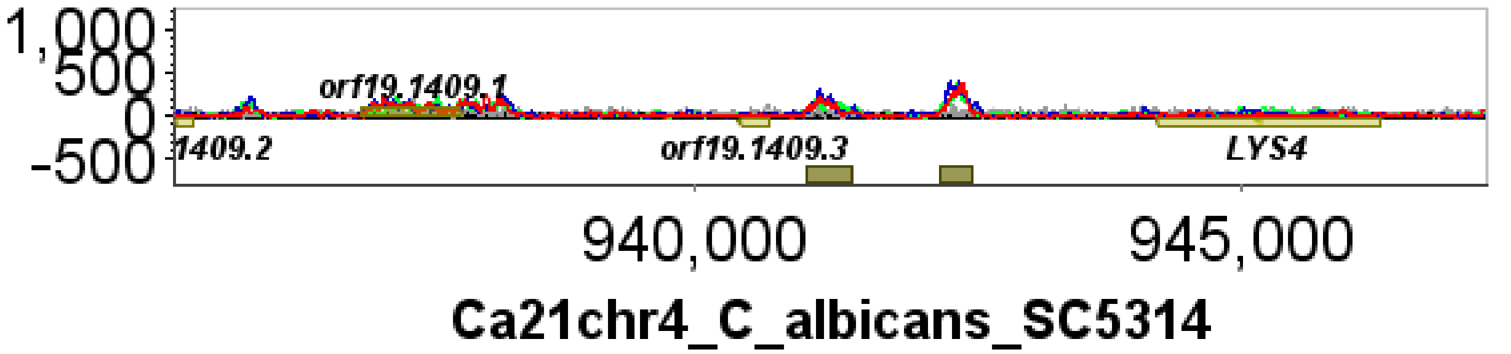

[2.99] Ca21chr2_C_albicans_SC5314:198217-210216 [+] [orf19.2002, HNM1, orf19.2001, REG1, orf19.2000]

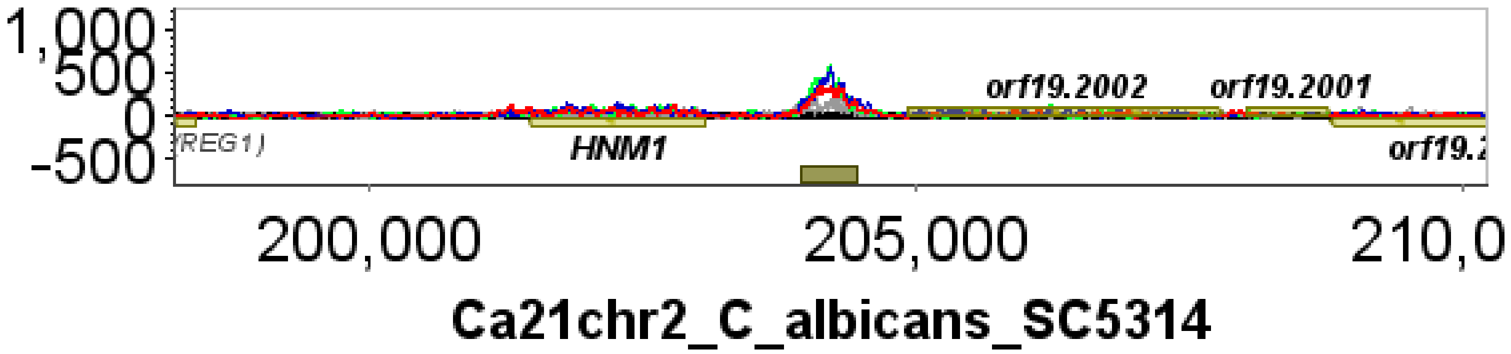

[2.96] Ca21chrR_C_albicans_SC5314:1456699-1468698 [+] [orf19.1852, HHT2, HHF22, SIW14, orf19.1849]

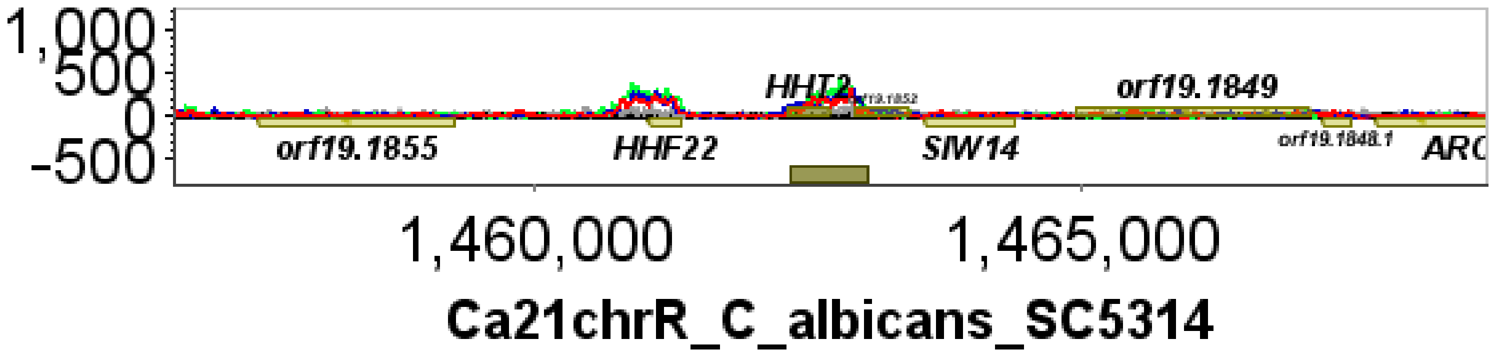

Supplement: Supporting Information [file supp_g3.115.024885_FileS2.pdf]
