# Supplementary material for: Identification and Characterization of Wor4, a New Transcriptional Regulator of White-Opaque Switching
Source: G3 (Bethesda). 2016 Jan 13;6(3):721–9. doi: 10.1534/g3.115.024885 (PMC4777133; doi:10.1534/g3.115.024885)
Supplement: Supporting Information [file supp_g3.115.024885_FigureS1.pdf]

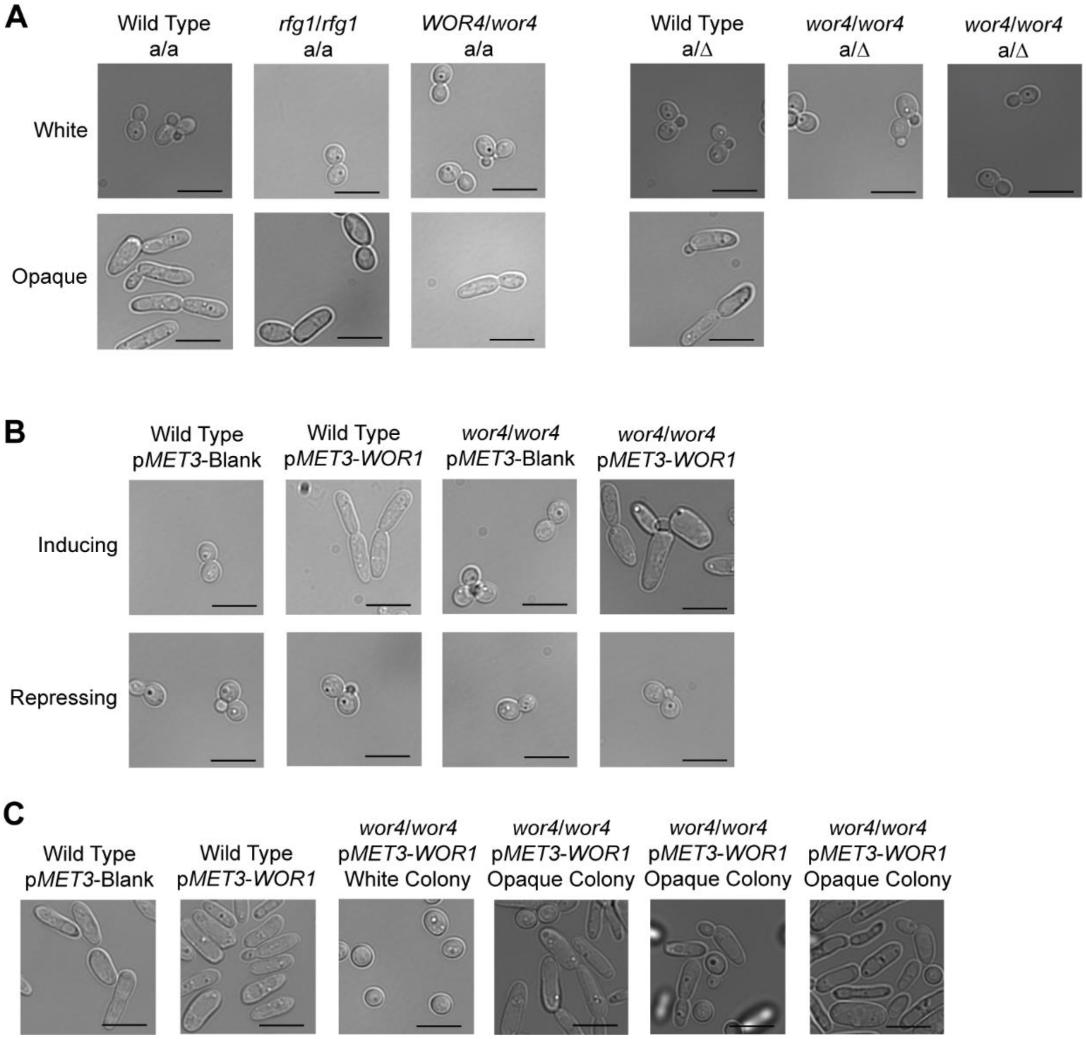

Figure S1: Single cell morphology of *wor4* and *rfg1* deletions. (a) Typical white and opaque cells for the *wor4* heterozygous deletion as well as the *wor4* and *rfg1* homozygous deletions as well as the matching wild type strains. (b) Typical cellular morphology for strains with the p*MET3* ectopic expression system driving *WOR1* or an empty control in the wild type and *wor4* deletion backgrounds on inducing or repressing media. (c) Opaque colonies from the WOR1 ectopic expression assay in (b) were restreaked to repressing media plates and the resulting colonies were then resuspended in water, diluted, and plated on repressing media and allowed to grow.

Images were taken of typical cells from one white and three opaque looking p*MET3-WOR1* *wor4/wor4* colonies as well as opaque colonies of the control strains. Scale bars are 10µm.
