# Supplementary material for: Identification and Characterization of Wor4, a New Transcriptional Regulator of White-Opaque Switching
Source: G3 (Bethesda). 2016 Jan 13;6(3):721–9. doi: 10.1534/g3.115.024885 (PMC4777133; doi:10.1534/g3.115.024885)
Supplement: Supporting Information [file supp_g3.115.024885_FigureS2.pdf]

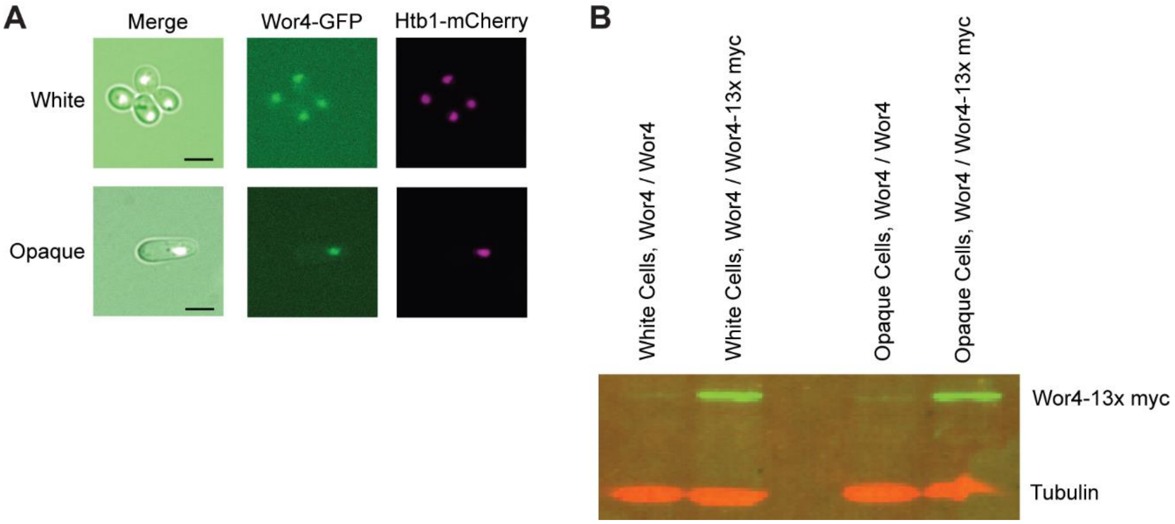

Figure S2: Wor4 localizes to the nucleus in both white and opaque cells. (a) Visualization of Wor4-GFP and Htb1-mCherry fusion proteins in both white and opaque cells. Merged images (DIC, GFP, mCherry), GFP fluorescence, and mCherry fluorescence are shown. Scale bar is 5µm. (b) Western blot of white and opaque strains either containing (Wor4/Wor4-13x myc) or lacking (Wor4/Wor4) c-terminally 13x myc tagged Wor4. Wor4 in green, α-tubulin loading control in red.
